# Prevalence and correlation of sarcopenia with Alzheimer’s disease: A systematic review and meta-analysis

**DOI:** 10.1371/journal.pone.0318920

**Published:** 2025-03-03

**Authors:** Chen Su, Sen Zhang, Qiandan Zheng, Jie Miao, Junhong Guo

**Affiliations:** 1 First Clinical Medical College, Shanxi Medical University, Taiyuan, China; 2 Department of Neurology, First Hospital, Shanxi Medical University, No.85, Jiefang South Street, Taiyuan, China; 3 Department of Neurology, First Hospital, Shanxi Medical University, No.85, Jiefang South Street, Taiyuan, China; University of Naples Federico II, ITALY

## Abstract

**Background:**

Sarcopenia, which is defined by a decline in skeletal muscle mass and strength associated with aging, is common among older individuals and presents considerable health dangers. Alzheimer’s disease (AD) is a prevalent degenerative brain condition linked to a decrease in cognitive function. The intersection of these conditions remains underexplored. The goal of this systematic review and meta-analysis was to establish the frequency of sarcopenia in individuals with AD and investigate the relationship between sarcopenia and AD.

**Methods:**

We performed an extensive review of literature databases, including PubMed, Embase, Web of Science, and the Cochrane Library, through April 2024. The inclusion criteria included studies that provided data on the frequency of sarcopenia in patients with AD or that examined the odds ratios (ORs) associated with these comorbidities. R Studio (4.3.1) was utilized for conducting the statistical analyses.

**Results:**

A total of 27 studies, comprising 3902 AD patients were included. In patients with AD, the combined occurrence of sarcopenia was 33.9%, with a confidence interval (CI) of 95%, ranging from 27.6% to 40.2%. Sarcopenia was found in 31.2% (95% CI: 0.223–0.402) and 41.9% (95% CI: 0.321–0.516) of patients with mild and moderate AD, respectively. The OR for the association between AD and sarcopenia was 2.670 (95% CI: 1.566–4.555), suggesting a robust correlation.

**Conclusion:**

Sarcopenia is highly prevalent in AD patients, highlighting the need for integrated care approaches to address both cognitive and physical health issues. Further research is needed to elucidate the pathophysiological links between AD and sarcopenia.

## Introduction

Sarcopenia, characterized by the progressive and generalized loss of skeletal muscle mass and strength, is a critical public health issue affecting older adults worldwide [1]. This condition not only contributes to physical frailty and disability but also increases the risk of falls, fractures, and mortality [2,3]. The etiology of sarcopenia is complex, encompassing various factors such as age-related shifts in muscle metabolism, persistent inflammation, hormonal changes, and diminished levels of physical activity [4–7]. Alzheimer’s disease (AD), the primary cause of dementia, is a degenerative brain condition that results in notable decreases in cognitive ability and daily functioning [8]. With the aging of the world’s population, the incidence of AD is projected to increase, creating substantial obstacles for healthcare systems and those providing care [9]. Additionally, AD is linked to various comorbidities such as nutritional deficiencies, metabolic disorders, and physical frailty, which further complicate the management and care of patients [10,11].

Recent research has highlighted a potential association between sarcopenia and AD, suggesting that these conditions may share common pathological mechanisms, such as chronic inflammation, oxidative stress, and metabolic dysfunction [12,13]. The interaction between sarcopenia and AD is particularly concerning, as it could exacerbate the progression of both conditions, leading to a vicious cycle of worsening physical and cognitive health [14,15]. Studies have shown that individuals with AD are at a greater risk of showing symptoms of sarcopenia than are those who are mentally sound [16]. The connection between these two factors is significant in terms of medical implications, since sarcopenia can worsen the existing functional limitations in AD, resulting in a greater decrease in the capacity to carry out everyday tasks and placing a heavier load on those providing care [17].

Although interest in this overlap is increasing, research on how common sarcopenia is in patients with AD and the connection between these two conditions is still scattered and inconclusive. Studies vary widely in terms of their diagnostic criteria for sarcopenia, assessment methods for AD, sample sizes, and demographic characteristics. The variability in data makes it difficult to come to definitive conclusions about the connection between sarcopenia and AD. A comprehensive examination and statistical analysis can fill these voids by combining the existing data, offering a more accurate assessment of the frequency of sarcopenia in patients with AD, and elucidating the degree of the relationship between these ailments.

At present, there has been no meta-analysis conducted on the occurrence and correlation of sarcopenia in AD. The goal of this systematic review and meta-analysis is to establish the frequency of sarcopenia in individuals with AD in various populations and environments and to investigate the relationship between sarcopenia and AD, considering factors such as age, gender, and the extent of cognitive decline.

## Methods

### Study selection and selection criteria

We followed the recommendations outlined in the Preferred Reporting Items for Systematic Reviews and Meta-Analyses (PRISMA) 2020 statement for conducting systematic reviews and meta-analyses. There is no registered protocol for this review. We thoroughly searched electronic databases such as PubMed, Embase, Web of Science, and Cochrane Library to find relevant studies published until April 1, 2024.The search formula utilized included terms such as “sarcopenia”, “muscle loss”, “muscle weakness”, “muscular atrophy”, “Alzheimer*”, and “dementia”. Inclusion criteria for studies required an observational design (cross-sectional, case-control, or cohort), subjects diagnosed with both sarcopenia and AD, and data on sarcopenia prevalence in AD subjects or the correlation between sarcopenia and AD. The criteria for exclusion included studies that lacked adequate data for analysis or lacked clear diagnostic criteria for sarcopenia, articles not written in English, unavailable full text, and articles categorized as review articles, editorials, case reports, or conference abstracts.

### Data extraction

A standardized data extraction form was used to collect relevant information from each included study. The form collected information on study features, participant characteristics, diagnostic standards, frequency rates, and association metrics. Two reviewers independently conducted data extraction to ensure accuracy, resolving discrepancies through discussion or consultation with a third reviewer. The study primarily focused on determining the frequency of sarcopenia in patients with AD and examining the correlation between AD and sarcopenia, which was quantified using odds ratios (ORs) and a 95% confidence interval (CI). The results were adjusted for different confounding factors.

### Research quality assessment

Two researchers independently evaluated the quality of each study. Cohort studies were assessed using the Newcastle–Ottawa Scale (NOS), while cross-sectional studies were evaluated with the Agency for Healthcare Research and Quality (AHRQ) tool. Cohort or case-control studies received a maximum score of 9 points, and cross-sectional studies received a maximum score of 6 points. Higher scores represent better methodological quality (S1 and S2 Tables). The two researchers resolved their differences through conversation.

### Statistical analyses

Statistical analyses were conducted via R Studio version 4.3.1. In cases of significant heterogeneity in the results of the meta-analysis (I^2^ >  50%, p < 0.05), a random-effects model was used for a more conservative overestimation. Subgroup analyses were conducted to explore variations in results based on study region, study type, diagnostic criteria for sarcopenia and AD, methods of muscle mass assessment, source of participants, age, gender, and body mass index (BMI). We conducted subgroup analyses of sarcopenia prevalence across different AD stages based on staging criteria and sarcopenia diagnostic standards. Univariate meta-regression analyses were conducted to investigate potential sources of heterogeneity, including mean age, gender, diagnostic criteria for AD, diagnostic criteria for sarcopenia, muscle mass measurements, participant origin, study region, and study type. Multivariate meta-regression analysis was performed based on the following criteria: (1) variables with a p value < 0.10 in the univariate meta-regression analysis were included, and (2) variables exhibiting collinearity were excluded. Publication bias was assessed via Begg’s test and Egger’s test (p < 0.05 was considered significant) [18,19]. Sensitivity analysis was performed by sequentially excluding individual studies to evaluate the stability and robustness of the pooled estimates. Studies lacking core variables were excluded, and the reasons for exclusion were recorded. Studies with missing BMI data were grouped into a separate “Unknown” category to evaluate the potential impact of incomplete data. The significance level was set at bilateral P < 0.05.

## Results

### Search process

**Fig 1:**
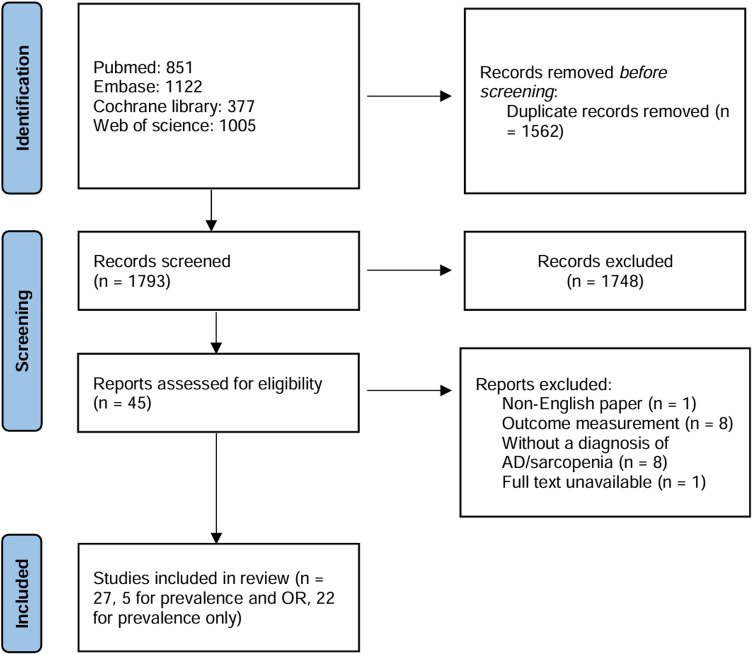
The flowchart of research screening. AD, Alzheimer’s disease; OR, odds ratio.

The flowchart of the literature screening process is shown in Fig 1. After removing duplicate articles, a total of 1793 articles were gathered from the database. After screening, 27 articles were included in the analysis [14,16,17,20–43]. Five articles included information on both the occurrence of sarcopenia in AD patients and the OR between sarcopenia and AD patients [14,27,31,36,38], whereas the other 22 articles focused only on the prevalence. S3 Table showed all studies identified in the literature search after the exclusion of duplicates and gives reasons for their exclusion from the meta-analysis.

### Study characteristics

All the data extracted from the primary research sources are shown in S4 Table. Table 1 provides a summary of the characteristics of the studies included in the analysis. Studies were conducted in Asia, Europe, and America. The majority of the studies were cross-sectional, with only one being a cohort study. The subjects were categorized as community, hospital, or both. Most of the standards for sarcopenia are based on the Asian Working Group for Sarcopenia (AWGS) or the European Working Group on Sarcopenia in Older People (EWGSOP1) or its revised version (EWGSOP2). Most studies used the Diagnostic and Statistical Manual of Mental Disorders (DSM), guidelines from the National Institute on Aging-Alzheimer’s Association (NIA/AA), or the National Institute of Neurological and Communicative Disorders and Stroke and the Alzheimer’s Disease and Related Disorders Association (NINCDS-ADRDA) for diagnosing AD. One study employed the Ministry of Health criteria for AD diagnosis. The prevalence of sarcopenia among patients with mild to moderate AD was reported in nine studies, whereas one study documented the occurrence of sarcopenia in individuals with severe AD.

**Table 1:** Characteristics of studies included in the meta-analysis.

Study	Study region	Study design	Sample size	Study population	Age (mean ± SD)	Male (%)	BMI (mean ± SD)	Diagnostic criteria of sarcopenia	Diagnostic criteria of AD
Yusuke Ogawa 2018	Japan	Cross‐sectional	352	Hospital participants	81.8 ± 5.2	37.5%	22.5 ± 3.3	AWGS	DSM-5
Giulia Bramato 2022	Italy	Cross‐sectional	130	Hospital participants	70.7 ± 8.5	45.4%	26.7 ± 4.1	EWGSOP1 EWGSOP2	DSM-5
Shanwen Liu 2023	China	Cross‐sectional	112	Hospital participants	71.6 ± 8.1	0.0%	Unknown	AWGS	NIA/AA
T Sugimoto 2022	Japan	Cross‐sectional	1181	Hospital participants	77.7 ± 5.6	36.2%	Unknown	EWGSOP2	NIA/AA
Ai Kimura 2018	Japan	Cross‐sectional	205	Hospital participants	77.2 ± 5.1	36.6%	Unknown	AWGS	NIA/AA
Cemile Özsürekci 2019	Turkey	Cross‐sectional	76	Hospital participants	78.9 ± 6.4	43.6%	Unknown	EWGSOP2	NIA/AA
Pelin Unsal 2023	Turkey	Cross‐sectional	253	Hospital participants	79.8 ± 6.5	41.9%	27.3 ± 5.6	EWGSOP2	DSM-5
Liss Elin Larsson 2023	Sweden	Cross‐sectional	368	Hospital participants	59.0 ± 7.3	41.3%	24.5 ± 3.8	EWGSOP2	DSM-5
Zekeriya Ülger 2022	Turkey	Cross‐sectional	221	Hospital participants	73.9 ± 7.0	35.3%	27.8 ± 5.7	EWGSOP2	DSM-5
Daisuke Hirose 2016	Japan	Cross‐sectional	107	Hospital participants	79.6 ± 5.9	48.6%	23.1 ± 3.3	AWGS	DSM-5
Fatma Sena Dost 2022	Turkey	Cross‐sectional	662	Hospital participants	73.6 ± 7.5	32.6%	Unknown	EWGSOP2	NIA/AA
Akito Tsugawa 2017	Japan	Cross‐sectional	216	Hospital participants	82.5 ± 5.1	40.7%	22.6 ± 3.4	AWGS	DSM-5
Xiaofen Weng 2023 (a)	China	Cross‐sectional	79	Hospital participants	73.3 ± 6.5	31.6%	23.2 ± 3.3	AWGS	NIA/AA
Taichi Demura 2023	Japan	Cross‐sectional	95	Hospital participants	80.9 ± 6.8	42.1%	22.8 ± 2.7	AWGS	DSM-5
Taiki Sugimoto 2016	Japan	Cross‐sectional	418	Hospital participants	77.3 ± 7.0	33.3%	21.8 ± 3.0	EWGSOP1	NINCDS-ADRDA
Xiaofen Weng 2023 (b)	China	Cross‐sectional	176	Hospital participants	71.8 ± 6.5	36.9%	23.4 ± 3.2	AWGS	NIA/AA
L Tay 2018	Singapore	Cross‐sectional	108	Community participants	76.7 ± 6.5	35.2%	Unknown	EWGSOP1	NINCDS-ADRDA
Fatma Sena Dost 2023	Turkey	Cross‐sectional	128	Hospital participants	76.6 ± 7.5	35.2%	26.7 ± 4.5	EWGSOP2	NIA/AA
Odete Vicente de Sousa 2022	Portugal	Cross‐sectional	79	Community participants	79.0 ± 5.8	40.6%	Unknown	EWGSOP2	DSM-5, NIA/AA
Mei Sian Chong 2015	Singapore	Cross‐sectional	299	Community participants	70.8 ± 8.5	32.8%	22.8 ± 3.5	AWGS	NINCDS-ADRDA
Veysel SUZAN 2022	Turkey	Cross‐sectional	339	Hospital participants	76.9 ± 7.1	28.3%	Unknown	EWGSOP2	DSM-5
Osamu Iritani 2021	Japan	Cross‐sectional	135	Hospital participants	79.5 ± 6.8	34.8%	Unknown	AWGS	DSM-5
Danielle Rodrigues Lecheta 2017	Brazil	Cross‐sectional	96	Hospital participants	78.0 ± 6.5	29.2%	Unknown	EWGSOP1	Ministry of Health criteria
Hsin Ning Lee 2020	Taiwan	Cross‐sectional	125	Hospital participants	79.5 ± 7.9	32.0%	24.1 ± 3.7	EWGSOP2	DSM-5
Shanwen Liu 2022	China	Cross‐sectional	82	Hospital participants	71.5 ± 7.6	31.7%	23.1 ± 3.0	AWGS	NIA/AA
Taiki Sugimoto 2017	Japan	Cross‐sectional	208	Hospital participants	78.0 ± 6.0	35.1%	22.1 ± 3.0	AWGS	NIA/AA
Michal S. Beeri 2021	USA	Cohort study	1175	Community participants	80.9 ± 7.1	22.8%	Unknown	EWGSOP2	NINCDS-ADRDA

AD, Alzheimer’s disease; ASMI, appendicular skeletal muscle mass index; AWGS, Asian Working Group for Sarcopenia; BMI, body mass index; DSM, Diagnostic and Statistical Manual of Mental Disorders; EWGSOP, European Working Group on Sarcopenia in Older People; NIA/AA, National Institute on Aging-Alzheimer’s Association; NINCDS-ADRDA, Alzheimer’s Disease and Related Disorders Association; SD, standard deviation.

### Diagnostic methods for sarcopenia

Details of the diagnostic criteria and methods for sarcopenia in each study are presented in Table 2. Studies were categorized separately due to the use of differing diagnostic criteria within the same study. Among these, 26 studies defined sarcopenia based on low muscle mass, low muscle strength, and low physical performance, whereas three studies relied on low muscle mass and low muscle strength. Muscle mass was assessed via various methods: 25 studies utilized bioelectrical impedance analysis (BIA), three employed dual‐energy X‐ray absorptiometry (DXA), and one study used ultrasound. Muscle strength was consistently measured with a handgrip dynamometer, whereas physical performance was evaluated through gait speed or the timed up-and-go test (TUG).

**Table 2:** Diagnostic criteria and methods for sarcopenia.

Assessment method		References
Muscle strength		
BIA	AWGS 2019 or 2016:	
	SMI < 7.0 kg/m2 for men and < 5.7 kg/m2 for women	Yusuke Ogawa 2018, Ai Kimura 2018, Daisuke Hirose 2016, Akito Tsugawa 2017, Osamu Iritani 2021, Taiki Sugimoto 2017, Shanwen Liu 2023, Xiaofen Weng 2023 (a), Taichi Demura 2023, Xiaofen Weng 2023 (b), Shanwen Liu 2022
	EWGSOP1:	
	SMI ≤ 8.87 kg/m2 for men and ≤ 6.42 kg/m2 for women	Giulia Bramato 2022 (a)^*^
	SMI < 7.0 kg/m2 for men and <5.7 kg/m2 for women	Taiki Sugimoto 2016
	SMI ≤ 10.75 kg/m2 for men and ≤6.75 kg/m2 for women	Danielle Rodrigues Lecheta 2017
	EWGSOP2:	
	ASM ≤ 20 kg for men and ≤15 kg for women	Giulia Bramato 2022 (b)^*^
	SMI < 7.0 kg/m2 for men and <5.7 kg/m2 for women	T Sugimoto 2022
	SMI < 9.2 kg/m2 for men and <7.4 kg/m2 for women	Cemile Özsürekci 2019, Zekeriya Ülger 2022 (a)^*^
	Fat‐free mass index (FFMI) of < 17 kg/m2 for men and <15 kg/m2 for women	Pelin Unsal 2023, Liss Elin Larsson 2023
	SMI < 8.33 kg/m2 for men and <5.70 kg/m2 for women	Fatma Sena Dost 2022, Fatma Sena Dost 2023
	Phase angle (PA) < 3.6° for men and <3.2° for women	Odete Vicente de Sousa 2022
	SMI < 8.9 kg/m2 for men and <6.4 kg/m2 for women	Hsin Ning Lee 2020
	SMI < 8.87 kg/m2 for men and <6.42 kg/m2 for women	Michal S. Beeri 2021
DXA	AWGS 2014:	
	SMI < 7.0 kg/m2 for men and <5.7 kg/m2 for women	Mei Sian Chong 2015
	EWGSOP1:	
	SMI < 7.0 kg/m2 for men and <5.7 kg/m2 for women	L Tay 2018
	EWGSOP2:	
	SMI < 7.0 kg/m2 for men and <5.5 kg/m2 for women	Veysel SUZAN 2022
Ultrasound	EWGSOP2:	
	Gastrocnemius muscle thickness <13 mm	Zekeriya Ülger 2022 (b)^*^
**Muscle strength**		
HGS	AWGS 2014:	
	HGS < 26 kg for men and <18 kg for women	Yusuke Ogawa 2018, Ai Kimura 2018, Daisuke Hirose 2016, Akito Tsugawa 2017, Mei Sian Chong 2015, Osamu Iritani 2021, Taiki Sugimoto 2017
	AWGS 2019:	
	HGS < 28 kg for men and <18 kg for women	Shanwen Liu 2023, Xiaofen Weng 2023 (a), Taichi Demura 2023, Xiaofen Weng 2023 (b), Shanwen Liu 2022
	EWGSOP1:	
	HGS ≤ 30 kg for men and ≤20 kg for women	Giulia Bramato 2022 (a)^*^
	HGS < 26 kg for men and <18 kg for women	Taiki Sugimoto 2016, L Tay 2018
	HGS reference values according to the BMI and gender	Danielle Rodrigues Lecheta 2017
	EWGSOP2:	
	HGS ≤ 27 kg for men and ≤16 kg for women	Giulia Bramato 2022 (b)^*^, Cemile Özsürekci 2019
	HGS < 28 kg for men and <18 kg for women	T Sugimoto 2022
	HGS < 27 kg for men and <16 kg for women	Pelin Unsal 2023, Liss Elin Larsson 2023, Veysel SUZAN 2022
	HGS < 32 kg for men and <19 kg for women	Zekeriya Ülger 2022 (a)^*^, Zekeriya Ülger 2022 (b)^*^
	HGS < 28 kg for men and <14 kg for women	Fatma Sena Dost 2022, Fatma Sena Dost 2023
	HGS ≤ 22.4 kg for men and ≤10.2 kg for women	Odete Vicente de Sousa 2022
	HGS < 22.4 kg for men and <14.3 kg for women	Hsin Ning Lee 2020
	HGS < 30 kg for men and <20 kg for women	Michal S. Beeri 2021
**Physical performance**		
Gait speed	AWGS 2014:	
	Low gait speed ( ≤0.8 m/s)	Yusuke Ogawa 2018, Akito Tsugawa 2017
	Low gait speed ( <0.8 m/s)	Ai Kimura 2018, Daisuke Hirose 2016, Mei Sian Chong 2015, Osamu Iritani 2021
	AWGS 2019:	
	Low gait speed (≤1.0 m/s)	Shanwen Liu 2023, Xiaofen Weng 2023 (a), Xiaofen Weng 2023 (b), Shanwen Liu 2022
	Low gait speed (<1.0 m/s)	Taichi Demura 2023
	EWGSOP1:	
	Low gait speed ( ≤0.8 m/s)	L Tay 2018
	EWGSOP2:	
	Low gait speed ( <0.8 m/s)	Cemile Özsürekci 2019, Fatma Sena Dost 2022, Fatma Sena Dost 2023
	Low gait speed ( ≤0.8 m/s)	Pelin Unsal 2023, Liss Elin Larsson 2023, Zekeriya Ülger 2022 (a)^*^, Zekeriya Ülger 2022 (b)^*^, Odete Vicente de Sousa 2022, Veysel SUZAN 2022
	Low gait speed (<1.0 m/s)	Hsin Ning Lee 2020
TUG	AWGS 2014:	
	TUG ≥ 14 seconds	Taiki Sugimoto 2017
	EWGSOP1:	
	TUG ≥ 13.56 seconds	Taiki Sugimoto 2016
	TUG > 10 seconds	Danielle Rodrigues Lecheta 2017
	EWGSOP2:	
	TUG ≥ 20 seconds	T Sugimoto 2022

ASM, appendicular skeletal muscle mass; AWGS, Asian Working Group for Sarcopenia; BIA, bioelectrical impedance analysis; DXA, dual‐energy X‐ray absorptiometry; EWGSOP, European Working Group on Sarcopenia in Older People; SMI, skeletal muscle mass index; HGS, handgrip strength; TUG, timed up and go test.

### Meta‐analysis results

#### Prevalence of sarcopenia in all patients with AD.

**Fig 2:**
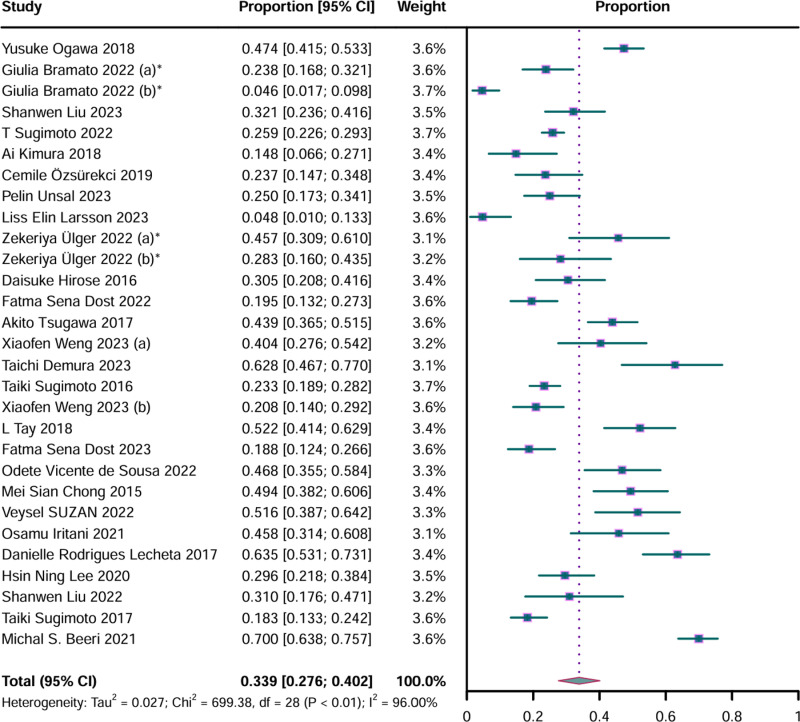
Forest plot of prevalence of sarcopenia in Alzheimer’s disease. CI, confidence interval. Results from the random-effects model. *  represents results from the same study.

From a total of 3902 AD patient samples, 1284 were identified as having sarcopenia. The pooled prevalence of sarcopenia among AD patients was 33.9% (95% CI: 0.276–0.402). The analysis revealed a significant degree of heterogeneity (P < 0.01, I^2^ = 96.00%) (Fig 2).

#### Meta-regression analyses.

To investigate potential variations in sources, we performed meta-regression analyses. Factors such as study region, study type, diagnostic criteria for AD, diagnostic criteria for sarcopenia, participant origin, gender, and age were considered. In the univariate meta-regression analysis, the occurrence of sarcopenia was notably linked to the origin of the participants (β = −1.0913, SE = 0.4169, P = 0.0143) and the mean age (β = 0.1030, SE = 0.0316, P = 0.0030) (Table 3); this was followed by multivariate meta-regression analysis. The results of the collinearity analysis between variables are shown in S5 Table. The variables included the source of the participants, type of study design and mean age of the participants, and the results are shown in Table 4. Source of participants (β = −0.9187, SE = 0.4006, P = 0.0305) and mean age (β = 0.0926, SE = 0.0295, P = 0.0043) remained significantly associated with prevalence.

**Table 3:** Univariate meta-regression analysis of the prevalence of sarcopenia in AD.

Variables	β	SE	P
AD: DSM-5 vs. other	−0.0683	0.3343	0.8396
Sarcopenia: EWGSOP2 vs. other	−0.3917	0.3251	0.2387
Muscle mass measurements: BIA vs. other	−0.6366	0.4638	0.1811
Study participants: Hospital participants vs. other	−1.0913	0.4169	0.0143
Gender: Mix vs. Female	0.0105	0.8937	0.9907
Study region: Asia vs. other	0.0815	0.4165	0.8464
Study design: Cohort study vs. Cross‐sectional study	1.6379	0.8093	0.0530
Mean age	0.1030	0.0316	0.0030

AD, Alzheimer’s disease; BIA, bioelectrical impedance analysis; DSM, Diagnostic and Statistical Manual of Mental Disorders; EWGSOP, European Working Group on Sarcopenia in Older People.

**Table 4:** Multivariate meta-regression analysis of the prevalence of sarcopenia in AD.

Variables	β	SE	P
Study participants: Hospital participants vs. other	−0.9187	0.4006	0.0305
Study design: Cohort study vs. Cross‐sectional study	0.3649	0.7478	0.6298
Mean age	0.0926	0.0295	0.0043

#### Subgroup analyses.

Subgroup analysis indicated that the prevalence of sarcopenia varied by region and demographic factors. In Asian AD patients, the prevalence was 0.333 (95% CI: 0.279, 0.387), whereas it was to 0.195 (95% CI: 0.003–0.388) in European patients and 0.680 (95% CI: 0.621–0.738) in American patients. The prevalence among hospital participants was 0.304 (95% CI: 0.243–0.365). Cross-sectional studies reported a prevalence of 0.324 (95% CI: 0.265–0.383). The diagnostic criteria also influenced prevalence: 0.335 (95% CI: 0.239–0.431) for DSM-5, 0.234 (95% CI: 0.195–0.274) for NIA/AA, 0.355 (95% CI: 0.246–0.463) for AWGS 2014, and 0.300 (95% CI: 0.197–0.403) for EWGSOP2. The prevalence was 0.321 (95% CI: 0.253–0.389) for those diagnosed with sarcopenia by BIA and 0.511 (95% CI: 0.447–0.574) for those diagnosed with sarcopenia by DXA. The prevalence of sarcopenia was greater in patients with a body mass index (BMI) < 24 kg/m^2^ (0.362, 95% CI: 0.274–0.450) than in those with a BMI ≥  24 kg/m^2^ (0.216, 95% CI: 0.125–0.307). The older the patients were, the greater the prevalence was. The highest prevalence was found in patients over 80 years old (0.557, 95% CI: 0.431–0.683) (Table 5) (subgroup analysis graphs can be viewed in S1 Fig).

**Table 5:** The results of subgroup analysis in prevalence of sarcopenia in AD.

Variables	Numbers of studies	Meta‐analysis results	I^2^	P
Study region				
Asia	23	0.333 (0.279, 0.387)	88.67%	< 0.01
Europe	4	0.195 (0.003, 0.388)	95.66%	< 0.01
America	2	0.680 (0.621, 0.738)	20.4%	0.26
Study participants				
Hospital participants	25	0.304 (0.243, 0.365)	94.18%	< 0.01
Community participants	4	0.553 (0.443, 0.664)	86.86%	< 0.01
Study design				
Cross−sectional study	28	0.324 (0.265, 0.383)	94.34%	< 0.01
Cohort study	1	0.700 (0.638, 0.757)	/	/
AD diagnostic criteria				
DSM − 5	13	0.335 (0.239, 0.431)	96.26%	< 0.01
NIA/AA	10	0.234 (0.195, 0.274)	63.12%	< 0.01
NINCDS−ADRDA	4	0.486 (0.292, 0.680)	98.16%	< 0.01
DSM − 5, NIA/AA	1	0.468 (0.355, 0.584)	/	/
Ministry of Health criteria	1	0.635 (0.531, 0.731)	/	/
Sarcopenia diagnostic criteria				
AWGS 2014	7	0.355 (0.246, 0.463)	93.24%	< 0.01
AWGS 2019	5	0.367 (0.232, 0.502)	85.8%	< 0.01
EWGSOP1	4	0.404 (0.205, 0.603)	96%	< 0.01
EWGSOP2	13	0.300 (0.197, 0.403)	97.38%	< 0.01
Assessment method of muscle mass				
BIA	25	0.321 (0.253, 0.389)	96.27%	< 0.01
Ultrasound	1	0.283 (0.160, 0.435)	/	/
DXA	3	0.511 (0.447, 0.574)	0%	0.93
BMI				
<24	10	0.362 (0.274, 0.450)	92.24%	< 0.01
≥24	8	0.216 (0.125, 0.307)	92.35%	< 0.01
Unknown	11	0.404 (0.293, 0.515)	96.32%	< 0.01
Age				
>80	4	0.557 (0.431, 0.683)	92.95%	< 0.01
>75, <80	14	0.330 (0.251, 0.410)	90.72%	< 0.01
>70, <75	10	0.287 (0.201, 0.373)	93.12%	< 0.01
<70	1	0.048 (0.010, 0.133)	/	/
Gender				
Mix	28	0.340 (0.274, 0.405)	96.13%	< 0.01
Female	1	0.321 (0.236, 0.416)	/	/

AD, Alzheimer’s disease; ASMI, appendicular skeletal muscle mass index; AWGS, Asian Working Group for Sarcopenia; BIA, bioelectrical impedance analysis; BMI, body mass index; DSM, Diagnostic and Statistical Manual of Mental Disorders; DXA, dual‐energy X‐ray absorptiometry; EWGSOP, European Working Group on Sarcopenia in Older People; NIA/AA, National Institute on Aging-Alzheimer’s Association; NINCDS-ADRDA, Alzheimer’s Disease and Related Disorders Association.

#### Sensitivity analysis.

To assess the robustness of our findings, we performed sensitivity analyses by sequentially excluding each study from the meta-analysis. The prevalence estimates remained stable, indicating the reliability of the results (S2 Fig).

#### Prevalence of sarcopenia in mild AD patients.

**Fig 3:**
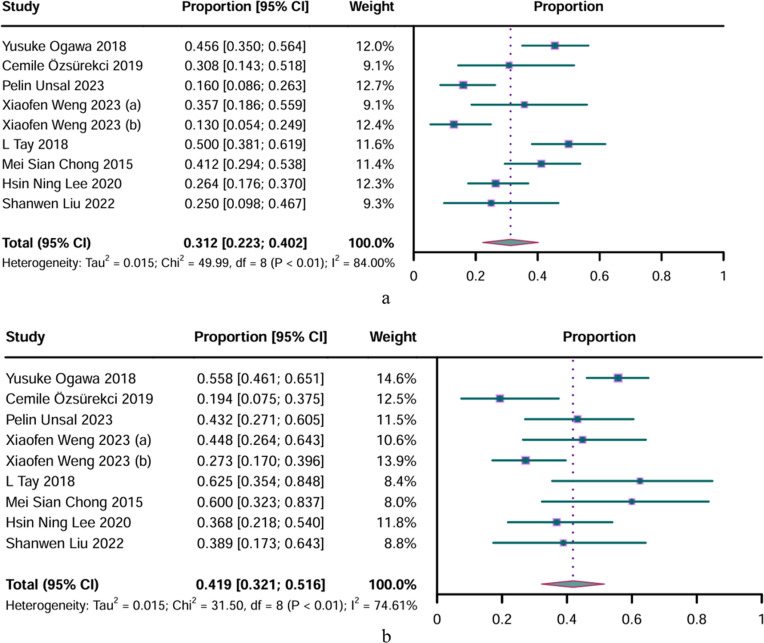
Forest plot of sarcopenia prevalence in different stages of Alzheimer’s disease. a. Forest plot of prevalence of sarcopenia in mild Alzheimer’s disease; b. Forest plot of prevalence of sarcopenia in moderate Alzheimer’s disease. CI, confidence interval. Results from the random-effects model.

A meta-analysis of nine studies reporting sarcopenia prevalence in mild AD patients revealed a prevalence of 31.2% (95% CI: 0.223–0.402). Considerable heterogeneity was observed across the studies (P < 0.01, I^2^ = 84.00%) (Fig 3a). Different studies have applied various diagnostic criteria for mild AD staging, including 21 ≤ Mini-Mental State Examination (MMSE) ≤ 23, 21 ≤ MMSE ≤ 26, and Clinical Dementia Rating (CDR) = 1. Subgroup analysis revealed that the prevalence of sarcopenia among participants with 21 ≤ MMSE ≤ 26 was 0.228 (95% CI: 0.091–0.364), whereas it was 0.326 (95% CI: 0.204–0.447) for participants with a CDR = 1. Subgroup analysis based on different sarcopenia diagnostic criteria revealed a prevalence of 0.436 (95% CI: 0.359–0.514) using AWGS 2014, 0.288 (95% CI: 0.091–0.364) using AWGS 2019, and 0.227 (95% CI: 0.140–0.315) using EWGSOP2 (Table 6) (subgroup analysis graphs can be viewed in S3 Fig). Sensitivity analyses confirmed the stability of these results (S2 Fig).

**Table 6:** The results of subgroup analysis in prevalence of sarcopenia in mild AD.

Variables	Numbers of studies	Meta‐analysis results	I^2^	P
AD staging criteria				
21 ≤ MMSE ≤ 23	1	0.456 (0.350–0.564)	/	/
21 ≤ MMSE ≤ 26	3	0.228 (0.091–0.364)	64.36%	0.06
CDR = 1	5	0.326 (0.204–0.447)	85.11%	<0.01
Sarcopenia diagnostic criteria				
AWGS 2014	2	0.436 (0.359–0.514)	0%	0.58
AWGS 2019	3	0.288 (0.091–0.364)	64.36%	0.06
EWGSOP1	1	0.500 (0.381–0.619)	/	/
EWGSOP2	3	0.227 (0.140–0.315)	47.65%	0.15

AD, Alzheimer’s disease; AWGS, Asian Working Group for Sarcopenia; CDR, Clinical Dementia Rating; EWGSOP, European Working Group on Sarcopenia in Older People; MMSE, Mini-mental State Examination.

#### Prevalence of sarcopenia in moderate and severe AD patients.

A meta-analysis of nine studies involving patients with moderate AD revealed that 41.9% (95% CI: 0.321–0.516) had sarcopenia, indicating significant heterogeneity (P < 0.01, I^2^ = 74.61%) (Fig 3b). Subgroup analysis revealed that the prevalence of sarcopenia was highest among participants with MMSE ≤ 20, at 0.588 (95% CI: 0.463–0.652), followed by those with CDR = 2, with a prevalence of 0.423 (95% CI: 0.269–0.578). Participants with 10 ≤ MMSE ≤ 20 had a lower prevalence of 0.294 (95% CI: 0.197–0.391). Subgroup analysis based on sarcopenia diagnostic criteria revealed a prevalence of 0.563 (95% CI: 0.477–0.649) using AWGS 2014, 0.348 (95% CI: 0.288–0.468) using AWGS 2019, and 0.327 (95% CI: 0.185–0.470) using EWGSOP2. This highlighted the variability in sarcopenia prevalence depending on the diagnostic criteria and staging methods used (Table 7) (subgroup analysis graphs can be viewed in S3 Fig). Sensitivity analyses validated the robustness of these findings (S2 Fig). Only one study reported the prevalence of sarcopenia in patients with severe AD, which was 21.1% [41].

**Table 7:** The results of subgroup analysis in prevalence of sarcopenia in moderate AD.

Variables	Numbers of studies	Meta‐analysis results	I^2^	P
AD staging criteria				
MMSE ≤ 20	1	0.588 (0.461–0.651)	/	/
15 ≤ MMSE ≤ 20	1	0.448 (0.264–0.643)	/	/
10 ≤ MMSE ≤ 20	2	0.294 (0.197–0.391)	0%	0.36
CDR = 2	5	0.423 (0.269–0.578)	72.34%	<0.01
Sarcopenia diagnostic criteria				
AWGS 2014	2	0.563 (0.477–0.649)	0%	0.75
AWGS 2019	3	0.348 (0.288–0.468)	33.19%	0.22
EWGSOP1	1	0.625 (0.354–0.848)	/	/
EWGSOP2	3	0.327 (0.185–0.470)	63.4%	0.07

AD, Alzheimer’s disease; AWGS, Asian Working Group for Sarcopenia; CDR, Clinical Dementia Rating; EWGSOP, European Working Group on Sarcopenia in Older People; MMSE, Mini-mental State Examination.

#### Odds ratio results.

**Fig 4:**
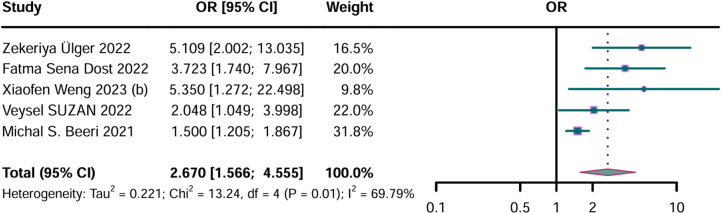
Forest plot of the adjusted odds ratios (ORs) between Alzheimer’s disease and sarcopenia. CI, confidence interval. Results from the random-effects model. *  represents results from the same study.

An analysis was conducted on five studies with a total of 2573 participants to determine the general OR for the link between AD and sarcopenia. A summary of the adjusted ORs and adjustment factors of the studies is provided in S6 Table. The adjusted OR was 2.670 (95% CI: 1.566–4.555), indicating a significant positive correlation, with considerable heterogeneity (P = 0.01, I² = 69.79%) (Fig 4).

#### Meta-regression analyses.

Univariate meta-regression analysis identified mean age (β = −0.1339, SE = 0.0199, P = 0.0067) as a significant source of heterogeneity (Table 8). The results of the collinearity analysis between variables are shown in S7 Table. However, the significant association between age and OR disappeared in the multivariate meta-regression (Table 9).

**Table 8:** Univariate meta-regression analysis of the correlation between sarcopenia and AD.

Variables	β	SE	P
AD: DSM-5 vs. other	0.2678	0.5888	0.6801
Sarcopenia: EWGSOP2 vs. AWGS 2019	−0.8270	0.9915	0.4654
Muscle mass measurements: BIA vs. other	−0.2678	0.5888	0.6801
Study participants: Hospital participants vs. other	0.7660	0.2554	0.0577
Study region: Asia vs. America	0.7660	0.2554	0.0577
Study design: Cohort study vs. Cross‐sectional study	−0.7660	0.2554	0.0577
Mean age	−0.1339	0.0199	0.0067

AD, Alzheimer’s disease; BIA, bioelectrical impedance analysis; EWGSOP, European Working Group on Sarcopenia in Older People; NIA/AA, National Institute on Aging-Alzheimer’s Association.

**Table 9:** Multivariate meta-regression analysis of the correlation between sarcopenia and AD.

Variables	β	SE	P
Study population: Hospital participants vs. other	−0.4874	0.3596	0.3801
Mean age	−0.2063	0.0562	0.0669

#### Subgroup analyses.

Among individuals diagnosed with AD, the adjusted OR for sarcopenia was 3.043 (95% CI: 1.252–7.393) using DSM-5 criteria and 4.031 (95% CI: 2.058–7.895) with NIA/AA criteria. For sarcopenia patients diagnosed by EWGSOP2 criteria, the OR was 2.470 (95% CI: 1.417–4.306), whereas those diagnosed with the AWGS 2019 criteria had a higher OR of 5.350 (95% CI: 1.272–22.498). The muscle mass assessment methods used varied, with ultrasound showing the highest OR of 5.109 (95% CI: 2.002–13.035). The OR decreased with increasing age, from 4.369 (95% CI: 2.530–7.544) for those 70 ~ 75 years to 1.500 (95% CI: 1.205–1.867) for those >  80 years. BMI < 24 had an OR of 5.350, whereas an unknown BMI showed moderate heterogeneity (I² = 63.78%, P = 0.06). Asian populations, hospital participants, and cross-sectional studies showed a consistent OR of 3.333 (95% CI: 2.041–5.441) with low heterogeneity (I² = 9.38%, P = 0.35), whereas a cohort study from America reported a lower OR of 1.500 (95% CI: 1.205–1.867), contributing to heterogeneity [14] (Table 10) (subgroup analysis graphs can be viewed in S4 Fig).

**Table 10:** The results of subgroup analysis for OR between sarcopenia and AD.

Variables	Numbers of studies	Meta‐analysis results	I^2^	P
AD assessment				
DSM−5	2	3.043 (1.252, 7.393)	58.73%	0.12
NIA/AA	2	4.031 (2.058, 7.895)	0%	0.66
NINCDS−ADRDA	1	1.500 (1.205, 1.867)	/	/
Sarcopenia assessment				
EWGSOP2	4	2.470 (1.417, 4.306)	72.49%	0.01
AWGS 2019	1	5.350 (1.272, 22.498)	/	/
Assessment method of muscle mass				
BIA	3	2.585 (1.177–5.676)	74.02%	0.02
DXA	1	2.048 (1.049–3.998)	/	/
Ultrasound	1	5.109 (2.002–13.035)	/	/
Age				
>70, <75	3	4.369 (2.530, 7.544)	0%	0.84
>75, <80	1	2.048 (1.049, 3.998)	/	/
>80	1	1.500 (1.205, 1.867)	/	/
BMI				
<24	1	5.350 (1.272, 22.498)	/	/
≥24	1	5.109 (2.002, 13.035)	/	/
Unknown	3	2.040 (1.223, 3.401)	63.78%	0.06
Study region				
Asia	4	3.333 (2.041, 5.441)	9.38%	0.35
America	1	1.500 (1.205, 1.867)	/	/
Study participants				
Hospital participants	4	3.333 (2.041, 5.441)	9.38%	0.35
Community participants	1	1.500 (1.205, 1.867)	/	/
Study design				
Cross−sectional study	4	3.333 (2.041, 5.441)	9.38%	0.35
Cohort study	1	1.500 (1.205, 1.867)	/	/

AD, Alzheimer’s disease; AWGS, Asian Working Group for Sarcopenia; BIA, bioelectrical impedance analysis; BMI, body mass index; DSM, Diagnostic and Statistical Manual of Mental Disorders; DXA, dual‐energy X‐ray absorptiometry; EWGSOP, European Working Group on Sarcopenia in Older People; NIA/AA, National Institute on Aging-Alzheimer’s Association; NINCDS-ADRDA, Alzheimer’s Disease and Related Disorders Association; OR, odds ratio.

#### Sensitivity analysis.

To ensure the quality and reliability of our results, a sensitivity analysis was performed by excluding one study at a time. The results confirmed the stability of the meta-analysis (S2 Fig ).

#### Publication bias.

Egger’s test and Begg’s test clearly revealed publication bias between studies (P < 0.05, S5 Fig). Publication bias was not assessed for in OR studies due to an insufficient amount of studies.

## Discussion

This research is the first meta-analysis to examine the frequency and connection of sarcopenia in different stages of AD. Our findings indicate that the overall prevalence of sarcopenia among AD patients is 33.9%, which is significantly higher than the prevalence reported in the general elderly population [44]. This elevated prevalence underscores a substantial association between AD and sarcopenia, highlighting sarcopenia as a notable comorbidity in individuals with AD that can adversely impact their quality of life and overall health outcomes. The prevalence of sarcopenia varied according to the AD stage: 31.2% in patients with mild AD and 41.9% in those with moderate AD. This result suggested that the incidence of sarcopenia tended to change as the course of AD progressed, especially in patients with moderate AD, where the incidence of sarcopenia was significantly greater. The results suggested that with the decline in cognitive function, a patient’s physical function may also gradually decline, thus increasing the risk of sarcopenia.

The strong association between AD and sarcopenia, reflected in an OR of 2.670, suggested that sarcopenia was not simply a byproduct of aging but was potentially linked to the underlying pathophysiology of AD. This connection was observed consistently across various subgroups, reinforcing the need to consider sarcopenia as a significant component of the clinical profile in AD patients. The cognitive decline inherent in AD often results in reduced physical activity, malnutrition, and difficulties in maintaining daily routines, all of which contribute to muscle loss [45–47]. Furthermore, AD and sarcopenia may share common pathological mechanisms, such as chronic inflammation, mitochondrial dysfunction, and metabolic abnormalities [48–51]. The intricate relationship between AD and sarcopenia necessitates a comprehensive approach to patient care. This includes routine screening for sarcopenia in AD patients and the implementation of interventions aimed at maintaining muscle mass and function. The treatment of sarcopenia primarily includes nutritional intervention, exercise therapy, and pharmacological treatment. Nutritional intervention emphasizes increasing protein intake, especially high-quality proteins rich in essential amino acids such as leucine, to promote muscle synthesis. Additionally, supplementation with vitamin D and calcium, among other nutrients, is crucial for maintaining muscle health. Exercise therapy recommends engaging in aerobic exercises and resistance training to improve muscle strength and endurance. Furthermore, in terms of pharmacological treatment, although there are currently no specific drugs for sarcopenia, certain medications, such as androgen, growth hormone and its analogs, and selective androgen receptor modulators may help improve muscle mass and function in specific situations [51]. These therapeutic measures provide potential strategies for the prevention of sarcopenia in AD patients.

To further explore the factors influencing the prevalence and OR of sarcopenia, subgroup analyses were performed. Analysis of the subgroups revealed that variables such as age and BMI were significant factors in the occurrence of sarcopenia in individuals with AD. In particular, elderly people, particularly those above 80 years of age, had a greater occurrence of sarcopenia at a rate of 53.6%, which supported the idea that increasing age is a major contributor to muscle decline. Furthermore, individuals with AD who had a BMI below 24 kg/m² had a greater occurrence of sarcopenia (36.6%) than did those with a higher BMI. These findings indicate that inadequate nutrition and low body weight play crucial roles in the development of muscle loss in this group.

Because there was considerable heterogeneity in the diagnostic methods of sarcopenia and AD among the included studies, we tried to consider the possible influence of different diagnostic criteria and methods during the analysis. Subgroup analysis revealed that the prevalence of sarcopenia was the highest (40.4%) when the EWGSOP1 was used to define sarcopenia. The OR of using AWGS 2019 (5.350) to define sarcopenia was greater than that of EWGSOP2 (2.470). The diagnostic cutoff value of sarcopenia had a great impact on the assessment of prevalence and the OR. We found that there is no consensus on the diagnostic cutoff values for sarcopenia, which may vary depending on ethnicity, cultural background, dietary habits, and quality of life. Among them, the AWGS provides a suitable diagnostic cutoff value for Asian patients. Therefore, there are differences in the prevalence and OR of sarcopenia among AD patients in different countries and regions. This was also confirmed in our meta-regression analysis and subgroup analysis. In addition, most of the included studies were from Asia, and factors such as diet patterns, physical activity levels, and health care systems may contribute to regional differences in the prevalence and correlation of sarcopenia among older adults in Asia [52]. Therefore, the results of this study may be more representative of AD patients in Asia, while the generalizability to other regions may be limited. Future studies should include more research data from different geographic regions and cultural backgrounds and establish diagnostic cutoff values for sarcopenia in AD patients from different regions and races to verify whether the relationship between sarcopenia and AD is universal across the globe.

There are many methods used to evaluate muscle mass, with most studies using DXA and BIA to measure muscle mass [1]. Three methods were included in this study, among which DXA had the highest diagnostic rate (51.1%). DXA can rapidly and noninvasively assess fat mass and bone mineral density, but it requires specialized radiological equipment and is expensive [53]. BIA is inexpensive and easy to perform, but it may underestimate fat mass and overestimate muscle mass [54]. Ultrasound is less commonly used. Therefore, the heterogeneity introduced by the detection methods should be minimized in future studies.

Notably, the diagnostic criteria for AD significantly influenced the prevalence of sarcopenia and its associated OR. Studies using stricter criteria, such as NIA/AA, reported lower sarcopenia prevalence (23.4%) and higher OR (4.031), with minimal heterogeneity, reflecting the consistency and reliability of results driven by stringent biomarker-based definitions. In contrast, studies employing broader criteria, such as DSM-5, showed higher prevalence (33.5%) and lower OR (3.043), accompanied by moderate heterogeneity, indicating greater variability among study populations. Older criteria, such as NINCDS-ADRDA, reported the highest prevalence (48.6%) but with lower consistency. These findings highlight the significant impact of diagnostic criteria on the magnitude and variability of outcomes [55]. Therefore, NIA/AA is recommended as the diagnostic standard for AD in future studies.

Our univariate and multivariate meta-regression analyses revealed that the heterogeneity of the study results may have partly stemmed from the source of the study participants. We found that, compared with community-based patients, the prevalence of sarcopenia was lower in hospital-based patients, but the OR for the association between AD and sarcopenia was greater in the hospital group. This can be explained by several factors. Hospital-based patients, including outpatient participants, are generally subject to stricter inclusion criteria, whereas community-based patients typically represent a broader, more diverse population, leading to a higher prevalence of sarcopenia in the community group. Additionally, the diagnostic criteria and assessment tools used to identify sarcopenia may differ between the two groups. Hospital patients often undergo more detailed and rigorous evaluations, including muscle strength tests, physical performance tests, and precise imaging techniques, which allow for a more accurate diagnosis of sarcopenia [56]. Furthermore, hospital-based participants generally have more severe conditions and may suffer from a greater burden of comorbidities, which likely contributes to the stronger association between AD and sarcopenia in this population [44]. Importantly, the AD population in hospital-based studies is generally larger than that in community-based studies, which may limit the representativeness of community-based data. Therefore, further high-quality studies are needed to better understand the relationship between sarcopenia and AD in both hospital and community settings.

Despite these robust findings, this study has several limitations. The studies included showed considerable variability in terms of diagnostic criteria and methods. The study population was predominantly Asian, and the study population was mostly hospital outpatients, which limits global adaptability. The cross-sectional design of most of the studies restricted the ability to establish a causal relationship between AD and sarcopenia. Additionally, the high degree of heterogeneity among the studies suggested that a variety of confounding factors, such as nutritional status, physical activity level, and comorbidities, may influence the prevalence of sarcopenia in AD patients. In the included studies, OR levels were adjusted for demographic statistics, including age, gender, BMI, and education. However, some confounding factors, such as nutritional intake, physical activity or other comorbidities, may affect the degree of association between AD and sarcopenia. For example, the higher the comorbidity index is, the greater the risk of sarcopenia [44]. However, due to differences in the design and methods of different studies, the degree of control of confounding factors varies. All confounding factors could not be unified in this study. Therefore, more longitudinal and multicenter studies that control for these potential confounding factors as much as possible will be helpful revealing the causal relationship between AD and sarcopenia. With a longitudinal study design, it is possible to systematically assess the role of these factors and provide deeper causal inference.

## Conclusions

In conclusion, our meta-analysis highlights a significant prevalence of sarcopenia in AD patients, emphasizing the need for integrated care approaches that address both cognitive and physical health issues. Further research is needed to elucidate the pathophysiological connections between AD and sarcopenia and to develop effective interventions for this vulnerable population.

## Supporting information

S1 Fig Subgroup analysis in prevalence of sarcopenia in AD  . a. Study region, b. Study participants, c. Study design, d. AD diagnostic criteria, e. Sarcopenia diagnostic criteria, f. Assessment methods of muscle mass, g. BMI, h. Age, i. Gender.
(PNG)

S2 Fig Sensitivity analysis for included studies. a. The sensitivity analysis for prevalence of sarcopenia in AD, b. The sensitivity analysis for the adjusted OR between AD and sarcopenia, c. The sensitivity analysis for prevalence of sarcopenia in mild AD, d. The sensitivity analysis for prevalence of sarcopenia in moderate AD.
(PNG)

S3 Fig Subgroup analysis in prevalence of sarcopenia in different AD stages. a. Subgroup analysis of AD staging criteria for mild AD, b. Subgroup analysis of sarcopenia criteria for mild AD, c. Subgroup analysis of AD staging criteria for moderate AD, d. Subgroup analysis of sarcopenia criteria for moderate AD.
(PNG)

S4 Fig Subgroup analysis for the adjusted OR between AD and sarcopenia. a. AD diagnostic criteria, b. Sarcopenia diagnostic criteria, c. Assessment methods of muscle mass, d. Age, e. BMI, f. Study region/ population/design.
(PNG)

S5 Fig Publication bias assessment of sarcopenia prevalence. (PNG)

S1 Table AHRQ cross-sectional study evaluation criteria. (DOCX)

S2 Table The NOS score for cohort studies. (DOCX)

S3 Table Studies identified after excluding duplications. (DOC)

S4 Table Data extracted from included studies and used for all analyses. (XLSX)

S5 Table Collinearity between variables in sarcopenia prevalence. (DOCX)

S6 Table The adjusted ORs and adjustment factors of the studies. (DOCX)

S7 Table Collinearity between variables in OR. (DOCX)

S1 File PRISMA 2020 checklist for systematic review. (DOCX)
