# Supplementary figures and images for: Prevalence and correlation of sarcopenia with Alzheimer’s disease: A systematic review and meta-analysis
Source: PLoS One. 2025 Mar 3;20(3):e0318920. doi: 10.1371/journal.pone.0318920 (PMC11875368; doi:10.1371/journal.pone.0318920)

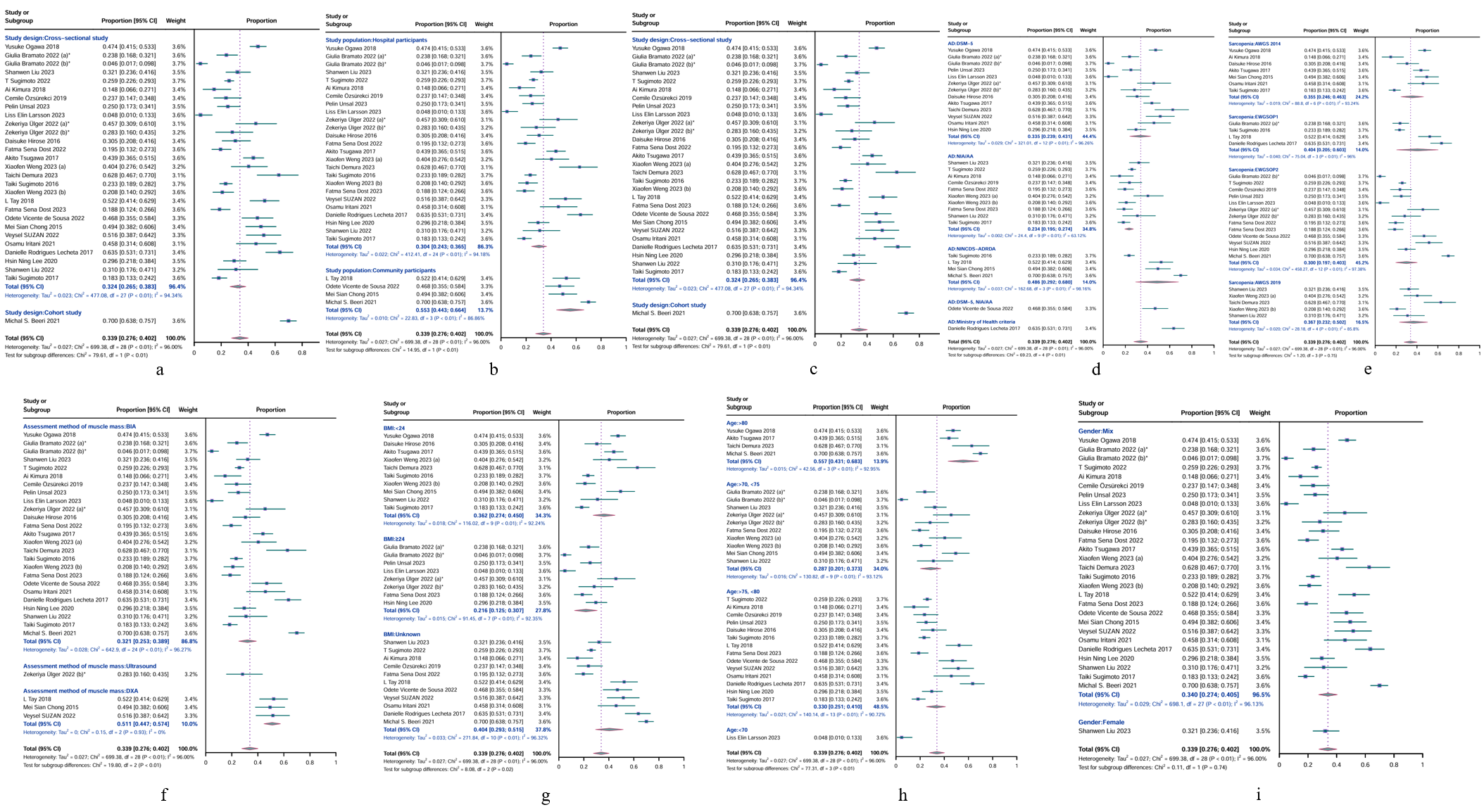

Supplement: S1 Fig — . a. Study region, b. Study participants, c. Study design, d. AD diagnostic criteria, e. Sarcopenia diagnostic criteria, f. Assessment methods of muscle mass, g. BMI, h. Age, i. Gender. (PNG) [file pone.0318920.s001.png]

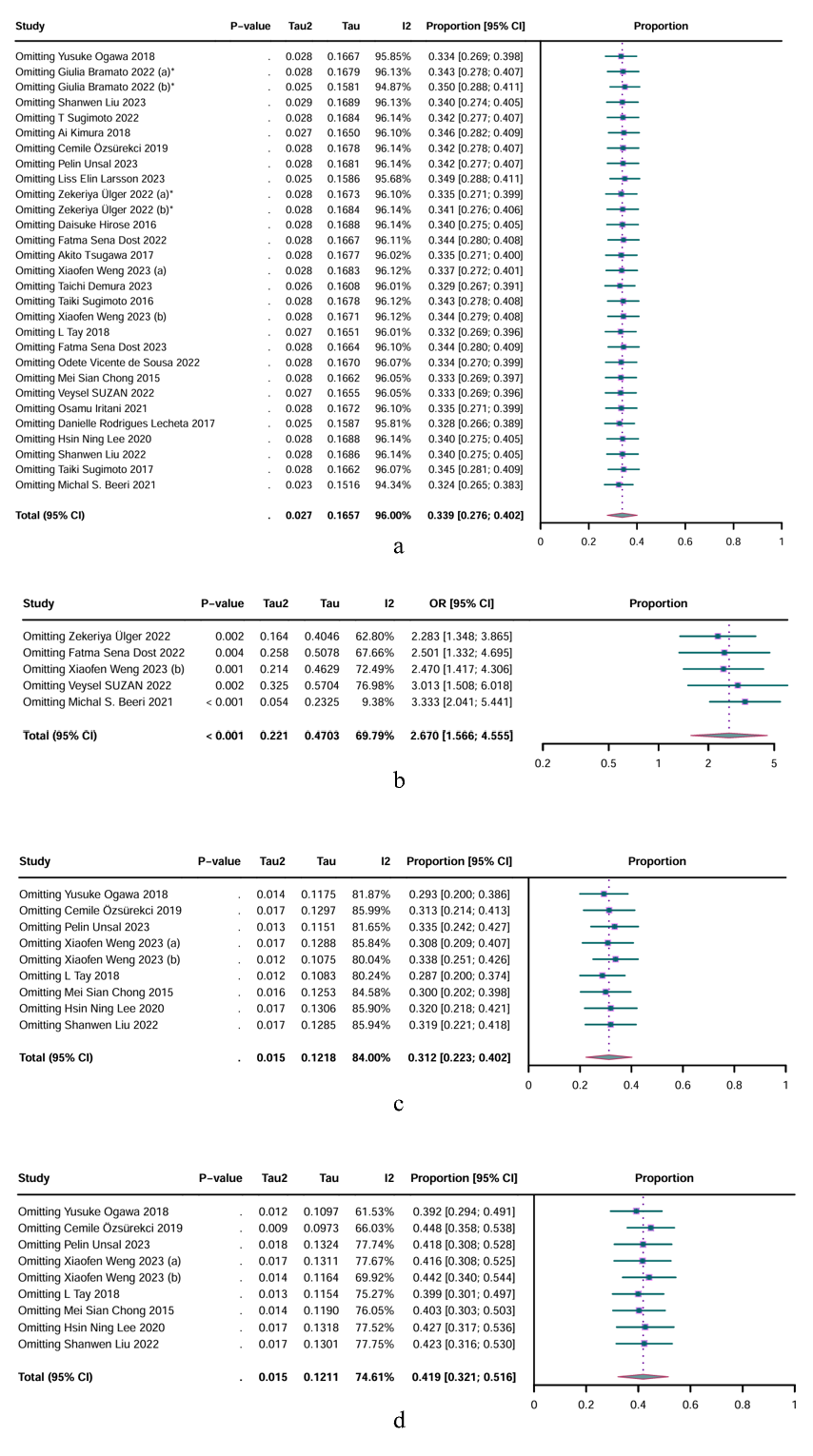

Supplement: S2 Fig — a. The sensitivity analysis for prevalence of sarcopenia in AD, b. The sensitivity analysis for the adjusted OR between AD and sarcopenia, c. The sensitivity analysis for prevalence of sarcopenia in mild AD, d. The sensitivity analysis for prevalence of sarcopenia in moderate AD. (PNG) [file pone.0318920.s002.png]

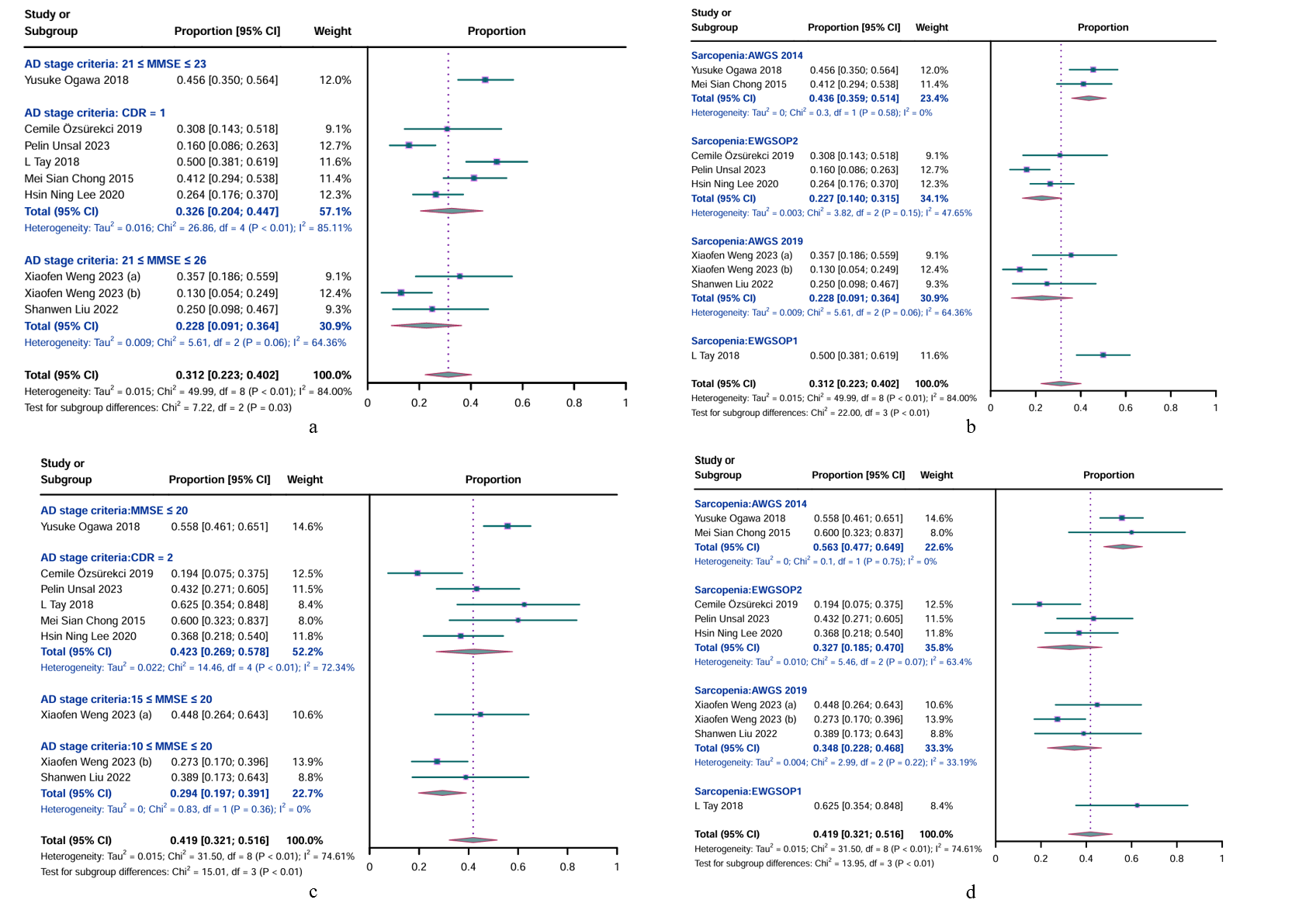

Supplement: S3 Fig — a. Subgroup analysis of AD staging criteria for mild AD, b. Subgroup analysis of sarcopenia criteria for mild AD, c. Subgroup analysis of AD staging criteria for moderate AD, d. Subgroup analysis of sarcopenia criteria for moderate AD. (PNG) [file pone.0318920.s003.png]

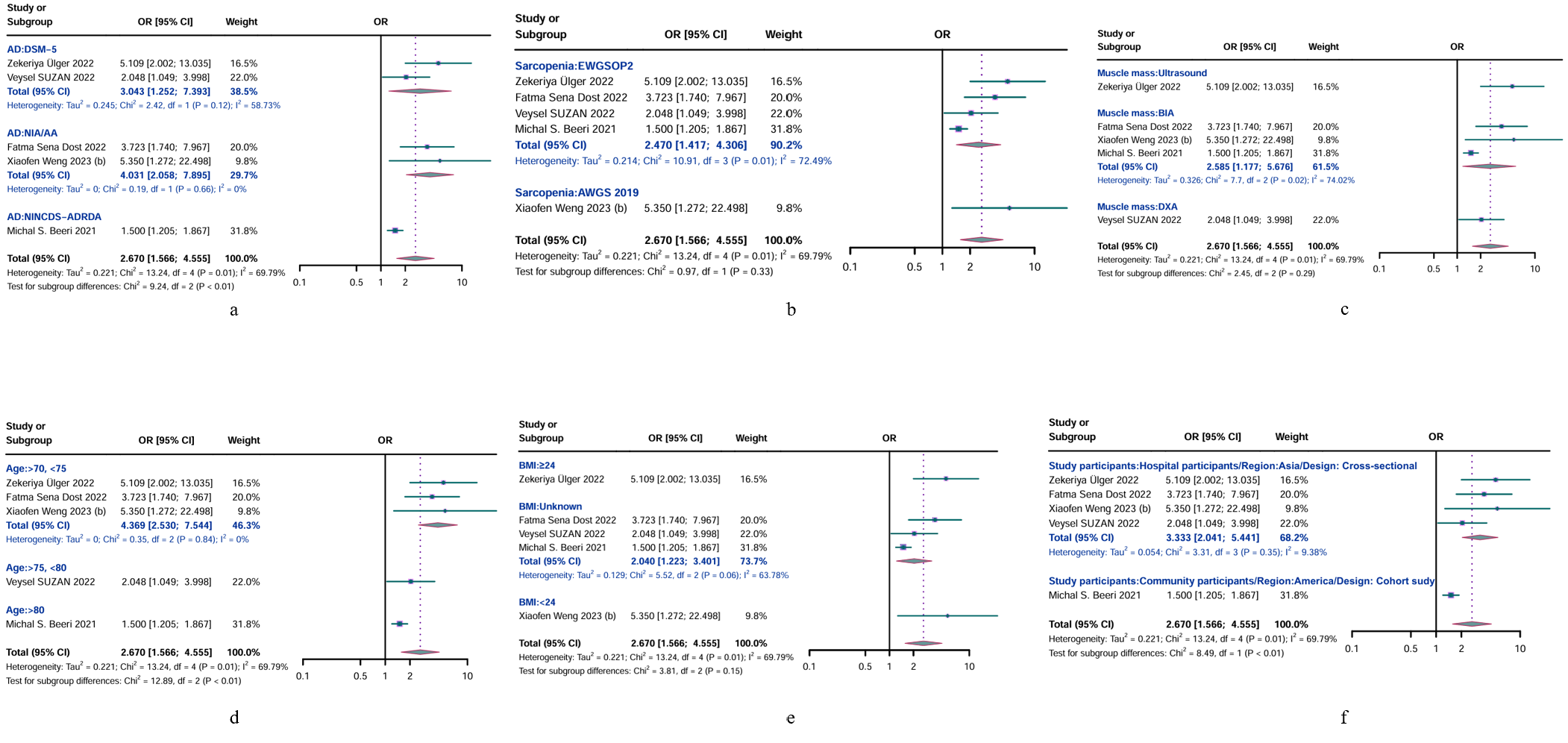

Supplement: S4 Fig — a. AD diagnostic criteria, b. Sarcopenia diagnostic criteria, c. Assessment methods of muscle mass, d. Age, e. BMI, f. Study region/ population/design. (PNG) [file pone.0318920.s004.png]

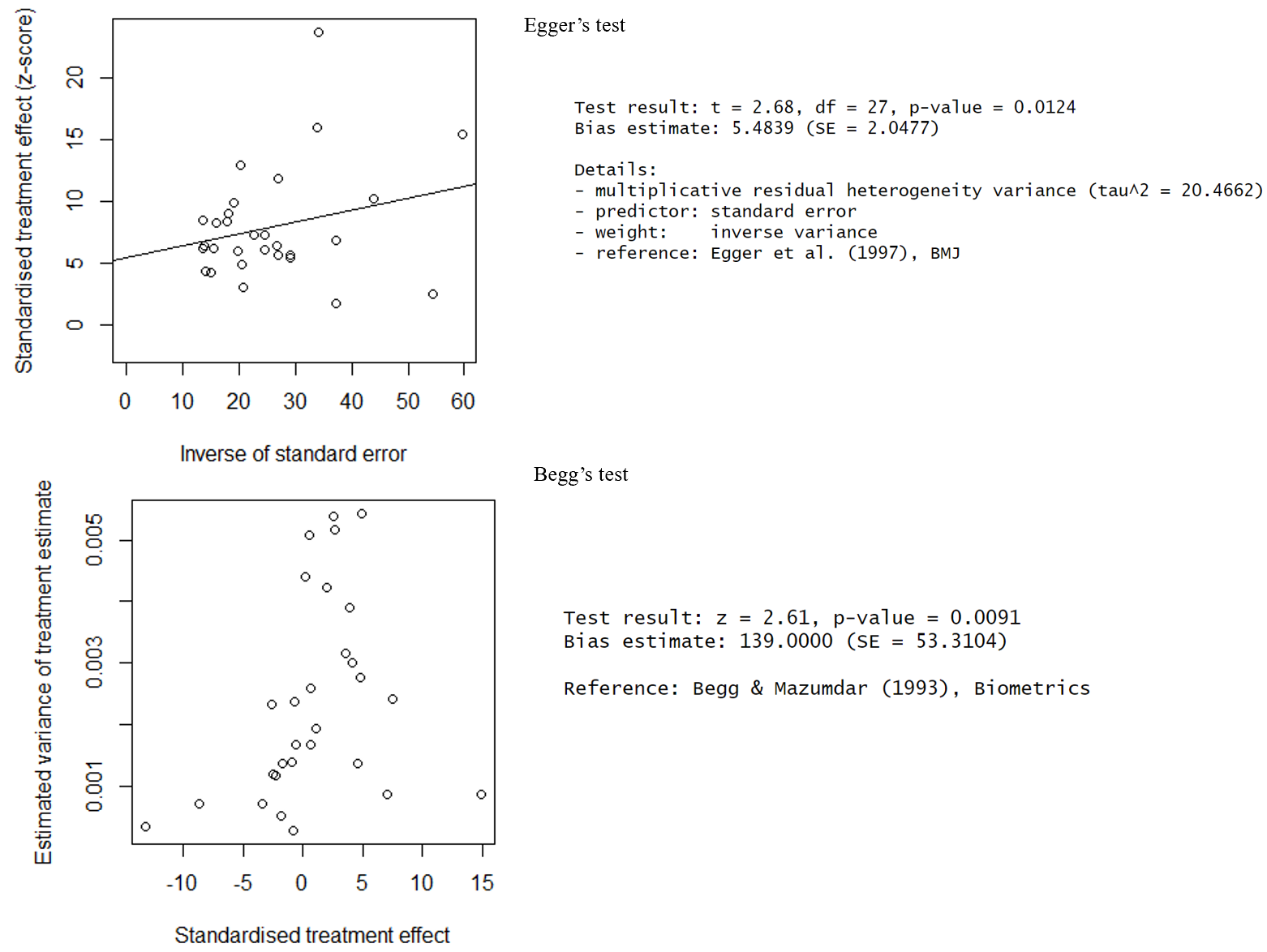

Supplement: S5 Fig — (PNG) [file pone.0318920.s005.png]
