# Supplementary material for: Prevalence and correlation of sarcopenia with Alzheimer’s disease: A systematic review and meta-analysis
Source: PLoS One. 2025 Mar 3;20(3):e0318920. doi: 10.1371/journal.pone.0318920 (PMC11875368; doi:10.1371/journal.pone.0318920)
Supplement: S1 Table — (DOCX) [file pone.0318920.s006.docx]

**S1 Table. AHRQ cross-sectional study evaluation criteria**

| Study | (1) Define the source of information (survey, record review) | (2) List inclusion and exclusion criteria for exposed and unexposed subjects (cases and controls) or refer to previous publications | (3) Indicate time period used for identifying patients | (4) Indicate whether or not subjects were consecutive if not population-based | (5) Indicate if evaluators of subjective components of study were masked to other aspects of the status participants | (6) Describe any assessments undertaken for quality assurance purposes (e.g., test/retest of primary outcome measurements) | (7) Explain any patient exclusions from analysis | (8) Describe how confounding was assessed and/or controlled. | (9) If applicable, explain how missing data were handled in the analysis | (10) Summarize patient response rates and completeness of data collection | (11) Clarify what follow-up, if any, was expected  and the percentage of patients for which incomplete data or follow-up was obtained | Score |
| --- | --- | --- | --- | --- | --- | --- | --- | --- | --- | --- | --- | --- |
| Yusuke Ogawa 2018 | YES | YES | NO | UNCLEAR | UNCLEAR | YES | UNCLEAR | YES | NO | YES | NO | 5 |
| Giulia Bramato 2022 | YES | YES | YES | YES | UNCLEAR | YES | YES | NO | NO | YES | NO | 7 |
| Shanwen Liu 2023 | YES | YES | YES | YES | UNCLEAR | YES | YES | YES | NO | YES | NO | 8 |
| T Sugimoto 2022 | YES | YES | YES | YES | UNCLEAR | YES | YES | YES | YES | YES | NO | 9 |
| Ai Kimura 2018 | YES | YES | YES | YES | UNCLEAR | YES | YES | YES | YES | YES | NO | 9 |
| Cemile Özsürekci 2019 | YES | YES | UNCLEAR | YES | UNCLEAR | YES | YES | NO | YES | YES | NO | 7 |
| Pelin Unsal 2023 | YES | YES | YES | YES | UNCLEAR | YES | NO | YES | NO | NO | UNCLEAR | 6 |
| Liss Elin Larsson 2023 | YES | YES | YES | YES | UNCLEAR | YES | YES | YES | YES | YES | UNCLEAR | 9 |
| Zekeriya Ülger 2022 | YES | YES | YES | YES | UNCLEAR | YES | NO | YES | NO | YES | UNCLEAR | 7 |
| Daisuke Hirose 2016 | YES | YES | NO | UNCLEAR | UNCLEAR | YES | NO | NO | NO | NO | UNCLEAR | 3 |
| Fatma Sena Dost 2022 | YES | YES | YES | YES | UNCLEAR | YES | YES | YES | YES | YES | UNCLEAR | 9 |
| Akito Tsugawa 2017 | YES | YES | NO | UNCLEAR | UNCLEAR | YES | NO | NO | NO | YES | UNCLEAR | 4 |
| Xiaofen Weng 2023 (a) | YES | YES | YES | YES | UNCLEAR | YES | NO | YES | NO | NO | UNCLEAR | 6 |
| Taichi Demura 2023 | YES | YES | NO | UNCLEAR | UNCLEAR | YES | NO | YES | NO | NO | UNCLEAR | 4 |
| Taiki Sugimoto 2016 | YES | NO | YES | YES | UNCLEAR | YES | YES | YES | YES | YES | UNCLEAR | 8 |
| Xiaofen Weng 2023 (b) | YES | YES | YES | YES | UNCLEAR | YES | NO | YES | UNCLEAR | NO | UNCLEAR | 6 |
| L Tay 2018 | YES | YES | YES | YES | UNCLEAR | YES | NO | YES | NO | YES | UNCLEAR | 7 |
| Fatma Sena Dost 2023 | YES | YES | YES | YES | UNCLEAR | YES | UNCLEAR | YES | UNCLEAR | YES | UNCLEAR | 7 |
| Odete Vicente de Sousa 2022 | YES | YES | YES | YES | UNCLEAR | YES | NO | YES | NO | NO | UNCLEAR | 6 |
| Mei Sian Chong 2015 | YES | YES | YES | YES | UNCLEAR | YES | UNCLEAR | YES | NO | YES | UNCLEAR | 7 |
| Veysel SUZAN 2022 | YES | YES | YES | YES | UNCLEAR | YES | UNCLEEAR | YES | UNCLEAR | NO | UNCLEAR | 6 |
| Osamu Iritani 2021 | YES | YES | NO | UNCLEAR | UNCLEAR | YES | NO | YES | NO | YES | UNCLEAR | 5 |
| Danielle Rodrigues Lecheta 2017 | YES | YES | YES | SYES | UNCLEAR | YES | YES | YES | NO | NO | UNCLEAR | 7 |
| Hsin Ning Lee 2020 | YES | YES | YES | YES | UNCLEAR | YES | NO | YES | NO | NO | UNCLEAR | 6 |
| Shanwen Liu 2022 | YES | YES | YES | YES | UNCLEAR | YES | NO | YES | NO | NO | UNCLEAR | 6 |
| Taiki Sugimoto 2017 | YES | YES | NO | UNCLEAR | UNCLEAR | YES | YES | YES | YES | UNCLEAR | UNCLEAR | 6 |
