# Supplementary material for: Prevalence and correlation of sarcopenia with Alzheimer’s disease: A systematic review and meta-analysis
Source: PLoS One. 2025 Mar 3;20(3):e0318920. doi: 10.1371/journal.pone.0318920 (PMC11875368; doi:10.1371/journal.pone.0318920)
Supplement: S2 Table — (DOCX) [file pone.0318920.s007.docx]

**S2 Table.** The NOS score for cohort studies

| **Study** | **Selection (1)** | | | | **Comparability (2)** | **Outcome (3)** | | | **Score** |
| --- | --- | --- | --- | --- | --- | --- | --- | --- | --- |
|  |  |  |  |  |  |  |  |  |  |
|  | Representativeness of the exposed cohort | Selection of the non-exposed cohort | Ascertainment of exposure | Demonstration that outcome of Interest was not Present at start of study | Comparability of cohorts on the basis of the design or analysis | Assessment of outcome | Was follow-up long enough for outcomes to occur | Adequacy of follow up of cohorts |  |
| Michal 2021 | ✮ | ✮ | ✮ |  | ✮✮ | ✮ | ✮ | ✮ | 8 |
