# Supplementary material for: Prevalence and correlation of sarcopenia with Alzheimer’s disease: A systematic review and meta-analysis
Source: PLoS One. 2025 Mar 3;20(3):e0318920. doi: 10.1371/journal.pone.0318920 (PMC11875368; doi:10.1371/journal.pone.0318920)
Supplement: S3 Table — (DOC) [file pone.0318920.s008.doc]

**S3 Table. The numbered table of all studies identified in the literature search**

| **Studies identified in the literature search (N = 1793)** | |
| --- | --- |
| **The included studies** | **Citations** |
| n = 27 | [1] OGAWA Y, KANEKO Y, SATO T, et al. Sarcopenia and Muscle Functions at Various Stages of Alzheimer Disease [J]. Front Neurol, 2018, 9: 710.  [2] BRAMATO G, BARONE R, BARULLI M R, et al. Sarcopenia screening in elderly with Alzheimer's disease: performances of the SARC-F-3 and MSRA-5 questionnaires [J]. BMC Geriatr, 2022, 22(1): 761.  [3] LIU S, ZHUANG S, LI M, et al. Relationship between sarcopenia and sleep status in female patients with mild to moderate Alzheimer's disease [J]. Psychogeriatrics, 2023, 23(1): 94-107.  [4] SUGIMOTO T, KURODA Y, MATSUMOTO N, et al. Cross-Sectional Associations of Sarcopenia and Its Components with Neuropsychological Performance among Memory Clinic Patients with Mild Cognitive Impairment and Alzheimer's Disease [J]. J Frailty Aging, 2022, 11(2): 182-9.  [5] KIMURA A, SUGIMOTO T, NIIDA S, et al. Association Between Appetite and Sarcopenia in Patients With Mild Cognitive Impairment and Early-Stage Alzheimer's Disease: A Case-Control Study [J]. Front Nutr, 2018, 5: 128.  [6] ÖZSüREKCI C, ARSLAN S S, DEMIR N, et al. Timing of Dysphagia Screening in Alzheimer's Dementia [J]. JPEN J Parenter Enteral Nutr, 2020, 44(3): 516-24.  [7] UNSAL P, GUNER M, OZSUREKCI C, et al. Prevalence of nutrition disorders and nutrition-related conditions in older patients with Alzheimer's disease [J]. Nutr Clin Pract, 2023, 38(5): 1142-53.  [8] LARSSON L E, WANG R, CEDERHOLM T, et al. Association of Sarcopenia and Its Defining Components with the Degree of Cognitive Impairment in a Memory Clinic Population [J]. J Alzheimers Dis, 2023, 96(2): 777-88.  [9] ÜLGER Z, AYçIçEK G, KARA Ö, et al. Ultrasonographic/regional muscle measurements for diagnosing sarcopenia in older adults with and without dementia [J]. Turk J Med Sci, 2022, 52(6): 1926-32.  [10] HIROSE D, HANYU H, FUKASAWA R, et al. Frailty and sarcopenia in subjects with Alzheimer's disease with or without cerebrovascular disease [J]. Geriatr Gerontol Int, 2016, 16(11): 1235-6.  [11] DOST F S, ATES BULUT E, DOKUZLAR O, et al. Sarcopenia is as common in older patients with dementia with Lewy bodies as it is in those with Alzheimer's disease [J]. Geriatr Gerontol Int, 2022, 22(5): 418-24.  [12] TSUGAWA A, OGAWA Y, TAKENOSHITA N, et al. Decreased Muscle Strength and Quality in Diabetes-Related Dementia [J]. Dement Geriatr Cogn Dis Extra, 2017, 7(3): 454-62.  [13] WENG X, LIU S, LI M, et al. White matter hyperintensities: a possible link between sarcopenia and cognitive impairment in patients with mild to moderate Alzheimer's disease [J]. Eur Geriatr Med, 2023, 14(5): 1037-47.  [14] DEMURA T, OKUNO T, MIWA T, et al. Sarcopenia and decline in appendicular skeletal muscle mass are associated with hypoperfusion in key hubs of central autonomic network on 3DSRT in older adults with progression of normal cognition to Alzheimer's disease [J]. Geriatr Gerontol Int, 2023, 23(1): 16-24.  [15] SUGIMOTO T, ONO R, MURATA S, et al. Prevalence and associated factors of sarcopenia in elderly subjects with amnestic mild cognitive impairment or Alzheimer disease [J]. Curr Alzheimer Res, 2016, 13(6): 718-26.  [16] WENG X F, LIU S W, LI M, et al. Relationship between sarcopenic obesity and cognitive function in patients with mild to moderate Alzheimer's disease [J]. Psychogeriatrics, 2023, 23(6): 944-53.  [17] TAY L, LEUNG B P, WEE S, et al. Association of nutrition and immune-endocrine dysfunction with muscle mass and performance in cognitively impaired older adults [J]. Arch Gerontol Geriatr, 2018, 75: 20-7.  [18] DOST F S, ERKEN N, ONTAN M S, et al. Muscle Strength Seems to be Related to The Functional Status and Severity of Dementia in Older Adults with Alzheimer's Disease [J]. Curr Aging Sci, 2023, 16(1): 75-83.  [19] VICENTE DE SOUSA O, MENDES J, AMARAL T F. Association between nutritional and functional status indicators with caregivers' burden in Alzheimer's disease [J]. Nutr Diet, 2022, 79(3): 380-9.  [20] CHONG M S, TAY L, ISMAIL N H, et al. The Case for Stage-Specific Frailty Interventions Spanning Community Aging to Cognitive Impairment [J]. J Am Med Dir Assoc, 2015, 16(11): 1003.e13-9.  [21] SUZAN V, YAVUZER H. ASSOCIATION OF NEURODEGENERATIVE DISEASES WITH SARCOPENIA AND OTHER GERIATRIC SYNDROMES [J]. Turk Geriatri Dergisi, 2022, 25(2): 246-53.  [22] IRITANI O, OKUNO T, MIWA T, et al. Olfactory-cognitive index distinguishes involvement of frontal lobe shrinkage, as in sarcopenia from shrinkage of medial temporal areas, and global brain, as in Kihon Checklist frailty/dependence, in older adults with progression of normal cognition to Alzheimer's disease [J]. Geriatr Gerontol Int, 2021, 21(3): 291-8.  [23] LECHETA D R, SCHIEFERDECKER M E M, DE MELLO A P, et al. Nutritional problems in older adults with Alzheimer's disease: Risk of malnutrition and sarcopenia [J]. Revista De Nutricao-Brazilian Journal of Nutrition, 2017, 30(3): 273-85.  [24] LEE H N, CHANG Y S, WU Y H, et al. Sarcopenia in female patients with Alzheimer's disease are more likely to have lower levels of haemoglobin and 25-hydroxyvitamin D [J]. Psychogeriatrics, 2020, 20(6): 858-64.  [25] LIU S, ZHANG Y, PENG B, et al. Correlation between parameters related to sarcopenia and gray matter volume in patients with mild to moderate Alzheimer's disease [J]. Aging Clin Exp Res, 2022, 34(12): 3041-53.  [26] SUGIMOTO T, ONO R, MURATA S, et al. Sarcopenia is Associated With Impairment of Activities of Daily Living in Japanese Patients With Early-Stage Alzheimer Disease [J]. Alzheimer Dis Assoc Disord, 2017, 31(3): 256-8.  [27] BEERI M S, LEUGRANS S E, DELBONO O, et al. Sarcopenia is associated with incident Alzheimer's dementia, mild cognitive impairment, and cognitive decline [J]. J Am Geriatr Soc, 2021, 69(7): 1826-35. |
| **Reasons for exclusion** | **Citations** |
| Non-English paper (n = 1) | [1] YAZAR T, OLGUN YAZAR H. The prevalence of sarcopenia and dynapenia according to stage among Alzheimer-type dementia patients [J]. Ideggyogy Sz, 2019, 72(5-6): 171-9. |
| Outcome measurement (n = 8) | [1] KARIM A, IQBAL M S, MUHAMMAD T, et al. Elevated plasma zonulin and CAF22 are correlated with sarcopenia and functional dependency at various stages of Alzheimer's diseases [J]. Neurosci Res, 2022, 184: 47-53.  [2] OHTA Y, NOMURA E, HATANAKA N, et al. Female dominant association of sarcopenia and physical frailty in mild cognitive impairment and Alzheimer's disease [J]. J Clin Neurosci, 2019, 70: 96-101.  [3] CHEN X, HAN P, YU X, et al. Relationships between sarcopenia, depressive symptoms, and mild cognitive impairment in Chinese community-dwelling older adults [J]. J Affect Disord, 2021, 286: 71-7.  [4] CHOU H H, LAI T J, YEN C H, et al. Sarcopenic Obesity Tendency and Nutritional Status Is Related to the Risk of Sarcopenia, Frailty, Depression and Quality of Life in Patients with Dementia [J]. Int J Environ Res Public Health, 2022, 19(5).  [5] KOHARA K, OKADA Y, OCHI M, et al. Muscle mass decline, arterial stiffness, white matter hyperintensity, and cognitive impairment: Japan Shimanami Health Promoting Program study [J]. J Cachexia Sarcopenia Muscle, 2017, 8(4): 557-66.  [6] LIN A, WANG T, LI C, et al. Association of Sarcopenia with Cognitive Function and Dementia Risk Score: A National Prospective Cohort Study [J]. Metabolites, 2023, 13(2).  [7] MANISCALCO L, VERONESE N, RAGUSA F S, et al. Sarcopenia using muscle mass prediction model and cognitive impairment: A longitudinal analysis from the English longitudinal study on ageing [J]. Arch Gerontol Geriatr, 2024, 117: 105160.  [8] MOON Y, MOON W J, KIM I O, et al. Muscle Strength Is Independently Related to Brain Atrophy in Patients with Alzheimer's Disease [J]. Dementia and Geriatric Cognitive Disorders, 2019, 47(4-6): 306-14. |
| Without specified diagnostic criteria for AD/sarcopenia (n = 8) | [1] SALINAS-RODRíGUEZ A, PALAZUELOS-GONZáLEZ R, RIVERA-ALMARAZ A, et al. Longitudinal association of sarcopenia and mild cognitive impairment among older Mexican adults [J]. J Cachexia Sarcopenia Muscle, 2021, 12(6): 1848-59.  [2] CHO J, PARK M, MOON W J, et al. Sarcopenia in patients with dementia: correlation of temporalis muscle thickness with appendicular muscle mass [J]. Neurol Sci, 2022, 43(5): 3089-95.  [3] CAVAZZOTTO T G, DE CAMPOS C D V, MAZUR C E, et al. Association between cognitive performance and sarcopenic obesity in older adults with Alzheimer's disease [J]. Dement Neuropsychol, 2022, 16(1): 28-32.  [4] SOYSAL P, TAN S G. The prevalence and co-incidence of geriatric syndromes in older patients with early-stage Alzheimer's disease and dementia with Lewy bodies [J]. Aging Clin Exp Res, 2021, 33(9): 2599-603.  [5] YILDIRIM D D, KIRAZ M B, TASDELEN B, et al. Investigation of the complex structure between the severity of alzheimer’s disease and influencing factors using latent class cluster analysis [J]. Neurological Sciences and Neurophysiology, 2021, 38(2): 120-6.  [6] TAKAGI D, HIRANO H, WATANABE Y, et al. Relationship between skeletal muscle mass and swallowing function in patients with Alzheimer's disease [J]. Geriatr Gerontol Int, 2017, 17(3): 402-9.  [7] CINTRA M T, REZENDE N A, TORRES H O. Advanced dementia in a sample of Brazilian elderly: Sociodemographic and morbidity analysis [J]. Rev Assoc Med Bras (1992), 2016, 62(8): 735-41.  [8] GüNER OYTUN M, TOPUZ S, BAŞ A O, et al. Relationships of Fall Risk With Frailty, Sarcopenia, and Balance Disturbances in Mild-to-Moderate Alzheimer's Disease [J]. J Clin Neurol, 2023, 19(3): 251-9. |
| Full text unavailable (n = 1) | [1] GILLETTE-GUYONNET S, NOURHASHEMI F, ANDRIEU S, et al. Determination of appendicular muscle mass by dual energy X-ray absorptiometry method in women with sarcopenia and Alzheimer's disease [J]. J Nutr Health Aging, 2000, 4(3): 165-9. |
| Irrelevant studies (n = 767) | [1] Stanley Prusiner gets Nobel Prize for prion discovery [J]. Australasian Biotechnology, 1997, 7(5): 275-7.  [2] ABADILLA K A, DOBS A S. Topical Testostrone Supplementation for the Treatment of Male Hypogonadism [J]. Drugs, 2012, 72(12): 1591-603.  [3] ABATI E, GAGLIARDI D, MANINI A, et al. Investigating the prevalence of MFN2 mutations in amyotrophic lateral sclerosis: insights from an Italian cohort [J]. Brain Commun, 2024, 6(5): fcae312.  [4] ABAY R J Y, GOLD L S, CAWTHON P M, et al. Lean mass, grip strength, and hospital-associated disability among older adults in Health ABC [J]. Alzheimers Dement, 2022, 18(10): 1898-906.  [5] ABE S, EZAKI O, SUZUKI M. Medium-Chain Triglycerides (8:0 and 10:0) Increase Mini-Mental State Examination (MMSE) Score in Frail Elderly Adults in a Randomized Controlled Trial [J]. J Nutr, 2020, 150(9): 2383-90.  [6] AHMAD W, EBERT P R. 5-Methoxyindole-2-carboxylic acid (MICA) suppresses Aβ-mediated pathology in <i>C-elegans</i> [J]. Experimental Gerontology, 2018, 108: 215-25.  [7] AKITA K, HARADA K, ICHIHARA J, et al. A novel selective androgen receptor modulator, NEP28, is efficacious in muscle and brain without serious side effects on prostate [J]. Eur J Pharmacol, 2013, 720(1-3): 107-14.  [8] AKSU F, CHRISTEN H J, HANEFELD F. [Progressive ataxia and distal muscular atrophy--differential diagnostic considerations on Roussy-Lévy syndrome] [J]. Klin Padiatr, 1986, 198(2): 114-8.  [9] AL KHLEIFAT A, IACOANGELI A, JONES A R, et al. Telomere length analysis in amyotrophic lateral sclerosis using large-scale whole genome sequence data [J]. Frontiers in Cellular Neuroscience, 2022, 16.  [10] ALDUNATE R, MINNITI A N, REBOLLEDO D, et al. Synaptic defects associated with s-inclusion body myositis are prevented by copper [J]. Biometals, 2012, 25(4): 815-24.  [11] ALEMI M, PRIGIONE A, WONG A, et al. Mitochondrial DNA deletions inhibit proteasomal activity and stimulate an autophagic transcript [J]. Free Radic Biol Med, 2007, 42(1): 32-43.  [12] ALHARTHI H M, ALMURDI M M. Association between cognitive impairment and motor dysfunction among patients with multiple sclerosis: a cross-sectional study [J]. Eur J Med Res, 2023, 28(1): 110.  [13] ALVAREZ K L F, AGUILAR-PINEDA J A, ORTIZ-MANRIQUE M M, et al. Co-occurring pathogenic variants in 6q27 associated with dementia spectrum disorders in a Peruvian family [J]. Frontiers in Molecular Neuroscience, 2023, 16.  [14] ANAND R S, GANESAN D, SELVAM S, et al. Distinct utilization of biotin in and between adipose and brain during aging is associated with a lipogenic shift in Wistar rat brain [J]. Nutr Res, 2020, 79: 68-76.  [15] ANDO T, YOKOI F, RIKU Y, et al. The hot cross bun sign in corticobasal degeneration [J]. Neuropathology, 2021, 41(5): 376-80.  [16] ANDRéS-BENITO P, POVEDANO M, DOMíNGUEZ R, et al. Increased C-X-C Motif Chemokine Ligand 12 Levels in Cerebrospinal Fluid as a Candidate Biomarker in Sporadic Amyotrophic Lateral Sclerosis [J]. Int J Mol Sci, 2020, 21(22).  [17] ANZOVINO A, CANEPA E, ALVES M, et al. Amyloid Beta Oligomers Activate Death Receptors and Mitochondria-Mediated Apoptotic Pathways in Cerebral Vascular Smooth Muscle Cells; Protective Effects of Carbonic Anhydrase Inhibitors [J]. Cells, 2023, 12(24).  [18] APRAHAMIAN I, COATS A J, MORLEY J E, et al. Anorexia of aging: An international assessment of healthcare providers' knowledge and practice gaps [J]. J Cachexia Sarcopenia Muscle, 2023, 14(6): 2779-92.  [19] ARATA H, TAKASHIMA H, HIRANO R, et al. Early clinical signs and imaging findings in Gerstmann-Sträussler-Scheinker syndrome (Pro102Leu) [J]. Neurology, 2006, 66(11): 1672-8.  [20] ARBOLEDA-VELASQUEZ J F, MANENT J, LEE J H, et al. Hypomorphic Notch 3 alleles link Notch signaling to ischemic cerebral small-vessel disease [J]. Proceedings of the National Academy of Sciences of the United States of America, 2011, 108(21): E128-E35.  [21] ARNULF I. REM sleep behavior disorder: An overt access to motor and cognitive control during sleep [J]. Revue Neurologique, 2010, 166(10): 785-92.  [22] AROCH I, OHAD D G, BANETH G. Paresis and unusual electrocardiographic signs in a severely hypomagnesaemic, hypocalcaemic lactating bitch [J]. Journal of Small Animal Practice, 1998, 39(6): 299-302.  [23] ATES BULUT E, SOYSAL P, ISIK A T. Frequency and coincidence of geriatric syndromes according to age groups: single-center experience in Turkey between 2013 and 2017 [J]. Clin Interv Aging, 2018, 13: 1899-905.  [24] AUAIS M, MORIN S, NADEAU L, et al. Changes in frailty-related characteristics of the hip fracture population and their implications for healthcare services: evidence from Quebec, Canada [J]. Osteoporos Int, 2013, 24(10): 2713-24.  [25] AUDIRA G, NGOC ANH N T, NGOC HIEU B T, et al. Evaluation of the Adverse Effects of Chronic Exposure to Donepezil (An Acetylcholinesterase Inhibitor) in Adult Zebrafish by Behavioral and Biochemical Assessments [J]. Biomolecules, 2020, 10(9).  [26] AUDOUARD E, VAN HEES L, SUAIN V, et al. Motor Deficit in a Tauopathy Model Is Induced by Disturbances of Axonal Transport Leading to Dying-Back Degeneration and Denentation of Neuromuscular Junctions [J]. American Journal of Pathology, 2015, 185(10): 2685-97.  [27] AULITZKY A, FRIEDRICH K, GLäSER D, et al. A complex form of hereditary spastic paraplegia in three siblings due to somatic mosaicism for a novel <i>SPAST</i> mutation in the mother [J]. Journal of the Neurological Sciences, 2014, 347(1-2): 352-5.  [28] AURANEN M, YLIKALLIO E, SHCHERBII M, et al. CHCHD10 variant p.(Gly66Val) causes axonal Charcot-Marie-Tooth disease [J]. Neurol Genet, 2015, 1(1): e1.  [29] AUYEUNG T W, KWOK T, LEE J, et al. Functional decline in cognitive impairment--the relationship between physical and cognitive function [J]. Neuroepidemiology, 2008, 31(3): 167-73.  [30] AUYEUNG T W, KWOK T, LEE J, et al. Functional decline in cognitive impairment - The relationship between physical and cognitive function [J]. Neuroepidemiology, 2008, 31(3): 167-73.  [31] AYATA C. CADASIL Experimental Insights From Animal Models [J]. Stroke, 2010, 41(10): S129-S34.  [32] AYYADEVARA S, BALASUBRAMANIAM M, SURI P, et al. Proteins that accumulate with age in human skeletal-muscle aggregates contribute to declines in muscle mass and function in Caenorhabditis elegans [J]. Aging (Albany NY), 2016, 8(12): 3486-97.  [33] BABIZHAYEV M A. Biochemical, Biomedical and Metabolic Aspects of Imidazole-Containing Dipeptides with the Inherent Complexity to Neurodegenerative Diseases and Various States of Mental Well-Being: A Challenging Correction and Neurotherapeutic Pharmaceutical Biotechnology for Treating Cognitive Deficits, Depression and Intellectual Disabilities [J]. Current Pharmaceutical Biotechnology, 2014, 15(8): 738-78.  [34] BADADANI M, NALBANDIAN A, WATTS G D, et al. VCP associated inclusion body myopathy and paget disease of bone knock-in mouse model exhibits tissue pathology typical of human disease [J]. PLoS One, 2010, 5(10).  [35] BAHRMANN A, BAHRMANN P, BAUMANN J, et al. S2k guideline: Diagnosis, therapy and follow-up of diabetes mellitus in the elderly: 2nd edition 2018 - AWMF Register no. 057-017 [J]. Diabetologie und Stoffwechsel, 2018, 13(5): 423-89.  [36] BAHRMANN A, BAHRMANN P, BAUMANN J, et al. S2k Guideline Diagnosis, Therapy and Follow-up of Diabetes mellitus in Old Age [J]. Diabetologie Und Stoffwechsel, 2018, 13(5): 423-89.  [37] BANDA K J, CHU H, CHEN R, et al. Prevalence of Oropharyngeal Dysphagia and Risk of Pneumonia, Malnutrition, and Mortality in Adults Aged 60 Years and Older: A Meta-Analysis [J]. Gerontology, 2022, 68(8): 841-53.  [38] BANNWARTH S, AIT-EL-MKADEM S, CHAUSSENOT A, et al. A mitochondrial origin for frontotemporal dementia and amyotrophic lateral sclerosis through <i>CHCHD10</i> involvement [J]. Brain, 2014, 137: 2329-45.  [39] BARAñANO D E, SNYDER S H. Neural roles for heme oxygenase:: Contrasts to nitric oxide synthase [J]. Proceedings of the National Academy of Sciences of the United States of America, 2001, 98(20): 10996-1002.  [40] BARONE R, BRAMATO G, GNONI V, et al. Sarcopenia in subjects with Alzheimer's disease: prevalence and comparison of agreement between EGWSOP1, EGWSOP2, and FNIH criteria [J]. BMC Geriatr, 2024, 24(1): 278.  [41] BARRY H C, RICH B S E, CARLSON R T. How Exercise Can Benefit Older Patients [J]. Phys Sportsmed, 1993, 21(2): 124-40.  [42] BASUN H, ALMKVIST O, AXELMAN K, et al. Clinical characteristics of a chromosome 17-linked rapidly progressive familial frontotemporal dementia [J]. Archives of Neurology, 1997, 54(5): 539-44.  [43] BATSIS J A, HAUDENSCHILD C, ROTH R M, et al. Incident Impaired Cognitive Function in Sarcopenic Obesity: Data From the National Health and Aging Trends Survey [J]. J Am Med Dir Assoc, 2021, 22(4): 865-72.e5.  [44] BELAYA I, KUCHáRIKOVá N, GóROVá V, et al. Regular Physical Exercise Modulates Iron Homeostasis in the 5xFAD Mouse Model of Alzheimer's Disease [J]. International Journal of Molecular Sciences, 2021, 22(16).  [45] BENATAR M, WUU J, FERNANDEZ C, et al. Motor neuron involvement in multisystem proteinopathy: Implications for ALS [J]. Neurology, 2013, 80(20): 1874-80.  [46] BERNARD B L, BRACEY L E, LANE K A, et al. Correlation Between Caregiver Reports of Physical Function and Performance-based Measures in a Cohort of Older Adults With Alzheimer Disease [J]. Alzheimer Dis Assoc Disord, 2016, 30(2): 169-74.  [47] BERSANO A, DEL BO R, LAMPERTI C, et al. Inclusion body myopathy and frontotemporal dementia caused by a novel <i>VCP</i> mutation [J]. Neurobiology of Aging, 2009, 30(5): 752-8.  [48] BETANCOR M, MORENO-MARTíNEZ L, LóPEZ-PéREZ Ó, et al. Therapeutic Assay with the Non-toxic C-Terminal Fragment of Tetanus Toxin (TTC) in Transgenic Murine Models of Prion Disease [J]. Mol Neurobiol, 2021, 58(10): 5312-26.  [49] BEZDICEK O, NIKOLAI T, NEPOZITEK J, et al. Prospective memory impairment in idiopathic REM sleep behavior disorder [J]. Clinical Neuropsychologist, 2018, 32(5): 1019-37.  [50] BIASIN F, CEOLIN C, CELLI S, et al. Interrelation between functional decline and dementia: The potential role of balance assessment [J]. Hum Mov Sci, 2023, 89: 103095.  [51] BIASIN F, CEOLIN C, CELLI S, et al. Interrelation between functional decline and dementia: The role of balance assessment [J]. Human Movement Science, 2023, 89.  [52] BIDOOKI S, JACKSON M J, JOHNSON M A, et al. Sporadic mitochondrial myopathy due to a new mutation in the mitochondrial tRNASer(UCN) gene [J]. Neuromuscul Disord, 2004, 14(7): 417-20.  [53] BIDOOKI S, JACKSON M J, JOHNSON M A, et al. Sporadic mitochondrial myopathy due to a new mutation in the mitochondrial tRNA<SUP>Ser(UCN)</SUP> gene [J]. Neuromuscular Disorders, 2004, 14(7): 417-20.  [54] BINDER J, HOFMANN S, KREISEL S, et al. Clinical and molecular findings in a patient with a novel mutation in the deafness-dystonia peptide (DDP1) gene [J]. Brain, 2003, 126: 1814-20.  [55] BLANCHARD J, CHOHAN M O, LI B, et al. Beneficial effect of a CNTF tetrapeptide on adult hippocampal neurogenesis, neuronal plasticity, and spatial memory in mice [J]. J Alzheimers Dis, 2010, 21(4): 1185-95.  [56] BLASS J P, CYRUS P A, BIEBER F, et al. Randomized, double-blind, placebo-controlled, multicenter study to evaluate the safety and tolerability of metrifonate in patients with probable Alzheimer disease. The Metrifonate Study Group [J]. Alzheimer Dis Assoc Disord, 2000, 14(1): 39-45.  [57] BLASS J P, CYRUS P A, BIEBER F, et al. Randomized, double-blind, placebo-controlled, multicenter study to evaluate the safety and tolerability of metrifonate in patients with probable Alzheimer disease [J]. Alzheimer Disease and Associated Disorders, 2000, 14(1): 39-45.  [58] BLE A, CHERUBINI A, VOLPATO S, et al. Lower plasma vitamin e levels are associated with the frailty syndrome: The InCHIANTI study [J]. Journals of Gerontology Series a-Biological Sciences and Medical Sciences, 2006, 61(3): 278-83.  [59] BLONDET B, AïT-IKHLEF A, MURAWSKY M, et al. Transient massive DNA fragmentation in nervous system during the early course of a murine neurodegenerative disease [J]. Neuroscience Letters, 2001, 305(3): 202-6.  [60] BODICK N C, OFFEN W W, LEVEY A I, et al. Effects of xanomeline, a selective muscarinic receptor agonist, on cognitive function and behavioral symptoms in Alzheimer disease [J]. Archives of Neurology, 1997, 54(4): 465-73.  [61] BOLLHEIMER L C, VOLKERT D, BERTSCH T, et al. Translational research in geriatrics?: A plea based on current biomedical key publications [Z]. Translationale Forschung in der Geriatrie?: Ein Plädoyer anhand aktueller biomedizinischer Schlüsselpublikationen. 2013: 569-76.10.1007/s00391-012-0414-3  [62] BOLTSHAUSER E, JERUSALEM F, NIEMEYER G, et al. Kearns syndrome [J]. Schweizerische Medizinische Wochenschrift, 1977, 107(50): 1880-8.  [63] BONFIGLIO V, UMEGAKI H, KUZUYA M. Quality of life in cognitively impaired older adults [J]. Geriatr Gerontol Int, 2019, 19(10): 999-1005.  [64] BOOCHOLEZ H, MARQUES F C, LEVINE A, et al. Neuropeptide signaling and SKN-1 orchestrate differential responses of the proteostasis network to dissimilar proteotoxic insults [J]. Cell Reports, 2022, 38(6).  [65] BORDA M G, BANI HASSAN E, WEON J H, et al. Muscle Volume and Intramuscular Fat of the Tongue Evaluated With MRI Predict Malnutrition in People Living With Dementia: A 5-Year Follow-up Study [J]. J Gerontol A Biol Sci Med Sci, 2022, 77(2): 228-34.  [66] BORDA M G, CASTELLANOS-PERILLA N, TOVAR-RIOS D A, et al. Tongue muscle mass is associated with total grey matter and hippocampal volumes in Dementia with Lewy Bodies [J]. Arch Gerontol Geriatr, 2022, 100: 104647.  [67] BORDA M G, DUQUE G, PéREZ-ZEPEDA M U, et al. Using magnetic resonance imaging to measure head muscles: An innovative method to opportunistically determine muscle mass and detect sarcopenia [J]. J Cachexia Sarcopenia Muscle, 2024, 15(1): 189-97.  [68] BORDA M G, JARAMILLO-JIMENEZ A, TOVAR-RIOS D A, et al. Hippocampal subfields and decline in activities of daily living in Alzheimer's disease and dementia with Lewy bodies [J]. Neurodegenerative Disease Management, 2020, 10(6): 357-67.  [69] BORGES-MACHADO F, BARROS D, TEIXEIRA L, et al. Health-related physical indicators and self-rated quality of life in older adults with neurocognitive disorder [J]. Qual Life Res, 2021, 30(8): 2255-64.  [70] BOURASSA P, TREMBLAY C, SCHNEIDER J A, et al. Brain mural cell loss in the parietal cortex in Alzheimer's disease correlates with cognitive decline and TDP-43 pathology [J]. Neuropathology and Applied Neurobiology, 2020, 46(5): 458-77.  [71] BOYLE P A, BUCHMAN A S, WILSON R S, et al. Association of Muscle Strength With the Risk of Alzheimer Disease and the Rate of Cognitive Decline in Community-Dwelling Older Persons [J]. Archives of Neurology, 2009, 66(11): 1339-44.  [72] BRANYAN K, LABELLE-DUMAIS C, WANG X W, et al. Elevated TGF8 signaling contributes to cerebral small vessel disease in mouse models of Gould syndrome [J]. Matrix Biology, 2023, 115: 48-70.  [73] BREM A S, DIMARIO C, LEVY D L. Perceived aluminum-related disease in a dialysis population. A report from the End-Stage Renal Disease Network 28 [J]. Arch Intern Med, 1989, 149(11): 2541-4.  [74] BRENNAN-KROHN T, SALLOWAY S, CORREIA S, et al. Glial Vascular Degeneration in CADASIL [J]. Journal of Alzheimers Disease, 2010, 21(4): 1393-402.  [75] BROWN R B. Parkinson's Disease Etiology: Insights and Associations with Phosphate Toxicity [J]. Int J Mol Sci, 2022, 23(15).  [76] BRUCK J, GERSTENBRAND F, PROSENZ P, et al. [ON A FAMILIAL DISEASE WITH AN UNUSUAL NEUROLOGIC SYMPTOM COMBINATION, OLIGOPHRENIA, DEMENTIA, MULTIPLE SKIN ULCERATIONS, SPLENOMEGALY AND AMINO ACID METABOLIC DISORDERS] [J]. Dtsch Z Nervenheilkd, 1964, 185: 579-97.  [77] BRUNETTI D, BOTTANI E, SEGALA A, et al. Targeting Multiple Mitochondrial Processes by a Metabolic Modulator Prevents Sarcopenia and Cognitive Decline in SAMP8 Mice [J]. Front Pharmacol, 2020, 11: 1171.  [78] BUCELLI R C, ARHZAOUY K, PESTRONK A, et al. <i>SQSTM1</i> splice site mutation in distal myopathy with rimmed vacuoles [J]. Neurology, 2015, 85(8): 665-74.  [79] BUCHMAN A S, BOYLE P A, WILSON R S, et al. Association Between Late-Life Social Activity and Motor Decline in Older Adults [J]. Archives of Internal Medicine, 2009, 169(12): 1139-46.  [80] BUCHMAN A S, CAPUANO A W, VANDERHORST V, et al. Brain β-Amyloid Links the Association of Change in Body Mass Index With Cognitive Decline in Community-Dwelling Older Adults [J]. J Gerontol A Biol Sci Med Sci, 2023, 78(2): 277-85.  [81] BUCHMAN A S, YU L, WILSON R S, et al. Post-mortem brain pathology is related to declining respiratory function in community-dwelling older adults [J]. Frontiers in Aging Neuroscience, 2015, 7.  [82] BUIGUES C, THEOU O, FONFRíA-VIVAS R, et al. Can Leucine Supplementation Improve Frailty Index Scores? [J]. Geriatrics (Basel), 2023, 8(5).  [83] BUNIATIAN G H, SCHWINGHAMMER U, TREMMEL R, et al. Consequences of Amyloid-β Deficiency for the Liver [J]. Advanced Science, 2024, 11(18).  [84] BURNS J M, JOHNSON D K, WATTS A, et al. Reduced lean mass in early Alzheimer disease and its association with brain atrophy [J]. Arch Neurol, 2010, 67(4): 428-33.  [85] BURSTEIN S R, VALSECCHI F, KAWAMATA H, et al. <i>In</i> <i>vitro</i> and <i>in</i> <i>vivo</i> studies of the ALS-FTLD protein CHCHD10 reveal novel mitochondrial topology and protein interactions [J]. Human Molecular Genetics, 2018, 27(1): 160-77.  [86] BUTTI Z, PAN Y E, GIACOMOTTO J, et al. Reduced C9orf72 function leads to defective synaptic vesicle release and neuromuscular dysfunction in zebrafish [J]. Communications Biology, 2021, 4(1).  [87] BYUN S E, KIM S, KIM K H, et al. Psoas cross-sectional area as a predictor of mortality and a diagnostic tool for sarcopenia in hip fracture patients [J]. J Bone Miner Metab, 2019, 37(5): 871-9.  [88] CABETT CIPOLLI G, SANCHES YASSUDA M, APRAHAMIAN I. Sarcopenia Is Associated with Cognitive Impairment in Older Adults: A Systematic Review and Meta-Analysis [J]. J Nutr Health Aging, 2019, 23(6): 525-31.  [89] CABRERA G T, MEIJBOOM K E, ABDALLAH A, et al. Artificial microRNA suppresses C9ORF72 variants and decreases toxic dipeptide repeat proteins in vivo [J]. Gene Ther, 2024, 31(3-4): 105-18.  [90] CABRERA G T, MEIJBOOM K E, ABDALLAH A, et al. Artificial microRNA suppresses <i>C9ORF72</i> variants and decreases toxic dipeptide repeat proteins in vivo [J]. Gene Therapy, 2024, 31(3-4): 105-18.  [91] CALLAHAN C M, BOUSTANI M A, SCHMID A A, et al. Alzheimer's disease multiple intervention trial (ADMIT): study protocol for a randomized controlled clinical trial [J]. Trials, 2012, 13: 92.  [92] CALLAHAN C M, BOUSTANI M A, SCHMID A A, et al. Targeting Functional Decline in Alzheimer Disease: A Randomized Trial [J]. Ann Intern Med, 2017, 166(3): 164-71.  [93] CAMERO S, BENíTEZ M J, JIMéNEZ J S. Anomalous protein-DNA interactions behind neurological disorders [Z]. 2013: 37-63.10.1016/B978-0-12-411637-5.00002-0  [94] CAMINA MARTíN M A, DE MATEO SILLERAS B, CARREñO ENCISO L, et al. [Changes in body composition in relation to the stage of dementia in a group of institutionalized elderly] [J]. Nutr Hosp, 2013, 28(4): 1093-101.  [95] CANDIDO J B, ZANIN C, JORGE M S G, et al. Risk of sarcopenia and health conditions in institutionalized elderly with osteoporosis [J]. Rbone-Revista Brasileira De Obesidade Nutricao E Emagrecimento, 2019, 13(83): 1106-14.  [96] CANON M E, CRIMMINS E M. Sex differences in the association between muscle quality, inflammatory markers, and cognitive decline [J]. J Nutr Health Aging, 2011, 15(8): 695-8.  [97] CAO C, HASEGAWA Y, HAYASHI K, et al. Chronic Angiotensin 1-7 Infusion Prevents Angiotensin-II-Induced Cognitive Dysfunction and Skeletal Muscle Injury in a Mouse Model of Alzheimer's Disease [J]. J Alzheimers Dis, 2019, 69(1): 297-309.  [98] CARELLI V, MUSUMECI O, CAPORALI L, et al. Syndromic parkinsonism and dementia associated with <i>OPA1</i> missense mutations [J]. Annals of Neurology, 2015, 78(1): 21-38.  [99] CARMELA M. The paradigm of amyloid precursor protein in amyotrophic lateral sclerosis: The potential role of the 682YENPTY687 motif [J]. Computational and Structural Biotechnology Journal, 2023, 21: 923-30.  [100] CARRARO U. 30 Years of Translational Mobility Medicine: 2020 Padua Muscle Days go virtual from Euganean Hills, November 19th to 21st [J]. Eur J Transl Myol, 2020, 30(4): 9437.  [101] CARTER S J, GRUBER A H, RAGLIN J S, et al. Potential health effects of dietary nitrate supplementation in aging and chronic degenerative disease [J]. Med Hypotheses, 2020, 141: 109732.  [102] CASTILLO-MARIQUEO L, GIMéNEZ-LLORT L. Impact of Behavioral Assessment and Re-Test as Functional Trainings That Modify Survival, Anxiety and Functional Profile (Physical Endurance and Motor Learning) of Old Male and Female 3xTg-AD Mice and NTg Mice with Normal Aging [J]. Biomedicines, 2022, 10(5).  [103] CASTILLO-MARIQUEO L, PéREZ-GARCíA M J, GIMéNEZ-LLORT L. Modeling Functional Limitations, Gait Impairments, and Muscle Pathology in Alzheimer's Disease: Studies in the 3xTg-AD Mice [J]. Biomedicines, 2021, 9(10).  [104] CASTRO-GOMEZ S, RADERMACHER B, TACIK P, et al. Teaching an old dog new tricks: serum troponin T as a biomarker in amyotrophic lateral sclerosis [J]. Brain Communications, 2021, 3(4).  [105] CASULA M, STEENTJES K, ARONICA E, et al. Concomitant CNS pathology in a patient with amyotrophic lateral sclerosis following poliomyelitis in childhood [J]. Clinical Neuropathology, 2011, 30(3): 111-7.  [106] CATIKKAS N M, ERDOGAN T O, REGINSTER J Y, et al. Prevalence and Determinants of Falls in Community-dwelling Older Adults in Türkiye: A Population-based Cross-sectional Study Conducted between 2014-2015 [J]. Curr Aging Sci, 2023, 16(2): 133-42.  [107] CATIKKAS N M, TUNC M, SOYSAL P. The prevalence of excessive daytime sleepiness and associated factors in older diabetic patients [J]. Aging Clin Exp Res, 2023, 35(12): 3205-14.  [108] CHAKRABORTY A, DIWAN A. Molecular mechanisms of neurodegenerative disease (NDD) [J]. Aims Molecular Science, 2023, 10(3): 171-85.  [109] CHAMBERLAIN-CARTER J, JACKSON J. Does resistance training reduce falls and improve quality of life in people with Parkinson's disease using strength training exercise programmes? [J]. Physical Therapy Reviews, 2021, 26(1): 1-9.  [110] CHANG C F, YEH Y L, CHANG H Y, et al. Prevalence and Risk Factors of Sarcopenia among Older Adults Aged ≥65 Years Admitted to Daycare Centers of Taiwan: Using AWGS 2019 Guidelines [J]. Int J Environ Res Public Health, 2021, 18(16).  [111] CHEN B, SHIN S. Bibliometric Analysis on Research Trend of Accidental Falls in Older Adults by Using Citespace-Focused on Web of Science Core Collection (2010-2020) [J]. Int J Environ Res Public Health, 2021, 18(4).  [112] CHEN B, SHIN S. Bibliometric analysis on research trend of accidental falls in older adults by using citespace—focused on web of science core collection (2010–2020) [J]. International Journal of Environmental Research and Public Health, 2021, 18(4): 1-17.  [113] CHEN H J, TOPP S D, HUI H S, et al. RRM adjacent TARDBP mutations disrupt RNA binding and enhance TDP-43 proteinopathy [J]. Brain, 2019, 142(12): 3753-70.  [114] CHEN H J, TOPP S D, HUI H S, et al. RRM adjacent <i>TARDBP</i> mutations disrupt RNA binding and enhance TDP-43 proteinopathy [J]. Brain, 2019, 142: 3753-70.  [115] CHEN J, LI C G, YANG L X, et al. MYPT1<SUP>SMKO</SUP> Mice Function as a Novel Spontaneous Age- and Hypertension-Dependent Animal Model of CSVD [J]. Translational Stroke Research, 2024, 15(3): 606-19.  [116] CHEN L, XU Y, FANG M J, et al. Case report: A Chinese patient with spinocerebellar ataxia finally confirmed as Gerstmann-Straussler-Scheinker syndrome with P102L mutation [J]. Frontiers in Neurology, 2023, 14.  [117] CHEN P C, QIN L N, LI X M, et al. The Proteasome-Associated Deubiquitinating Enzyme Usp14 Is Essential for the Maintenance of Synaptic Ubiquitin Levels and the Development of Neuromuscular Junctions [J]. Journal of Neuroscience, 2009, 29(35): 10909-19.  [118] CHEN X Z, WU Y P, YANG T H, et al. Salidroside alleviates cachexia symptoms in mouse models of cancer cachexia via activating mTOR signalling [J]. Journal of Cachexia Sarcopenia and Muscle, 2016, 7(2): 224-32.  [119] CHEN Y, ZHOU H, BINYIN W, et al. Construction of a New Protein-Protein Interaction and Molecular Biomarkers Networks in Alzheimer's Disease Patients by Bioinformatics Screening [J]. Journal of Biomedical Nanotechnology, 2023, 19(1): 154-71.  [120] CHEN Y L, TAN X Y, YAN D, et al. A Composite Fabric-Based Soft Rehabilitation Glove With Soft Joint for Dementia in Parkinsons Disease [J]. Ieee Journal of Translational Engineering in Health and Medicine, 2020, 8.  [121] CHEN Y T, YU C C, LIN Y C, et al. Brain CT can predict low lean mass in the elderly with cognitive impairment: a community-dwelling study [J]. BMC Geriatr, 2022, 22(1): 3.  [122] CHEN Z, XU Z, CHENG Q, et al. Phenotypic bases of NOTCH2NLC GGC expansion positive neuronal intranuclear inclusion disease in a Southeast Asian cohort [J]. Clin Genet, 2020, 98(3): 274-81.  [123] CHEN Z Y, XU Z Y, CHENG Q H, et al. Phenotypic bases of <i>NOTCH2NLC</i> GGC expansion positive neuronal intranuclear inclusion disease in a Southeast Asian cohort [J]. Clinical Genetics, 2020, 98(3): 274-81.  [124] CHING J K, ELIZABETH S V, JU J S, et al. mTOR dysfunction contributes to vacuolar pathology and weakness in valosin-containing protein associated inclusion body myopathy [J]. Human Molecular Genetics, 2013, 22(6): 1167-79.  [125] CHO J Y, HWANG D Y, KANG T S, et al. Use of NSE/PS2m-transgenic mice in the study of the protective effect of exercise on Alzheimer's disease [J]. J Sports Sci, 2003, 21(11): 943-51.  [126] CHO J Y, HWANG D Y, KANG T S, et al. Use of <i>NSE/PS2m</i>-transgenic mice in the study of the protective effect of exercise on Alzheimer's disease [J]. Journal of Sports Sciences, 2003, 21(11): 943-51.  [127] CHOI P G, PARK S H, JEONG H Y, et al. Geniposide attenuates muscle atrophy via the inhibition of FoxO1 in senescence-accelerated mouse prone-8 [J]. Phytomedicine, 2024, 123: 155281.  [128] CHOMPOOPONG P, MILONE M, NIU Z Y, et al. A novel missense<i> HNRNPA1</i> variant in the PY-NLS domain in a patient with late-onset distal myopathy [J]. Neuromuscular Disorders, 2022, 32(6): 521-6.  [129] CHOMPOOPONG P, OSKARSSON B, MADIGAN N N, et al. Multisystem proteinopathies (MSPs) and MSP-like disorders: Clinical-pathological-molecular spectrum [J]. Annals of Clinical and Translational Neurology, 2023, 10(4): 632-43.  [130] CHONG M S, TAY L, CHAN M, et al. Stage-Specific Relationship between Frailty and Cognitive Impairment in a Specialist Memory Clinic Setting [J]. J Frailty Aging, 2014, 3(2): 113-9.  [131] CHONG M S, TAY L, CHAN M, et al. Prospective longitudinal study of frailty transitions in a community-dwelling cohort of older adults with cognitive impairment [J]. Bmc Geriatrics, 2015, 15.  [132] CHRISTENSEN R A, SHTIFMAN A, ALLEN P D, et al. Calcium dyshomeostasis in β-amyloid and Tau-bearing skeletal myotubes [J]. Journal of Biological Chemistry, 2004, 279(51): 53524-32.  [133] CHUNG T F, SIPE J D, MCKEE A, et al. Serum amyloid A in Alzheimer's disease brain is predominantly localized to myelin sheaths and axonal membrane [J]. Amyloid-International Journal of Experimental and Clinical Investigation, 2000, 7(2): 105-10.  [134] CISTERNAS P, HENRIQUEZ J P, BRANDAN E, et al. Wnt Signaling in Skeletal Muscle Dynamics: Myogenesis, Neuromuscular Synapse and Fibrosis [J]. Molecular Neurobiology, 2014, 49(1): 574-89.  [135] CISTERNAS P, VIO C P, INESTROSA N C. Role of Wnt Signaling in Tissue Fibrosis, Lessons from Skeletal Muscle and Kidney [J]. Current Molecular Medicine, 2014, 14(4): 510-22.  [136] CLIFFORD P M, SIU G, KOSCIUK M, et al. α7 nicotinic acetylcholine receptor expression by vascular smooth muscle cells facilitates the deposition of AP peptides and promotes cerebrovascular amyloid angiopathy [J]. Brain Research, 2008, 1234: 158-71.  [137] COLUMBRES R C A, LUU V, NGUYEN M, et al. Cross-sectional study of patients with VCP multisystem proteinopathy 1 using dual-energy x-ray absorptiometry [J]. Muscle Nerve, 2024, 69(6): 699-707.  [138] CONCHINHA N V, SOKOL L, TEUWEN L A, et al. Protocols for endothelial cell isolation from mouse tissues: brain, choroid, lung, and muscle [J]. STAR Protoc, 2021, 2(3): 100508.  [139] CONCHINHA N V, SOKOL L, TEUWEN L A, et al. Protocol Protocols for endothelial cell isolation from mouse tissues: brain, choroid, lung, and muscle [J]. Star Protocols, 2021, 2(3).  [140] CORDEIRO L M, SOARES M V, DA SILVA A F, et al. Toxicity of copper and zinc alone and in combination in<i> Caenorhabditis</i><i> elegans</i> model of Huntington's disease and protective effects of rutin [J]. Neurotoxicology, 2023, 97: 120-32.  [141] CORK L C, KITT C A, STRUBLE R G, et al. Animal models of degenerative neurological disease [J]. Prog Clin Biol Res, 1987, 229: 241-69.  [142] CORTéS-VICENTE E, TURON-SANS J, GELPI E, et al. Distinct Clinical Features and Outcomes in Motor Neuron Disease Associated with Behavioural Variant Frontotemporal Dementia [J]. Dement Geriatr Cogn Disord, 2018, 45(3-4): 220-31.  [143] COX D C, GUAN X, XIA Z, et al. Increased nuclear but not cytoplasmic activities of CELF1 protein leads to muscle wasting [J]. Hum Mol Genet, 2020, 29(10): 1729-44.  [144] COX L E, FERRAIUOLO L, GOODALL E F, et al. Mutations in CHMP2B in lower motor neuron predominant amyotrophic lateral sclerosis (ALS) [J]. PLoS One, 2010, 5(3): e9872.  [145] COX L E, FERRAIUOLO L, GOODALL E F, et al. Mutations in <i>CHMP2B</i> in Lower Motor Neuron Predominant Amyotrophic Lateral Sclerosis (ALS) [J]. Plos One, 2010, 5(3).  [146] CULETTO E, SATTELLE D B. A role for <i>Caenorhabditis</i> <i>elegans</i> in understanding the function and interactions of human disease genes [J]. Human Molecular Genetics, 2000, 9(6): 869-77.  [147] CUSTER S K, NEUMANN M, LU H, et al. Transgenic mice expressing mutant forms VCP/p97 recapitulate the full spectrum of IBMPFD including degeneration in muscle, brain and bone [J]. Hum Mol Genet, 2010, 19(9): 1741-55.  [148] DAMLUJI A A, FORMAN D E, VAN DIEPEN S, et al. Older Adults in the Cardiac Intensive Care Unit: Factoring Geriatric Syndromes in the Management, Prognosis, and Process of Care: A Scientific Statement From the American Heart Association [J]. Circulation, 2020, 141(2): e6-e32.  [149] DANIELS K, BONNECHèRE B. Harnessing digital health interventions to bridge the gap in prevention for older adults [J]. Front Public Health, 2023, 11: 1281923.  [150] DAO E, HSIUNG G R, SOSSI V, et al. Cerebral Amyloid-β Deposition Is Associated with Impaired Gait Speed and Lower Extremity Function [J]. J Alzheimers Dis, 2019, 71(s1): S41-s9.  [151] DASKALOPOULOU C, WU Y T, PAN W, et al. Factors related with sarcopenia and sarcopenic obesity among low- and middle-income settings: the 10/66 DRG study [J]. Sci Rep, 2020, 10(1): 20453.  [152] DAUVILLIERS Y, SCHENCK C H, POSTUMA R B, et al. REM sleep behaviour disorder [J]. Nature Reviews Disease Primers, 2018, 4.  [153] DAVIS H M, ESSEX A L, VALDEZ S, et al. Short-term pharmacologic RAGE inhibition differentially affects bone and skeletal muscle in middle-aged mice [J]. Bone, 2019, 124: 89-102.  [154] DAVIS J, CRIBBS D H, COTMAN C W, et al. Pathogenic amyloid β-protein induces apoptosis in cultured human cerebrovascular smooth muscle cells [J]. Amyloid-International Journal of Experimental and Clinical Investigation, 1999, 6(3): 157-64.  [155] DAVIS J, WAGNER M R, ZHANG W B, et al. Amyloid β-protein stimulates the expression of urokinase-type plasminogen activator (uPA) and its receptor (uPAR) in human cerebrovascular smooth muscle cells [J]. Journal of Biological Chemistry, 2003, 278(21): 19054-61.  [156] DE CICCO V, BARRESI M, FANTOZZI M P T, et al. Oral Implant-Prostheses: New Teeth for a Brighter Brain [J]. Plos One, 2016, 11(2).  [157] DE RIDDER W, AZMI A, CLEMEN C S, et al. Multisystem proteinopathy due to a homozygous p.Arg159His <i>VCP</i> mutation: A tale of the unexpected [J]. Neurology, 2020, 94(8): E785-E96.  [158] DE SOUSA O V, MENDES J, AMARAL T F. Nutritional and Functional Indicators and Their Association With Mortality Among Older Adults With Alzheimer's Disease [J]. American Journal of Alzheimers Disease and Other Dementias, 2020, 35.  [159] DE VRIES B S, RUSTEMEIJER L M M, BAKKER L A, et al. Cognitive and behavioural changes in PLS and PMA:challenging the concept of restricted phenotypes [J]. J Neurol Neurosurg Psychiatry, 2019, 90(2): 141-7.  [160] DE WIT J, BEELEN A, VAN DEN HEERIK M S, et al. Psychological distress in partners of patients with amyotrophic lateral sclerosis and progressive muscular atrophy: what's the role of care demands and perceived control? [J]. Psychol Health Med, 2020, 25(3): 319-30.  [161] DELGADO-ESCUETA A V, GANESH S, YAMAKAWA K. Advances in the genetics of progressive myoclonus epilepsy [J]. American Journal of Medical Genetics, 2001, 106(2): 129-38.  [162] DELL' AMICO C, TATA A, PELLEGRINO E, et al. Genome editing in stem cells for genetic neurodisorders [J]. Prog Mol Biol Transl Sci, 2021, 182: 403-38.  [163] DHAR M, ARUN B G, TEBAIBIA A. Synonyms: Triple A syndrome, 4 A Syndrome, Achalasia-Addisonianism-Alacrima syndrome [J]. Anasthesiologie und Intensivmedizin, 2019, 60(1): S1-S7.  [164] DHAR M, ARUN B G, TEBAIBIA A. Allgrove Syndrome [J]. Anasthesiologie & Intensivmedizin, 2019, 60: S1-S7.  [165] DING X B, CHEN Y K, GUO C C, et al. Mutations in <i>ARHGEF15</i> cause autosomal dominant hereditary cerebral small vessel disease and osteoporotic fracture [J]. Acta Neuropathologica, 2023, 145(5): 681-705.  [166] DJAMSHIDIAN A, SCHAEFER J, HAUBENBERGER D, et al. A novel mutation in the VCP gene (G157R) in a German family with inclusion-body myopathy with Paget disease of bone and frontotemporal dementia [J]. Muscle Nerve, 2009, 39(3): 389-91.  [167] DOHERTY M J, BIRD T D, LEVERENZ J B. Alpha-synuclein in motor neuron disease: an immunohistologic study [J]. Acta Neuropathol, 2004, 107(2): 169-75.  [168] DOI H, TATEISHI J, OHTA M. Neuropathological study of amyotrophic lateral sclerosis and Parkinsonism-dementia on Guam: an analysis of 24 autopsy cases [J]. Brain and Nerve, 1982, 34(1): 63-70.  [169] DONAHUE J E, BERZIN T M, RAFII M S, et al. Agrin in Alzheimer's disease: Altered solubility and abnormal distribution within microvasculature and brain parenchyma [J]. Proceedings of the National Academy of Sciences of the United States of America, 1999, 96(11): 6468-72.  [170] DOTTI M T, DE STEFANO N, BIANCHI S, et al. A novel <i>NOTCH3</i> frameshift deletion and mitochondrial abnormalities in a patient with CADASIL [J]. Archives of Neurology, 2004, 61(6): 942-5.  [171] DRENTH H, ZUIDEMA S U, KRIJNEN W P, et al. Psychometric Properties of the MyotonPRO in Dementia Patients with Paratonia [J]. Gerontology, 2018, 64(4): 401-12.  [172] DREY M. Neurodegeneration and sarcopenia [J]. Osteologie, 2017, 26(1): 25-7.  [173] DU Q, LU Y, HU F, et al. Dietary diversity and possible sarcopenia among older people in China: a nationwide population-based study [J]. Front Nutr, 2023, 10: 1218453.  [174] DUGGER B N, MURRAY M E, BOEVE B F, et al. Neuropathological analysis of brainstem cholinergic and catecholaminergic nuclei in relation to rapid eye movement (REM) sleep behaviour disorder [J]. Neuropathology and Applied Neurobiology, 2012, 38(2): 142-52.  [175] DULSAT C. A report from the 65th Annual Meeting of the American Academy of Neurology (March 16-23, 2013, San Diego, California, USA) [J]. Drugs Today (Barc), 2013, 49(5): 341-5.  [176] DUWEL V, DE KORT J M L, BECKER C M, et al. A Cross-Sectional Study of the Physical and Mental Well-Being of Long COVID Patients in Aruba [J]. Clinical Medicine & Research, 2023, 21(2): 69-78.  [177] DZIEWULSKA D, NYCZ E, RAJCZEWSKA-OLESZKIEWICZ C, et al. Nuclear abnormalities in vascular myocytes in cerebral autosomal-dominant arteriopathy with subcortical infarcts and leukoencephalopathy (CADASIL) [J]. Neuropathology, 2018, 38(6): 601-8.  [178] EDLEFSEN K L, TAIT J F, WENER M H, et al. Utilization and diagnostic yield of neurogenetic testing at a tertiary care facility [J]. Clin Chem, 2007, 53(6): 1016-22.  [179] ELSORADY K E. Predictors of Multi-Drug Resistant Gram-Negative Bacterial Infection in Critically III Older Adults [J]. Aging Medicine and Healthcare, 2022, 13(1): 32-9.  [180] ENDO T, AKAI K, KIJIMA T, et al. An association analysis between hypertension, dementia, and depression and the phases of pre-sarcopenia to sarcopenia: A cross-sectional analysis [J]. PLoS One, 2021, 16(7): e0252784.  [181] ERCAN S, AKTAS A. Neuroprotective Effects of Sildenafil on Traumatic Brain Injury in an Experimental Rat Model [J]. Indian Journal of Neurotrauma, 2022, 19(02): 100-4.  [182] ERVIN J F, PANNELL C, SZYMANSKI M, et al. Vascular smooth muscle actin is reduced in Alzheimer disease brain: A quantitative analysis [J]. Journal of Neuropathology and Experimental Neurology, 2004, 63(7): 735-41.  [183] ESSEX A L, HUOT J R, DEOSTHALE P, et al. Triggering Receptor Expressed on Myeloid Cells 2 (TREM2) R47H Variant Causes Distinct Age- and Sex-Dependent Musculoskeletal Alterations in Mice [J]. J Bone Miner Res, 2022, 37(7): 1366-81.  [184] EYMARD B. Polymyositis, dermatomyositis and inclusion body myositis Nosological aspects [J]. Presse Medicale, 2003, 32(35): 1656-67.  [185] FANTINI M L, COSSU G, MOLARI A, et al. Sleep in Genetically Confirmed Pantothenate Kinase-Associated Neurodegeneration: A Video-Polysomnographic Study [J]. Parkinsons Disease, 2010, 2010.  [186] FANTINI M L, POSTURNA R B, MONTPLAISIR J, et al. Olfactory deficit in idiopathic rapid eye movements sleep behavior disorder [J]. Brain Research Bulletin, 2006, 70(4-6): 386-90.  [187] FENG X, PENG Y, LIU M, et al. DL-3-n-butylphthalide extends survival by attenuating glial activation in a mouse model of amyotrophic lateral sclerosis [J]. Neuropharmacology, 2012, 62(2): 1004-10.  [188] FERINI-STRAMBI L, ZUCCONI M. REM sleep behavior disorder [J]. Clinical Neurophysiology, 2000, 111: S136-S40.  [189] FERRER I, CARMONA M, BLANCO R, et al. Involvement of clusterin and the aggresome in abnormal protein deposits in myofibrillar myopathies and inclusion body myositis [J]. Brain Pathology, 2005, 15(2): 101-8.  [190] FERRERI F, PAURI F, PASQUALETTI P, et al. Motor cortex excitability in Alzheimer's disease: a transcranial magnetic stimulation study [J]. Annals of Neurology, 2003, 53(1): 102-8.  [191] FIELDING R A, GUNSTAD J, GUSTAFSON D R, et al. The paradox of overnutrition in aging and cognition [J]. Ann N Y Acad Sci, 2013, 1287: 31-43.  [192] FIELDING R A, GUNSTAD J, GUSTAFSON D R, et al. The paradox of overnutrition in aging and cognition [Z]. 2013: 31-43.10.1111/nyas.12138  [193] FIELDING R A, GUNSTAD J, GUSTAFSON D R, et al. The paradox of overnutrition in aging and cognition [M]. 2013.  [194] FONKEM E, DAYAWANSA S, STROBERG E, et al. Neurological presentations of intravascular lymphoma (IVL): meta-analysis of 654 patients [J]. BMC Neurol, 2016, 16: 9.  [195] FONSECA A C, ALMEIDA A G, SANTOS M O, et al. Neurological complications of cardiomyopathies [J]. Handb Clin Neurol, 2021, 177: 91-109.  [196] FOSTER P P, ROSENBLATT K P, KULJIŠ R O. Exercise-induced cognitive plasticity, implications for mild cognitive impairment and Alzheimer's disease [J]. Front Neurol, 2011, 2: 28.  [197] FRANCO-PALACIOS M A, MARTIN M D, WIERENGA K J, et al. Adult polyglucosan body disease with reduced glycogen branching enzyme activity and heterozygous <i>GBE1</i> mutation mimicking a low-grade glioma [J]. International Journal of Clinical and Experimental Pathology, 2016, 9(3): 4092-100.  [198] FRANZON K, ZETHELIUS B, CEDERHOLM T, et al. The impact of muscle function, muscle mass and sarcopenia on independent ageing in very old Swedish men [J]. BMC Geriatr, 2019, 19(1): 153.  [199] FU Y, LI X, WANG T, et al. The Prevalence and Agreement of Sarcopenic Obesity Using Different Definitions and Its Association with Mild Cognitive Impairment [J]. J Alzheimers Dis, 2023, 94(1): 137-46.  [200] FUMAGALLI S, POTPARA T S, LARSEN T B, et al. Frailty syndrome: an emerging clinical problem in the everyday management of clinical arrhythmias. The results of the European Heart Rhythm Association survey [J]. Europace, 2017, 19(11): 1896-902.  [201] GAIANI A, MARTINELLI I, BELLO L, et al. Diagnostic and Prognostic Biomarkers in Amyotrophic Lateral Sclerosis: Neurofilament Light Chain Levels in Definite Subtypes of Disease [J]. JAMA Neurol, 2017, 74(5): 525-32.  [202] GALVIN J E, TOLEA M I, ROSENFELD A, et al. The Quick Physical Activity Rating (QPAR) scale: A brief assessment of physical activity in older adults with and without cognitive impairment [J]. PLoS One, 2020, 15(10): e0241641.  [203] GARRUTO R M, PLATO C C, YANAGIHARA R, et al. BONE MASS IN GUAMANIAN PATIENTS WITH AMYOTROPHIC LATERAL SCLEROSIS AND PARKINSONISM-DEMENTIA [J]. American Journal of Physical Anthropology, 1989, 80(1): 107-13.  [204] GATT E R, ZILBER E, PERELMAN M, et al. Do low levels of alanine aminotransferase, a baseline marker of sarcopenia and frailty, associate with worse clinical outcomes among hospitalized COVID-19 patients? A Retrospective Cohort Study [J]. J Frailty Sarcopenia Falls, 2023, 8(3): 148-54.  [205] GATTI J R, ZHANG X J, KORCARI E, et al. Redistribution of Mature Smooth Muscle Markers in Brain Arteries in Cerebral Autosomal Dominant Arteriopathy with Subcortical Infarcts and Leukoencephalopathy [J]. Translational Stroke Research, 2019, 10(2): 160-9.  [206] GAVRIILIDOU N N, PIHLSGåRD M, ELMSTåHL S. Anthropometric reference data for elderly Swedes and its disease-related pattern [J]. European Journal of Clinical Nutrition, 2015, 69(9): 1066-75.  [207] GAZULLA J, FERRER I, IZQUIERDO-ALVAREZ S, et al. Hereditary primary lateral sclerosis and progressive nonfluent aphasia [J]. Journal of Neurology, 2019, 266(5): 1079-90.  [208] GEBAI A, GORELIK A, LI Z, et al. Structural basis for the activation of acid ceramidase [J]. Nat Commun, 2018, 9(1): 1621.  [209] GENDRON T F, PETRUCELLI L. Disease Mechanisms of <i>C9ORF72</i> Repeat Expansions [J]. Cold Spring Harbor Perspectives in Medicine, 2018, 8(4).  [210] GENIN E C, BANNWARTH S, LESPINASSE F, et al. Loss of MICOS complex integrity and mitochondrial damage, but not TDP-43 mitochondrial localisation, are likely associated with severity of CHCHD10-related diseases [J]. Neurobiol Dis, 2018, 119: 159-71.  [211] GENIN E C, BANNWARTH S, LESPINASSE F, et al. Loss of MICOS complex integrity and mitochondrial damage, but not TDP-43 mitochondrial localisation, are likely associated with severity of <i>CHCHD10</i>-related diseases [J]. Neurobiology of Disease, 2018, 119: 159-71.  [212] GENIN E C, HOUNOUM B M, BANNWARTH S, et al. Mitochondrial defect in muscle precedes neuromuscular junction degeneration and motor neuron death in CHCHD10<SUP>S59L/+</SUP> mouse [J]. Acta Neuropathologica, 2019, 138(1): 123-45.  [213] GENIN E C, PLUTINO M, BANNWARTH S, et al. <i>CHCHD10</i> mutations promote loss of mitochondrial cristae junctions with impaired mitochondrial genome maintenance and inhibition of apoptosis [J]. Embo Molecular Medicine, 2016, 8(1): 58-72.  [214] GHAZI L, YAFFE K, TAMURA M K, et al. Association of 24-Hour Ambulatory Blood Pressure Patterns with Cognitive Function and Physical Functioning in CKD [J]. Clin J Am Soc Nephrol, 2020, 15(4): 455-64.  [215] GHZAIEL I, MAALOUL S, KSILA M, et al. In Vitro Evaluation of the Effects of 7-Ketocholesterol and 7β-Hydroxycholesterol on the Peroxisomal Status: Prevention of Peroxisomal Damages and Concept of Pexotherapy [J]. Adv Exp Med Biol, 2024, 1440: 437-52.  [216] GIANNOS P, PROKOPIDIS K, RALEIGH S M, et al. Altered mitochondrial microenvironment at the spotlight of musculoskeletal aging and Alzheimer's disease [J]. Sci Rep, 2022, 12(1): 11290.  [217] GILLIOT S, BASTIJNS S, PERKISAS S, et al. Investigating sarcopenia awareness using Google Trends [J]. J Frailty Sarcopenia Falls, 2021, 6(1): 32-5.  [218] GINANNESCHI F, VOLPI N, GIANNINI F, et al. Rhabdomyolysis in an elderly multitreated patient: Multiple drug interactions after statin withdrawal [J]. Journal of the Neurological Sciences, 2014, 336(1-2): 284-7.  [219] GIULIA P, MICHELE L, ANDREA F, et al. Brain Atrophy, Anti-Smooth Muscle Antibody and Cognitive Impairment: An Association Study [J]. Aging and Disease, 2016, 7(4): 318-25.  [220] GODINHO W D N, VASCONCELOS F S L, PINTO D V, et al. High-Intense Interval Training Prevents Cognitive Impairment and Increases the Expression of Muscle Genes FNDC5 and PPARGC1A in a Rat Model of Alzheimer's Disease [J]. Current Alzheimer Research, 2022, 19(12): 830-40.  [221] GOLSHIRI K, ATAABADI E A, BRANDT R, et al. Chronic Sildenafil Treatment Improves Vasomotor Function in a Mouse Model of Accelerated Aging [J]. International Journal of Molecular Sciences, 2020, 21(13).  [222] GOODENOWE D B, HAROON J, KLING M A, et al. Targeted Plasmalogen Supplementation: Effects on Blood Plasmalogens, Oxidative Stress Biomarkers, Cognition, and Mobility in Cognitively Impaired Persons [J]. Front Cell Dev Biol, 2022, 10: 864842.  [223] GOODMAN A O G, MURGATROYD P R, MEDINA-GOMEZ G, et al. The metabolic profile of early Huntington's disease - a combined human and transgenic mouse study [J]. Experimental Neurology, 2008, 210(2): 691-8.  [224] GöTZ J, BARMETTLER R, FERRARI A, et al. <i>In vivo</i> analysis of wild-type and FTDP-17 tau transgenic mice [M]//GROWDON J H, WURTMAN R J, CORKIN S, et al. Molecular Basis of Dementia. 2000: 126-33.  [225] GRANIC A, POTTER H. Mitotic Spindle Defects and Chromosome Mis-Segregation Induced by LDL/Cholesterol-Implications for Niemann-Pick C1, Alzheimer's Disease, and Atherosclerosis [J]. Plos One, 2013, 8(4).  [226] GRöNSTEDT H, VIKSTRöM S, CEDERHOLM T, et al. Effect of Sit-to-Stand Exercises Combined With Protein-Rich Oral Supplementation in Older Persons: The Older Person's Exercise and Nutrition Study [J]. J Am Med Dir Assoc, 2020, 21(9): 1229-37.  [227] GROSS M G. RESEARCH CHALLENGES ON THE INTERACTION BETWEEN EXERCISE AND NUTRITION [J]. Anales de la Real Academia Nacional de Farmacia, 2021, 87(4): 395-401.  [228] GRüNBERGER J, LINZMAYER L, WALTER H, et al. Receptor test (pupillary dilatation after application of 0.01% tropicamide solution) and determination of central nervous activation (Fourier analysis of pupillary oscillations) in patients with Alzheimer's disease [J]. Neuropsychobiology, 1999, 40(1): 40-6.  [229] GRUZMAN A, WOOD W L, ALPERT E, et al. Common molecular signature in SOD1 for both sporadic and familial amyotrophic lateral sclerosis [J]. Proc Natl Acad Sci U S A, 2007, 104(30): 12524-9.  [230] GUERREIRO R, KARA E, LE BER I, et al. Genetic Analysis of Inherited Leukodystrophies Genotype-Phenotype Correlations in the <i>CSF1R</i> Gene [J]. Jama Neurology, 2013, 70(7): 875-82.  [231] GUIDETTI D, CASALI B, MAZZEI R L, et al. An Italian case of CADASIL with mutation CGC-TCG in codon 1006, exon 19 <i>Notch3</i> gene [J]. Neurological Sciences, 2004, 24(6): 401-6.  [232] GüNER M, BAS A O, CEYLAN S, et al. Dysphagia is closely related to frailty in mild-to-moderate Alzheimer's disease [J]. Bmc Geriatrics, 2023, 23(1).  [233] GUO H H, XIONG L, PAN J X, et al. Hepcidin contributes to Swedish mutant APP-induced osteoclastogenesis and trabecular bone loss [J]. Bone Research, 2021, 9(1).  [234] GUO J, SHANG Y, FRATIGLIONI L, et al. Individual changes in anthropometric measures after age 60 years: a 15-year longitudinal population-based study [J]. Age and Ageing, 2021, 50(5): 1666-74.  [235] GUO X, ZHAO Z, SHEN H R, et al. VCP myopathy: A family with unusual clinical manifestations [J]. Muscle & Nerve, 2019, 59(3): 365-9.  [236] GURHOLT T P, BORDA M G, PARKER N, et al. Linking sarcopenia, brain structure and cognitive performance: a large-scale UK Biobank study [J]. Brain Commun, 2024, 6(2): fcae083.  [237] GUZMáN B C F, CHAFFEY T E, PALPAGAMA T H, et al. The Interplay Between Beta-Amyloid 1-42 (Aβ<sub>1-42</sub>)-Induced Hippocampal Inflammatory Response, p-tau, Vascular Pathology, and Their Synergistic Contributions to Neuronal Death and Behavioral Deficits [J]. Frontiers in Molecular Neuroscience, 2020, 13.  [238] HAGIHARA K, NUNOMURA K, LIN B, et al. Gosha-jinki-Gan (GJG) shows anti-aging effects through suppression of TNF-α production by Chikusetsusaponin V [J]. Gene, 2022, 815: 146178.  [239] HAGIYA H, TAKASE R, HONDA H, et al. Prevalence of medical factors related to aging among older car drivers: a multicenter, cross-sectional, descriptive study [J]. BMC Geriatr, 2022, 22(1): 792.  [240] HAGLUND M, KALARIA R, SLADE J Y, et al. Differential deposition of amyloid β peptides in cerebral amyloid angiopathy associated with Alzheimer's disease and vascular dementia [J]. Acta Neuropathologica, 2006, 111(5): 430-5.  [241] HALSETH M, MAHONEY R, HSIOU J, et al. Remote respiratory resistance exercise training improves respiratory function in individuals with VCP multisystem proteinopathy [J]. Neuromuscul Disord, 2024, 34: 68-74.  [242] HAMEL E, NICOLAKAKIS N, ABOULKASSIM T, et al. Oxidative stress and cerebrovascular dysfunction in mouse models of Alzheimer's disease [J]. Experimental Physiology, 2008, 93(1): 116-20.  [243] HAMMERSEN S, BROCK M, CERVóS-NAVARRO J. Adult neuronal ceroid lipofuscinosis with clinical findings consistent with a butterfly glioma [J]. Journal of Neurosurgery, 1998, 88(2): 314-8.  [244] HAN B H, ZHOU M L, ABOUSALEH F, et al. Cerebrovascular Dysfunction in Amyloid Precursor Protein Transgenic Mice: Contribution of Soluble and Insoluble Amyloid-β Peptide, Partial Restoration via γ-Secretase Inhibition [J]. Journal of Neuroscience, 2008, 28(50): 13542-50.  [245] HANYU H. Diabetes-Related Dementia [M]//NAKABEPPU Y, NINOMIYA T. Diabetes Mellitus: A Risk Factor for Alzheimer's Disease. 2019: 147-60.  [246] HARDING A E, HOLT I J, SWEENEY M G, et al. Prenatal diagnosis of mitochondrial DNA8993 T----G disease [J]. Am J Hum Genet, 1992, 50(3): 629-33.  [247] HARDING A E, HOLT I J, SWEENEY M G, et al. Prenatal diagnosis of mitochondrial DNA(8993 T→G) disease [J]. American Journal of Human Genetics, 1992, 50(3): 629-33.  [248] HARITUNIANS T, BOULTER J, HICKS C, et al. CADASIL Notch3 mutant proteins localize to the cell surface and bind ligand [J]. Circulation Research, 2002, 90(5): 506-8.  [249] HARTIKAINEN P H, PIKKARAINEN M, HäNNINEN T, et al. Unusual clinical presentation and neuropathology in two subjects with fused-in sarcoma (FUS) positive inclusions [J]. Neuropathology, 2012, 32(1): 60-8.  [250] HATANAKA S, SASAI H, SHIDA T, et al. Association between dynapenia and cognitive decline in community-dwelling older Japanese adults: The IRIDE Cohort Study [J]. Geriatr Gerontol Int, 2024, 24 Suppl 1: 123-9.  [251] HATIPOGLU E, YURUYEN M, KESKIN E, et al. Acromegaly and aging: a comparative cross-sectional study [J]. Growth Horm IGF Res, 2015, 25(1): 47-52.  [252] HATTORI Y, KITAMURA A, TSUJI M, et al. Motor and cognitive impairment in a mouse model of ischemic carotid artery disease [J]. Neurosci Lett, 2014, 581: 1-6.  [253] HAYASHI K, HASEGAWA Y, TAKEMOTO Y, et al. Continuous intracerebroventricular injection of Porphyromonas gingivalis lipopolysaccharide induces systemic organ dysfunction in a mouse model of Alzheimer's disease [J]. Exp Gerontol, 2019, 120: 1-5.  [254] HAYASHI M, KOBAYASHI K, ISHIDA C, et al. Non-Alzheimer dementia with status spongiosus and neuronal cells loss showing unusual perineuronal structures and point mutation at 129 codon of prion protein [J]. Dementia and Geriatric Cognitive Disorders, 1997, 8(1): 55-9.  [255] HEATH D A, WRIGHT A D, BARNES A D, et al. Surgical treatment of primary hyperparathyroidism in the elderly [J]. Br Med J, 1980, 280(6229): 1406-8.  [256] HECK M V, AZIZOV M, STEHNING T, et al. Dysregulated expression of lipid storage and membrane dynamics factors in Tia1 knockout mouse nervous tissue [J]. Neurogenetics, 2014, 15(2): 135-44.  [257] HECK M V, AZIZOV M, STEHNING T, et al. Dysregulated expression of lipid storage and membrane dynamics factors in <i>Tia1</i> knockout mouse nervous tissue [J]. Neurogenetics, 2014, 15(2): 135-44.  [258] HENWOOD T, NEVILLE C, BAGULEY C, et al. Aquatic exercise for residential aged care adults with dementia: benefits and barriers to participation [J]. International Psychogeriatrics, 2017, 29(9): 1439-49.  [259] HICKEY M A, ZHU C, MEDVEDEVA V, et al. Improvement of neuropathology and transcriptional deficits in CAG 140 knock-in mice supports a beneficial effect of dietary curcumin in Huntington's disease [J]. Molecular Neurodegeneration, 2012, 7.  [260] HIGUCHI Y, HASHIGUCHI A, YUAN J, et al. Mutations in MME cause an autosomal-recessive Charcot-Marie-Tooth disease type 2 [J]. Ann Neurol, 2016, 79(4): 659-72.  [261] HIGUCHI Y, HASHIGUCHI A, YUAN J H, et al. Mutations in <i>MME</i> cause an autosomal-recessive Charcot-Marie-Tooth disease type 2 [J]. Annals of Neurology, 2016, 79(4): 659-72.  [262] HIRANO K, FUJIMAKI M, SASAZAWA Y, et al. Neuroprotective effects of memantine via enhancement of autophagy [J]. Biochem Biophys Res Commun, 2019, 518(1): 161-70.  [263] HIRAOKA A, TAMURA R, OKA M, et al. Prediction of risk of falls based on handgrip strength in chronic liver disease patients living independently [J]. Hepatol Res, 2019, 49(7): 823-9.  [264] HOF K, SHIBLY S, BERGER S. Encephalopathy and Alzheimer type II astrocytes in a post laparotomy recumbent horse [J]. Deutsche Tierarztliche Wochenschrift, 2009, 116(6): 227-32.  [265] HONG M, KANG M J, PAK S, et al. Bee venom phospholipase A2 ameliorates amyotrophic lateral sclerosis by increasing regulatory T cell population [J]. Advances in Traditional Medicine, 2022, 22(3): 599-606.  [266] HSIAO C W, PENG T I, PENG A C, et al. Long-term Aβ exposure augments mCa2+-independent mROS-mediated depletion of cardiolipin for the shift of a lethal transient mitochondrial permeability transition to its permanent mode in NARP cybrids: a protective targeting of melatonin [J]. J Pineal Res, 2013, 54(1): 107-25.  [267] HSIAO C W, PENG T I, PENG A C, et al. Long-term Ab exposure augments mCa2+-independent mROSmediated depletion of cardiolipin for the shift of a lethal transient mitochondrial permeability transition to its permanent mode in NARP cybrids: A protective targeting of melatonin [J]. Journal of Pineal Research, 2013, 54(1): 107-25.  [268] HSIAO C W, PENG T I, PENG A C, et al. Long-term Aβ exposure augments mCa<SUP>2+</SUP>-independent mROS-mediated depletion of cardiolipin for the shift of a lethal transient mitochondrial permeability transition to its permanent mode in NARP cybrids: a protective targeting of melatonin [J]. Journal of Pineal Research, 2013, 54(1): 107-25.  [269] HSIEH P I, HUANG T H, CHIOU J M, et al. Cohort profile: the Taiwan Initiative for Geriatric Epidemiological Research - a prospective cohort study on cognition [J]. Epidemiol Health, 2024, 46: e2024057.  [270] HSUEH J T, PENG T C, CHEN W L, et al. Association between frailty and a measure of cognition: a cross-sectional study on community-dwelling older adults [J]. European Geriatric Medicine, 2018, 9(1): 39-43.  [271] HUANG C, HUANG L, WANG Y, et al. 6-month consequences of COVID-19 in patients discharged from hospital: a cohort study [J]. Lancet, 2023, 401(10393): e21-e33.  [272] HUANG C L, HUANG L X, WANG Y M, et al. RETRACTED: 6-month consequences of COVID-19 in patients discharged from hospital: a cohort study (Publication with Expression of Concern. See vol. 401, pg. 90, 2023) (Retracted Article) [J]. Lancet, 2021, 397(10270): 220-32.  [273] HUANG C Y, HWANG A C, LIU L K, et al. Association of Dynapenia, Sarcopenia, and Cognitive Impairment Among Community-Dwelling Older Taiwanese [J]. Rejuvenation Res, 2016, 19(1): 71-8.  [274] HUANG P, LUO K, WANG C Y, et al. Urinary Incontinence Is Associated With Increased All-Cause Mortality in Older Nursing Home Residents: A Meta-Analysis [J]. Journal of Nursing Scholarship, 2021, 53(5): 561-7.  [275] HURTADO M L, FULLER H R, WONG A M S, et al. Proteomic mapping of differentially vulnerable pre-synaptic populations identifies regulators of neuronal stability <i>in vivo</i> [J]. Scientific Reports, 2017, 7.  [276] HWANG S H, KIM E J, HONG Y B, et al. Distal hereditary motor neuropathy type 7B with Dynactin 1 mutation [J]. Mol Med Rep, 2016, 14(4): 3362-8.  [277] HWANG S H, KIM E J, HONG Y B, et al. Distal hereditary motor neuropathy type 7B with <i>Dynactin 1</i> mutation [J]. Molecular Medicine Reports, 2016, 14(4): 3362-8.  [278] IHARA Y, NAMBA R, DEMIYA M. A case of mitochondrial encephalomyopathy - mitochondrial myopathy, encephalopathy, lactic acidosis and strokelike episodes (MELAS) [J]. Clinical Neurology, 1987, 27(8): 969-75.  [279] IKEDA M, KUWABARA T, TAKAI E, et al. Increased Neurofilament Light Chain and YKL-40 CSF Levels in One Japanese IBMPFD Patient With VCP R155C Mutation: A Clinical Case Report With CSF Biomarker Analyses [J]. Frontiers in Neurology, 2020, 11.  [280] IKEGAMI S, HARADA A, HIROKAWA N. Muscle weakness, hyperactivity, and impairment in fear conditioning in tau-deficient mice [J]. Neurosci Lett, 2000, 279(3): 129-32.  [281] IMAOKA M, NAKAO H, NAKAMURA M, et al. Associations between depressive symptoms and geriatric syndromes in community-dwelling older adults in Japan: A cross-sectional study [J]. Prev Med Rep, 2021, 22: 101353.  [282] IONESCU C, JOVANOVIC A. Rates, Variability and Associated Factors of Polypharmacy in Nursing Homes in Cyprus [J]. Aging Medicine and Healthcare, 2021, 12(4): 125-30.  [283] IRANZO A, MOLINUEVO J L, SANTAMARíA J, et al. Rapid-eye-movement sleep behaviour disorder as an early marker for a neurodegenerative disorder:: a descriptive study [J]. Lancet Neurology, 2006, 5(7): 572-7.  [284] ISHIDA C, KOMAI K, YONEZAWA K, et al. An autopsy case of an aged patient with spinocerebellar ataxia type 2 [J]. Neuropathology, 2011, 31(5): 510-8.  [285] ISHIDA T, KAWADA K, MORISAWA S, et al. Risk Factors for Pseudoaldosteronism with Yokukansan Use: Analysis Using the Japanese Adverse Drug Report (JADER) Database [J]. Biol Pharm Bull, 2020, 43(10): 1570-6.  [286] ISHIKAWA A, YAMADA M, MAKINO K, et al. Dementia and delirium in 4 patients with Machado-Joseph disease [J]. Archives of Neurology, 2002, 59(11): 1804-8.  [287] IWASAKI A, KOKUBUN N, FUNAKOSHI K, et al. Hydrocephalus due to marked enlargement of spinal roots in a patient with chronic inflammatory demyelinating polyradiculoneuropathy [J]. European Journal of Neurology, 2020, 27(11): 2385-8.  [288] JACOB L, KOSTEV K, SMITH L, et al. Sarcopenia and Mild Cognitive Impairment in Older Adults from Six Low- and Middle-Income Countries [J]. J Alzheimers Dis, 2021, 82(4): 1745-54.  [289] JADCZAK A D, EDWARDS S, VISVANATHAN R. Life-Space Mobility in Aged Care Residents: Frailty In Residential Sector over Time (FIRST) Study Findings [J]. J Am Med Dir Assoc, 2022, 23(11): 1869.e1-.e6.  [290] JADCZAK A D, ROBSON L, COOPER T, et al. The Frailty In Residential Sector over Time (FIRST) study: methods and baseline cohort description [J]. BMC Geriatr, 2021, 21(1): 99.  [291] JAMES B D, BOYLE P A, BENNETT D A, et al. Total Daily Activity Measured With Actigraphy and Motor Function in Community-dwelling Older Persons With and Without Dementia [J]. Alzheimer Disease & Associated Disorders, 2012, 26(3): 238-45.  [292] JANSSENS J, PHILTJENS S, KLEINBERGER G, et al. Investigating the role of filamin C in Belgian patients with frontotemporal dementia linked to GRN deficiency in FTLD-TDP brains [J]. Acta Neuropathologica Communications, 2015, 3.  [293] JEONG H O, PARK D, IM E, et al. Determination of the Mechanisms that Cause Sarcopenia through cDNA Microarray [J]. J Frailty Aging, 2017, 6(2): 97-102.  [294] JEONG S, KIM J. Prospective Association of Handgrip Strength with Risk of New-Onset Cognitive Dysfunction in Korean Adults: A 6-Year National Cohort Study [J]. Tohoku J Exp Med, 2018, 244(2): 83-91.  [295] JEONG Y H, LING J P, LIN S Z, et al. Tdp-43 cryptic exons are highly variable between cell types [J]. Molecular Neurodegeneration, 2017, 12.  [296] JESSE S, BRETTSCHNEIDER J, SüSSMUTH S D, et al. Summary of cerebrospinal fluid routine parameters in neurodegenerative diseases [J]. Journal of Neurology, 2011, 258(6): 1034-41.  [297] JIANG D, CHEN X, HUANG J, et al. Associations of sarcopenia, sarcopenia parameters and motoric cognitive risk syndrome in Chinese older adults [J]. Front Aging Neurosci, 2023, 15: 1302879.  [298] JIANG N, ZHOU W X, ZHANG Y X. Induced pluripotent stem cells: Powerful tools for the study of neurodegenerative diseases and clinical therapy [J]. Journal of International Pharmaceutical Research, 2016, 43(2): 183-90.  [299] JIANG S, CUI J, ZHANG L Q, et al. Role of a Urinary Biomarker in the Common Mechanism of Physical Performance and Cognitive Function [J]. Frontiers in Medicine, 2022, 9.  [300] JIN S, SUN Z Z, FANG X, et al. A patient carrying a heterozygous p.Asn267Ser TARDBP missense mutation diagnosed as ALS and only involving lower motor neurons [J]. Neurological Sciences, 2023, 44(2): 777-82.  [301] JOHNSON M, STUART G. Andersen diseas [J]. Anasthesiologie und Intensivmedizin, 2020, 61(2): S12-S9.  [302] JOHNSON S. The multifaceted and widespread pathology of magnesium deficiency [J]. Med Hypotheses, 2001, 56(2): 163-70.  [303] JOHNSTON I D A. The early diagnosis of primary hyperparathyroidism [J]. South African Journal of Surgery, 1976, 14(4): 185-9.  [304] JOUTEL A, MONET M, DOMENGA V, et al. Pathogenic mutations associated with cerebral autosomal dominant arteriopathy with subcortical infarcts and leukoencephalopathy differently affect Jagged1 binding and Notch3 activity via the RBP/JK signaling pathway [J]. American Journal of Human Genetics, 2004, 74(2): 338-47.  [305] JU J S, FUENTEALBA R A, MILLER S E, et al. Valosin-containing protein (VCP) is required for autophagy and is disrupted in VCP disease [J]. Journal of Cell Biology, 2009, 187(6): 875-88.  [306] JUNG S S, ZHANG W B, VAN NOSTRAND W E. Pathogenic Aβ induces the expression and activation of matrix metalloproteinase-2 in human cerebrovascular smooth muscle cells [J]. Journal of Neurochemistry, 2003, 85(5): 1208-15.  [307] JUTZI D, RUEPP M D. Alternative Splicing in Human Biology and Disease [J]. Methods Mol Biol, 2022, 2537: 1-19.  [308] JYVäKORPI S K, PITKäLä K H, PURANEN T M, et al. Low protein and micronutrient intakes in heterogeneous older population samples [J]. Arch Gerontol Geriatr, 2015, 61(3): 464-71.  [309] KACEM I, SGHAIER I, PEVERELLI S, et al. Optineurin in patients with Amyotrophic Lateral Sclerosis associated to atypical Parkinsonism in Tunisian population [J]. Amyotroph Lateral Scler Frontotemporal Degener, 2024, 25(1-2): 128-34.  [310] KAKINUMA Y, FURIHATA M, AKIYAMA T, et al. Donepezil, an acetylcholinesterase inhibitor against Alzheimer's dementia, promotes angiogenesis in an ischemic hindlimb model [J]. J Mol Cell Cardiol, 2010, 48(4): 680-93.  [311] KAKINUMA Y, NOGUCHI T, OKAZAKI K, et al. Antimuscle atrophy effect of nicotine targets muscle satellite cells partly through an α7 nicotinic receptor in a murine hindlimb ischemia model [J]. Transl Res, 2014, 164(1): 32-45.  [312] KALARIA R N. Cerebrovascular degeneration is related to amyloid-beta protein deposition in Alzheimer's disease [M]//DELATORRE J C, HACHINSKI V. Cerebrovascular Pathology in Alzheimer's Disease. 1997: 263-71.  [313] KALIMO H, RUCHOUX M M, VIITANEN M, et al. CADASIL: a common form of hereditary arteriopathy causing brain infarcts and dementia [J]. Brain Pathology, 2002, 12(3): 371-84.  [314] KAMATCHI K, PRIYA S, SENTHILNATHAN C V, et al. A comparative study to analyse the effectiveness of PNF versus balance exercises in Parkinsonism [J]. Indian Journal of Public Health Research and Development, 2019, 10(12): 536-41.  [315] KANG K S, YUN J W, LEE Y S. Protective effect of L-carnosine against 12-<i>O</i>-tetradecanoylphorbol-13-acetate- or hydrogen peroxide-induced apoptosis on <i>v</i>-<i>myc</i> transformed rat liver epithelial cells [J]. Cancer Letters, 2002, 178(1): 53-62.  [316] KANZAKI M, SATO M, OGAWA G, et al. A case of dementia with motor neuron disease associated with agraphia - The omission of kana letters [J]. Clinical Neurology, 2004, 44(10): 673-6.  [317] KARLSSON E S, GRöNSTEDT H K, FAXéN-IRVING G, et al. Response and Adherence of Nursing Home Residents to a Nutrition/Exercise Intervention [J]. J Am Med Dir Assoc, 2021, 22(9): 1939-45.e3.  [318] KAROLCZAK D, SAWICKA E, DORSZEWSKA J, et al. MEMANTINE - NEUROPROTECTIVE DRUG IN AGING BRAIN [J]. Polish Journal of Pathology, 2013, 64(3): 196-203.  [319] KATO T, MANABE R, IGARASHI H, et al. Candesartan prevents arteriopathy progression in cerebral autosomal recessive arteriopathy with subcortical infarcts and model [J]. Journal of Clinical Investigation, 2021, 131(22).  [320] KATO T, SEKINE Y, NOZAKI H, et al. Excessive Production of Transforming Growth Factor β1 Causes Mural Cell Depletion From Cerebral Small Vessels [J]. Frontiers in Aging Neuroscience, 2020, 12.  [321] KATZ M, WADDELL L B, YUEN M, et al. Case report: Adult-onset limb girdle muscular dystrophy in sibling pair due to novel homozygous <i>LAMA2</i> missense variant [J]. Frontiers in Neurology, 2023, 14.  [322] KAY G G, ABOU-DONIA M B, MESSER W S, et al. Antimuscarinic drugs for overactive bladder and their potential effects on cognitive function in older patients [J]. Journal of the American Geriatrics Society, 2005, 53(12): 2195-201.  [323] KAZAMEL M, SORENSON E J, MCEVOY K M, et al. Clinical spectrum of valosin containing protein (VCP)-opathy [J]. Muscle Nerve, 2016, 54(1): 94-9.  [324] KAZAMEL M, SORENSON E J, MCEVOY K M, et al. Clinical spectrum of valosin containing protein (<i>VCP</i>)-opathy [J]. Muscle & Nerve, 2016, 54(1): 94-9.  [325] KHALIL M S, KHAMIS N, AL-DREES A, et al. Does coenzyme-Q have a protective effect against atorvastatin induced myopathy? A histopathological and immunohistochemical study in albino rats [J]. Histology and Histopathology, 2015, 30(3): 383-90.  [326] KHANDELWAL S. Obesity in midlife: lifestyle and dietary strategies [J]. Climacteric, 2020, 23(2): 140-7.  [327] KHAW K T. Epidemiological aspects of ageing [J]. Philosophical Transactions of the Royal Society B-Biological Sciences, 1997, 352(1363): 1829-35.  [328] KHOURY R, GHANTOUS Z, IBRAHIM R, et al. Anxiety, depression and post-traumatic stress disorder in patients on hemodialysis in the setting of the pandemic, inflation, and the Beirut blast: a cross-sectional study [J]. BMC Psychiatry, 2023, 23(1): 284.  [329] KHOVASOVA N O, NAUMOV A V, TKACHEVA O N. [Anemia in the elderly: influence on physical, functional status and prognosis.] [J]. Adv Gerontol, 2020, 33(3): 501-6.  [330] KICHURA A B, DUDERIJA E, VIDIC A, et al. Does a brief functional assessment in the emergency department predict outcomes of patients admitted with heart failure? The FASTER-HF study [J]. Arch Cardiovasc Dis, 2020, 113(12): 766-71.  [331] KIM B, YOUM C, PARK H, et al. Machine learning approach to classifying declines of physical function and muscle strength associated with cognitive function in older women: gait characteristics based on three speeds [J]. Front Public Health, 2024, 12: 1376736.  [332] KIM C, SRIVASTAVA S, RICE M, et al. Expression of human amyloid precursor protein in the skeletal muscles of Drosophila results in age- and activity-dependent muscle weakness [J]. BMC Physiol, 2011, 11: 7.  [333] KIM H B, KIM D, KIM H, et al. Aβ Accumulation in Vmo Contributes to Masticatory Dysfunction in 5XFAD Mice [J]. Journal of Dental Research, 2021, 100(9): 960-7.  [334] KIM J, CHOI K H, CHO S G, et al. Association of muscle and visceral adipose tissues with the probability of Alzheimer's disease in healthy subjects [J]. Sci Rep, 2019, 9(1): 949.  [335] KIM J Y, RASHEED A, YOO S J, et al. Distinct amyloid precursor protein processing machineries of the olfactory system [J]. Biochemical and Biophysical Research Communications, 2018, 495(1): 533-8.  [336] KIM M, WON C W. Sarcopenia Is Associated with Cognitive Impairment Mainly Due to Slow Gait Speed: Results from the Korean Frailty and Aging Cohort Study (KFACS) [J]. Int J Environ Res Public Health, 2019, 16(9).  [337] KIM S, KIM J O, KWON K J, et al. Associations of truncal body composition with cognitive status in patients with dementia [J]. Neurol Sci, 2021, 42(1): 209-14.  [338] KIMYAGAROV S, LEVENKRON S, SHABI A, et al. [Changes of skeletal muscle mass among disabled elderly] [J]. Harefuah, 2010, 149(2): 67-70, 126.  [339] KIRBY J, GOODALL E F, SMITH W, et al. Broad clinical phenotypes associated with TAR-DNA binding protein (TARDBP) mutations in amyotrophic lateral sclerosis [J]. Neurogenetics, 2010, 11(2): 217-25.  [340] KIRK R. Clinical trials in CNS--SMi's eighth annual conference [J]. IDrugs, 2010, 13(2): 66-9.  [341] KISLER K, NIKOLAKOPOULOU A M, SWEENEY M D, et al. Acute Ablation of Cortical Pericytes Leads to Rapid Neurovascular Uncoupling [J]. Frontiers in Cellular Neuroscience, 2020, 14.  [342] KITO Y, KAZUI H, YOSHIDA T, et al. Language and semantic memory impairment in a patient with motor neuron disease and semantic dementia - A case report [J]. Brain and Nerve, 2010, 62(6): 625-30.  [343] KLAWITTER L, MAHONEY S J, DAHL L, et al. Evaluating Additional Aspects of Muscle Function with a Digital Handgrip Dynamometer and Accelerometer for Cognitive Functioning in Older Adults: A Pilot Study [J]. J Alzheimers Dis Rep, 2020, 4(1): 495-9.  [344] KLOSE R, PRINZ A, TETZLAFF F, et al. Loss of the serine protease HTRA1 impairs smooth muscle cells maturation [J]. Scientific Reports, 2019, 9.  [345] KLOTH K, COZMA C, BESTER M, et al. Dystonia as initial presentation of compound heterozygous <i>GBA2</i> mutations: Expanding the phenotypic spectrum of SPG46 [J]. European Journal of Medical Genetics, 2020, 63(9).  [346] KOBAYASHI K, FUKUTANI Y, HAYASHI M, et al. Non-familial olivopontocerebellar atrophy combined with late onset Alzheimer's disease: a clinico-pathological case report [J]. Journal of the Neurological Sciences, 1998, 154(1): 106-12.  [347] KOBAYASHI K, MORIKAWA K, FUKUTANI Y, et al. RAMSAY HUNT SYNDROME - PROGRESSIVE MENTAL DETERIORATION IN ASSOCIATION WITH UNUSUAL CEREBRAL WHITE-MATTER CHANGE [J]. Clinical Neuropathology, 1994, 13(2): 88-96.  [348] KOCYIGIT S E, ATES BULUT E A P, AYDIN A E, et al. Improvement of nutritional status enhances cognitive and physical functions in older adults with orthostatic hypotension [J]. Nutrition, 2021, 90: 111261.  [349] KOGA S, FUJIMOTO T, HASEGAWA K, et al. Disseminated necrotizing leukoencephalopathy following intrathecal methotrexate in childhood leukemia (Japanese) [J]. FUKUOKA ACT MED, 1976, 67(1): 24-31.  [350] KONAGAYA M, KONAGAYA Y, IIDA M. [CSF acetylcholinesterase activity in central neurological diseases involving cholinergic systems] [J]. Rinsho Shinkeigaku, 1992, 32(3): 266-71.  [351] KOPPERS M, GROEN E J, VAN VUGHT P W, et al. Screening for rare variants in the coding region of ALS-associated genes at 9p21.2 and 19p13.3 [J]. Neurobiol Aging, 2013, 34(5): 1518.e5-7.  [352] KOPPERS M, VAN BLITTERSWIJK M M, VLAM L, et al. VCP mutations in familial and sporadic amyotrophic lateral sclerosis [J]. Neurobiol Aging, 2012, 33(4): 837.e7-13.  [353] KöRNER S, KOLLEWE K, ILSEMANN J, et al. Prevalence and prognostic impact of comorbidities in amyotrophic lateral sclerosis [J]. European Journal of Neurology, 2013, 20(4): 647-E52.  [354] KOVáCS G G, ERTSEY C, MAJTéNYI C, et al. Inherited prion disease with A117V mutation of the prion protein gene:: a novel Hungarian family [J]. Journal of Neurology Neurosurgery and Psychiatry, 2001, 70(6): 802-5.  [355] KRAL A M, OZEROVA N, CLOSE J, et al. Divergent kinetics differentiate the mechanism of action of two HDAC inhibitors [J]. Biochemistry, 2014, 53(4): 725-34.  [356] KRAMER A. An Overview of the Beneficial Effects of Exercise on Health and Performance [M]//XIAO J. Physical Exercise for Human Health. 2020: 3-22.  [357] KRAUSE S, GöHRINGER T, WALTER M C, et al. Brain imaging and neuropsychology in late-onset dementia due to a novel mutation (R93C) of valosin-containing protein [J]. Clinical Neuropathology, 2007, 26(5): 232-40.  [358] KRISHNAMURTHY K, TROTTI D, PASINELLI P, et al. Real-Time Fluorescent Measurement of Synaptic Functions in Models of Amyotrophic Lateral Sclerosis [J]. Jove-Journal of Visualized Experiments, 2021, (173).  [359] KRZNARIĆ Z, BENDER D V, KELECIĆ D L, et al. [Croatian guidelines for nutrition in the elderly, part II--clinical nutrition] [J]. Lijec Vjesn, 2011, 133(9-10): 299-307.  [360] KüGLER S, STRATEN G, KREPPEL F, et al. The X-linked inhibitor of apoptosis (XIAP) prevents cell death in axotomized CNS neurons in vivo [J]. Cell Death Differ, 2000, 7(9): 815-24.  [361] KüGLER S, STRATEN G, KREPPEL F, et al. The X-linked inhibitor of apoptosis (XIAP) prevents cell death in axotomized CNS neurons <i>in vivo</i> [J]. Cell Death and Differentiation, 2000, 7(9): 815-24.  [362] KUO H K, YEN C J, BEAN J F. Levels of homocysteine are inversely associated with cardiovascular fitness in women, but not in men: data from the National Health and Nutrition Examination Survey 1999-2002 [J]. Journal of Internal Medicine, 2005, 258(4): 328-35.  [363] KUPELI I, YüKSEL F. Coronavirus (COVID-19), Advanced Age and Malnutrition: A Risky Coexistence [J]. Aging Medicine and Healthcare, 2020, 11(4): 142-5.  [364] KURMAEV D P, BULGAKOVA S V, TRENEVA E V, et al. [COVID-19, neuroCOVID-19 and cognitive impairment in elderly and old patients (literature review).] [J]. Adv Gerontol, 2023, 36(1): 98-108.  [365] KUSABA T, HATTA T, KIMURA T, et al. Renal involvement in cerebral autosomal dominant arteriopathy with subcortical infarcts and leukoencephalopathy (CADASIL) [J]. Clinical Nephrology, 2007, 67(3): 182-7.  [366] KUSTERMANN M, MANTA L, PAONE C, et al. Loss of the novel Vcp (valosin containing protein) interactor Washc4 interferes with autophagy-mediated proteostasis in striated muscle and leads to myopathy <i>in vivo</i> [J]. Autophagy, 2018, 14(11): 1911-27.  [367] KUZUHARA S, KOKUBO Y. Atypical parkinsonism of Japan: Amyotrophic lateral sclerosis-parkinsonism-dementia complex of the Kii peninsula of Japan (Muro disease): An update [J]. Movement Disorders, 2005, 20: S108-S13.  [368] KWON S H, KIM M J, MA S X, et al. <i>Eucommia ulmoides</i> Oliv. Bark. protects against hydrogen peroxide-induced neuronal cell death in SH-SY5Y cells [J]. Journal of Ethnopharmacology, 2012, 142(2): 337-45.  [369] KYOTANI M, KENZAKA T, AKITA H, et al. <i>Campylobacter insulaenigrae</i> bacteremia with meningitis: a case report [J]. Bmc Infectious Diseases, 2021, 21(1).  [370] LABRIE F. DHEA, important source of sex steroids in men and even more in women [J]. Prog Brain Res, 2010, 182: 97-148.  [371] LACLAIR K D, ZHOU Q H, MICHAELSEN M, et al. Congenic expression of poly-GA but not poly-PR in mice triggers selective neuron loss and interferon responses found in <i>C9orf72</i> ALS [J]. Acta Neuropathologica, 2020, 140(2): 121-42.  [372] LAMBERTS S W J. The endocrinology of gonadal involution: menopause and andropause [J]. Annales D Endocrinologie, 2003, 64(2): 77-81.  [373] LAMBERTS S W J, VANDENBELD A W, VANDERLELY A J. The endocrinology of aging [J]. Science, 1997, 278(5337): 419-24.  [374] LAMERS S, KASIM Z, RODRíGUEZ-GARCíA W D, et al. Validation of SARC-F-Proxy for the Screening of Sarcopenia in Older Patients with Cognitive Impairment [J]. J Frailty Sarcopenia Falls, 2023, 8(4): 204-10.  [375] LAURETANI F, RUFFINI L, SCARLATTEI M, et al. Relationship between comprehensive geriatric assessment and amyloid PET in older persons with MCI [J]. Bmc Geriatrics, 2020, 20(1).  [376] LE BER I, MARTINEZ M, CAMPION D, et al. A non-DM1, non-DM2 multisystem myotonic disorder with frontotemporal dementia: phenotype and suggestive mapping of the DM3 locus to chromosome 15q21-24 [J]. Brain, 2004, 127(Pt 9): 1979-92.  [377] LE N T T, CHANG L, KOVLYAGINA I, et al. Motor neuron disease, TDP-43 pathology, and memory deficits in mice expressing ALS-FTD-linked <i>UBQLN2</i> mutations [J]. Proceedings of the National Academy of Sciences of the United States of America, 2016, 113(47): E7580-E9.  [378] LEAL A, MARQUES C. Optical Fiber-Integrated Smart Structures: Towards Transparent Devices for Healthcare 4.0 [J]. Ieee Instrumentation & Measurement Magazine, 2021, 24(5): 41-9.  [379] LEE A, RAYNER S L, DE LUCA A, et al. Casein kinase II phosphorylation of cyclin F at serine 621 regulates the Lys48-ubiquitylation E3 ligase activity of the SCF((cyclin F)) complex [J]. Open Biol, 2017, 7(10).  [380] LEE A, RAYNER S L, DE LUCA A, et al. Casein kinase II phosphorylation of cyclin F at serine 621 regulates the Lys48-ubiquitylation E3 ligase activity of the SCF<SUP>(cyclin F)</SUP> complex [J]. Open Biology, 2017, 7(10).  [381] LEE D H, CHOI Y H, CHO K H, et al. A case of rivastigmine toxicity caused by transdermal patch [J]. American Journal of Emergency Medicine, 2011, 29(6): 695.e1-.e2.  [382] LEE S Y, PARK J H, KIM S H, et al. A case of adult polyglucosan body disease [J]. Yonsei Medical Journal, 2007, 48(4): 701-3.  [383] LEE W J, PENG L N, LIANG C K, et al. Cognitive frailty predicting all-cause mortality among community-living older adults in Taiwan: A 4-year nationwide population-based cohort study [J]. PLoS One, 2018, 13(7): e0200447.  [384] LEE W J, PENG L N, LIN M H, et al. Six-year transition of physio-cognitive decline syndrome: Results from I-Lan Longitudinal Aging Study [J]. Arch Gerontol Geriatr, 2022, 102: 104743.  [385] LEMON J A, AKSENOV V, SAMIGULLINA R, et al. A multi-ingredient dietary supplement abolishes large-scale brain cell loss, improves sensory function, and prevents neuronal atrophy in aging mice [J]. Environmental and Molecular Mutagenesis, 2016, 57(5): 382-404.  [386] LI C L, CHANG H Y, TSAI Y H. Sarcopenia Screened with SARC-F and Subjective Memory Complaints Are Independently Associated with Increased Risk of Incident Dementia among Cognitively Unimpaired Older Adults [J]. J Nutr Health Aging, 2023, 27(11): 940-5.  [387] LI H Y, RUBERU K, KARL T, et al. Cerebral Apolipoprotein-D Is Hypoglycosylated Compared to Peripheral Tissues and Is Variably Expressed in Mouse and Human Brain Regions [J]. Plos One, 2016, 11(2).  [388] LIEWLUCK T, MILONE M, MAUERMANN M L, et al. A NOVEL <i>VCP</i> MUTATION UNDERLIES SCAPULOPERONEAL MUSCULAR DYSTROPHY AND DROPPED HEAD SYNDROME FEATURING LOBULATED FIBERS [J]. Muscle & Nerve, 2014, 50(2): 295-9.  [389] LIN Y C, CHUNG C P, LEE P L, et al. The Flexibility of Physio-Cognitive Decline Syndrome: A Longitudinal Cohort Study [J]. Frontiers in Public Health, 2022, 10.  [390] LIN Y S, LIN F Y, HSIAO Y H. Myostatin Is Associated With Cognitive Decline in an Animal Model of Alzheimer's Disease [J]. Mol Neurobiol, 2019, 56(3): 1984-91.  [391] LISOWIEC J, MAGNER D, KIERZEK E, et al. Structural determinants for alternative splicing regulation of the MAPT pre-mRNA [J]. RNA Biol, 2015, 12(3): 330-42.  [392] LIU G Q, YU Q T, ZHU H Z, et al. Amyloid-β mediates intestinal dysfunction and enteric neurons loss in Alzheimer's disease transgenic mouse [J]. Cellular and Molecular Life Sciences, 2023, 80(12).  [393] LIU J Y H, SUN M Y Y, SOMMERVILLE N, et al. Soy flavonoids prevent cognitive deficits induced by intra-gastrointestinal administration of beta-amyloid [J]. Food and Chemical Toxicology, 2020, 141.  [394] LIU M, WANG L, GAO J, et al. Inhibition of Calpain Protects Against Tauopathy in Transgenic P301S Tau Mice [J]. J Alzheimers Dis, 2019, 69(4): 1077-87.  [395] LIU Q Y, KOUKIEKOLO R, ZHANG D L, et al. Molecular events linking cholesterol to Alzheimer's disease and inclusion body myositis in a rabbit model [J]. Am J Neurodegener Dis, 2016, 5(1): 74-84.  [396] LIU S, LI M, ZHU J, et al. Correlation of muscle strength with cognitive function and medial temporal lobe atrophy in patients with mild to moderate Alzheimer′s disease [J]. National Medical Journal of China, 2022, 102(35): 2786-92.  [397] LIU S W, LI M, ZHU J T, et al. [Correlation of muscle strength with cognitive function and medial temporal lobe atrophy in patients with mild to moderate Alzheimer's disease] [J]. Zhonghua Yi Xue Za Zhi, 2022, 102(35): 2786-92.  [398] LIU W F. Effects of cholinesterase inhibitors on a two-component chained schedule performance in rats [J]. Neurotoxicology and Teratology, 2000, 22(3): 389-96.  [399] LIU X Y, GONZALEZ-TOLEDO M E, FAGAN A, et al. Stem cell factor and granulocyte colony-stimulating factor exhibit therapeutic effects in a mouse model of CADASIL [J]. Neurobiology of Disease, 2015, 73: 189-203.  [400] LIU X Z, HALVORSEN S, BLANKE N, et al. Progressive mechanical and structural changes in anterior cerebral arteries with Alzheimer's disease [J]. Alzheimers Research & Therapy, 2023, 15(1).  [401] LIU Y, YU J T, ZONG Y, et al. <i>C9ORF72</i> Mutations in Neurodegenerative Diseases [J]. Molecular Neurobiology, 2014, 49(1): 386-98.  [402] LLAVERO HURTADO M, FULLER H R, WONG A M S, et al. Proteomic mapping of differentially vulnerable pre-synaptic populations identifies regulators of neuronal stability in vivo [J]. Sci Rep, 2017, 7(1): 12412.  [403] LLEWELLYN K J, NALBANDIAN A, JUNG K M, et al. Lipid-enriched diet rescues lethality and slows down progression in a murine model of VCP-associated disease [J]. Human Molecular Genetics, 2014, 23(5): 1333-44.  [404] LóPEZ JIMéNEZ E, NEIRA ÁLVAREZ M, MENéNDEZ COLINO R, et al. Muscle mass loss measured with portable ultrasound in hospitalized older adults: The ECOSARC study [J]. J Nutr Health Aging, 2024, 28(1): 100010.  [405] LóPEZ-VALDéS H E, GARCíA-COLUNGA J. Nicotinic acetylcholine receptors envolvement in central nervous system disorders [J]. Salud Mental, 2003, 26(3): 66-72.  [406] LOVE M N, CLARK D G, COCHRAN J N, et al. Clinical, imaging, pathological, and biochemical characterization of a novel presenilin 1 mutation (N135Y) causing Alzheimer's disease [J]. Neurobiology of Aging, 2017, 49.  [407] LU A, THAN S, BEARE R, et al. Interactions between muscle volume and body mass index on brain structure in the UK Biobank [J]. Front Dement, 2024, 3: 1456716.  [408] LU C F, LIU W S, CANG X M, et al. The bidirectional associations between sarcopenia-related traits and cognitive performance [J]. Sci Rep, 2024, 14(1): 7591.  [409] LUDVIGSSON J F, OLSSON T, EKBOM A, et al. A population-based study of coeliac disease, neurodegenerative and neuroinflammatory diseases [J]. Aliment Pharmacol Ther, 2007, 25(11): 1317-27.  [410] LUNDY J, HAYDEN D, PYLAND S, et al. An Age-Friendly Health System [J]. J Am Geriatr Soc, 2021, 69(3): 806-12.  [411] LUO Y X, ZHU Y H, YAO X Q. Knowledge mapping of exercise and physical activity research in older adults: Hotspots, bursts, and trends of the last decade [J]. Heliyon, 2023, 9(12): e23181.  [412] LUZZI A, WANG F, LI S, et al. Skeletal muscle cell protein dysregulation highlights the pathogenesis mechanism of myopathy-associated p97/VCP R155H mutations [J]. Front Neurol, 2023, 14: 1211635.  [413] MA C, LU Q L, SHI W C, et al. Diagnosis and treatment of a dural arteriovenous fistula presenting with progressive parkinsonism and dementia: A case report and literature review [J]. Experimental and Therapeutic Medicine, 2015, 9(2): 523-6.  [414] MAAT-SCHIEMAN M L, VAN DUINEN S G, ROZEMULLER A J, et al. Association of vascular amyloid beta and cells of the mononuclear phagocyte system in hereditary cerebral hemorrhage with amyloidosis (Dutch) and Alzheimer disease [J]. J Neuropathol Exp Neurol, 1997, 56(3): 273-84.  [415] MAAT-SCHIEMAN M L C, VAN DUINEN S G, ROZEMULLER A J M, et al. Association of vascular amyloid β and cells of the mononuclear phagocyte system in hereditary cerebral hemorrhage with amyloidosis (Dutch) and Alzheimer disease [J]. Journal of Neuropathology and Experimental Neurology, 1997, 56(3): 273-84.  [416] MACHII N, KUDO A, SAITO H, et al. Walking Speed is the Sole Determinant Criterion of Sarcopenia of Mild Cognitive Impairment in Japanese Elderly Patients with Type 2 Diabetes Mellitus [J]. J Clin Med, 2020, 9(7).  [417] MACHUCA-PARRA A I, BIGGER-ALLEN A A, SANCHEZ A V, et al. Therapeutic antibody targeting of Notch3 signaling prevents mural cell loss in CADASIL [J]. Journal of Experimental Medicine, 2017, 214(8): 2271-82.  [418] MAHMOUDI E, SADAGHIYANI S, LIN P, et al. Diagnosis of Alzheimer's disease and related dementia among people with multiple sclerosis: Large cohort study, USA [J]. Multiple Sclerosis and Related Disorders, 2022, 57.  [419] MAKHOURI F R, GHASEMI J B. <i>In Silico</i> Studies in Drug Research Against Neurodegenerative Diseases [J]. Current Neuropharmacology, 2018, 16(6): 664-725.  [420] MALEKPOUR M, BRIDGHAM K, JAAP K, et al. The Effect of Sarcopenia on Outcomes in Geriatric Blunt Trauma [J]. Am Surg, 2017, 83(11): 1203-8.  [421] MANABE T, MIZUKAMI K, AKATSU H, et al. Prognostic Factors Related to Dementia with Lewy Bodies Complicated with Pneumonia: An Autopsy Study [J]. Intern Med, 2016, 55(19): 2771-6.  [422] MANI R J, DOGRA N, KATARE D P. The Connection between Chronic Liver Damage and Sporadic Alzheimer's Disease: Evidence and Insights from a Rat Model [J]. Brain Sciences, 2023, 13(10).  [423] MARKIANOS M, PANAS M, KALFAKIS N, et al. Plasma testosterone in male patients with Huntington's disease: Relations to severity of illness and dementia [J]. Annals of Neurology, 2005, 57(4): 520-5.  [424] MARTíN I S, BARATO V P, OLIVA S L, et al. Body Composition, Dietary, and Gustatory Function Assessment in People With Alzheimer's Disease [J]. American Journal of Alzheimers Disease and Other Dementias, 2018, 33(8): 508-15.  [425] MATSUBARA S, SHIMIZU T, KOMORI T, et al. Nuclear inclusions mimicking poly(A)-binding protein nuclear 1 inclusions in a case of inclusion body myopathy associated with Paget disease of bone and frontotemporal dementia with a novel mutation in the valosin-containing protein gene [J]. Neuromuscular Disorders, 2016, 26(7): 436-40.  [426] MATSUMOTO S, KUSAKA H, ITO H, et al. Sporadic amyotrophic lateral sclerosis with dementia and Cu/Zn superoxide dismutase-positive Lewy body-like inclusions [J]. Clinical Neuropathology, 1996, 15(1): 41-6.  [427] MATSUNO K, ASAOKA D, SUGANO K, et al. Rationale and design of Juntendo Sarcopenia Registration to explore the predictors and prognosis of sarcopenia and frailty in the elderly in TOKYO (JUSTICE-TOKYO) [J]. Geriatr Gerontol Int, 2024, 24(1): 168-72.  [428] MAYFIELD R D, ZHU L, SMITH T A, et al. The SMYD1 and skNAC transcription factors contribute to neurodegenerative diseases [J]. Brain, Behavior, & Immunity - Health, 2020, 9.  [429] MCGRATH R, ROBINSON-LANE S G, COOK S, et al. Handgrip Strength Is Associated with Poorer Cognitive Functioning in Aging Americans [J]. J Alzheimers Dis, 2019, 70(4): 1187-96.  [430] MCGRATH R, VINCENT B M, HACKNEY K J, et al. The Longitudinal Associations of Handgrip Strength and Cognitive Function in Aging Americans [J]. J Am Med Dir Assoc, 2020, 21(5): 634-9.e1.  [431] MEAKIN P J, HARPER A J, HAMILTON D L, et al. Reduction in BACE1 decreases body weight, protects against diet-induced obesity and enhances insulin sensitivity in mice [J]. Biochemical Journal, 2012, 441: 285-96.  [432] MEHRABAN-FAR S, ALRASSI J, PATEL R, et al. Dysphagia in the elderly population: A Videofluoroscopic study [J]. Am J Otolaryngol, 2021, 42(2): 102854.  [433] MELCHOR J P, MCVOY L, VAN NOSTRAND W E. Charge alterations of E22 enhance the pathogenic properties of the amyloid β-protein [J]. Journal of Neurochemistry, 2000, 74(5): 2209-12.  [434] MELCHOR J P, VAN NOSTRAND W E. Fibrillar amyloid β-protein mediates the pathologic accumulation of its secreted precursor in human cerebrovascular smooth muscle cells [J]. Journal of Biological Chemistry, 2000, 275(13): 9782-91.  [435] MERCER H M, NAIR A M, RIDGEL A, et al. Alterations in RNA editing in skeletal muscle following exercise training in individuals with Parkinson's disease [J]. Plos One, 2023, 18(12).  [436] MERCHANT R A, CHAN Y H, ANBARASAN D, et al. Association of Motoric Cognitive Risk Syndrome with Sarcopenia and Systemic Inflammation in Pre-Frail Older Adults [J]. Brain Sci, 2023, 13(6).  [437] MERLINI M, MEYER E P, ULMANN-SCHULER A, et al. Vascular β-amyloid and early astrocyte alterations impair cerebrovascular function and cerebral metabolism in transgenic arcAβ mice [J]. Acta Neuropathologica, 2011, 122(3): 293-311.  [438] MERLINI M, WANNER D, NITSCH R M. Tau pathology-dependent remodelling of cerebral arteries precedes Alzheimer's disease-related microvascular cerebral amyloid angiopathy [J]. Acta Neuropathologica, 2016, 131(5): 737-52.  [439] MERRILEES J, KLAPPER J, MURPHY J, et al. Cognitive and behavioral challenges in caring for patients with frontotemporal dementia and amyotrophic lateral sclerosis [J]. Amyotrophic Lateral Sclerosis, 2010, 11(3): 298-302.  [440] METHODS IN MEDICINE C A M. Retracted: Diagnostic Values of Advanced Glycation End Products and Homocysteine in Patients with Alzheimer's Disease and Sarcopenia [J]. Comput Math Methods Med, 2023, 2023: 9786485.  [441] MILLER N, FENG Z, EDENS B M, et al. Non-aggregating tau phosphorylation by cyclin-dependent kinase 5 contributes to motor neuron degeneration in spinal muscular atrophy [J]. J Neurosci, 2015, 35(15): 6038-50.  [442] MIMURA M, TOMINAGA I, KASHIMA H, et al. Presenile non-Alzheimer dementia with motor neuron disease and laminar spongiform degeneration [J]. Neuropathology, 1998, 18(1): 19-26.  [443] MINN Y K, SUK S H. Higher skeletal muscle mass may protect against ischemic stroke in community-dwelling adults without stroke and dementia: The PRESENT project [J]. BMC Geriatr, 2017, 17(1): 45.  [444] MIWA H, MORI H, SUMINO S, et al. An 80-year-old woman with four years history of muscle atrophy involving lower extremities predominantly on the right side [J]. Brain and Nerve, 1997, 49(9): 857-65.  [445] MIYAZAKI A, OKUYAMA T, MORI H, et al. Effects of Two Short-Term Aerobic Exercises on Cognitive Function in Healthy Older Adults during COVID-19 Confinement in Japan: A Pilot Randomized Controlled Trial [J]. Int J Environ Res Public Health, 2022, 19(10).  [446] MIZUSAWA H. [Overcoming neurological diseases-breakthrough for new era] [J]. Rinsho Shinkeigaku, 2013, 53(11): 893-7.  [447] MOK S S, TURNER B J, BEYREUTHER K, et al. Toxicity of substrate-bound amyloid peptides on vascular smooth muscle cells is enhanced by homocysteine [J]. European Journal of Biochemistry, 2002, 269(12): 3014-22.  [448] MOMBIELA R M, BORRAS C. The Usefulness of Radiomics Methodology for Developing Descriptive and Prognostic Image-Based Phenotyping in the Aging Population: Results From a Small Feasibility Study [J]. Frontiers in Aging, 2022, 3.  [449] MONINE M, NORRIS D, WANG Y, et al. A physiologically-based pharmacokinetic model to describe antisense oligonucleotide distribution after intrathecal administration [J]. J Pharmacokinet Pharmacodyn, 2021, 48(5): 639-54.  [450] MOON J H, MOON J H, KIM K M, et al. Sarcopenia as a Predictor of Future Cognitive Impairment in Older Adults [J]. J Nutr Health Aging, 2016, 20(5): 496-502.  [451] MOON Y, CHOI Y J, KIM J O, et al. Muscle profile and cognition in patients with Alzheimer's disease dementia [J]. Neurological Sciences, 2018, 39(11): 1861-6.  [452] MOON Y, MOON W J, KIM J O, et al. Role of Muscle Profile in Alzheimer's Disease: A 3-Year Longitudinal Study [J]. Eur Neurol, 2019, 81(5-6): 209-15.  [453] MOOSAVI M, NAGHDI N, MAGHSOUDI N, et al. Insulin protects against stress-induced impairments in water maze performance [J]. Behavioural Brain Research, 2007, 176(2): 230-6.  [454] MORELLA I, NEGRO M, DOSSENA M, et al. Gut-muscle-brain axis: Molecular mechanisms in neurodegenerative disorders and potential therapeutic efficacy of probiotic supplementation coupled with exercise [J]. Neuropharmacology, 2023, 240.  [455] MORI N, MORI M. Quest for a long life: Paradoxes and essentials of evolving longevity [J]. Acta Medica Nagasakiensia, 2011, 56(3): 73-80.  [456] MORIMOTO N, KURATA T, SATO K, et al. Frontal dysfunctions of ALS-PBP patients in relation to their bulbar symptoms and rCBF decline [J]. J Neurol Sci, 2012, 319(1-2): 96-101.  [457] MORITANI T. Electrical muscle stimulation: Application and potential role in aging society [J]. J Electromyogr Kinesiol, 2021, 61: 102598.  [458] MOUSSA C E H, FU Q H, KUMAR P, et al. Transgenic expression of β-APP in fast-twitch skeletal muscle leads to calcium dyshomeostasis and IBM-like pathology [J]. Faseb Journal, 2006, 20(12): 2165-+.  [459] MUNIR R, ZAIB S, ZIA-UR-REHMAN M, et al. Ultrasound-Assisted Synthesis of Piperidinyl-Quinoline Acylhydrazones as New Anti-Alzheimer's Agents: Assessment of Cholinesterase Inhibitory Profile, Molecular Docking Analysis, and Drug-like Properties [J]. Molecules, 2023, 28(5).  [460] MURAGE B, TAN H, MASHIMO T, et al. Spinal cord neurone loss and foot placement changes in a rat knock-in model of amyotrophic lateral sclerosis Type 8 [J]. Brain Commun, 2024, 6(3): fcae184.  [461] MURLIDHARAN G, SAKAMOTO K, RAO L, et al. CNS-restricted Transduction and CRISPR/Cas9-mediated Gene Deletion with an Engineered AAV Vector [J]. Mol Ther Nucleic Acids, 2016, 5(7): e338.  [462] NAGAO S, YOKOTA O, NANBA R, et al. Progressive supranuclear palsy presenting as primary lateral sclerosis but lacking parkinsonism, gaze palsy, aphasia, or dementia [J]. Journal of the Neurological Sciences, 2012, 323(1-2): 147-53.  [463] NAIDU S, BIBAT G, LIN D, et al. Progressive cavitating leukoencephalopathy: A novel childhood disease [J]. Annals of Neurology, 2005, 58(6): 929-38.  [464] NAIR T A, VADIVELAN R. BEHAVIORAL STUDIES OF DASATINIB AND RESVERATROL IN ROTENONE INDUCED PARKINSON'S RAT MODEL [J]. International Journal of Pharmaceutical Sciences and Research, 2019, 10(4): 2004-11.  [465] NAKAGAWA M, TOKIMURA M, KURIYAMA M, et al. [Chronic progressive external ophthalmoplegia (CPEO); mitochondrial DNA deletion, brain MRI and electrophysiological studies] [J]. Rinsho Shinkeigaku, 1991, 31(9): 981-6.  [466] NAKANE S, YOSHIOKA M, ODA N, et al. The characteristics of camptocormia in patients with Parkinson's disease: A large cross-sectional multicenter study in Japan [J]. J Neurol Sci, 2015, 358(1-2): 299-303.  [467] NAKAO-KATO M, IZUMI S I, NISHIOKA S, et al. The Relationship between Low Skeletal Muscle Mass and Subsequent Oral Intake Ability among the Aged Population [J]. Healthcare (Basel), 2023, 11(5).  [468] NALBANDIAN A, GHIMBOVSCHI S, RADOM-AIZIK S, et al. Global gene profiling of VCP-associated inclusion body myopathy [J]. Clin Transl Sci, 2012, 5(3): 226-34.  [469] NALBANDIAN A, GHIMBOVSCHI S, WANG Z, et al. Global gene expression profiling in R155H knock-in murine model of VCP disease [J]. Clin Transl Sci, 2015, 8(1): 8-16.  [470] NALBANDIAN A, LLEWELLYN K J, BADADANI M, et al. A progressive translational mouse model of human valosin-containing protein disease: the VCP(R155H/+) mouse [J]. Muscle Nerve, 2013, 47(2): 260-70.  [471] NALBANDIAN A, LLEWELLYN K J, BADADANI M, et al. A progressive translational mouse model of human valosin-containing protein disease: The <i>VCPR155H</i>/+ mouse [J]. Muscle & Nerve, 2013, 47(2): 260-70.  [472] NALBANDIAN A, LLEWELLYN K J, KITAZAWA M, et al. The Homozygote VCP<SUP>R155H/R155H</SUP> Mouse Model Exhibits Accelerated Human VCP-Associated Disease Pathology [J]. Plos One, 2012, 7(9).  [473] NALBANDIAN A, LLEWELLYN K J, NGUYEN C, et al. Targeted excision of VCP R155H mutation by Cre-LoxP technology as a promising therapeutic strategy for valosin-containing protein disease [J]. Hum Gene Ther Methods, 2015, 26(1): 13-24.  [474] NALBANDIAN A, LLEWELLYN K J, NGUYEN C, et al. Targeted Excision of VCP R155H Mutation by Cre-<i>LoxP</i> Technology as a Promising Therapeutic Strategy for Valosin-Containing Protein Disease [J]. Human Gene Therapy Methods, 2015, 26(1): 13-24.  [475] NALBANDIAN A, LLEWELLYN K J, NGUYEN C, et al. Rapamycin and chloroquine: the in vitro and in vivo effects of autophagy-modifying drugs show promising results in valosin containing protein multisystem proteinopathy [J]. PLoS One, 2015, 10(4): e0122888.  [476] NALBANDIAN A, LLEWELLYN K J, NGUYEN C, et al. Rapamycin and Chloroquine: The <i>In Vitro</i> and <i>In Vivo</i> Effects of Autophagy-Modifying Drugs Show Promising Results in Valosin Containing Protein Multisystem Proteinopathy [J]. Plos One, 2015, 10(4).  [477] NALBANDIAN A, NGUYEN C, KATHERIA V, et al. Exercise training reverses skeletal muscle atrophy in an experimental model of VCP disease [J]. PLoS One, 2013, 8(10): e76187.  [478] NAZAROVA A, PADNYA P, KHARLAMOVA A, et al. Peptidomimetics based on ammonium decasubstituted pillar 5 arenes: Influence of the alpha-amino acid residue nature on cholinesterase inhibition [J]. Bioorganic Chemistry, 2023, 141.  [479] NG N T H, TAN M P. Training Needs Among Healthcare Professionals Managing Patients with Dementia [J]. Aging Medicine and Healthcare, 2022, 13(1): 25-31.  [480] NGUYEN T T T, THE T H N, MCFARLAND P L, et al. Dementia Prevalence Among Older Hospitalized Patients in Vietnam and Dementia Understanding of Their Caregivers [J]. Aging Medicine and Healthcare, 2019, 10(4): 128-32.  [481] NIGHTINGALE J M D, WALSH N, BULLOCK M E, et al. Three simple methods of detecting malnutrition on medical wards [J]. Journal of the Royal Society of Medicine, 1996, 89(3): 144-8.  [482] NIKOLAKOPOULOU A M, ZHAO Z, MONTAGNE A, et al. Regional early and progressive loss of brain pericytes but not vascular smooth muscle cells in adult mice with disrupted platelet-derived growth factor receptor-β signaling [J]. Plos One, 2017, 12(4).  [483] NISHIKAGE S, HIROTA Y, OGAWA W. [Diagnosis and treatment of obesity disease in older adults] [J]. Nihon Ronen Igakkai Zasshi, 2023, 60(4): 317-30.  [484] NISHIOMASU K, OGAWA T, SATO K. Indicators of Improvement in Performing Activities of Daily Living Among Older Patients Undergoing Rehabilitation Following Hip Fractures [J]. J Aging Phys Act, 2023, 31(1): 75-80.  [485] NIZARI S, CARARE R O, ROMERO I A, et al. 3D Reconstruction of the Neurovascular Unit Reveals Differential Loss of Cholinergic Innervation in the Cortex and Hippocampus of the Adult Mouse Brain [J]. Frontiers in Aging Neuroscience, 2019, 11.  [486] NOMURA E, OHTA Y, SATO K, et al. A Japanese patient with a VCP mutation c.290G > A (p.G97E) presenting a rapid progressive respiratory failure [J]. Neurology and Clinical Neuroscience, 2019, 7(6): 361-4.  [487] NOMURA E, OHTA Y, SATO K, et al. A Japanese patient with a <i>VCP</i> mutation c.290G &gt; A (p.G97E) presenting a rapid progressive respiratory failure [J]. Neurology and Clinical Neuroscience, 2019, 7(6): 361-4.  [488] NOZAKI M, OTOMO A, MITSUI S, et al. SQSTM1(L341V) variant that is linked to sporadic ALS exhibits impaired association with MAP1LC3 in cultured cells [J]. eNeurologicalSci, 2021, 22: 100301.  [489] NUDELMAN I, DEUTSCH A A, REISS R. Surgical treatment of primary hyperparathyroidism in the elderly patient [J]. Isr J Med Sci, 1983, 19(2): 150-2.  [490] NYUNT M S Z, SOH C Y, GAO Q, et al. Characterisation of Physical Frailty and Associated Physical and Functional Impairments in Mild Cognitive Impairment [J]. Frontiers in Medicine, 2017, 4.  [491] O'CALLAGHAN J, MILLERY D B. Neuroinflammation disorders exacerbated by environmental stressors [J]. Metabolism-Clinical and Experimental, 2019, 100.  [492] O'CONNELL P G. AIDS [J]. Occup Ther Health Care, 1991, 7(2-4): 19-43.  [493] OBA N, FUJIMOTO Y, HIRATA K, et al. A case of Gerstmann-Straussler-Scheinker disease with severe muscular atrophy and vertical gaze palsy [J]. Clinical Neurology, 2000, 40(7): 726-31.  [494] OGAMA N, ENDO H, SATAKE S, et al. Impact of regional white matter hyperintensities on specific gait function in Alzheimer's disease and mild cognitive impairment [J]. J Cachexia Sarcopenia Muscle, 2021, 12(6): 2045-55.  [495] OGAMA N, SAKURAI T, KAWASHIMA S, et al. Association of Glucose Fluctuations with Sarcopenia in Older Adults with Type 2 Diabetes Mellitus [J]. J Clin Med, 2019, 8(3).  [496] OHNAKA K. [Aging and homeostasis. Sex hormones and aging.] [J]. Clin Calcium, 2017, 27(7): 947-54.  [497] OHTA T, SASAI H, OSUKA Y, et al. Age- and sex-specific associations between sarcopenia severity and poor cognitive function among community-dwelling older adults in Japan: The IRIDE Cohort Study [J]. Front Public Health, 2023, 11: 1148404.  [498] OKSENBERG A, RADWAN H, ARONS E, et al. Rapid eye movement (REM) sleep behavior disorder: A sleep disturbance affecting mainly older men [J]. Israel Journal of Psychiatry and Related Sciences, 2002, 39(1): 28-35.  [499] OKUDUR S K, SMITH L, TAN S G, et al. Sarcopenia, but not malnutrition, is associated with fear of falling in older patients with dementia [J]. North Clin Istanb, 2024, 11(1): 45-51.  [500] OLNEY N T, BISCHOF A, ROSEN H, et al. Measurement of spinal cord atrophy using phase sensitive inversion recovery (PSIR) imaging in motor neuron disease [J]. PLoS One, 2018, 13(11): e0208255.  [501] OLSON E J, BOEVE B F, SILBER M H. Rapid eye movement sleep behaviour disorder: demographic, clinical and laboratory findings in 93 cases [J]. Brain, 2000, 123: 331-9.  [502] ONG M L, HOLBROOK J D. Novel region discovery method for Infinium 450K DNA methylation data reveals changes associated with aging in muscle and neuronal pathways [J]. Aging Cell, 2014, 13(1): 142-55.  [503] ONN L V, TEO S P. Assessment of Cognitive Impairment in Geriatrics Outpatient Clinics-Achieving Standards of Care [J]. Aging Medicine and Healthcare, 2019, 10(3): 100-3.  [504] OREMUS M, WOLFSON C, VANDAL A C, et al. Caregiver acceptance of adverse effects and use of cholinesterase inhibitors in Alzheimer's disease [J]. Canadian Journal on Aging-Revue Canadienne Du Vieillissement, 2007, 26(3): 205-12.  [505] ORMROD D, SPENCER C. Metrifonate - A review of its use in Alzheimer's disease [J]. Cns Drugs, 2000, 13(6): 443-67.  [506] OSTROUMOVA O D, STARODUBOVA A V, DE V A, et al. Hypoglycemias in elderly patients with type 2 diabetes mellitus: Possible risks and ways to prevent them [J]. Profilakticheskaya Meditsina, 2019, 22(1): 109-17.  [507] OTSUKI M, NAKAGAWA Y, MORI F, et al. Progressive anterior operculum syndrome due to FTLD-TDP: a clinico-pathological investigation [J]. Journal of Neurology, 2010, 257(7): 1148-53.  [508] OVEISGHARAN S, YU L, AGRAWAL S, et al. Relation of Motor Impairments to Neuropathologic Changes of Limbic-Predominant Age-Related TDP-43 Encephalopathy in Older Adults [J]. Neurology, 2023, 101(15): e1542-e53.  [509] OZAWA K, TOMIYAMA T, MAAT-SCHIEMAN M L, et al. Enhanced Aβ<sub>40</sub> deposition was associated with increased Aβ<sub>42/43</sub> in cerebral vasculature with Dutch-type hereditary cerebral hemorrhage with amyloidosis (HCHWA-D) [M]//DELATORRE J C, KALARIA R, NAKAJIMA K, et al. Alzheimer's Disease: Vascular Etiology and Pathology. 2002: 149-54.  [510] PACIFICO J, REIJNIERSE E M, LIM W K, et al. The Association between Sarcopenia as a Comorbid Disease and Incidence of Institutionalisation and Mortality in Geriatric Rehabilitation Inpatients: REStORing health of acutely unwell adulTs (RESORT) [J]. Gerontology, 2022, 68(5): 498-508.  [511] PALMIO J, SANDELL S, SUOMINEN T, et al. Distinct distal myopathy phenotype caused by VCP gene mutation in a Finnish family [J]. Neuromuscul Disord, 2011, 21(8): 551-5.  [512] PALMIO J, SANDELL S, SUOMINEN T, et al. Distinct distal myopathy phenotype caused by <i>VCP</i> gene mutation in a Finnish family [J]. Neuromuscular Disorders, 2011, 21(8): 551-5.  [513] PAN J X, LEE D, SUN D, et al. Muscular Swedish mutant APP-to-Brain axis in the development of Alzheimer's disease [J]. Cell Death Dis, 2022, 13(11): 952.  [514] PARIS D, TOWNSEND K P, OBREGON D F, et al. Pro-inflammatory effect of freshly solubilized β-amyloid peptides in the brain [J]. Prostaglandins & Other Lipid Mediators, 2002, 70(1-2): 1-12.  [515] PARK L, KOIZUMI K, EL JAMAL S, et al. Age-Dependent Neurovascular Dysfunction and Damage in a Mouse Model of Cerebral Amyloid Angiopathy [J]. Stroke, 2014, 45(6): 1815-+.  [516] PASCO J A, STUART A L, SUI S X, et al. Dynapenia and Low Cognition: A Cross-Sectional Association in Postmenopausal Women [J]. J Clin Med, 2021, 10(2).  [517] PASDAR Y, DARBANDI M, REZAEIAN S, et al. Association of Obesity, Sarcopenia, and Sarcopenic Obesity With Hypertension in Adults: A Cross-Sectional Study From Ravansar, Iran During 2014-2017 [J]. Front Public Health, 2021, 9: 705055.  [518] PATT Y S, BEN-SHABAT N, FISHER L, et al. The Prevalence of Dementia among Dermatomyositis and Polymyositis Patients: A Retrospective Cohort Study [J]. Isr Med Assoc J, 2023, 25(7): 479-84.  [519] PEDRINOLLA A, ISANEJAD M, ANTOGNELLI C, et al. Randomised controlled trial combining vitamin E-functionalised chocolate with physical exercise to reduce the risk of protein-energy malnutrition in predementia aged people: study protocol for Choko-Age [J]. BMJ Open, 2023, 13(12): e072291.  [520] PEKER N, SHARMA M, KAMBADUR R. <i>Parkin</i> deficiency exacerbates fasting-induced skeletal muscle wasting in mice [J]. Npj Parkinsons Disease, 2022, 8(1).  [521] PENACHO LáZARO M, CALLEJA FERNáNDEZ A, CASTRO PENACHO S, et al. [Assessment of the risk of undernutrition in institutionalized patients under the degree of dependence] [J]. Nutr Hosp, 2019, 36(2): 296-302.  [522] PENG L N, CHENG Y C, YU P C, et al. Oral Nutritional Supplement with β-hydroxy-β-methylbutyrate (HMB) Improves Nutrition, Physical Performance and Ameliorates Intramuscular Adiposity in Pre-Frail Older Adults: A Randomized Controlled Trial [J]. J Nutr Health Aging, 2021, 25(6): 767-73.  [523] PENG T C, CHIOU J M, CHEN Y C, et al. Handgrip strength asymmetry and cognitive impairment risk: Insights from a seven-year prospective cohort study [J]. J Nutr Health Aging, 2024, 28(1): 100004.  [524] PEREIRA P E, SCHUERMANS N, MEYLEMANS A, et al. C-terminal frameshift variant of TDP-43 with pronounced aggregation-propensity causes rimmed vacuole myopathy but not ALS/FTD [J]. Acta Neuropathologica, 2023, 145(6): 793-814.  [525] PERRETTI A, GROSSI D, FRAGASSI N, et al. Evaluation of the motor cortex by magnetic stimulation in patients with Alzheimer disease [J]. Journal of the Neurological Sciences, 1996, 135(1): 31-7.  [526] PETERSON M D, CASTEN K, COLLINS S, et al. Muscle weakness is a prognostic indicator of disability and chronic disease multimorbidity [J]. Exp Gerontol, 2021, 152: 111462.  [527] PHADKE M, LOKESHWAR M R, BHUTADA S, et al. Kearns sayre syndrome - Case report with review of literature [J]. Indian Journal of Pediatrics, 2012, 79(5): 650-4.  [528] PHADKE M, LOKESHWAR M R, BHUTADA S, et al. RETRACTED: Kearns Sayre Syndrome-Case Report with Review of Literature (Retracted article. See vol. 80, pg. 982, 2013) [J]. Indian Journal of Pediatrics, 2012, 79(5): 650-4.  [529] PIA S, LUI F. Melas Syndrome [M]. StatPearls. Treasure Island (FL) ineligible companies. Disclosure: Forshing Lui declares no relevant financial relationships with ineligible companies.; StatPearls Publishing Copyright © 2024, StatPearls Publishing LLC. 2024.  [530] PICHER-MARTEL V, RENAUD L, BAREIL C, et al. Neuronal Expression of UBQLN2<SUP>P497H</SUP> Exacerbates TDP-43 Pathology in TDP-43<SUP>G348C</SUP> Mice through Interaction with Ubiquitin [J]. Molecular Neurobiology, 2019, 56(7): 4680-96.  [531] PIZZONIA K L, SUHR J A, CLARK L A, et al. The relation of ApoE and COMT gene-gene interactions to cognitive and motor function in community-dwelling older adults: a pilot study [J]. Front Aging Neurosci, 2023, 15: 1206473.  [532] PIZZONIA K L, SUHR J A, CLARK L A, et al. The relation of ApoE and COMT gene–gene interactions to cognitive and motor function in community-dwelling older adults: a pilot study [J]. Frontiers in Aging Neuroscience, 2023, 15.  [533] PLACEK K, BENATAR M, WUU J, et al. Machine learning suggests polygenic risk for cognitive dysfunction in amyotrophic lateral sclerosis [J]. EMBO Mol Med, 2021, 13(1): e12595.  [534] PLEWA J, SURAMPALLI A, WENCEL M, et al. A cross-sectional analysis of clinical evaluation in 35 individuals with mutations of the valosin-containing protein gene [J]. Neuromuscul Disord, 2018, 28(9): 778-86.  [535] POEHLMAN E T, DVORAK R V. Energy expenditure, energy intake, and weight loss in Alzheimer disease [J]. American Journal of Clinical Nutrition, 2000, 71(2): 650S-5S.  [536] POPPE W, TENNSTEDT A. [CLINICAL AND ANATOMOPATHOLOGICAL STUDIES ON COMBINED FORMS OF PRESENILE BRAIN ATROPHY (PICK, ALZHEIMER) WITH ATROPHYING SPINAL PROCESSES] [J]. Psychiatr Neurol (Basel), 1963, 145: 322-44.  [537] PORTARO S, CACCIOLA A, NARO A, et al. A case report of recessive myotonia congenita and early onset cognitive impairment [J]. Medicine (United States), 2018, 97(22).  [538] PORTER V R, AVIDAN A Y. Clinical Overview of REM Sleep Behavior Disorder [J]. Seminars in Neurology, 2017, 37(4): 461-70.  [539] POSTHAUER M E, COLLINS N, DORNER B, et al. Nutritional strategies for frail older adults [J]. Adv Skin Wound Care, 2013, 26(3): 128-40; quiz 41-2.  [540] PRADONO J, SUDIKNO S, SUSWANTI I, et al. Incidence and Risk Factors of Subjective Memory Complaints in Women in Central Bogor City, Indonesia [J]. Aging Medicine and Healthcare, 2020, 11(3): 95-101.  [541] PRASHER V P. AGE-SPECIFIC PREVALENCE, THYROID-DYSFUNCTION AND DEPRESSIVE SYMPTOMATOLOGY IN ADULTS WITH DOWN-SYNDROME AND DEMENTIA [J]. International Journal of Geriatric Psychiatry, 1995, 10(1): 25-31.  [542] PRATHER R S, LORSON M, ROSS J W, et al. Genetically Engineered Pig Models for Human Diseases [M]//LEWIN H A, ROBERTS R M. Annual Review of Animal Biosciences, Vol 1. 2013: 203-19.  [543] PREVITI M L, ZHANG W B, VAN NOSTRAND W E. Dexamethasone diminishes the pro-inflammatory and cytotoxic effects of amyloid β-protein in cerebrovascular smooth muscle cells [J]. Journal of Neuroinflammation, 2006, 3.  [544] PRICE D L, WONG P C, BORCHELT D R, et al. Amyotrophic lateral sclerosis and Alzheimer's disease. Lessons from model systems [J]. Revue Neurologique, 1997, 153(8-9): 484-95.  [545] PRIMO V, GRAHAM M, BIGGER-ALLEN A A, et al. Blood biomarkers in a mouse model of CADASIL [J]. Brain Research, 2016, 1644: 118-26.  [546] PRIø T K, BRUUNSGAARD H, RøGE B, et al. Asymptomatic bacteriuria in elderly humans is associated with increased levels of circulating TNF receptors and elevated numbers of neutrophils [J]. Exp Gerontol, 2002, 37(5): 693-9.  [547] PROBST A, GöTZ J, WIEDERHOLD K H, et al. Axonopathy and amyotrophy in mice transgenic for human four-repeat tau protein [J]. Acta Neuropathol, 2000, 99(5): 469-81.  [548] PUCCI S, GREGGI C, POLIDORO C, et al. Clusterin silencing restores myoblasts viability and down modulates the inflammatory process in osteoporotic disease [J]. J Transl Med, 2019, 17(1): 118.  [549] QUERFURTH H W, SUHARA T, ROSEN K M, et al. β-amyloid peptide expression is sufficient for myotube death:: Implications for human inclusion body myopathy [J]. Molecular and Cellular Neuroscience, 2001, 17(5): 793-810.  [550] QUILLIOT D, BöHME P, MALGRAS A, et al. Obesity in the elderly [J]. Nutrition Clinique et Metabolisme, 2013, 27(2): 95-101.  [551] RADAVELLI-BAGATINI S, MACPHERSON H, SCOTT D, et al. Impaired muscle function, including its decline, is related to greater long-term late-life dementia risk in older women [J]. Journal of Cachexia Sarcopenia and Muscle, 2023, 14(3): 1508-19.  [552] RADZIWONIK W, ELERT-DOBKOWSKA E, TOMCZUK F, et al. C9orf72 hexanucleotide repeat expansion found in suspected spinobulbar muscular atrophy (SBMA) [J]. Neurol Neurochir Pol, 2022, 56(3): 276-80.  [553] RADZIWONIK W, ELERT-DOBKOWSKA E, TOMCZUK F, et al. <i>C9orf72</i> hexanucleotide repeat expansion found in suspected spinobulbar muscular atrophy (SBMA) [J]. Neurologia I Neurochirurgia Polska, 2022, 56(3): 276-80.  [554] RAHAYEL S, TREMBLAY C, VO A, et al. Mitochondrial function-associated genes underlie cortical atrophy in prodromal synucleinopathies [J]. Brain, 2023, 146(8): 3301-18.  [555] RAHMATI M, JONEYDI M S, KOYANAGI A, et al. Resistance training restores skeletal muscle atrophy and satellite cell content in an animal model of Alzheimer's disease [J]. Scientific Reports, 2023, 13(1).  [556] RAMíREZ RAMíREZ J U, CADENA SANABRIA M O, OCHOA M E. [Edmonton Frail Scale in Colombian older people. Comparison with the Fried criteria] [J]. Rev Esp Geriatr Gerontol, 2017, 52(6): 322-5.  [557] RAMíREZ-VéLEZ R, IZQUIERDO M, GARCíA-HERMOSO A, et al. Sit to stand muscle power reference values and their association with adverse events in Colombian older adults [J]. Sci Rep, 2022, 12(1): 11820.  [558] RAMROOP H, CRUZ R. Electrodiagnostic Evaluation of Motor Neuron Disease [M]. StatPearls. Treasure Island (FL) ineligible companies. Disclosure: Ricardo Cruz declares no relevant financial relationships with ineligible companies.; StatPearls Publishing Copyright © 2024, StatPearls Publishing LLC. 2024.  [559] RATNI H. Contribution to the Discovery of a Novel Medicine for a Neuromuscular Disease and of other Promising Molecules for the Treatment of Neurodevelopmental and Neurodegenerative Diseases [J]. Chimia (Aarau), 2021, 75(7-8): 614-9.  [560] RAVITS J, APPEL S, BALOH R H, et al. Deciphering amyotrophic lateral sclerosis: what phenotype, neuropathology and genetics are telling us about pathogenesis [J]. Amyotroph Lateral Scler Frontotemporal Degener, 2013, 14 Suppl 1(0 1): 5-18.  [561] RICHARD P, FENG S, TSAI Y L, et al. SETX (senataxin), the helicase mutated in AOA2 and ALS4, functions in autophagy regulation [J]. Autophagy, 2021, 17(8): 1889-906.  [562] RIEMSLAGH F W, VAN DER TOORN E C, VERHAGEN R F M, et al. Inducible expression of human C9ORF72 36x G(4)C(2) hexanucleotide repeats is sufficient to cause RAN translation and rapid muscular atrophy in mice [J]. Dis Model Mech, 2021, 14(2).  [563] RIEMSLAGH F W, VAN DER TOORN E C, VERHAGEN R F M, et al. Inducible expression of human C9ORF72 36× G4C2 hexanucleotide repeats is sufficient to cause RAN translation and rapid muscular atrophy in mice [J]. DMM Disease Models and Mechanisms, 2021, 14(2).  [564] RIZWANI W, FASIM A, SHARMA D, et al. S137 phosphorylation of profilin 1 is an important signaling event in breast cancer progression [J]. PLoS One, 2014, 9(8): e103868.  [565] ROGERS S D, JARROTT S E. Cognitive impairment and effects on upper body strength of adults with dementia [J]. Journal of Aging and Physical Activity, 2008, 16(1): 61-8.  [566] ROHER A E, KUO Y M, POTTER P E, et al. Cortical cholinergic denervation elicits vascular Aβ deposition [M]//KALARIA R N, INCE P. Vascular Factors in Alzheimer's Disease. 2000: 366-73.  [567] ROLLAND Y, BARRETO P S, MALTAIS M, et al. Effect of Long-Term Omega 3 Polyunsaturated Fatty Acid Supplementation with or without Multidomain Lifestyle Intervention on Muscle Strength in Older Adults: Secondary Analysis of the Multidomain Alzheimer Preventive Trial (MAPT) [J]. Nutrients, 2019, 11(8).  [568] ROSANO C, NEWMAN A, SANTANASTO A, et al. Increase in skeletal muscular adiposity and cognitive decline in a biracial cohort of older men and women [J]. Journal of the American Geriatrics Society, 2023, 71(9): 2759-68.  [569] ROSENDAHL-RIISE H, DIERKES J, ÅDNANES S, et al. Weight changes and mobility in the early phase after hip fracture in community-dwelling older persons [J]. Eur Geriatr Med, 2020, 11(4): 545-53.  [570] RUCHOUX M M, BRULIN P, BRILLAULT J, et al. Lessons from CADASIL [M]//DELATORRE J C, KALARIA R, NAKAJIMA K, et al. Alzheimer's Disease: Vascular Etiology and Pathology. 2002: 224-31.  [571] RUSMINI P, CRISTOFANI R, TEDESCO B, et al. Enhanced Clearance of Neurotoxic Misfolded Proteins by the Natural Compound Berberine and Its Derivatives [J]. Int J Mol Sci, 2020, 21(10).  [572] RYAN É B, YAN J, MILLER N, et al. Early death of ALS-linked CHCHD10-R15L transgenic mice with central nervous system, skeletal muscle, and cardiac pathology [J]. iScience, 2021, 24(2): 102061.  [573] SAGARE A P, SWEENEY M D, MAKSHANOFF J, et al. Shedding of soluble platelet-derived growth factor receptor-β from human brain pericytes [J]. Neuroscience Letters, 2015, 607: 97-101.  [574] SAGI O, WOLFSON M, UTKO N, et al. p66<SUP>ShcA</SUP> and ageing:: modulation by longevity-promoting agent aurintricarboxylic acid [J]. Mechanisms of Ageing and Development, 2005, 126(2): 249-54.  [575] SAIKRISHNA K, KUMARI R, CHAITANYA K, et al. Combined Administration of Monosodium Glutamate and High Sucrose Diet Accelerates the Induction of Type 2 Diabetes, Vascular Dysfunction, and Memory Impairment in Rats [J]. Journal of Environmental Pathology Toxicology and Oncology, 2018, 37(1): 63-80.  [576] SAJI N, ARAI H, SAKURAI T, et al. [Frailty and sarcopenia: a new bridge to dementia] [J]. Nihon Rinsho, 2016, 74(3): 505-9.  [577] SAKA B, BEKTAS M, BAKKALOGLU O K, et al. Malnutrition treatment and follow-up in clinical nutrition outpatient clinics associated with increased muscle mass [J]. Nutrition, 2022, 101: 111680.  [578] SáNCHEZ-CASTELLANO C, MARTíN-ARAGóN S, VAQUERO-PINTO N, et al. [Prevalence of sarcopenia and characteristics of sarcopenic subjects in patients over 80 years with hip fracture] [J]. Nutr Hosp, 2019, 36(4): 813-8.  [579] SANDBERG C, JOHANSSON K, CHRISTERSSON C, et al. Low bone mineral density in adults with complex congenital heart disease [J]. Int J Cardiol, 2020, 319: 62-6.  [580] SANDELL L L, BAAR T M, ZELL A M, et al. Virtual Visit Trends in Nursing Homes During the COVID-19 Pandemic [J]. Telemed J E Health, 2024, 30(3): 743-7.  [581] SANFORD A M, MORLEY J E, BERG-WEGER M, et al. High prevalence of geriatric syndromes in older adults [J]. PLoS One, 2020, 15(6): e0233857.  [582] SANTANA-SOSA E, BARRIOPEDRO M I, LOPEZ-MOJARES L M, et al. Exercise Training is Beneficial for Alzheimer's Patients [J]. International Journal of Sports Medicine, 2008, 29(10): 845-50.  [583] SANTOS R, NASCIMENTO S B D, MENDES T D R, et al. Wasting syndrome and associated factors in hospitalized older people [J]. Experimental Gerontology, 2022, 170.  [584] SASAKI S. Phenotypes in ALS - Clinical features and pathology [J]. Brain and Nerve, 2007, 59(10): 1013-21.  [585] SCHATTEN H, CHAKRABARTI A, HEDRICK J. Centrosome and microtubule instability in aging Drosophila cells [J]. J Cell Biochem, 1999, 74(2): 229-41.  [586] SCHATTEN H, CHAKRABARTI A, HEDRICK J. Centrosome and microtubule instability in aging <i>Drosophila</i> cells [J]. Journal of Cellular Biochemistry, 1999, 74(2): 229-41.  [587] SCHENCK C H. REM sleep behavior disorder: relevance to epileptologists [J]. Zeitschrift Fur Epileptologie, 2019, 32(1): 6-11.  [588] SCHIAVA M, IKENAGA C, VILLAR-QUILES R N, et al. Genotype-phenotype correlations in valosin-containing protein disease: a retrospective muticentre study [J]. J Neurol Neurosurg Psychiatry, 2022.  [589] SCHIMKE N, KRAMPFL K, PETRI S, et al. Cerebral symptoms with motor neuronal disorders: A special form of ALS-plus syndrome [J]. Nervenarzt, 2002, 73(8): 751-3.  [590] SCUDIERO D A, POLINSKY R J, BRUMBACK R A, et al. Alzheimer disease fibroblasts are hypersensitive to the lethal effects of a DNA-damaging chemical [J]. Mutat Res, 1986, 159(1-2): 125-31.  [591] SEGEV A, ITELMAN E, AVAKY C, et al. Low ALT Levels Associated with Poor Outcomes in 8700 Hospitalized Heart Failure Patients [J]. J Clin Med, 2020, 9(10).  [592] SEILER W O, ITIN P, STäHELIN H B. Zinc deficiency, a problem of old age frequently not recognized [J]. Ernahrungs-Umschau, 2002, 49(7): 260-+.  [593] SELCUK N A, FENERCIOGLU A. Reduction of glucose metabolism in basal ganglia diagnosed with FDG-PET scan: A neuroacanthocytosis case [J]. Clinical Nuclear Medicine, 2010, 35(7): 557-8.  [594] SHAMMAS M K, NIE Y, GILSRUD A, et al. CHCHD10 mutations induce tissue-specific mitochondrial DNA deletions with a distinct signature [J]. Hum Mol Genet, 2023, 33(1): 91-101.  [595] SHAW M P, HIGGINBOTTOM A, MCGOWN A, et al. Stable transgenic C9orf72 zebrafish model key aspects of the ALS/FTD phenotype and reveal novel pathological features [J]. Acta Neuropathologica Communications, 2018, 6.  [596] SHIMADA H, DOI T, LEE S, et al. Cognitive Frailty Predicts Incident Dementia among Community-Dwelling Older People [J]. J Clin Med, 2018, 7(9).  [597] SHIN Y, CHO H S, REBECK G W, et al. Vascular changes in Iowa-type hereditary cerebral amyloid angiopathy [M]//DELATORRE J C, KALARIA R, NAKAJIMA K, et al. Alzheimer's Disease: Vascular Etiology and Pathology. 2002: 245-51.  [598] SHIUE I. Chronic diseases and life events accounted for 2-18 % population attributable risks for adult hearing loss: UK Adult Psychiatric Morbidity Survey, 2007 [J]. European Archives of Oto-Rhino-Laryngology, 2016, 273(1): 93-103.  [599] SIMPSON I. Therapeutic delivery: industry update covering November 2019 [J]. Ther Deliv, 2020, 11(4): 217-24.  [600] SINGH N N, SEO J, RAHN S J, et al. A multi-exon-skipping detection assay reveals surprising diversity of splice isoforms of spinal muscular atrophy genes [J]. PLoS One, 2012, 7(11): e49595.  [601] SINGHAL S, BANSAL R, DEWANGAN G C, et al. Low one-repetition-maximum knee extension is significantly associated with poor grip strength, female sex, and various aging-related syndromes [J]. Aging Med (Milton), 2020, 3(2): 125-31.  [602] SKOOG I. A review on blood pressure and ischaemic white matter lesions [J]. Dementia and Geriatric Cognitive Disorders, 1998, 9: 13-9.  [603] SKRE H. Hereditary spastic paraplegia in Western Norway [J]. Clinical Genetics, 1974, 6(3): 165-83.  [604] SOLANKI S, VELUGOTI L. Delayed Presentation of Antipsychotic Withdrawal Tardive Dyskinesia: A Case Report [J]. Cureus Journal of Medical Science, 2023, 15(8).  [605] SOLé M, UNZETA M. Vascular cell lines expressing SSAO/VAP-1: a new experimental tool to study its involvement in vascular diseases [J]. Biology of the Cell, 2011, 103(11): 543-57.  [606] SOMEYA Y, TAMURA Y, KAGA H, et al. Sarcopenic obesity is associated with cognitive impairment in community-dwelling older adults: The Bunkyo Health Study [J]. Clin Nutr, 2022, 41(5): 1046-51.  [607] SONE J, MORI K, INAGAKI T, et al. Clinicopathological features of adult-onset neuronal intranuclear inclusion disease [J]. Brain, 2016, 139(Pt 12): 3170-86.  [608] SONE J, SOBUE G. [Neuronal Intranuclear Inclusion Disease] [J]. Brain Nerve, 2017, 69(1): 5-16.  [609] SOONTRAPA P, SEVEN N A, LIEWLUCK T, et al. Adolescent-onset multisystem proteinopathy due to a novel VCP variant [J]. Neuromuscular Disorders, 2024, 34: 89-94.  [610] SORBERA L A, BOLóS J, SERRADELL N. Ibutamoren mesilate -: Growth hormone secretagogue [J]. Drugs of the Future, 2006, 31(5): 390-9.  [611] SOYSAL P, HEYBELI C, KOC OKUDUR S, et al. Prevalence and co-incidence of geriatric syndromes according to glomerular filtration rate in older patients [J]. Int Urol Nephrol, 2023, 55(2): 469-76.  [612] SOYSAL P, SMITH L. The prevalence and co-existence of geriatric syndromes in older patients with dementia compared to those without dementia [J]. Aging Clin Exp Res, 2024, 36(1): 66.  [613] SPERLICH E, FLEINER T, ZIJLSTRA W, et al. Sarcopenia in geriatric psychiatry: feasibility of the diagnostic process and estimation of prevalence within a hospital context [J]. J Cachexia Sarcopenia Muscle, 2021, 12(5): 1153-60.  [614] SPINA S, VAN LAAR A D, MURRELL J R, et al. Phenotypic variability in three families with valosin-containing protein mutation [J]. Eur J Neurol, 2013, 20(2): 251-8.  [615] SPINA S, VAN LAAR A D, MURRELL J R, et al. Phenotypic variability in three families with <i>valosin</i>-<i>containing protein</i> mutation [J]. European Journal of Neurology, 2013, 20(2): 251-+.  [616] SRIVASTAVA V, ZELMANOVICH V, SHUKLA V, et al. Distinct designer diamines promote mitophagy, and thereby enhance healthspan in C. elegans and protect human cells against oxidative damage [J]. Autophagy, 2023, 19(2): 474-504.  [617] SRIVASTAVA V, ZELMANOVICH V, SHUKLA V, et al. Distinct designer diamines promote mitophagy, and thereby enhance healthspan in <i>C. elegans</i> and protect human cells against oxidative damage [J]. Autophagy, 2023, 19(2): 474-504.  [618] STARR A, SATTLER R. Synaptic dysfunction and altered excitability in C90RF72 ALS/FTD [J]. Brain Research, 2018, 1693: 98-108.  [619] STEEN B. Maximizing outcome of dementia treatment: the role of nutrition [J]. Arch Gerontol Geriatr Suppl, 2004, (9): 413-7.  [620] ȘTEFĂNESCU C, DAVIDSON M. Should dietary restrictions be imposed on Alzheimer's Disease patients affected by type 2 diabetes? [J]. Dialogues Clin Neurosci, 2024, 26(1): 53-5.  [621] STEFANOVA N A, MAKSIMOVA K Y, RUDNITSKAYA E A, et al. Association of cerebrovascular dysfunction with the development of Alzheimer's disease-like pathology in OXYS rats [J]. Bmc Genomics, 2018, 19.  [622] STERKE S, NASCIMENTO DA CUNHA A P, OOMEN H, et al. Physiotherapy in nursing homes. A qualitative study of physiotherapists' views and experiences [J]. BMC Geriatr, 2021, 21(1): 150.  [623] STORBECK M, HUPPERICH K, GASPAR J A, et al. Neuronal-Specific Deficiency of the Splicing Factor <i>Tra2b</i> Causes Apoptosis in Neurogenic Areas of the Developing Mouse Brain [J]. Plos One, 2014, 9(2).  [624] STORBECK M, HUPPERICH K, GASPAR J A, et al. Neuronal-specific deficiency of the splicing factor Tra2b causes apoptosis in neurogenic areas of the developing mouse brain [J]. PLoS One, 2014, 9(2): e89020.  [625] SUBRA J, GILLETTE-GUYONNET S, CESARI M, et al. The integration of frailty into clinical practice: Preliminary results from the Gerontopole [J]. Journal of Nutrition Health & Aging, 2012, 16(8): 714-20.  [626] SUN J, YUAN W, CHEN M, et al. Malnutrition and its risk factors in a home for seniors in Shanghai [J]. Asia Pac J Clin Nutr, 2023, 32(1): 63-9.  [627] SUN M, LU Z, CHEN W M, et al. Sarcopenia and diabetes-induced dementia risk [J]. Brain Commun, 2024, 6(1): fcad347.  [628] SURESH N T, E R V, KRISHNAKUMAR U. Topology Driven Analysis of Protein - Protein Interactome for Prioritizing Key Comorbid Genes via Sub Graph Based Average Path Length Centrality [J]. IEEE/ACM Trans Comput Biol Bioinform, 2023, 20(1): 742-51.  [629] SURIASTINI N W, TURANA Y, SUPRAPTILAH B, et al. Prevalence and Risk Factors of Dementia and Caregiver's Knowledge of the Early Symptoms of Alzheimer's Disease [J]. Aging Medicine and Healthcare, 2020, 11(2): 60-6.  [630] SUTTON J P, PULST S M. Atypical parkinsonism in a family of Portuguese ancestry: Absence of CAG repeat expansion in the MJD1 gene [J]. Neurology, 1997, 48(5): 1285-90.  [631] SZPAK G M, LEWANDOWSKA E, WIERZBA-BOBROWICZ T, et al. Small cerebral vessel disease in familial amyloid and non-amyloid angiopathies: FAD-PS-1 (P117L) mutation and CADASIL. Immunohistochemical and ultrastructural studies [J]. Folia Neuropathologica, 2007, 45(4): 192-204.  [632] TAFAKHORI A, NG A Y J, TOHARI S, et al. Mutation in <i>TWINKLE</i> in a Large Iranian Family with Progressive External Ophthalmoplegia, Myopathy, Dysphagia and Dysphonia, and Behavior Change [J]. Archives of Iranian Medicine, 2016, 19(2): 87-91.  [633] TAFAKHORI A, YU JIN NG A, TOHARI S, et al. Mutation in TWINKLE in a Large Iranian Family with Progressive External Ophthalmoplegia, Myopathy, Dysphagia and Dysphonia, and Behavior Change [J]. Arch Iran Med, 2016, 19(2): 87-91.  [634] TAKANE K, HASEGAWA Y, LIN B, et al. Detrimental Effects of Centrally Administered Angiotensin II are Enhanced in a Mouse Model of Alzheimer Disease Independently of Blood Pressure [J]. J Am Heart Assoc, 2017, 6(4).  [635] TAKATA M, TANAKA H, KIMURA M, et al. Fasudil, a rho kinase inhibitor, limits motor neuron loss in experimental models of amyotrophic lateral sclerosis [J]. Br J Pharmacol, 2013, 170(2): 341-51.  [636] TAMURA Y, ISHIKAWA J, FUJIWARA Y, et al. Prevalence of frailty, cognitive impairment, and sarcopenia in outpatients with cardiometabolic disease in a frailty clinic [J]. BMC Geriatr, 2018, 18(1): 264.  [637] TAN A, SALGADO M, FAHN S. Rapid eye movement sleep behavior disorder preceding Parkinson's disease with therapeutic response to levodopa [J]. Movement Disorders, 1996, 11(2): 214-6.  [638] TAN N, KAKULAS B A, MASTERS C L, et al. Observations on the clinical presentations and the neuropathological findings of amyotrophic lateral sclerosis in Australia and Guam [J]. Ann Acad Med Singap, 1986, 15(1): 62-6.  [639] TAN S Y, TEO S P. Purple Urine Bag Syndrome [J]. Aging Medicine and Healthcare, 2019, 10(4): 146-7.  [640] TANAKA M, NAGAI K, KOSHIBA H, et al. [Sarcopenia and its relationship with falling among outpatients attending a geriatric and memory clinic at Kyorin University Hospital] [J]. Nihon Ronen Igakkai Zasshi, 2017, 54(1): 63-74.  [641] TANAKA T, WAKABAYASHI T, OIZUMI H, et al. CLAC-P/Collagen Type XXV Is Required for the Intramuscular Innervation of Motoneurons during Neuromuscular Development [J]. Journal of Neuroscience, 2014, 34(4): 1370-9.  [642] TANIS J E, MA Z M, KRAJACIC P, et al. CLHM-1 is a Functionally Conserved and Conditionally Toxic Ca<SUP>2+</SUP>-Permeable Ion Channel in <i>Caenorhabditis elegans</i> [J]. Journal of Neuroscience, 2013, 33(30): 12275-86.  [643] TAVAKOLIAN S, GOUDARZI H, ESLAMI G, et al. Detection of Enterovirus, Herpes Simplex, Varicella Zoster, Epstein-Barr and Cytomegalovirus in cerebrospinal fluid in meningitis patients in Iran [J]. J Clin Lab Anal, 2021, 35(7): e23836.  [644] TAVAKOLIAN S, GOUDARZI H, ESLAMI G, et al. Detection of <i>Enterovirus</i>, <i>Herpes Simplex</i>, <i>Varicella Zoster</i>, <i>Epstein-Barr</i> and <i>Cytomegalovirus</i> in cerebrospinal fluid in meningitis patients in Iran [J]. Journal of Clinical Laboratory Analysis, 2021, 35(7).  [645] TAY C L, ISHAK N H, ALI M F, et al. A malnourished post-stroke man with multimorbidity and sarcopenia risk in a long-term stroke clinic: A case report [J]. Malaysian Family Physician, 2023, 18.  [646] TAYLOR C L, ALBANESE E, STEWART R. The Association of Dementia With Upper Arm and Waist Circumference in Seven Low- and Middle-Income Countries: The 10/66 Cross-Sectional Surveys [J]. Journals of Gerontology Series a-Biological Sciences and Medical Sciences, 2012, 67(8): 897-904.  [647] TELENIUS E W, ENGEDAL K, BERGLAND A. Effect of a High-Intensity Exercise Program on Physical Function and Mental Health in Nursing Home Residents with Dementia: An Assessor Blinded Randomized Controlled Trial [J]. Plos One, 2015, 10(5).  [648] TESSEUR I, VAN DORPE J, SPITTAELS K, et al. Expression of human apolipoprotein E4 in neurons causes hyperphosphorylation of protein tau in the brains of transgenic mice [J]. American Journal of Pathology, 2000, 156(3): 951-64.  [649] THAU N, KNIPPENBERG S, KöRNER S, et al. Decreased mRNA Expression of PGC-1α and PGC-1α-Regulated Factors in the SOD1<SUP>G93A</SUP> ALS Mouse Model and in Human Sporadic ALS [J]. Journal of Neuropathology and Experimental Neurology, 2012, 71(12): 1064-74.  [650] THOMAS V S, HAGEMAN P A. A preliminary study on the reliability of physical performance measures in older day-care center clients with dementia [J]. International Psychogeriatrics, 2002, 14(1): 17-23.  [651] THOMAS V S, HAGEMAN P A. Can neuromuscular strength and function in people with dementia be rehabilitated using resistance-exercise training? Results from a preliminary intervention study [J]. Journals of Gerontology Series a-Biological Sciences and Medical Sciences, 2003, 58(8): 746-51.  [652] TIAN J, SHI J, SMALLMAN R, et al. Relationships in Alzheimer's disease between the extent of Aβ deposition in cerebral blood vessel walls, as cerebral amyloid angiopathy, and the amount of cerebrovascular smooth muscle cells and collagen [J]. Neuropathology and Applied Neurobiology, 2006, 32(3): 332-40.  [653] TIAN Y, ZHOU L, GAO J, et al. Clinical features of NOTCH2NLC-related neuronal intranuclear inclusion disease [J]. J Neurol Neurosurg Psychiatry, 2022, 93(12): 1289-98.  [654] TIAN Y, ZHOU L, GAO J, et al. Clinical features of <i>NOTCH2NLC</i>-related neuronal intranuclear inclusion disease [J]. Journal of Neurology Neurosurgery and Psychiatry, 2022, 93(12): 1289-98.  [655] TIKKA S, BAUMANN M, SIITONEN M, et al. CADASIL and CARASIL [J]. Brain Pathology, 2014, 24(5): 525-44.  [656] TIMMINS P. Industry update: the latest developments in the field of therapeutic delivery, July 2023 [J]. Therapeutic Delivery, 2023, 14(9): 527-41.  [657] TIPTON P W, DEUTSCHLAENDER A B, SAVICA R, et al. Differences in Motor Features of <i>C9orf72</i>, <i>MAPT</i>, or <i>GRN</i> Variant Carriers With Familial Frontotemporal Lobar Degeneration [J]. Neurology, 2022, 99(11): E1154-E67.  [658] TOLEA M I, GALVIN J E. Sarcopenia and impairment in cognitive and physical performance [J]. Clin Interv Aging, 2015, 10: 663-71.  [659] TOMASI G, EDISON P, BERTOLDO A, et al. Novel reference region model reveals increased microglial and reduced vascular binding of <SUP>11</SUP>C(<i>R</i>)-PK11195 in patients with Alzheimer's disease [J]. Journal of Nuclear Medicine, 2008, 49(8): 1249-56.  [660] TOMINAGA I, HATTORI M, KAIHOU M, et al. Kuf's disease (adult type neuronal ceroid lipofuscinosis) with amyotrophy and progressive dementia [J]. Revue Neurologique, 1994, 150(6-7): 413-7.  [661] TORCINARO A, RICCI V, STRIMPAKOS G, et al. Peripheral Nerve Impairment in a Mouse Model of Alzheimer's Disease [J]. Brain Sci, 2021, 11(9).  [662] TORRES-LISTA V, LóPEZ-POUSA S, GIMéNEZ-LLORT L. Impact of Chronic Risperidone Use on Behavior and Survival of 3xTg-AD Mice Model of Alzheimer's Disease and Mice With Normal Aging [J]. Front Pharmacol, 2019, 10: 1061.  [663] TORVIK A, DIETRICHSON P, SVAAR H, et al. Myopathy with tremor and dementia: a metabolic disorder? Case report with postmortem study [J]. Journal of the Neurological Sciences, 1974, 21(2): 181-90.  [664] TOWN L, MCGLINN E, FIORENZA S, et al. The Metalloendopeptidase Gene <i>Pitrm1</i> Is Regulated by Hedgehog Signaling in the Developing Mouse Limb and Is Expressed in Muscle Progenitors [J]. Developmental Dynamics, 2009, 238(12): 3175-84.  [665] TOYOSHIMA K, ARAKI A, TAMURA Y, et al. Use of Dementia Assessment Sheet for Community-based Integrated Care System 8-items (DASC-8) for the screening of frailty and components of comprehensive geriatric assessment [J]. Geriatr Gerontol Int, 2020, 20(12): 1157-63.  [666] TOYOSHIMA Y, TAN C F, KOZAKAI T, et al. Is motor neuron disease-inclusion dementia a <i>forme fruste</i> of amyotrophic lateral sclerosis with dementia?: An autopsy case further supporting the disease concept [J]. Neuropathology, 2005, 25(3): 214-9.  [667] TRINCA V, MORRISON J, SLAUGHTER S, et al. Making the Most of Mealtimes (M3): effect of eating occasions and other covariates on energy and protein intake among Canadian older adult residents in long-term care [J]. J Hum Nutr Diet, 2020, 33(1): 3-11.  [668] TROAKES C, MAEKAWA S, WIJESEKERA L, et al. An MND/ALS phenotype associated with C9orf72 repeat expansion: Abundant p62-positive, TDP-43-negative inclusions in cerebral cortex, hippocampus and cerebellum but without associated cognitive decline [J]. Neuropathology, 2012, 32(5): 505-14.  [669] TSAO C Y, MENDELL J R, BARTHOLOMEW D. High mitochondrial DNA T8993G mutation (<90%) without typical features of Leigh's and NARP syndromes [J]. J Child Neurol, 2001, 16(7): 533-5.  [670] TSAO C Y, MENDELL J R, BARTHOLOMEW D. High mitochondrial DNA T8993G mutation (>90%) without typical features of leigh's and NARP syndromes [J]. Journal of Child Neurology, 2001, 16(7): 533-5.  [671] TSAO C Y, MENDELL J R, BARTHOLOMEW D. High mitochondrial DNA T8993G mutation (&gt;90%) without typical features of Leigh's and NARP syndromes [J]. Journal of Child Neurology, 2001, 16(7): 533-5.  [672] TSUCHIYA K, MITANI K, ARAI T, et al. Argyrophilic grain disease mimicking temporal Pick's disease: a clinical, radiological, and pathological study of an autopsy case with a clinical course of 15 years [J]. Acta Neuropathologica, 2001, 102(2): 195-9.  [673] TSUGAWA A, SHIMIZU S, HIROSE D, et al. Effects of 12-month exercise intervention on physical and cognitive functions of nursing home residents requiring long-term care: a non-randomised pilot study [J]. Psychogeriatrics, 2020, 20(4): 419-26.  [674] TSUGIHASHI Y, HIROSE M, IIDA H, et al. Validating care-needs level against self-reported measures of functioning, disability and sarcopenia among Japanese patients receiving home medical care: The Zaitaku Evaluative Initiatives and Outcome Study [J]. Geriatr Gerontol Int, 2021, 21(2): 229-37.  [675] TUNA F, ÜSTüNDAĞ A, BAŞAK CAN H, et al. Rapid Geriatric Assessment, Physical Activity, and Sleep Quality in Adults Aged more than 65 Years: A Preliminary Study [J]. J Nutr Health Aging, 2019, 23(7): 617-22.  [676] TURKSEVEN C H, BUYUKAKILLI B, BALLI E, et al. Effects of Huperzin-A on the Beta-amyloid accumulation in the brain and skeletal muscle cells of a rat model for Alzheimer's disease [J]. Life Sciences, 2017, 184: 47-57.  [677] TYLER S E B, TYLER L D K. Pathways to healing: Plants with therapeutic potential for neurodegenerative diseases [J]. IBRO Neurosci Rep, 2023, 14: 210-34.  [678] UCHIDA K, SUGIMOTO T, TANGE C, et al. Association between Reduction of Muscle Mass and Faster Declines in Global Cognition among Older People: A 4-Year Prospective Cohort Study [J]. J Nutr Health Aging, 2023, 27(11): 932-9.  [679] ULLEY J, ABDELHAFIZ A H. Frailty predicts adverse outcomes in older people with diabetes [J]. Practitioner, 2017, 261(1800): 17-20.  [680] ULUGERGER AVCI G, SUZAN V, BEKTAN KANAT B, et al. Depressive symptoms are associated with sarcopenia and malnutrition in older adults [J]. Psychogeriatrics, 2023, 23(1): 63-70.  [681] UMEGAKI H, BONFIGLIO V, KOMIYA H, et al. Association Between Sarcopenia and Quality of Life in Patients with Early Dementia and Mild Cognitive Impairment [J]. J Alzheimers Dis, 2020, 76(1): 435-42.  [682] UMEGAKI H, MAKINO T, UEMURA K, et al. The Associations among Insulin Resistance, Hyperglycemia, Physical Performance, Diabetes Mellitus, and Cognitive Function in Relatively Healthy Older Adults with Subtle Cognitive Dysfunction [J]. Front Aging Neurosci, 2017, 9: 72.  [683] URUSHITANI M. [Recent Advances in Novel Therapies for Neurological Diseases: An Overview and Future Scope] [J]. Brain Nerve, 2023, 75(5): 411-7.  [684] UYAMA E, HIRANO T, ITO K, et al. Adult Chediak-Higashi syndrome presenting as parkinsonism and dementia [J]. Acta Neurologica Scandinavica, 1994, 89(3): 175-83.  [685] VACCHIANO V, MASTRANGELO A, ZENESINI C, et al. Elevated plasma p-tau181 levels unrelated to Alzheimer's disease pathology in amyotrophic lateral sclerosis [J]. J Neurol Neurosurg Psychiatry, 2023, 94(6): 428-35.  [686] VAES B, PASQUET A, WALLEMACQ P, et al. The BELFRAIL (BFC80+) study: a population-based prospective cohort study of the very elderly in Belgium [J]. BMC Geriatr, 2010, 10: 39.  [687] VAES B, PASQUET A, WALLEMACQ P, et al. The BELFRAIL (BF<sub>C80+</sub>) study: a population-based prospective cohort study of the very elderly in Belgium [J]. Bmc Geriatrics, 2010, 10.  [688] VAN DEN BELD A W, DE JONG F H, GROBBEE D E, et al. Measures of bioavailable serum testosterone and estradiol and their relationships with muscle strength, bone density, and body composition in elderly men [J]. Journal of Clinical Endocrinology & Metabolism, 2000, 85(9): 3276-82.  [689] VAN DORPE J, SMEIJERS L, DEWACHTER I, et al. Prominent cerebral amyloid angiopathy in transgenic mice overexpressing the London mutant of human APP in neurons [J]. American Journal of Pathology, 2000, 157(4): 1283-98.  [690] VAN GINNEKEN C, SCHäFER K H, VAN DAM D, et al. Morphological changes in the enteric nervous system of aging and APP23 transgenic mice [J]. Brain Research, 2011, 1378: 43-53.  [691] VAN HELMOND Z K, MINERS J S, BEDNALL E, et al. Caveolin-1 and-2 and their relationship to cerebral amyloid angiopathy in Alzheimer's disease [J]. Neuropathology and Applied Neurobiology, 2007, 33(3): 317-27.  [692] VAN HUMMEL A, SABALE M, PRZYBYLA M, et al. TDP-43 pathology and functional deficits in wild-type and ALS/FTD mutant cyclin F mouse models [J]. Neuropathol Appl Neurobiol, 2023, 49(2): e12902.  [693] VAN NOSTRAND W E, MELCHOR J, WAGNER M, et al. Cerebrovascular smooth muscle cell surface fibrillar Aβ -: Alteration of the proteolytic environment in the cerebral vessel wall [M]//KALARIA R N, INCE P. Vascular Factors in Alzheimer's Disease. 2000: 89-96.  [694] VAN RHEENEN W, VAN BLITTERSWIJK M, HUISMAN M H, et al. Hexanucleotide repeat expansions in C9ORF72 in the spectrum of motor neuron diseases [J]. Neurology, 2012, 79(9): 878-82.  [695] VAN RHEENEN W, VAN BLITTERSWIJK M, HUISMAN M H B, et al. Hexanucleotide repeat expansions in <i>C9ORF72</i> in the spectrum of motor neuron diseases [J]. Neurology, 2012, 79(9): 878-82.  [696] VAREA O, GUINOVART J J, DURAN J. Malin restoration as proof of concept for gene therapy for Lafora disease [J]. Brain Communications, 2022, 4(4).  [697] VáZQUEZ-COSTA J F, CARRATALà-BOSCà S, TEMBL J I, et al. The width of the third ventricle associates with cognition and behaviour in motor neuron disease [J]. Acta Neurol Scand, 2019, 139(2): 118-27.  [698] VESA J, SU H, WATTS G D, et al. Valosin containing protein associated inclusion body myopathy: abnormal vacuolization, autophagy and cell fusion in myoblasts [J]. Neuromuscul Disord, 2009, 19(11): 766-72.  [699] VIITANEN M, KALIMO H. CADASIL: Hereditary arteriopathy leading to multiple brain infarcts and dementia [M]//KALARIA R N, INCE P. Vascular Factors in Alzheimer's Disease. 2000: 273-84.  [700] VINCIGUERRA C, DI FONZO A, MONFRINI E, et al. Case report: Asp194Ala variant in MFN2 is associated with ALS-FTD in an Italian family [J]. Frontiers in Genetics, 2023, 14.  [701] VISVANATHAN R. Undernutrition and housebound older people [J]. Nutrition & Dietetics, 2009, 66(4): 238-42.  [702] VOISARD P, DIOFANO F, GLAZIER A A, et al. CRISPR/Cas9-Mediated Constitutive Loss of VCP (Valosin-Containing Protein) Impairs Proteostasis and Leads to Defective Striated Muscle Structure and Function In Vivo [J]. International Journal of Molecular Sciences, 2022, 23(12).  [703] VONGS A, SOLLY K J, KISS L, et al. A miniaturized homogenous assay of mitochondrial membrane potential [J]. Assay Drug Dev Technol, 2011, 9(4): 373-81.  [704] WAGGONER B, KOVACH M J, WINKELMAN M, et al. Heterogeneity in familial dominant Paget disease of bone and muscular dystrophy [J]. American Journal of Medical Genetics, 2002, 108(3): 187-91.  [705] WANG H. Phylogenetic analysis of microRNA biomarkers for amyotrophic lateral sclerosis [J]. Biocell, 2021, 45(3): 547-61.  [706] WANG J K T, LANGFELDER P, HORVATH S, et al. Exosomes and Homeostatic Synaptic Plasticity Are Linked to Each other and to Huntington's, Parkinson's, and Other Neurodegenerative Diseases by Database-Enabled Analyses of Comprehensively Curated Datasets [J]. Front Neurosci, 2017, 11: 149.  [707] WANG Z X, ZHANG W, YANG Y L, et al. Clinical and radiological features of the late-onset methylmalonic aciduria: A review of three cases [J]. Chinese Journal of Neurology, 2004, 37(4): 327-30.  [708] WANG Z Z, JENSSON O, THORSTEINSSON L, et al. Microvascular degeneration in hereditary cystatin C amyloid angiopathy of the brain [J]. Apmis, 1997, 105(1): 41-7.  [709] WARWICK T C, MONINGI V, JAMI P, et al. Neuroleptic malignant syndrome variant in a patient receiving donepezil and olanzapine [J]. Nature Clinical Practice Neurology, 2008, 4(3): 170-4.  [710] WEI Y C, HSU C H, HUANG W Y, et al. White Matter Integrity Underlies the Physical-Cognitive Correlations in Subjective Cognitive Decline [J]. Front Aging Neurosci, 2021, 13: 700764.  [711] WEIHL C C, BALOH R H, LEE Y J, et al. Targeted sequencing and identification of genetic variants in sporadic inclusion body myositis [J]. Neuromuscular Disorders, 2015, 25(4): 289-96.  [712] WEIHL C C, MILLER S E, HANSON P I, et al. Transgenic expression of inclusion body myopathy associated mutant p97/VCP causes weakness and ubiquitinated protein inclusions in mice [J]. Human Molecular Genetics, 2007, 16(8): 919-28.  [713] WETMORE J B, HONEA R A, VIDONI E D, et al. Role of lean body mass in estimating glomerular filtration rate in Alzheimer disease [J]. Nephrol Dial Transplant, 2011, 26(7): 2222-31.  [714] WIEDL A, FöRCH S, FENWICK A, et al. Prognostic value of orthogeriatric assessment parameters on mortality: a 2-year follow-up [J]. Eur J Trauma Emerg Surg, 2022, 48(4): 2905-14.  [715] WILLIAMS A M, KRULL K R, HOWELL C R, et al. Physiologic Frailty and Neurocognitive Decline Among Young-Adult Childhood Cancer Survivors: A Prospective Study From the St Jude Lifetime Cohort [J]. J Clin Oncol, 2021, 39(31): 3485-95.  [716] WINDAHL K, IRVING G F, ALMQUIST T, et al. Prevalence and Risk of Protein-Energy Wasting Assessed by Subjective Global Assessment in Older Adults With Advanced Chronic Kidney Disease: Results From the EQUAL Study [J]. Journal of Renal Nutrition, 2018, 28(3): 165-74.  [717] WOLF J, SAFER A, WöHRLE J C, et al. Causes of death in amyotrophic lateral sclerosis. Results from the Rhineland-Palatinate ALS registry [J]. Nervenarzt, 2017, 88(8): 911-8.  [718] WON C W, YOO H J, YU S H, et al. Lists of geriatric syndromes in the Asian-Pacific geriatric societies [J]. European Geriatric Medicine, 2013, 4(5): 335-8.  [719] WU R, SHAO S, YIN L, et al. Frameshift mutation in SQSTM1 causes proximal myopathy with rimmed vacuoles: A case report [J]. Frontiers in Neurology, 2023, 14.  [720] WU X, ZHANG T, ZHANG Y, et al. Natural population cohort study on long-lived adults: West China longevity and ageing procedure (WCLAP) [J]. BMJ Open, 2022, 12(6): e055407.  [721] WU Z H, WU A, DONG J, et al. Grape skin extract improves muscle function and extends lifespan of a <i>Drosophila</i> model of Parkinson's disease through activation of mitophagy [J]. Experimental Gerontology, 2018, 113: 10-7.  [722] WURTMAN R J. Narcolepsy and the hypocretins [J]. Metabolism-Clinical and Experimental, 2006, 55(10): S36-S9.  [723] WYTTENBACH A. Role of heat shock proteins during polyglutamine neurodegeneration - Mechanisms and hypothesis [J]. Journal of Molecular Neuroscience, 2004, 23(1-2): 69-95.  [724] XIONG Y, ZHAO K, WU J X, et al. <i>HDAC6</i> mutations rescue human tau-induced microtubule defects in <i>Drosophila</i> [J]. Proceedings of the National Academy of Sciences of the United States of America, 2013, 110(12): 4604-9.  [725] XU H, BHASKARAN S, PIEKARZ K M, et al. Age Related Changes in Muscle Mass and Force Generation in the Triple Transgenic (3xTgAD) Mouse Model of Alzheimer's Disease [J]. Front Aging Neurosci, 2022, 14: 876816.  [726] XU L Z, WANG D, ZHAO L, et al. C9orf72 poly(PR) aggregation in nucleus induces ALS/FTD-related neurodegeneration in cynomolgus monkeys [J]. Neurobiology of Disease, 2023, 184.  [727] XU W, ZHAO X, ZENG M, et al. Exercise for frailty research frontiers: a bibliometric analysis and systematic review [J]. Front Med (Lausanne), 2024, 11: 1341336.  [728] YADAV N, RAJA P, SHETTY S S, et al. Neuronal Intranuclear Inclusion Disease A Rare Etiology for Rapidly Progressive Dementia [J]. Alzheimer Disease & Associated Disorders, 2019, 33(4): 359-61.  [729] YAGIHASHI T, KATO M, IZUMI K, et al. Case Report: Adult Phenotype of Mulvihill-Smith Syndrome [J]. American Journal of Medical Genetics Part A, 2009, 149A(3): 496-500.  [730] YAMADA S, NIWA J, ISHIGAKI S, et al. Archaeal proteasomes effectively degrade aggregation-prone proteins and reduce cellular toxicities in mammalian cells [J]. J Biol Chem, 2006, 281(33): 23842-51.  [731] YAMADA S, TOKUMOTO M, KANSUI Y, et al. Severe metabolic alkalosis, hypokalemia, and respiratory acidosis induced by the Chinese herbal medicine yokukansan in an elderly patient with muscle weakness and drowsiness [J]. CEN Case Rep, 2013, 2(1): 23-7.  [732] YAMADA T, HIRAYAMA K, AKAI J. One autopsy case of Levine-Critchley syndrome. Clinico-pathological study [J]. Clinical Neurology, 1986, 26(2): 156-61.  [733] YAMADA Y, UMEGAKI H, KINOSHITA F, et al. Cross-Sectional Examination of Homocysteine Levels with Sarcopenia and Its Components in Memory Clinic Outpatients [J]. J Alzheimers Dis, 2021, 82(3): 975-84.  [734] YAMADA Y, UMEGAKI H, SUGIMOTO T, et al. Relationship of creatinine cystatin C ratio with muscle mass and grip strength in memory clinic outpatients [J]. Exp Gerontol, 2022, 168: 111935.  [735] YAMASHITA M, YAMAMOTO T, NAKAMURA K. Concurrence of amyotrophic lateral sclerosis with limbic degeneration and Alzheimer's disease [J]. Neuropathology, 1997, 17(4): 334-9.  [736] YANG E J, LEE S H. Anti-Inflammatory Effects of Chaenomeles sinensis Extract in an ALS Animal Model [J]. Frontiers in Bioscience-Landmark, 2023, 28(12).  [737] YANG S, TIAN M, DAI Y, et al. Infection and chronic disease activate a brain-muscle signaling axis that regulates muscle performance [J]. bioRxiv, 2022.  [738] YANG Y, DA J, YUAN J, et al. One-year change in sarcopenia was associated with cognitive impairment among haemodialysis patients [J]. J Cachexia Sarcopenia Muscle, 2023, 14(5): 2264-74.  [739] YAO J J, NI M K, TIAN S S, et al. A Gain-of-function Mutation in the Gating Domain of ITPR1 Impairs Motor Movement and Increases Thermal and Mechanical Sensitivity [J]. Neuroscience, 2023, 522: 11-22.  [740] YASSIN M, GARTI A, KHATIB M, et al. Retentive Cup Arthroplasty in Selected Hip Fracture Patients-A Prospective Series With a Minimum 3-Year Follow-Up [J]. Geriatr Orthop Surg Rehabil, 2016, 7(4): 178-82.  [741] YATSUKA H, HADA K, SHIRAISHI H, et al. Exosc2 deficiency leads to developmental disorders by causing a nucleotide pool imbalance in zebrafish [J]. Biochem Biophys Res Commun, 2020, 533(4): 1470-6.  [742] YE C, KONG L, WANG Y, et al. Causal associations of sarcopenia-related traits with cardiometabolic disease and Alzheimer's disease and the mediating role of insulin resistance: A Mendelian randomization study [J]. Aging Cell, 2023, 22(9): e13923.  [743] YI S W, HONG J S, OHRR H, et al. Agent Orange exposure and disease prevalence in Korean Vietnam veterans: the Korean veterans health study [J]. Environ Res, 2014, 133: 56-65.  [744] YIN H Z, NALBANDIAN A, HSU C I, et al. Slow development of ALS-like spinal cord pathology in mutant valosin-containing protein gene knock-in mice [J]. Cell Death & Disease, 2012, 3.  [745] YIN Z, VALKENBURG F, HORNIX B E, et al. Progressive Motor Deficit is Mediated by the Denervation of Neuromuscular Junctions and Axonal Degeneration in Transgenic Mice Expressing Mutant (P301S) Tau Protein [J]. J Alzheimers Dis, 2017, 60(s1): S41-s57.  [746] YOSHIDA Y, HAMADA R, KAMITSUCHIBASHI H, et al. Determination of urine thiocyanate in patients with amyotrophic lateral sclerosis [J]. Acta Neurol Scand, 1989, 80(5): 444-50.  [747] YOUDING X, QIANJIN G, ERLI W. Skeletal muscle function and exercise regulating cognitive function of the older adults [J]. Chinese Journal of Tissue Engineering Research, 2022, 26(33): 5400-6.  [748] YUN J H, KIM D H, CHANG M C. A Simple Bedside Exercise Method to Enhance Lower Limb Muscle Strength in Moderate Alzheimer's Disease Patients with Sarcopenia [J]. Healthcare (Basel), 2021, 9(6).  [749] ZACCO E, GRAñA-MONTES R, MARTIN S R, et al. RNA as a key factor in driving or preventing self-assembly of the TAR DNA-binding protein 43 [J]. J Mol Biol, 2019, 431(8): 1671-88.  [750] ZAIB S, MUNIR R, YOUNAS M T, et al. Hybrid Quinoline-Thiosemicarbazone Therapeutics as a New Treatment Opportunity for Alzheimer's Disease-Synthesis, In Vitro Cholinesterase Inhibitory Potential and Computational Modeling Analysis [J]. Molecules, 2021, 26(21).  [751] ZAMBONE M A, LIBERMAN S, GARCIA M L B. Anthropometry, bioimpedance and densitometry: Comparative methods for lean mass body analysis in elderly outpatients from a tertiary hospital [J]. Exp Gerontol, 2020, 138: 111020.  [752] ZAMBRANO K P M, LóPEZ V N G, SOLANO M A C, et al. Approach to the prescription of physical activity in the elderly [J]. Revista Latinoamericana De Hipertension, 2021, 16(5): 341-+.  [753] ZANINI G, SELLERI V, NASI M, et al. Mitochondrial and Endoplasmic Reticulum Alterations in a Case of Amyotrophic Lateral Sclerosis Caused by TDP-43 A382T Mutation [J]. International Journal of Molecular Sciences, 2022, 23(19).  [754] ZARĘBA-KOZIOŁ M, BURDUKIEWICZ M, WYSŁOUCH-CIESZYŃSKA A. Intracellular Protein S-Nitrosylation-A Cells Response to Extracellular S100B and RAGE Receptor [J]. Biomolecules, 2022, 12(5).  [755] ZARĘBA-KOZIOŁ M, BURDUKIEWICZ M, WYSŁOUCH-CIESZYŃSKA A. Intracellular Protein S-Nitrosylation—A Cells Response to Extracellular S100B and RAGE Receptor [J]. Biomolecules, 2022, 12(5).  [756] ZENG T, CHEN Y, HUANG H, et al. Neuronal Intranuclear Inclusion Disease with NOTCH2NLC GGC Repeat Expansion: A Systematic Review and Challenges of Phenotypic Characterization [J]. Aging Dis, 2024.  [757] ZENGARINI E, GIACCONI R, MANCINELLI L, et al. Prognosis and Interplay of Cognitive Impairment and Sarcopenia in Older Adults Discharged from Acute Care Hospitals [J]. J Clin Med, 2019, 8(10).  [758] ZHANG J, NA X, LI Z, et al. Sarcopenic obesity is part of obesity paradox in dementia development: evidence from a population-based cohort study [J]. BMC Med, 2024, 22(1): 133.  [759] ZHANG K, LIU Q, LIU K Q, et al. <i>ANXA11</i> mutations prevail in Chinese ALS patients with and without cognitive dementia [J]. Neurology-Genetics, 2018, 4(3).  [760] ZHANG K, LU Y, CHEN J, et al. NEK1 and GRN mutations coexist in a sporadic Chinese Hui descent ALS patient [J]. Amyotroph Lateral Scler Frontotemporal Degener, 2020, 21(7-8): 624-6.  [761] ZHANG T, ZHANG Y, LV Z, et al. Sarcopenia and motoric cognitive risk syndrome: a moderated mediation model [J]. BMC Geriatr, 2022, 22(1): 141.  [762] ZHANG X L, MENG Y X, ZHANG W W, et al. RETRACTED: Diagnostic Values of Advanced Glycation End Products and Homocysteine in Patients with Alzheimer's Disease and Sarcopenia (Retracted Article) [J]. Computational and Mathematical Methods in Medicine, 2022, 2022.  [763] ZHANG Y T, LI K, PU C Q, et al. A novel application of tau PET in the diagnosis of sporadic inclusion body myositis A case report [J]. Medicine, 2020, 99(31).  [764] ZHOU Z, RYAN J, NELSON M R, et al. The association of allopurinol with persistent physical disability and frailty in a large community based older cohort [J]. J Am Geriatr Soc, 2023, 71(9): 2798-809.  [765] ZHU G M, ZHANG W W, LIU Y, et al. Arterioles in cerebral amyloid angiopathy and vascular dementia [J]. Chinese Medical Journal, 2009, 122(24): 2985-8.  [766] ZUCCHELLI A, MANZONI F, MORANDI A, et al. The association between low skeletal muscle mass and delirium: results from the nationwide multi-centre Italian Delirium Day 2017 [J]. Aging Clin Exp Res, 2022, 34(2): 349-57.  [767] ZULIANI G, GALVANI M, SIOULIS F, et al. Discharge diagnosis and comorbidity profile in hospitalized older patients with dementia [J]. Int J Geriatr Psychiatry, 2012, 27(3): 313-20. |
| Inappropriate publication type (n = 981) | [1] 134th ANA Meeting [J]. Annals of Neurology, 2009, 66.  [2] Japanese Society of Neuropathology - Abstracts of the 51st Annual Meeting [J]. Neuropathology, 2010, 30(3).  [3] Experimental Biology 2012, EB [J]. FASEB Journal, 2012, 26.  [4] Communications to the British Geriatrics Society Autumn Meeting 2011 [J]. Age and Ageing, 2012, 41.  [5] Singapore Health and Biomedical Congress, SHBC 2013 [J]. Annals of the Academy of Medicine Singapore, 2013, 42: S1.  [6] Proceedings of the 114th Meeting of the British Neuropathological Society [J]. Neuropathology and Applied Neurobiology, 2013, 39.  [7] 25th International Symposium on ALS/MND [J]. Amyotrophic Lateral Sclerosis and Frontotemporal Degeneration, 2014, 15.  [8] Abstracts of the 11th International Congress of the European Union Geriatric Medicine Society - Geriatric Medicine for Future Europeans - Successful Aging Creates New Challenges [J]. European Geriatric Medicine, 2015, 6.  [9] Abstracts of the 9th International Conference on Cachexia, Sarcopenia, and Muscle Wasting [J]. Journal of Cachexia, Sarcopenia and Muscle, 2017, 8(1).  [10] British Geriatrics Society Communications to the Autumn Meeting 2017 [J]. Age and Ageing, 2018, 47.  [11] ANZAN Annual Scientific Meeting 2019 [J]. Journal of Neurology, Neurosurgery and Psychiatry, 2019, 90(e7).  [12] Barshop Symposium on Aging 2018 Abstracts [J]. Pathobiology of Aging and Age-related Diseases, 2019, 9.  [13] Winter Conference Live 2020: Micronutrient Malnutrition Across the Life Course, Sarcopenia and Frailty [J]. Proceedings of the Nutrition Society, 2021, 80(OCE1).  [14] Abstracts of the 18th Congress of the European Geriatric Medicine Society [J]. European Geriatric Medicine, 2022, 13.  [15] Retracted: Diagnostic Values of Advanced Glycation End Products and Homocysteine in Patients with Alzheimer’s Disease and Sarcopenia(Computational and Mathematical Methods in Medicine (2022) 2022 (8949048) DOI: 10.1155/2022/8949048) [J]. Computational and Mathematical Methods in Medicine, 2023, 2023.  [16] ABAY R J, GOLD L S, ANDREWS J. Sarcopenia and ADL disability among hospitalized older adults with dementia in the health ABC study [J]. Journal of the American Geriatrics Society, 2020, 68(SUPPL 1): S297.  [17] ABBHI V, PIPLANI P. Rho-kinase (ROCK) Inhibitors - A Neuroprotective Therapeutic Paradigm with a Focus on Ocular Utility [J]. Current Medicinal Chemistry, 2020, 27(14): 2222-56.  [18] ABE K. Clinical and molecular analysis of neurodegenerative diseases [J]. The Tohoku journal of experimental medicine, 1997, 181(4): 389-409.  [19] ABE K. An early history of Japanese amyotrophic lateral sclerosis (ALS)-related diseases and the current development [J]. Clinical Neurology, 2018, 58(3): 141-65.  [20] ABOU M B, SUN L, WEI H. Approaches to Optimizing Dantrolene Neuroprotection for the Treatment of Alzheimer's Disease [J]. Current Alzheimer Research, 2020, 17(4): 324-8.  [21] ABYAD A, HAMMAMI S. Challenges of geriatrics and gerontology education in the eastern mediterranean region: Reflection on the collaboration of MEAMA and ATG [J]. European Geriatric Medicine, 2018, 9: S126.  [22] ADAMI R, BOTTAI D. Curcumin and neurological diseases [J]. Nutritional Neuroscience, 2022, 25(3): 441-61.  [23] ADAMI R, SCESA G, BOTTAI D. Stem cell transplantation in neurological diseases: improving effectiveness in animal models [J]. Front Cell Dev Biol, 2014, 2: 17.  [24] AGIWALE B T, JADHAV A B, KSHIRSAGAR S J, et al. Liposomal Drug Delivery System as an Emerging Technique for Treatment of “Neurodegenerative Diseases” [J]. Current Nanomedicine, 2023, 13(1): 17-26.  [25] AIT-EL-MKADEM SAADI S, CHAUSSENOT A, BANNWARTH S, et al. CHCHD10-Related Disorders [M]//ADAM M P, FELDMAN J, MIRZAA G M, et al. GeneReviews(®). Seattle (WA); University of Washington, Seattle  Copyright © 1993-2024, University of Washington, Seattle. GeneReviews is a registered trademark of the University of Washington, Seattle. All rights reserved. 1993.  [26] AKISHITA M. Polypharmacy and frailty [J]. Aging Medicine and Healthcare, 2019, 10: 6.  [27] AL RAJEH S, BADEMOSI O, ISMAIL H, et al. A community survey of neurological disorders in Saudi Arabia: the Thugbah study [J]. Neuroepidemiology, 1993, 12(3): 164-78.  [28] AL-KURAISHY H M, JABIR M S, SULAIMAN G M, et al. The role of statins in amyotrophic lateral sclerosis: protective or not? [J]. Front Neurosci, 2024, 18: 1422912.  [29] AL-SHAMAHI A, KIRKHAM K, HOOKES L. Society for Neuroscience - 39th Annual Meeting. Part 2 - Novel therapies for neurodegenerative disorders and other CNS diseases [J]. IDrugs, 2009, 12(12): 734-7.  [30] AL-SHAMAHI A, KIRKHAM K, HOOKES L. Society for Neuroscience-39th Annual Meeting Part 2-Novel therapies for neurodegenerative disorders and other CNS diseases 17-21 October 2009, Chicago, IL, USA [J]. Idrugs, 2009, 12(12): 734-7.  [31] ALAQEEL A M, ABOU AL-SHAAR H, SHARIFF R K, et al. THE ROLE OF RNA METABOLISM IN NEUROLOGICAL DISEASES [J]. Balkan Journal of Medical Genetics, 2015, 18(2): 5-13.  [32] ALENAZY M F, ALJOHAR H I, ALRUWAILI A R, et al. Gut Microbiota Dynamics in Relation to Long-COVID-19 Syndrome: Role of Probiotics to Combat Psychiatric Complications [J]. Metabolites, 2022, 12(10).  [33] ALHASANIAH A H. l-carnitine: Nutrition, pathology, and health benefits [J]. Saudi J Biol Sci, 2023, 30(2): 103555.  [34] ALTAMAR G, DUEñAS E, CASTAñEDA I. Results of an orthogeriatric unit in Latin America: First year of work in Southwest Colombia [J]. Osteoporosis International, 2020, 31(SUPPL 1): S416.  [35] ALTMAN K W, RICHARDS A, GOLDBERG L, et al. Dysphagia in Stroke, Neurodegenerative Disease, and Advanced Dementia [J]. Otolaryngologic Clinics of North America, 2013, 46(6): 1137-49.  [36] ALTUNTAŞ Y. Approach Toward Diabetes Treatment in the Elderly [J]. Sisli Etfal Hastan Tip Bul, 2019, 53(2): 96-102.  [37] ALYAZIDI A M, SHAKHAU A, HUANG A, et al. Frailty among older adult state hospital patients [J]. American Journal of Geriatric Psychiatry, 2018, 26(3): S109-S10.  [38] AMBROSE C T. The role of capillaries in the lesser ailments of old age and in Alzheimer's disease and vascular dementia: The potential of pro-therapeutic angiogenesis [J]. Journal of Alzheimer's Disease, 2016, 54(1): 31-43.  [39] AMICO C D, TATA A, PELLEGRINO E, et al. Genome editing in stem cells for genetic neurodisorders [M]//PETRIS G. Curing Genetic Diseases through Genome Reprogramming. 2021: 403-38.  [40] AMINI N, IBN HACH M, DUPONT J, et al. THE INTERRELATIONSHIP BETWEEN SARCOPENIA AND MILD COGNITIVE IMPAIRMENT, ALZHEIMER'S DISEASE AND DEMENTIA: A SYSTEMATIC REVIEW [J]. Aging Clinical and Experimental Research, 2023, 35: S365-S6.  [41] ANGELOPOULOU E, PYRGELIS E S, AHIRE C, et al. Functional Implications of Protein Arginine Methyltransferases (PRMTs) in Neurodegenerative Diseases [J]. Biology (Basel), 2023, 12(9).  [42] ANNUNZIATO L, SECONDO A, PIGNATARO G, et al. New perspectives for selective NCX activators in neurodegenerative diseases [J]. Cell Calcium, 2020, 87: 102170.  [43] ANNWEILER C, SOUBERBIELLE J C, SCHOTT A M, et al. [Vitamin D in the elderly: 5 points to remember] [J]. Gériatrie et psychologie neuropsychiatrie du vieillissement, 2011, 9(3): 259-67.  [44] ANTIKAINEN R, STRANDBERG T, BARBAGALLO M, et al. Paradoxes in the old age - Reverse epidemiology [J]. European Geriatric Medicine, 2012, 3: S10-S1.  [45] AOKI Y, MOCHIZUKI Y, ISOZAKI E, et al. [Autopsy case of frontotemporal lobar degeneration with motor neuron disease associated with numerous diffuse plaques, pretangles and neuropil threads] [J]. Rinsho Shinkeigaku, 2014, 54(4): 325-9.  [46] AOYAGI Y, TAYA M, OHASHI M, et al. Neuronal intranuclear inclusion disease presenting with dysphagia: a report of three cases [J]. Neurocase, 2020, 26(4): 252-7.  [47] ARAKI A. Diabetes education in elderly patients to maintain quality of life [J]. Nippon rinsho Japanese journal of clinical medicine, 2006, 64(1): 134-9.  [48] ARATA H, TAKASHIMA H. Familial prion disease (GSS, familial CJD, FFI) [J]. Nippon rinsho Japanese journal of clinical medicine, 2007, 65(8): 1433-7.  [49] AROOS R, CHEN M, LIM M Y, et al. Motoric cognitive risk syndrome (MCR): Prevalence and associated factors in older adults [J]. Journal of the American Geriatrics Society, 2018, 66: S93.  [50] ARTUSI C A, DE MERCANTI S, BUSSO M, et al. Sialorrhea and ultrasound-guided botulinum toxin-A: A 2-year prospective study [J]. Neurological Sciences, 2015, 36(2): S209-S10.  [51] ASAI K, SUMI-AKAMARU H, NISHIKAWA A, et al. Fused in sarcoma (FUS) pathology observed in an autopsy case of ALS/MND-plus clinical syndrome [J]. Journal of the Neurological Sciences, 2019, 405: 339.  [52] ASANO M, FUJIMOTO N, GEMBA K, et al. Acute onset of brain atrophy and dementia in a patient with small cell lung cancer: a case report [J]. Lung Cancer, 2011, 71(3): 367-9.  [53] ASHRAFI M R. The potential of stem cell therapies for pediatric neurological disorders [J]. BioImpacts, 2018, 8: 3.  [54] ASKANAS V, ENGEL W K. Inclusion-body myositis: muscle-fiber molecular pathology and possible pathogenic significance of its similarity to Alzheimer's and Parkinson's disease brains [J]. Acta Neuropathologica, 2008, 116(6): 583-95.  [55] ATHIRA K V, SADANANDAN P, CHAKRAVARTY S. Repurposing Vorinostat for the Treatment of Disorders Affecting Brain [J]. NeuroMolecular Medicine, 2021, 23(4): 449-65.  [56] AULITZKY A, FRIEDRICH K, GLäSER D, et al. A complex form of hereditary spastic paraplegia in three siblings due to somatic mosaicism for a novel SPAST mutation in the mother [J]. J Neurol Sci, 2014, 347(1-2): 352-5.  [57] AUNAN J R, WATSON M M, HAGLAND H R, et al. Molecular and biological hallmarks of ageing [J]. British Journal of Surgery, 2016, 103(2): e29-e46.  [58] BABIZHAYEV M A, KASUS-JACOBI A, VISHNYAKOVA K S, et al. Novel neuroendocrine and metabolic mechanism provides the patented platform for important rejuvenation therapies: targeted therapy of telomere attrition and lifestyle changes of telomerase activity with the timing of neuron-specific imidazole-containing dipeptide-dominant pharmaconutrition provision [J]. Recent Pat Endocr Metab Immune Drug Discov, 2014, 8(3): 153-79.  [59] BADII M, GOYAL N. A Central or Peripheral Disease? [J]. Journal of Clinical Neuromuscular Disease, 2023, 24: S14.  [60] BAENA C P, HAAGSMA A B, SOUZA D, et al. Longitudinal Analysis of Handgrip Strength and Cognitive Function during Aging- Share [J]. Circulation, 2022, 145.  [61] BAGHERI H, GHASEMI F, BARRETO G E, et al. The effects of statins on microglial cells to protect against neurodegenerative disorders: A mechanistic review [J]. BioFactors, 2020, 46(3): 309-25.  [62] BAGHERI S, HADDADI R, SAKI S, et al. Neuroprotective effects of coenzyme Q10 on neurological diseases: a review article [J]. Frontiers in Neuroscience, 2023, 17.  [63] BAHAT G, BAY I, SELCUK AKPINAR T, et al. Determinants of falls and/or fear of falls in community dwelling elderly [J]. Osteoporosis International, 2013, 24(1): S315-S6.  [64] BAHAT G, BOZKURT M E, CATIKKAS N M, et al. THE LONGITUDINAL ASSOCIATIONS OF SARCOPENIA DEFINITIONS WITH ADVERSE OUTCOMES: A COMPARATIVE STUDY [J]. Aging Clinical and Experimental Research, 2022, 34: S51.  [65] BALDUCCI L. Anemia, fatigue and aging [J]. Transfusion Clinique et Biologique, 2010, 17(5-6): 375-81.  [66] BALICZA P, GROSZ Z, PALASTI A, et al. Exome sequencing identifies CHCHD2 variant in a patient with early onset multiple system atrophy and coexisting mitochondrial pathology in muscle [J]. European Journal of Neurology, 2020, 27: 365.  [67] BARCIA G, ASSOULINE Z, PENNISI A, et al. Expanding the clinical spectrum of MTTF mutations [J]. Molecular Genetics and Metabolism Reports, 2019, 21.  [68] BARCIA G, ASSOULINE Z, PENNISI A, et al. Expanding the clinical spectrum of <i>MTTF</i> mutations [J]. Molecular Genetics and Metabolism Reports, 2019, 21.  [69] BARONI M, PRENNI V, FERRACCI M, et al. Fracture and dementia: From epide-miologicalto biological connections [J]. Osteoporosis International, 2017, 28: S614-S5.  [70] BARQUERO-JIMéNEZ M S, DOMíNGUEZ-SALGADO M. Dementia in progressive supranuclear paralysis patients [J]. Revista de Neurologia, 2001, 32(11): 1071-3.  [71] BASRI R, AWAN F M, YANG B B, et al. Brain-protective mechanisms of autophagy associated circRNAs: Kick starting self-cleaning mode in brain cells via circRNAs as a potential therapeutic approach for neurodegenerative diseases [J]. Frontiers in Molecular Neuroscience, 2023, 15.  [72] BASRI R, AWAN F M, YANG B B, et al. Brain-protective mechanisms of autophagy associated circRNAs: Kick starting self-cleaning mode in brain cells <i>via</i> circRNAs as a potential therapeutic approach for neurodegenerative diseases [J]. Frontiers in Molecular Neuroscience, 2023, 15.  [73] BASSO M, PENNUTO M. Serine phosphorylation and arginine methylation at the crossroads to neurodegeneration [J]. Experimental Neurology, 2015, 271: 77-83.  [74] BAUER J M, KAISER M J, SIEBER C C. Sarcopenia in Nursing Home Residents [J]. Journal of the American Medical Directors Association, 2008, 9(8): 545-51.  [75] BAUMAN A, MEROM D, BULL F C, et al. Updating the Evidence for Physical Activity: Summative Reviews of the Epidemiological Evidence, Prevalence, and Interventions to Promote "Active Aging" [J]. Gerontologist, 2016, 56: S268-S80.  [76] BECKER N, HAFNER T, PISHNAMAZ M, et al. Patient-specific risk factors for adverse outcomes following geriatric proximal femur fractures [J]. European journal of trauma and emergency surgery : official publication of the European Trauma Society, 2022, 48(2): 753-61.  [77] BEELDMAN E, GOVAARTS R, RAAPHORST J, et al. A cognitive screening tool for ALS patients: The ALS-FTD-Cog, preliminary results [J]. Neurology, 2016, 86(16).  [78] BEKENSTEIN U, SOREQ H. Heterogeneous nuclear ribonucleoprotein A1 in health and neurodegenerative disease: from structural insights to post-transcriptional regulatory roles [J]. Mol Cell Neurosci, 2013, 56: 436-46.  [79] BELL D S H. Protean manifestations of vitamin D deficiency, part 3: Association with cardiovascular disease and disorders of the central and peripheral nervous systems [J]. Southern Medical Journal, 2011, 104(5): 340-4.  [80] BELOVA K, GORDZHELADZE K, BELOV M. PREVALENCE OF GERIATRIC SYNDROMES IN PATIENTS 60 YEARS AND OLDER WITH HIP FRACTURE [J]. Aging Clinical and Experimental Research, 2022, 34: S330-S1.  [81] BELZIL V, VALDMANIS P, DION P, et al. Mutations in FUS cause fals and SALS in French and French Canadian populations [J]. Amyotrophic Lateral Sclerosis, 2009, 10: 131-2.  [82] BENATAR M, WUU J, ANDERSEN P M, et al. Design of a Phase 3, Randomized, Placebocontrolled Trial of Tofersen Initiated in Clinically Pre-symptomatic SOD1 Mutation Carriers with a Longitudinal Natural History Run-in [J]. Neurology, 2021, 96(15 SUPPL 1).  [83] BENATAR M, WUU J, MCHUTCHISON C, et al. Preventing amyotrophic lateral sclerosis: insights from pre-symptomatic neurodegenerative diseases [J]. Brain, 2022, 145(1): 27-44.  [84] BENCIVENGA L, STRUMIA M, BRUNO V, et al. ASSOCIATION BETWEEN PLASMA GROWTH DIFFERENTIATION FACTOR 15 AND BLOOD PRESSURE VARIABILITY IN COMMUNITY-DWELLING OLDER ADULTS: THE MAPT STUDY [J]. Journal of Nutrition, Health and Aging, 2022, 26(4): 463-4.  [85] BENNETT C F, KORDASIEWICZ H B, CLEVELAND D W. Antisense Drugs Make Sense for Neurological Diseases [J]. Annu Rev Pharmacol Toxicol, 2021, 61: 831-52.  [86] BENNETT C F, KRAINER A R, CLEVELAND D W. Antisense Oligonucleotide Therapies for Neurodegenerative Diseases [J]. Annu Rev Neurosci, 2019, 42: 385-406.  [87] BENNETT C F, KRAINER A R, CLEVELAND D W. Antisense Oligonucleotide Therapies for Neurodegenerative Diseases [M]//ROSKA B, ZOGHBI H Y. Annual Review of Neuroscience, Vol 42. 2019: 385-406.  [88] BENNETT FRANK C, KRAINER A R, CLEVELAND D W. Antisense Oligonucleotide Therapies for Neurodegenerative Diseases [Z]. 2019: 385-406.10.1146/annurev-neuro-070918-050501  [89] BERSANO A, DEL BO R, LAMPERTI C, et al. Inclusion body myopathy and frontotemporal dementia caused by a novel VCP mutation [J]. Neurobiol Aging, 2009, 30(5): 752-8.  [90] BERTELSEN A S, MASUD T, SUETTA C, et al. ROBot assisted physical training of older patients during acUte hospitaliSaTion (ROBUST): Study protocol for a randomised controlled trial [J]. European Geriatric Medicine, 2022, 13: S311.  [91] BERTHIAUME J M, SURYADEVARA V, KLUPPEL M, et al. Musculoskeletal alterations in a mouse model of alzheimer's disease [J]. JBMR Plus, 2019, 3.  [92] BERTOLOTTI M, LONARDO A, MUSSI C, et al. Nonalcoholic fatty liver disease and aging: epidemiology to management [J]. World J Gastroenterol, 2014, 20(39): 14185-204.  [93] BHAT M A, DHANESHWAR S. Neurodegenerative Diseases: New Hopes and Perspectives [J]. Curr Mol Med, 2024, 24(8): 1004-32.  [94] BHATANE D, PAMSHONG S R, SARNAIK S, et al. Potential applications of mesoporous silica nanoparticles for the treatment of neurological disorders [J]. Journal of Drug Delivery Science and Technology, 2023, 89.  [95] BIANCO A, ANTONACCI Y, LIGUORI M. Sex and Gender Differences in Neurodegenerative Diseases: Challenges for Therapeutic Opportunities [J]. International Journal of Molecular Sciences, 2023, 24(7).  [96] BIBA A, BAüMER D, GUBBIN J, et al. Functional analysis of FUS/TLS mutations involved in ALS [J]. Amyotrophic Lateral Sclerosis, 2011, 12: 128.  [97] BICALHO M A, SOARES T, SIMAS K, et al. STUDY OF CORRELATION BETWEEN BODY COMPOSITION AND THE ABSENCE OF OBJECTIVE COGNITIVE OR FUNCTIONAL IMPAIRMENT, MILD COGNITIVE IMPAIRMENT AND ALZHEIMER'S DEMENTIA [J]. Alzheimer's and Dementia, 2018, 14(7): P755.  [98] BICCHI I, EMILIANI C, VESCOVI A, et al. The Big Bluff of Amyotrophic Lateral Sclerosis Diagnosis: The Role of Neurodegenerative Disease Mimics [J]. Neurodegenerative Diseases, 2015, 15(6): 313-21.  [99] BIFERI M G, BOS C, TANGUY Y, et al. New model of ubiquilin2-related ALS using AAV vectors [J]. Amyotrophic Lateral Sclerosis and Frontotemporal Degeneration, 2014, 15: 181-2.  [100] BIFERI M G, BOS C, TANGUY Y, et al. AAV-mediated overexpression of Ubiquilin2 mimics ALS and ALS with dementia in naive mice [J]. Neuromuscular Disorders, 2014, 24(9-10): 921.  [101] BILGIç A B. Nutritional approach in sarcopenia [J]. Turk Beyin Damar Hastaliklar Dergisi, 2019, 25: 71-3.  [102] BIRD T D, SMITH C O. Clinical approach to the patient with neurogenetic disease [J]. Handbook of clinical neurology, 2018, 147: 3-9.  [103] BLANCO-BARCA M O, EIRIS-PUñAL J, PEñA-GUITIAN J, et al. [Mixed hypotonia, neurological regression and atrophy of the cerebellum: manifestations that suggest infantile neuroaxonal dystrophy] [J]. Rev Neurol, 2003, 37(1): 25-8.  [104] BOEVE B F, SILBER M H, SAPER C B, et al. Pathophysiology of REM sleep behaviour disorder and relevance to neurodegenerative disease [J]. Brain, 2007, 130: 2770-88.  [105] BOLLHEIMER L C, VOLKERT D, BERTSCH T, et al. [Translational research in geriatrics? A plea based on current biomedical key publications] [J]. Z Gerontol Geriatr, 2013, 46(6): 569-75.  [106] BOLTSHAUSER E, JERUSALEM F, NIEMEYER G, et al. [Kearns syndrome. Progressive external ophthalmoplegia, retinal pigment degeneration and heart conduction disorders] [J]. Schweiz Med Wochenschr, 1977, 107(50): 1880-8.  [107] BONEWALD L. Use it or lose it to age: A review of bone and muscle communication [J]. Bone, 2019, 120: 212-8.  [108] BONHAM-CARTER O, PEDERSEN J, NAJJAR L, et al. Modeling the Effects of Microgravity On Oxidation in Mitochondria: A Protein Damage Assessment Across a Diverse Set of Life Forms; proceedings of the IEEE 13th International Conference on Data Mining (ICDM), Dallas, TX, F Dec 07-10, 2013 [C]. 2013.  [109] BONNEFOY M, GILBERT T. [Body composition and comorbidity in the elderly] [J]. Geriatr Psychol Neuropsychiatr Vieil, 2015, 13 Suppl 1: 29-36.  [110] BOORANASUKSAKUL U, MACDONALD I, STEPHAN B, et al. Body Composition, Sarcopenic Obesity and Cognitive Function in Older Adults: Findings from the National Health and Nutrition Examination Survey (NHANES) 1999-2002 and 2011-2014 [J]. Obesity Facts, 2023, 16: 174-5.  [111] BOTELLA ROMERO F, ALFARO MARTíNEZ J J, LUNA LóPEZ V, et al. [Enteral nutrition in neurological patients: is there enough vitamin D content in commonly used formulas?] [J]. Nutrición hospitalaria : organo oficial de la Sociedad Española de Nutrición Parenteral y Enteral, 2012, 27(2): 341-8.  [112] BOUCHER B J. The Problems of Vitamin D Insufficiency in Older People [J]. Aging and Disease, 2012, 3(4): 313-29.  [113] BOURGOUIN P A, RAHAYEL S, GAUBERT M, et al. Neuroimaging of Rapid Eye Movement Sleep Behavior Disorder [M]//POLITIS M. Imaging in Movement Disorders: Imaging in Non-Parkinsonian Movement Disorders and Dementias, Pt 2. 2019: 185-210.  [114] BOUSQUET A, SANDERSON K, O'SHEA T M, et al. Accelerated Aging and the Life Course of Individuals Born Preterm [J]. Children (Basel), 2023, 10(10).  [115] BOUSSICAULT L, LAFFAIRE J, RINAUDO P, et al. A combination of acamprosate and baclofen (PXT864) as a potential new therapy for amyotrophic lateral sclerosis [J]. Amyotrophic Lateral Sclerosis and Frontotemporal Degeneration, 2018, 19: 232-3.  [116] BOWERS W J, BREAKEFIELD X O, SENA-ESTEVES M. Genetic therapy for the nervous system [J]. Hum Mol Genet, 2011, 20(R1): R28-41.  [117] BOZKURT M E, CATIKKAS N M, OREN M M, et al. THE IMPACT OF FRAILTY AND ITS ASSOCIATIONS AMONG A SAMPLE OF COMMUNITY-DWELLING OLDER ADULTS [J]. Aging Clinical and Experimental Research, 2022, 34: S129-S30.  [118] BOZKURT M E, GUNAYDIN S, KILIC C, et al. The impact of frailty and its associations among a sample of community dwelling older adults [J]. European Geriatric Medicine, 2022, 13: S274.  [119] BRANDãO R, SANTOS H, RODRIGUES M. Cognitive dual-task training to improve balance and functional mobility in Parkinson's disease: A case study [J]. Neurologie und Rehabilitation, 2023, 29: S39-S40.  [120] BRICKER P, FONKEM E. Spectrum of neurologic presentations of intravascular lymphoma: A 643 patient study [J]. Neuro-Oncology, 2013, 15: iii32-iii3.  [121] BRICKER P, WONG E T, FONKEM E. Spectrums of neurologic presentations of intravascular lymphoma (IVL) [J]. Annals of Neurology, 2013, 74: S86.  [122] BRISENDINE M H, DRAKE J C. Early-stage Alzheimer?s disease: are skeletal muscle and exercise the key? [J]. Journal of Applied Physiology, 2023, 134(3): 515-20.  [123] BROOKS D J, PAVESE N. Imaging biomarkers in Parkinson's disease [J]. Progress in Neurobiology, 2011, 95(4): 614-28.  [124] BUENO V, FRASCA D. Mini-review: Angiotensin- converting enzyme 1 (ACE1) and the impact for diseases such as Alzheimer's disease, sarcopenia, cancer, and COVID-19 [J]. Front Aging, 2023, 4: 1117502.  [125] BUFLER J. Diagnosis and treatment of amyotrophic lateral sclerosis [J]. MMW-Fortschritte der Medizin, 2007, 149(SUPPL. 2): 84-7.  [126] BULGAKOVA S V, TRENEVA E V, ZAKHAROVA N O. Гиповитаминоз D у пожилых: связь с саркопенией и деменцией (обзор литературы) [J]. Klinicheskaia laboratornaia diagnostika, 2021, 66(1): 5-9.  [127] BULGAKOVA S V, TRENEVA E V, ZAKHAROVA N O. Hypovitaminosis D in the elderly: relationship with sarcopenia and dementia (review of literature) [J]. Klin Lab Diagn, 2021, 66(1): 5-9.  [128] BULGAKOVA S V, TRENEVA E V, ZAKHAROVA N O, et al. Intestinal microbiota: relationship to age-associated diseases (review of literature) [J]. Klinicheskaia laboratornaia diagnostika, 2019, 64(4): 250-6.  [129] BULUT E A, SOYSAL P, AYDIN A E, et al. Vitamin B12 deficiency might be related to sarcopenia in older adults [J]. European Geriatric Medicine, 2018, 9: S87.  [130] BURTON D A, NICHOLSON G, HALL G M. Anaesthesia in elderly patients with neurodegenerative disorders - Special considerations [J]. Drugs & Aging, 2004, 21(4): 229-42.  [131] BUSH B, LLOYD J, DERUISSEAU K, et al. Alzheimer's disease is associated with muscle weakness [J]. FASEB Journal, 2012, 26.  [132] CABALLERO-ÁVILA M, IKENAGA C, MARINI-BETTOLO C, et al. Description of clinicalsymptoms of a large international cohort of patients with valosincontaining-protein related disease [J]. Neurology, 2021, 96(15 SUPPL 1).  [133] CADORE E L, DE ASTEASU M L S, IZQUIERDO M. Multicomponent exercise and the hallmarks of frailty: Considerations on cognitive impairment and acute hospitalization [J]. Experimental Gerontology, 2019, 122: 10-4.  [134] CAFFORIO G, PISTOLESI S, D'AVINO C, et al. Inclusion body myopathy associated with motor neuron syndrome: three case reports [J]. Clin Neuropathol, 2005, 24(1): 36-41.  [135] CAMERO S, BENíTEZ M J, JIMéNEZ J S. Anomalous protein-DNA interactions behind neurological disorders [J]. Adv Protein Chem Struct Biol, 2013, 91: 37-63.  [136] CAMERO S, BENíTEZ M J, JIMéNEZ J S. Anomalous Protein-DNA Interactions Behind Neurological Disorders [M]//DONEV R. Protein-Nucleic Acids Interactions. 2013: 37-63.  [137] CAMPAGNE S. U1 snRNP Biogenesis Defects in Neurodegenerative Diseases [J]. Chembiochem, 2024, 25(9): e202300864.  [138] CAMPOS E M C, DE ABREU F A, HAYAKAVA L A, et al. NUTRITION AND ALZHEIMER'S DISEASE: A BRIEF REVIEW [J]. Revista Univap, 2020, 26(50): 130-43.  [139] CARLILE C, SCHULTZ J, REES A, et al. 260. Comparing outcomes and mortality of upper cervical and subaxial cervical spine trauma in elderly patients [J]. Spine Journal, 2021, 21(9): S134.  [140] CARTER C S, HOFER T, SEO A Y, et al. Molecular mechanisms of life- And health-span extension: Role of calorie restriction and exercise intervention [J]. Applied Physiology, Nutrition and Metabolism, 2007, 32(5): 954-66.  [141] CASTEN K S. Associations of weakness and inflammation with disability, poor cognition, and multimorbidity in older adults [J]. PM and R, 2019, 11: S104-S5.  [142] CASTILLO MARIQUEO L, GIMENEZ-LLORT L. Gait impairments and functional limitations in the exploratory activity in an animal model of Alzheimer's disease [J]. European Journal of Neurology, 2021, 28(SUPPL 1): 446.  [143] CASULA M, STEENTJES K, ARONICA E, et al. Concomitant CNS pathology in a patient with amyotropic lateral sclerosis following poliomyelitis in childhood [J]. Clin Neuropathol, 2011, 30(3): 111-7.  [144] CAUCHI R J, VAN DEN HEUVEL M. The fly as a model for neurodegenerative diseases: Is it worth the jump? [J]. Neurodegenerative Diseases, 2006, 3(6): 338-56.  [145] CAVINESS J N. Myoclonus and neurodegenerative disease - what's in a name? [J]. Parkinsonism & Related Disorders, 2003, 9(4): 185-92.  [146] CEDA G P, DALL'AGLIO E, MAGGIO M, et al. Clinical implications of the reduced activity of the GH-IGF-I axis in older men [J]. Journal of endocrinological investigation, 2005, 28(11 Suppl Proceedings): 96-100.  [147] CENINI G, LLORET A, CASCELLA R. Oxidative Stress in Neurodegenerative Diseases: From a Mitochondrial Point of View [J]. Oxidative Medicine and Cellular Longevity, 2019, 2019.  [148] CHAE J, SUNG J. A Case of VCP Mutation Featuring With Lobulated Myofiber, Motor Neuron Disease and Frontotemporal Dementia [J]. Journal of Neuromuscular Diseases, 2022, 9: S257.  [149] CHANDRASEKHAR A, SCHLACHETZKI Z, COFFEY A, et al. eP329: Genome sequencing uncovers molecular cause in a case with epileptic encephalopathy [J]. Genetics in Medicine, 2022, 24(3): S206.  [150] CHANG K V, HSU T H, WU W T, et al. Association Between Sarcopenia and Cognitive Impairment: A Systematic Review and Meta-Analysis [J]. J Am Med Dir Assoc, 2016, 17(12): 1164.e7-.e15.  [151] CHANG M C, LEE A Y, KWAK S, et al. Effect of Resistance Exercise on Depression in Mild Alzheimer Disease Patients With Sarcopenia [J]. American Journal of Geriatric Psychiatry, 2020, 28(5): 587-9.  [152] CHANG X, WANG J, JIANG H, et al. Hyperpolarization-activated cyclic nucleotide-gated channels: An emerging role in neurodegenerative diseases [J]. Frontiers in Molecular Neuroscience, 2019, 12.  [153] CHARLTON K E. Eating well: ageing gracefully! [J]. Asia Pacific journal of clinical nutrition, 2002, 11 Suppl 3: S607-S17.  [154] CHAVDA V, CHAURASIA B, UMANA G E, et al. Narcolepsy-A Neuropathological Obscure Sleep Disorder: A Narrative Review of Current Literature [J]. Brain Sciences, 2022, 12(11).  [155] CHEN L, XU Y, FANG M J, et al. Case report: A Chinese patient with spinocerebellar ataxia finally confirmed as Gerstmann-Sträussler-Scheinker syndrome with P102L mutation [J]. Front Neurol, 2023, 14: 1187813.  [156] CHEN X, HE E, SU C, et al. Huntingtin-associated protein 1-associated intracellular trafficking in neurodegenerative diseases [J]. Frontiers in Aging Neuroscience, 2023, 15.  [157] CHEN Y P, KUO Y J. The high prevalence of sarcopenia and its associated outcomes following hip surgery in taiwanese geriatric patients with a hip fracture [J]. Osteoporosis International, 2020, 31(SUPPL 1): S213.  [158] CHERIN P. Treatment of inclusion body myositis [J]. Current Opinion in Rheumatology, 1999, 11(6): 456-61.  [159] CHIARIELLO A, CONTE M, MEDICI V, et al. EXPRESSION PATTERN OF THE MITOKINE GDF15 IN HUMAN BRAIN IN HEALTHY AGING AND IN ALZHEIMER'S DISEASE [J]. Journal of Nutrition, Health and Aging, 2022, 26(4): 475-6.  [160] CHIN A, LéCUYER E. RNA localization: Making its way to the center stage [J]. Biochimica et Biophysica Acta - General Subjects, 2017, 1861(11): 2956-70.  [161] CHING J K, WEIHL C C. Rapamycin-induced autophagy aggravates pathology and weakness in a mouse model of VCP-associated myopathy [J]. Autophagy, 2013, 9(5): 799-800.  [162] CHRISTIANSEN A R, LIPSHULTZ L I, HOTALING J M, et al. Selective androgen receptor modulators: The future of androgen therapy? [J]. Translational Andrology and Urology, 2020, 9: S135-S48.  [163] CIARAMBINO T, CRISPINO P, MINERVINI G, et al. COVID-19 and Frailty [J]. Vaccines, 2023, 11(3).  [164] ÇINAR Z, MACIT AYDIN E, NAVRUZVAI N, et al. Impact of neurological problems on icu outcome in pulmonary icu patients [J]. Intensive Care Medicine Experimental, 2020, 8(SUPPL 2).  [165] CLARKE J P, THIBAULT P A, SALAPA H E, et al. A Comprehensive Analysis of the Role of hnRNP A1 Function and Dysfunction in the Pathogenesis of Neurodegenerative Disease [J]. Front Mol Biosci, 2021, 8: 659610.  [166] CLEVELAND D. Mechanism and therapy in ALS/FTD and beyond [J]. Neurodegenerative Diseases, 2015, 15: 258.  [167] CLEVELAND D. Gene regulation [J]. Journal of the Neurological Sciences, 2017, 381: 31.  [168] CLEVELAND D. Gene silencing therapy for human neurodegenerative disease [J]. Neurodegenerative Diseases, 2017, 17: 2.  [169] CLEVELAND D. Designer DNA drug therapy for human neurodegenerative disease [J]. Amyotrophic Lateral Sclerosis and Frontotemporal Degeneration, 2019, 20: 24.  [170] COELHO-JúNIOR H J, TRICHOPOULOU A, PANZA F. Cross-sectional and longitudinal associations between adherence to Mediterranean diet with physical performance and cognitive function in older adults: A systematic review and meta-analysis [J]. Ageing Res Rev, 2021, 70: 101395.  [171] CONCEIçãO E F M, OLIVEIRA A S, MOURA M A O, et al. Cerebral amyloid angiopathy in capuchin monkey (Sapajus sp.) [J]. Brazilian Journal of Veterinary Pathology, 2020, 13(1): 495.  [172] CONFORTI L, ADALBERT R, COLEMAN M P. Neuronal death: where does the end begin? [J]. Trends in Neurosciences, 2007, 30(4): 159-66.  [173] CONTE M, MEDICI V, CHIARIELLO A, et al. DIFFERENTIAL EXPRESSION OF PERILIPIN FAMILY PROTEINS IN HUMAN BRAIN DURING AGING AND ALZHEIMER'S DISEASE [J]. Journal of Nutrition, Health and Aging, 2022, 26(4): 474-5.  [174] COOPER-KNOCK J, SHAW P J, KIRBY J. The widening spectrum of C9ORF72-related disease; Genotype/phenotype correlations and potential modifiers of clinical phenotype [J]. Acta Neuropathologica, 2014, 127(3): 333-45.  [175] COOPER-KNOCK J, SHAW P J, KIRBY J. The widening spectrum of <i>C9ORF72</i>-related disease; genotype/phenotype correlations and potential modifiers of clinical phenotype [J]. Acta Neuropathologica, 2014, 127(3): 333-45.  [176] CóRCOLES R A, JIMéNEZ E G, CéSPEDES A A, et al. Clinical guidelines and best practices to improve the management of elderly patients with dementia and multimorbility. a systematic review [J]. European Geriatric Medicine, 2022, 13: S14-S5.  [177] CORTES C J, LA SPADA A R. TFEB dysregulation as a driver of autophagy dysfunction in neurodegenerative disease: Molecular mechanisms, cellular processes, and emerging therapeutic opportunities [J]. Neurobiology of Disease, 2019, 122: 83-93.  [178] CORTéS MANCERA E A, SINISTERRA SOLIS F A, ROMERO-CASTELLANOS F R, et al. (18)F-FDG PET/CT as a molecular biomarker in the diagnosis of amyotrophic lateral sclerosis associated with prostate cancer and progressive supranuclear palsy: A case report [J]. Front Nucl Med, 2023, 3: 1137875.  [179] COSKUNER-WEBER O, MIRZANLI O, UVERSKY V N. Intrinsically disordered proteins and proteins with intrinsically disordered regions in neurodegenerative diseases [J]. Biophysical Reviews, 2022, 14(3): 679-707.  [180] COSTA C J, WILLIS D E. To the end of the line: Axonal mRNA transport and local translation in health and neurodegenerative disease [J]. Developmental Neurobiology, 2018, 78(3): 209-20.  [181] COSTERUS J M, BROUWER M C, VAN DE BEEK D. Technological advances and changing indications for lumbar puncture in neurological disorders [J]. The Lancet Neurology, 2018, 17(3): 268-78.  [182] CRISTOFANI R, CRIPPA V, CICARDI M E, et al. BAG1 prevents misfolded proteins accumulation when autophagy flux is blocked in neurodegenerative disorders [J]. Journal of Neurochemistry, 2017, 142: 65.  [183] CUARTAS J, GANGWANI L. R-loop Mediated DNA Damage and Impaired DNA Repair in Spinal Muscular Atrophy [J]. Frontiers in Cellular Neuroscience, 2022, 16.  [184] CUESTA-TRIANA F, VERDEJO-BRAVO C, FERNáNDEZ-PéREZ C, et al. Effect of Milk and Other Dairy Products on the Risk of Frailty, Sarcopenia, and Cognitive Performance Decline in the Elderly: A Systematic Review [J]. Advances in Nutrition, 2019, 10: S105-S19.  [185] CULBERSON J W. Clinical Aspects of Glucose Metabolism and Chronic Disease [J]. Prog Mol Biol Transl Sci, 2017, 146: 1-11.  [186] CULETTO E, SATTELLE D B. A role for Caenorhabditis elegans in understanding the function and interactions of human disease genes [J]. Hum Mol Genet, 2000, 9(6): 869-77.  [187] CURINHA A, OLIVEIRA BRAZ S, PEREIRA-CASTRO I, et al. Implications of polyadenylation in health and disease [J]. Nucleus, 2014, 5(6): 508-19.  [188] D’AMELIO P, QUACQUARELLI L. Hypovitaminosis d and aging: Is there a role in muscle and brain health? [J]. Nutrients, 2020, 12(3).  [189] DALLE S, ROSSMEISLOVA L, KOPPO K. The role of inflammation in age-related sarcopenia [J]. Frontiers in Physiology, 2017, 8(DEC).  [190] DAMULEVIČIENE G, ENČERYTE I, KNAŠIENE J. Prevalence and associated factors of dysphagia among geriatric in-patients at Kaunas clinical hospital, Lithuania [J]. European Geriatric Medicine, 2016, 7: S92.  [191] DANG J X. Clinical manifestations and advances in diagnosis and treatment of amyotrophic lateral sclerosis [J]. Journal of Xi'an Jiaotong University (Medical Sciences), 2018, 39(5): 613-9.  [192] DASARATHY J, ROGERS K, RAJESH R. SARCOPENIA, SARCOPENIC OBESITY AND FRAILTY; LINKS TO COGNITIVE PERFORMANCE IN ELDERS: Session 412 [J]. American Journal of Geriatric Psychiatry, 2019, 27(3): S46-S7.  [193] DAVIDSON A, LONGMAN C, FARRUGIA M. When your heart is aflutter and you're weak at the knees: A case report [J]. Journal of Neurology, Neurosurgery and Psychiatry, 2013, 84(11).  [194] DAYANGAC-ERDEN D, ESKICI N, ERDEM-OZDAMAR S. Altered expression of perineuronal net elements in SMN knockdown cells [J]. European Journal of Human Genetics, 2019, 26: 622.  [195] DE CAMPOS CALASSARA P, DIAS A L N, GOMES P S C, et al. Comparison between the 2010 and 2018 EWGOP sarcopenia criteria in elderly with type 2 diabetes [J]. Diabetology and Metabolic Syndrome, 2019, 11.  [196] DE CARVALHO M. Motor neuron diseases [J]. Clinical Neurophysiology, 2011, 122: S37.  [197] DE CARVALHO M, EISEN A, KRIEGER C, et al. Motoneuron firing in amyotrophic lateral sclerosis (ALS) [J]. Frontiers in Human Neuroscience, 2014, 8.  [198] DE COCK A M. FraMUGA Project: Combining muscle ultrasound and gait analysis in the early detection of frailty [J]. European Geriatric Medicine, 2019, 10: S263-S4.  [199] DE LA HERRáN-ARITA A K, GARCíA-GARCíA F. Current and Emerging Options for the Drug Treatment of Narcolepsy [J]. Drugs, 2013, 73(16): 1771-81.  [200] DE LA MONTE S M, LU B X, SOHN Y K, et al. Aberrant expression of nitric oxide synthase III in Alzheimer's disease: relevance to cerebral vasculopathy and neurodegeneration [J]. Neurobiology of Aging, 2000, 21(2): 309-19.  [201] DE LA TORRE J C. Cerebral Hemodynamics and Vascular Risk Factors: Setting the Stage for Alzheimer's Disease [J]. Journal of Alzheimers Disease, 2012, 32(3): 553-67.  [202] DE MARCHI F, BERSANO E, SARNELLI M F, et al. Obsessive Compulsive Disorder as the initial presentation of motor neuron disease/frontotemporal dementia: A case report [J]. European Journal of Neurology, 2016, 23: 387-8.  [203] DE SOUSA O V, DO AMARAL T F. Factors associated with mild Alzheimer's disease among community-dwelling older adults [J]. Sinapse, 2016, 16(2): 162.  [204] DE SOUSA O V, DO AMARAL T F. Sarcopenia, vitamin D, and functional status in mild Alzheimer's disease and community-dwelling older adults [J]. Journal of Cachexia, Sarcopenia and Muscle, 2017, 8(1): 163.  [205] DE TOMMASO M, ARENDT-NIELSEN L, DEFRIN R, et al. Pain in Neurodegenerative Disease: Current Knowledge and Future Perspectives [J]. Behavioural Neurology, 2016, 2016.  [206] DEARDORFF W J, KOEHLER R M, NIEHOFF M, et al. The antidiabetic drug metformin improves learning and memory in streptozotocin-induced diabetic cd1 mice [J]. Journal of the American Geriatrics Society, 2015, 63: S146.  [207] DEJESUS-HERNANDEZ M, VAN BLITTERSWIJK M, BROWN P, et al. Somatic heterogeneity of the ggggcc hexanucleotide repeat in C9ORF72 expanded repeat carriers [J]. Amyotrophic Lateral Sclerosis, 2012, 13: 16-7.  [208] DELL' AMICO C, TATA A, PELLEGRINO E, et al. Genome editing in stem cells for genetic neurodisorders [Z]. 2021: 403-38.10.1016/bs.pmbts.2020.12.006  [209] DENG W P, YANG Z, HUANG X J, et al. Case Report: Neuronal Intranuclear Inclusion Disease With Oromandibular Dystonia Onset [J]. Front Neurol, 2021, 12: 618595.  [210] DESCHENES M R, FLANNERY R, HAWBAKER A, et al. Adaptive Remodeling of the Neuromuscular Junction with Aging [J]. Cells, 2022, 11(7).  [211] DESLANDES A. The biological clock keeps ticking, but exercise may turn it back [J]. Arquivos De Neuro-Psiquiatria, 2013, 71(2): 113-8.  [212] DETIENNE S, VANDEWOUDE M, SUY R. Nutritional profile and body composition in geriatric rehabilitation after hip surgery [J]. European Geriatric Medicine, 2010, 1: S23-S4.  [213] DEUSCHL G, HERZOG J, KLEINER-FISMAN G, et al. Deep brain stimulation:: Postoperative issues [J]. Movement Disorders, 2006, 21: S219-S37.  [214] DEVI S, YADAV R, CHANANA P, et al. Fighting the Cause of Alzheimer's and GNE Myopathy [J]. Frontiers in Neuroscience, 2018, 12.  [215] DHALIWAL R, ADLER R, LEE R, et al. Abstract #1407491: Comorbidities and Characteristics of Men with Subsequent Osteoporotic Fracture: A Real-world Observational Study [J]. Endocrine Practice, 2023, 29(5): S56.  [216] DHANANJAYAN R. Biochemistry of Vitamin B12 and active Vitamin B12 [J]. Indian Journal of Clinical Biochemistry, 2018, 33: S21.  [217] DIBELLO V, LOZUPONE M, MANFREDINI D, et al. Oral frailty and neurodegeneration in Alzheimer's disease [J]. Neural Regeneration Research, 2021, 16(11): 2149-53.  [218] DICKSON D. Frequency of primary lateral sclerosis (PLS), an uncommon form of motor neuron disease, in a brain bank for neurodegenerative diseases [J]. Journal of Neuropathology and Experimental Neurology, 2020, 79(6): 654.  [219] DICKSON D W. Motor neuron disease pathology presenting as progressive supranuclear palsy [J]. Movement Disorders, 2013, 28: S293.  [220] DIJKSTRA F, VAN DEN BOSSCHE K, DE BRUYN B, et al. REM sleep without atonia and the relation with Lewy body disease [J]. Parkinsonism & Related Disorders, 2019, 67: 90-8.  [221] DINDA B, DINDA M, KULSI G, et al. Therapeutic potentials of plant iridoids in Alzheimer's and Parkinson's diseases: A review [J]. European Journal of Medicinal Chemistry, 2019, 169: 185-99.  [222] DOHERTY M J, BIRD T D, LEVERENZ J B. α-Synuclein in motor neuron disease: An immunohistologic study [J]. Acta Neuropathologica, 2004, 107(2): 169-75.  [223] DOHRN M F, MEDINA J, OLACIREGUI DAGUE K R, et al. Are we creating a new phenotype? Physiological barriers and ethical considerations in the treatment of hereditary transthyretin-amyloidosis [J]. Neurological Research and Practice, 2021, 3(1).  [224] DOS SANTOS J C C, MANO G B C, BARRETO-VIANNA A R D, et al. The Molecular Impact of <i>Glucosylceramidase Beta 1</i> (<i>Gba1</i>) in Parkinson's Disease: a New Genetic State of the Art [J]. Molecular Neurobiology, 2024, 61(9): 6754-70.  [225] DREY M, KAISER M J. Malnutrition in the elderly [J]. Deutsche Medizinische Wochenschrift, 2011, 136(5): 176-8.  [226] DUESBERG P, RASNICK D. The AIDS dilemma: drug diseases blamed on a passenger virus [J]. Genetica, 1998, 104(2): 85-132.  [227] DWORSKI S, JONES E E, SIKORA J, et al. Spatial distribution of brain ceramides in an acid ceramidase deficient murine model: Subsequent histological manifestations and functional deficits [J]. Molecular Genetics and Metabolism, 2015, 114(2): S38-S9.  [228] EBINA J, EBIHARA S, KANO O. Similarities, differences and overlaps between frailty and Parkinson's disease [J]. Geriatrics and Gerontology International, 2022, 22(4): 259-70.  [229] EDWARDS B, HOLMES H, SUN M, et al. Osteoporosis, low bone mass, and risk factors for fracturesin older cancer patients [J]. Supportive Care in Cancer, 2016, 24(1): S44.  [230] EGAWA T, HAYASHI T. Association of Glycative Stress With Motor and Muscle Function [J]. Frontiers in Physiology, 2022, 13.  [231] EISEN A. Amyotrophic lateral sclerosis (ALS/MND) clinical aspects [J]. Clinical Neurophysiology, 2010, 121: S51.  [232] EKSHYYAN O, AW T Y. Apoptosis: a key in neurodegenerative disorders [J]. Current neurovascular research, 2004, 1(4): 355-71.  [233] EKSHYYAN O, AW T Y. Apoptosis in acute and chronic neurological disorders [J]. Frontiers in bioscience : a journal and virtual library, 2004, 9: 1567-76.  [234] EL-HATTAB A W, ALMANNAI M, SCAGLIA F. MELAS [M]//ADAM M P, FELDMAN J, MIRZAA G M, et al. GeneReviews(®). Seattle (WA); University of Washington, Seattle  Copyright © 1993-2024, University of Washington, Seattle. GeneReviews is a registered trademark of the University of Washington, Seattle. All rights reserved. 1993.  [235] ENDURI S, TAYLOR M R G, LIEWLUCK T. Clinical Reasoning: A 52-year-old woman with progressive proximal weakness [J]. Neurology, 2014, 83(10): E106-E9.  [236] ENG J, FABRIZI C, NORTON L. Dysphagia as presentation of amyotrophic lateral sclerosis [J]. Journal of the American Geriatrics Society, 2011, 59: S25-S6.  [237] ENG J R, DUGGAN S, TANZOLA R, et al. Neostigmine-induced cholinergic crisis: A case report [J]. Canadian Journal of Anesthesia, 2017, 64(1): S264-S5.  [238] ERZURUMLU Y. p97/VCP and Inclusion Body Myopathy with Early-Onset Paget Disease and Frontotemporal Dementia (IBMPFD) [J]. Cyprus Journal of Medical Sciences, 2021, 6(4): 337-44.  [239] ESPEJO-PORRAS F, PISCITELLI F, VERDE R, et al. Changes in the endocannabinoid signaling system in CNS structures of TDP-43 transgenic mice: relevance for a neuroprotective therapy in TDP-43-related disorders [J]. Journal of Neuroimmune Pharmacology, 2015, 10(2): 233-44.  [240] ESPINOZA GUTIéRREZ R, ORTIZ ORTIZ M, GOMEZ MIRANDA L M, et al. Physical, anthropometric and nutritional status in older adults by sex of the US-Mexico border [J]. Obesity Facts, 2018, 11: 316-7.  [241] ETTCHETO M, OLLOQUEQUI J, SáNCHEZ-LóPEZ E, et al. Benzodiazepines and Related Drugs as a Risk Factor in Alzheimer's Disease Dementia [J]. Frontiers in Aging Neuroscience, 2020, 11.  [242] EVENS J, WILLEKENS S M A, VANWEEHAEGHE D, et al. Relationship between brain metabolism and cognitive/behavioral functioning in ALS-FTD [J]. European Journal of Nuclear Medicine and Molecular Imaging, 2016, 43(1): S133-S4.  [243] EVGENEVA E, SALAS FELIPE J, SORIANO NAVARRO M, et al. 4R Tauopathy with cognitive and motor impairment, not associated with Alzheimer Disease [J]. Virchows Archiv, 2013, 463(2): 234.  [244] FARSHIDFAR F, SUH M, SNOW W, et al. Creatine supplementation increases muscle branched-chain amino acids in an alzheimer mouse model [J]. FASEB Journal, 2015, 29(1).  [245] FATIMA K, MEHENDALE A M, REDDY H. Young-Onset Dementia and Neurodegenerative Disorders of the Young With an Emphasis on Clinical Manifestations [J]. Cureus, 2022, 14(10): e30025.  [246] FAUCHER J, DUBEAU C. Genetics and new onset leg weakness [J]. Journal of the American Geriatrics Society, 2013, 61: S79.  [247] FDEZ-MONTALBáN P, ALBéNIZ J, HERNáNDEZ L, et al. Prevalence of sarcopenia in very old patients hospitalized in a geriatric ward [J]. European Geriatric Medicine, 2017, 8: S109.  [248] FEELY S, GONZALEZ M, WEIHL C, et al. Novel mutation in VCP causes charcot-marie-tooth type 2 (CMT2) phenotype [J]. Neurology, 2014, 82(10).  [249] FEELY S, GONZALEZ M, WEIHL C, et al. Novel mutation in VCP causes charcot-marie tooth type 2 (CMT2) phenotype [J]. Neurology, 2014, 82(10).  [250] FEKI A, HIBAOUI Y. DYRK1A protein, a promising therapeutic target to improve cognitive deficits in down syndrome [J]. Brain Sciences, 2018, 8(10).  [251] FELLGIEBEL A, SCHEURICH A, SIESSMEIER T, et al. Persistence of disturbed thalamic glucose metabolism in a case of Wernicke-Korsakoff syndrome [J]. Psychiatry Res, 2003, 124(2): 105-12.  [252] FERENTINOS P, PAPARRIGOPOULOS T, RENTZOS M, et al. Duloxetine for pathological laughing and crying in amyotrophic lateral sclerosis [J]. European Neuropsychopharmacology, 2009, 19: S409.  [253] FERNáNDEZ-NOGALES M, CABRERA J R, SANTOS-GALINDO M, et al. Huntington's disease as a tauopathy [J]. Journal of Neurology, Neurosurgery and Psychiatry, 2014, 85: A17.  [254] FERRUCCI L, FABBRI E. Inflammageing: chronic inflammation in ageing, cardiovascular disease, and frailty [J]. Nature Reviews Cardiology, 2018, 15(9): 505-22.  [255] FINAN C, PARK K, ELWOOD H, et al. 44124 Nonspecific cutaneous eruption in a patient with statin-associated autoimmune myopathy [J]. Journal of the American Academy of Dermatology, 2023, 89(3): AB68.  [256] FINSTERER J. Neuropathy, Ataxia, and Retinitis Pigmentosa Syndrome [J]. Journal of Clinical Neuromuscular Disease, 2023, 24(3): 140-6.  [257] FLISIKOWSKA T, KIND A, SCHNIEKE A. Genetically modified pigs to model human diseases [J]. Journal of Applied Genetics, 2014, 55(1): 53-64.  [258] FOUGèRE B, BOULANGER E, NOURHASHéMI F, et al. Chronic Inflammation: Accelerator of Biological Aging [J]. The journals of gerontology Series A, Biological sciences and medical sciences, 2017, 72(9): 1218-25.  [259] FOUGèRE B, BOULANGER E, NOURHASHéMI F, et al. RETRACTED: Chronic Inflammation: Accelerator of Biological Aging (Retracted Article) [J]. Journals of Gerontology Series a-Biological Sciences and Medical Sciences, 2017, 72(9): 1218-25.  [260] FRANCESCHI C, GARAGNANI P, MORSIANI C, et al. The Continuum of Aging and Age-Related Diseases: Common Mechanisms but Different Rates [J]. Front Med (Lausanne), 2018, 5: 61.  [261] FRANZKE B, NEUBAUER O, WAGNER K H. Super DNAging-New insights into DNA integrity, genome stability and telomeres in the oldest old [J]. Mutation Research-Reviews in Mutation Research, 2015, 766: 48-57.  [262] FRANZON K, SOBESTIANSKY S, BYBERG L, et al. Is sarcopenia associated with independent ageing? A report from the Uppsala Longitudinal Study of Adult Men (ULSAM) [J]. European Geriatric Medicine, 2016, 7: S8.  [263] FRANZON K, ZETHELIUS B, CEDERHOLM T, et al. Muscle function, muscle mass and sarcopenia and its relation to independent ageing. A report from the Uppsala Longitudinal Study of Adult Men (ULSAM) [J]. European Geriatric Medicine, 2018, 9: S11.  [264] FREDERICK M C, WOLTJER R, SILBERT L, et al. Dementia syndrome in oculopharyngeal muscular dystrophy [J]. Annals of Neurology, 2014, 76: S91-S2.  [265] FREDERICK M C, WOLTJER R, SILBERT L C, et al. Dementia syndromein oculopharyngeal muscular dystrophy: A trinucleotide expansion disorder commonly presenting with muscle weakness [J]. Alzheimer's and Dementia, 2014, 10: P682-P3.  [266] FRIEDMANN T. Gene therapy for spinomuscular atrophy: A biomedical advance, a missed opportunity for more equitable drug pricing [J]. Gene Therapy, 2017, 24(9): 503-5.  [267] FRITZ N E, MCCARTHY C J, ADAMO D E. Handgrip strength as a means of monitoring progression of cognitive decline – A scoping review [J]. Ageing Research Reviews, 2017, 35: 112-23.  [268] FRITZ N E, MCCARTHY C J, ADAMO D E. Handgrip strength as a means of monitoring progression of cognitive decline - A scoping review [J]. Ageing Res Rev, 2017, 35: 112-23.  [269] FRöSEN J, JOUTEL A. Smooth muscle cells of intracranial vessels: from development to disease [J]. Cardiovascular Research, 2018, 114(4): 501-12.  [270] FUJIHARA Y, TSUCHIDA K, HIROKI J, et al. Significance of sarcopenia evaluation in acute decompensated heart failure: Skeletal muscle mass index versus fat-free mass index [J]. European Heart Journal, 2016, 37: 1115-6.  [271] FUJIMAKI S, WAKABAYASHI T, TAKEMASA T, et al. Diabetes and stem cell function [J]. Biomed Res Int, 2015, 2015: 592915.  [272] FUJITA M, UENO T, MIKI Y, et al. Case report: Adult-onset neuronal intranuclear inclusion disease with an amyotrophic lateral sclerosis phenotype [J]. Front Neurosci, 2022, 16: 960680.  [273] FUJIWARA S. Hip Fracture--Epidemiology, Management and Liaison Service. Risk factor for hip fracture [J]. Clinical calcium, 2015, 25(4): 499-504.  [274] FUKUMURA N, MAKIGAMI K. Oral Intake in the Complete Lateral Position as a Compensatory Method for a Patient with Severe Dysphagia: A Case Report [J]. Prog Rehabil Med, 2024, 9: 20240008.  [275] GAGNON J F, POSTUMA R B, MAZZA S, et al. Rapid-eye-movement sleep behaviour disorder and neurodegenerative diseases [J]. Lancet Neurology, 2006, 5(5): 424-32.  [276] GAIG C, IRANZO A. Sleep-Disordered Breathing in Neurodegenerative Diseases [J]. Current Neurology and Neuroscience Reports, 2012, 12(2): 205-17.  [277] GALBIATI A, CARLI G, HENSLEY M, et al. REM Sleep Behavior Disorder and Alzheimer's Disease: Definitely No Relationship? [J]. Journal of Alzheimers Disease, 2018, 63(1): 1-11.  [278] GALIL A, SCHIFFMANN R, NEEMAN Z, et al. [Infantile neuroaxonal dystrophy] [J]. Harefuah, 1992, 123(10): 387-90, 435.  [279] GALVIN J E, TOLEA M I, CHRISPHONTE S, et al. Obstructive airway disease and the risk of cognitive impairment in a multicultural community of older adults [J]. Alzheimer's and Dementia, 2016, 12(7): P1074-P5.  [280] GAMBOA-ESPARZA M, ANDRADE-AGUILAR A M, MORENO-PéREZ A, et al. Heart disease in people older than 95 years-the Cardiogeriatric Clinic experience in Mexico [J]. European Geriatric Medicine, 2022, 13: S433.  [281] GANAI S A. Small-molecule Modulation of HDAC6 activity: The propitious therapeutic strategy to vanquish neurodegenerative disorders [J]. Current Medicinal Chemistry, 2017, 24(37): 4104-20.  [282] GARAY-SEVILLA M E, BEERI M S, DE LA MAZA M P, et al. The potential role of dietary advanced glycation endproducts in the development of chronic non-infectious diseases: a narrative review [J]. Nutr Res Rev, 2020, 33(2): 298-311.  [283] GARCíA-LLORENTE A M, CASIMIRO-ANDúJAR A J, LINHARES D G, et al. Multidomain interventions for sarcopenia and cognitive flexibility in older adults for promoting healthy aging: a systematic review and meta-analysis of randomized controlled trials [J]. Aging Clinical and Experimental Research, 2024, 36(1).  [284] GARCíA-RUANO C, COSTA A, BARGAY E, et al. Cognitive decline is associated with low muscle mass and gait speed and with decreased BMI and adiposity in a pilot senior cohort study [J]. Obesity Facts, 2023, 16: 78.  [285] GAVRIILAKI M, KIMISKIDIS V K, GAVRIILAKI E. Precision medicine in neurology: The inspirational paradigm of complement therapeutics [J]. Pharmaceuticals, 2020, 13(11): 1-25.  [286] GAYTAN-GARCIA S, KAUFMANN J C, YOUNG G B. Adult onset Hallervorden-Spatz syndrome or Seitelberger's disease with late onset: variants of the same entity? A clinico-pathological study [J]. Clin Neuropathol, 1990, 9(3): 136-42.  [287] GEBAI A, NAGAR B, GORELIK A, et al. Crystal structure of acid ceramidase [J]. Protein Science, 2017, 26: 196.  [288] GEEVASINGA N, VAN DEN BOS M, MENON P, et al. Utility of transcranial magnetic simulation in studying upper motor neuron dysfunction in amyotrophic lateral sclerosis [J]. Brain Sciences, 2021, 11(7).  [289] GELON P A, DUTCHAK P A, SEPHTON C F. Synaptic dysfunction in ALS and FTD: anatomical and molecular changes provide insights into mechanisms of disease [J]. Frontiers in Molecular Neuroscience, 2022, 15.  [290] GEMIKONAKLI G, MACH J, HILMER S N. Interactions Between the Aging Gut Microbiome and Common Geriatric Giants: Polypharmacy, Frailty, and Dementia [J]. The journals of gerontology Series A, Biological sciences and medical sciences, 2021, 76(6): 1019-28.  [291] GENDRON T F, PETRUCELLI L. Disease Mechanisms of C9ORF72 Repeat Expansions [J]. Cold Spring Harb Perspect Med, 2018, 8(4).  [292] GEORGOULAS P, KARADIMOU D, DROSOS G. The necessity of multidisciplinary approach in fragility fractures [J]. Journal of Musculoskeletal Neuronal Interactions, 2021, 21(1): 172-3.  [293] GHIKA J. Paleoneurology: Neurodegenerative diseases are age-related diseases of specific brain regions recently developed by homo sapiens [J]. Medical Hypotheses, 2008, 71(5): 788-801.  [294] GHZAIEL I, MAALOUL S, KSILA M, et al. In Vitro Evaluation of the Effects of 7-Ketocholesterol and 7β-Hydroxycholesterol on the Peroxisomal Status: Prevention of Peroxisomal Damages and Concept of Pexotherapy [Z]. 2024: 437-52.10.1007/978-3-031-43883-7_21  [295] GIANFORCARO A, HAMADEH M J. Vitamin D as a Potential Therapy in Amyotrophic Lateral Sclerosis [J]. Cns Neuroscience & Therapeutics, 2014, 20(2): 101-11.  [296] GILLETTE GUYONNET S, ABELLAN VAN KAN G, ALIX E, et al. IANA (International Academy on Nutrition and Aging) Expert Group: weight loss and Alzheimer's disease [J]. J Nutr Health Aging, 2007, 11(1): 38-48.  [297] GLADE M J. Vitamin D: Health panacea or false prophet? [J]. Nutrition, 2013, 29(1): 37-41.  [298] GOLDBERG E L, DIXIT V D. Drivers of age-related inflammation and strategies for healthspan extension [J]. Immunol Rev, 2015, 265(1): 63-74.  [299] GOLDSTEIN A, FALK M J. Single Large-Scale Mitochondrial DNA Deletion Syndromes [M]//ADAM M P, FELDMAN J, MIRZAA G M, et al. GeneReviews(®). Seattle (WA); University of Washington, Seattle  Copyright © 1993-2024, University of Washington, Seattle. GeneReviews is a registered trademark of the University of Washington, Seattle. All rights reserved. 1993.  [300] GOLDSTEIN R, SINGH J, ISRAEL Y, et al. Villaret's syndrome from skull base aspergillosis in a diabetic man: A case report [J]. Journal of Hospital Medicine, 2011, 6(4): S185.  [301] GOLUBEV A G. Carving the senescent phenotype by the chemical reactivity of catecholamines: An integrative review [J]. Ageing Research Reviews, 2022, 75.  [302] GOMBASH LAMPE S E, COWLEY C J, CHRISTOFI F L, et al. Enteric nervous system transduction following intravascular delivery of AAV9 [J]. Molecular Therapy, 2014, 22: S91.  [303] GOMEZ LIMIA C, BAIRD M, SCHWARTZ M, et al. Emerging Perspectives on Gene Therapy Delivery for Neurodegenerative and Neuromuscular Disorders [J]. Journal of Personalized Medicine, 2022, 12(12).  [304] GóMEZ-GóMEZ M E, ZAPICO S C. Frailty, cognitive decline, neurodegenerative diseases and nutrition interventions [J]. International Journal of Molecular Sciences, 2019, 20(11).  [305] GONCHAROV N V, BELINSKAIA D A, AVDONIN P V. Organophospate-Induced Pathology: Mechanisms of Development, Principles of Therapy and Features of Experimental Studies [J]. Journal of Evolutionary Biochemistry and Physiology, 2023, 59(5): 1756-96.  [306] GONULTAS S, ERSOY A, BAHAT G, et al. Fatih province-geriatric study: Fragility and contributing factors in old population living the community [J]. European Geriatric Medicine, 2016, 7: S119.  [307] GONZALEZ M A, FEELY S M E, SPEZIANI F, et al. A novel mutation in VCP causes Charcot-Marie-tooth type 2 (CMT2) disease [J]. Journal of the Peripheral Nervous System, 2015, 20(2): 151.  [308] GONZáLEZ TOLEDO G, PéREZ PéREZ H, HERNáNDEZ GARCíA M, et al. Descriptive analysis of acquired motor neuron diseases in the northern area of tenerife [J]. Journal of Neuromuscular Diseases, 2021, 8(SUPPL 1): S15-S6.  [309] GORDON P H. Amyotrophic Lateral Sclerosis: An update for 2013 Clinical Features, Pathophysiology, Management and Therapeutic Trials [J]. Aging Dis, 2013, 4(5): 295-310.  [310] GORDZHELADZE K, BELOVA K, BELOV M. PREVALENCE OF GERIATRIC SYNDROMES IN PATIENTS 60 YEARS AND OLDER WITH PROXIMAL HUMERUS FRACTURE ENROLLED IN FRACTURE LIAISON SERVICE [J]. Aging Clinical and Experimental Research, 2023, 35: S545.  [311] GOSCH M, WICKLEIN S. [Antibodies as treatment option in older adults] [J]. Z Gerontol Geriatr, 2018, 51(2): 152-6.  [312] GOSSET P, CAMU W, RAOUL C, et al. Prionoids in amyotrophic lateral sclerosis [J]. Brain Commun, 2022, 4(3): fcac145.  [313] GöTZ J, BARMETTLER R, FERRARI A, et al. In vivo analysis of wild-type and FTDP-17 tau transgenic mice [Z]. 2000: 126-33.10.1111/j.1749-6632.2000.tb06914.x  [314] GöTZ J, BARMETTLER R, FERRARI A, et al. In vivo analysis of wild-type and FTDP-17 tau transgenic mice [J]. Ann N Y Acad Sci, 2000, 920: 126-33.  [315] GOVAARTS R, BEELDMAN E, BEELEN A, et al. Longitudinal validation of the ALS-FTD-questionnaire-preliminary results [J]. Neurology, 2016, 86(16).  [316] GOVINDAN V, GUPTA P N, GREESHMA S. Left ventricular non-compaction with hypertrophic cardiomyopathy, a mutually beneficial combination? [J]. Indian Heart Journal, 2021, 73: S59.  [317] GREWAL P, DOLBEC K, JASSIM T, et al. Mitochondrial encephalomyopathy with lactic acidosis and stroke-like episodes (MELAS)-identification through clinical and para-clinical features [J]. Annals of Neurology, 2019, 86: S46-S7.  [318] GROGAN J, DE JESUS S, NEWPORT K. Redefining Terminal: High Yield Neurology Updates for the Palliative Specialist (FR204) [J]. Journal of Pain and Symptom Management, 2022, 63(5): 802.  [319] GROPPER S, HUNT D, CHAPA D W. Sarcopenia and Psychosocial Variables in Patients in Intensive Care Units: The Role of Nutrition and Rehabilitation in Prevention and Treatment [J]. Critical care nursing clinics of North America, 2019, 31(4): 489-99.  [320] GU B J, WILEY J S. P2X7 as a scavenger receptor for innate phagocytosis in the brain [J]. British Journal of Pharmacology, 2018, 175(22): 4195-208.  [321] GUALANO B, ARTIOLI G G, POORTMANS J R, et al. Exploring the therapeutic role of creatine supplementation [J]. Amino Acids, 2010, 38(1): 31-44.  [322] GUERREIRO R, KARA E, LE BER I, et al. Genetic analysis of inherited leukodystrophies: Genotype phenotype correlations in the CSF1R gene [J]. Dementia and Geriatric Cognitive Disorders, 2012, 34: 9.  [323] GUERREIRO R, KARA E, LE BER I, et al. Genetic analysis of inherited leukodystrophies: genotype-phenotype correlations in the CSF1R gene [J]. JAMA Neurol, 2013, 70(7): 875-82.  [324] GUERREIRO R, KUN-RODRIGUES C, DARWENT L, et al. C9ORF72 in a large international cohort of neuropathologically diagnosed dementia with lewy bodies cases [J]. American Journal of Neurodegenerative Diseases, 2015, 4: 104.  [325] GUEST P C. The Impact of New Biomarkers and Drug Targets on Age-Related Disorders [J]. Methods Mol Biol, 2020, 2138: 3-28.  [326] GULCELIK N E, HAUL M, ARIOGUL S, et al. Adipocytokines and aging: Adiponectin and leptin [J]. Minerva Endocrinologica, 2013, 38(2): 203-10.  [327] GüNER OYTUN M, CEYLAN S, DOĞU B B, et al. Evaluation of quality of life related to dysphagia in Alzheimer dementia [J]. Clinical Nutrition ESPEN, 2021, 46: S685.  [328] GUO M. PINK1/Parkin and mitochondrial dynamics in neurodegeneration [J]. Free Radical Biology and Medicine, 2017, 112: 16.  [329] GUPTA D, MORLEY J E. Hypothalamic-pituitary-adrenal (HPA) axis and aging [J]. Comprehensive Physiology, 2014, 4(4): 1495-510.  [330] GUPTA R, THOMAS J, ANDERSON G, et al. The two-year outcome in patients with mhe and sarcopenia on mortality and the development of ohe [J]. American Journal of Gastroenterology, 2019, 114: S9-S11.  [331] GUPTA R, THOMAS J, ANDERSON G, et al. The adjunct of sarcopenia with MHE increases the 1 year risk of developing OHE [J]. Journal of Hepatology, 2019, 70(1): e645.  [332] GUPTA R, THOMAS J, ANDERSON G, et al. Understanding MHE: Screening, diagnosis and complications [J]. Hepatology, 2018, 68: 1147A-8A.  [333] GURHOLT T, BORDA M G, PARKER N, et al. 366. Connecting Sarcopenia, Brain Structure, and Cognitive Performance: Large-Scale Evidence From the UK Biobank [J]. Biological Psychiatry, 2023, 93(9): S241-S2.  [334] GUYONNET S, SECHER M, VELLAS B. Nutrition, Frailty, Cognitive Frailty and Prevention of Disabilities with Aging; proceedings of the 82nd Nestle-Nutrition-Institute Workshop, Gurgaon, INDIA, F Oct 13-18, 2013 [C]. 2015.  [335] HADDAD R, COHEN BITTAN J, CHALFINE C, et al. Association between actigraphy sleep parameters and recovery of walking ability after hip fracture [J]. European Geriatric Medicine, 2017, 8: S24.  [336] HAGE-MELIM L I D, FERREIRA J V, DE OLIVEIRA N K S, et al. The Impact of Natural Compounds on the Treatment of Neurodegenerative Diseases [J]. Current Organic Chemistry, 2019, 23(3): 335-60.  [337] HAGG S. The importance of nutrition for dementia and frailty: Evidence from epidemiological studies in aging [J]. Annals of Nutrition and Metabolism, 2023, 79: 25.  [338] HALBER M, BULANCEA S, TREIDLER S. Clinical and Electrophysiological Presentation of a Patient with Multi system Proteinopathy Associated with Valosin-Containing Protein Mutation [J]. Neurology, 2023, 100(17).  [339] HAMADA K, FUKAZAWA T, YANAGIHARA T, et al. Dementia with ALS features and diffuse Pick body-like inclusions (atypical Pick's disease?) [J]. Clin Neuropathol, 1995, 14(1): 1-6.  [340] HAMILTON J, KUSHNER B, HOLDEN S, et al. Age-Related Risk Factors in Ventral Hernia Repairs: A Review and Call to Action [J]. Journal of Surgical Research, 2021, 266: 180-91.  [341] HAMMERSEN S, BROCK M, CERVóS-NAVARRO J. Adult neuronal ceroid lipofuscinosis with clinical findings consistent with a butterfly glioma. Case report [J]. J Neurosurg, 1998, 88(2): 314-8.  [342] HAMSTRA S I, ROY B D, TIIDUS P, et al. Beyond its Psychiatric Use: The Benefits of Low-dose Lithium Supplementation [J]. Current Neuropharmacology, 2023, 21(4): 891-910.  [343] HAN X, ASHRAF M, TIPPARAJU S M, et al. Muscle–Brain crosstalk in cognitive impairment [J]. Frontiers in Aging Neuroscience, 2023, 15.  [344] HAN X, ASHRAF M, TIPPARAJU S M, et al. Muscle-Brain crosstalk in cognitive impairment [J]. Front Aging Neurosci, 2023, 15: 1221653.  [345] HANYU H. SARCOPENIA AND MUSCLE FUNCTIONS AT VARIOUS STAGES OF ALZHEIMER DISEASE [J]. Alzheimer's and Dementia, 2018, 14(7): P802.  [346] HANYU H. Prevention of dementia-with special reference to lifestyle and vascular risk factors [J]. Brain and Nerve, 2018, 70(3): 191-8.  [347] HANYU H. Diabetes-related dementia [Z]. 2019: 147-60.10.1007/978-981-13-3540-2_8  [348] HANYU H. Diabetes-Related Dementia [J]. Adv Exp Med Biol, 2019, 1128: 147-60.  [349] HARGUS G, EHRLICH M, HALLMANN A L, et al. Human stem cell models of neurodegeneration: A novel approach to study mechanisms of disease development [J]. Acta Neuropathologica, 2014, 127(2): 151-73.  [350] HASHIMOTO R, TAGUCHI T, KANO M, et al. [A case report of dementia with cluttering-like speech disorder and apraxia of gait] [J]. Rinsho Shinkeigaku, 1999, 39(5): 520-6.  [351] HATANO T, TAKANASHI M, SUMINO T, et al. [A 73-year-old woman with depression, dementia, and parkinsonism] [J]. No To Shinkei, 2005, 57(10): 907-14.  [352] HAWKINS K L, BROWN T T, MARGOLICK J B, et al. Geriatric syndromes: new frontiers in HIV and sarcopenia [J]. Aids, 2017, 31 Suppl 2(Suppl 2): S137-s46.  [353] HAWKINS S, HAYLEY D C. Incorporating a rapid geriatric assessment in primary care [J]. Journal of the American Geriatrics Society, 2021, 69(SUPPL 1): S244-S5.  [354] HAYASHI M, KOBAYASHI K, ISHIDA C, et al. Non-Alzheimer dementia with status spongiosus and neuronal cell loss showing unusual perineuronal structures and point mutation at 129 codon of prion protein [J]. Dement Geriatr Cogn Disord, 1997, 8(1): 55-9.  [355] HAYASHI Y. [Inclusion body myopathy with Paget's disease of bone and frontotemporal dementia] [J]. Rinsho Shinkeigaku, 2013, 53(11): 947-50.  [356] HE Y, WANG Z. The roles of HSP40/DNAJ protein family in neurodegenerative diseases [J]. Zhejiang da xue xue bao Yi xue ban = Journal of Zhejiang University Medical sciences, 2022, 51(5): 640-6.  [357] HEALTON E B, SAVAGE D G, BRUST J C M, et al. Neurologic aspects of cobalamin deficiency [J]. Medicine, 1991, 70(4): 229-45.  [358] HELLER J, BRCINA N, DOGAN I, et al. Brain imaging findings in idiopathic REM sleep behavior disorder (RBD) - A systematic review on potential biomarkers for neurodegeneration [J]. Sleep Medicine Reviews, 2017, 34: 23-33.  [359] HENSEL N, RADEMACHER S, CLAUS P. Chatting with the neighbors: crosstalk between Rho-kinase (ROCK) and other signaling pathways for treatment of neurological disorders [J]. Front Neurosci, 2015, 9: 198.  [360] HENSTRIDGE C M, PICKETT E, SPIRES-JONES T L. Synaptic pathology: A shared mechanism in neurological disease [J]. Ageing Research Reviews, 2016, 28: 72-84.  [361] HEROLD F, TöRPEL A, SCHEGA L, et al. Functional and/or structural brain changes in response to resistance exercises and resistance training lead to cognitive improvements - a systematic review [J]. European Review of Aging and Physical Activity, 2019, 16.  [362] HERSKOVITS A Z, GUARENTE L. Sirtuin deacetylases in neurodegenerative diseases of aging [J]. Cell Research, 2013, 23(6): 746-58.  [363] HIDAKA S, OHTAKE T, OKA M, et al. Mild cognitive impairment is associated with sarcopenia in hemodialysis patients [J]. Nephrology Dialysis Transplantation, 2019, 34: a300.  [364] HIGAMI Y. The quantity and quality controls of adipocyte mitochondria in extension of healthy lifespan [J]. Pathology International, 2022, 72(11): 572-3.  [365] HILTON G. MELAS: a mitochondrial encephalomyopathy syndrome [J]. The Journal of neuroscience nursing : journal of the American Association of Neuroscience Nurses, 1995, 27(5): 278-82.  [366] HIROSE D, HANYU H, FUKASAWA R, et al. Diabetes-related dementia is associated with dynapenia, but not with sarcopenia [J]. Geriatrics and Gerontology International, 2017, 17(1): 175-7.  [367] HIROSE D, SHIMIZU S, OGAWA Y, et al. NEUROIMAGING CHARACTERISTIC OF FRAILTY STATUS IN PATIENTS WITH ALZHEIMER'S DISEASE [J]. Alzheimer's and Dementia, 2018, 14(7): P445.  [368] HIRT J, VETSCH J, HEINRICH S. Facilitators and barriers to implement nurse-led physical activities for people with dementia in nursing homes: A protocol for a mixed-methods systematic review [J]. BMJ Open, 2021, 11(12).  [369] HOHLFELD R. Immunopathogenesis of myositis [J]. Nervenheilkunde, 1996, 15(1): 1-3.  [370] HOLM A, HANSEN S N, KLITGAARD H, et al. Clinical advances of RNA therapeutics for treatment of neurological and neuromuscular diseases [J]. RNA Biology, 2022, 19(1): 594-608.  [371] HOLM I E, ALSTRUP A K O, LUO Y. Genetically modified pig models for neurodegenerative disorders [J]. Journal of Pathology, 2016, 238(2): 267-87.  [372] HOLT I J, HARDING A E, PETTY R K, et al. A new mitochondrial disease associated with mitochondrial DNA heteroplasmy [J]. Am J Hum Genet, 1990, 46(3): 428-33.  [373] HOLZBAUR E L F. Motor neurons rely on motor proteins [J]. Trends in Cell Biology, 2004, 14(5): 233-40.  [374] HOOPER S, HACKING M, MANDER A. Osteoporosis and Sarcopenia. Are we missing the boat? Should first line treatment/prevention be transdermal oestrogen for women? [J]. Post Reproductive Health, 2022, 28(3): 171.  [375] HOPMANN D, KIVI A, STENZEL W, et al. Late onset myopathy and hyperkinetic movement disorder [J]. Clinical Neurophysiology, 2014, 125: S209.  [376] HOPPE K, PLUNIEN R, LEHMANN-HORN F, et al. OrphanAnesthesia – Recommendation for the anaesthesiological management of patients with mitochondrial cytopathy and hurler syndrome [J]. Anasthesiologie und Intensivmedizin, 2021, 62(7-8): 324-33.  [377] HOPPE K, PLUNIEN R, LEHMANN-HORN F, et al. OrphanAnesthesia - Recommendation for the anaesthesiological management of patients with Mitochondrial cytopathy and Hurler syndrome [J]. Anasthesiologie & Intensivmedizin, 2021, 62: 324-9.  [378] HUANG J H, HOOD D A. Age-associated mitochondrial dysfunction in skeletal muscle: Contributing factors and suggestions for long-term interventions [J]. IUBMB Life, 2009, 61(3): 201-14.  [379] HUANG K, CAI H L, BAO J P, et al. Dehydroepiandrosterone and age-related musculoskeletal diseases: Connections and therapeutic implications [J]. Ageing Research Reviews, 2020, 62.  [380] HUANG Z, ADACHI H. Natural Compounds Preventing Neurodegenerative Diseases Through Autophagic Activation [J]. Journal of UOEH, 2016, 38(2): 139-48.  [381] HUDSON J, FIGUEROA-BONAPARTE S, BARRESI R, et al. Mutational spectrum and phenotypic variability of VCP related neurological disease in the UK [J]. Neuromuscular Disorders, 2015, 25: S34.  [382] HUGHES S E, FITZPATRICK A S, KETTLE P J, et al. Polymyositis or another mimic? [J]. Journal of Neurology, Neurosurgery and Psychiatry, 2009, 80(4): 456.  [383] HUNT A P, MINETT G M, GIBSON O R, et al. Could Heat Therapy Be an Effective Treatment for Alzheimer's and Parkinson's Diseases? A Narrative Review [J]. Frontiers in Physiology, 2020, 10.  [384] HURLEY B F, HANSON E D, SHEAFF A K. Strength training as a countermeasure to aging muscle and chronic disease [J]. Sports medicine (Auckland, NZ), 2011, 41(4): 289-306.  [385] HURTADO M L, FULLER H R, EATON S L, et al. Molecular profiling of differentially vulnerable synaptic populations and in-vivo phenotypic assessment identifies regulators of neuronal stability [J]. Brain and Neuroscience Advances, 2017, 1: 177.  [386] IBRAHIM A, MEYER J. Neuromuscular respiratory dysfunction due to vitamin B12 deficiency: a case report [J]. Neurology, 2023, 100(17).  [387] ICHIKAWA H. Language in ALS/FTLD [J]. Clinical Neurology, 2010, 50(11): 1014-6.  [388] ICHIKAWA H. [Language disorders in ALS/FTLD] [J]. Rinshō shinkeigaku = Clinical neurology, 2010, 50(11): 1014-6.  [389] IDE H. The impact of testosterone in men's health [J]. Endocrine Journal, 2023, 70(7): 655-62.  [390] IGARI R, WADA M, SATO H, et al. [A case of inclusion body myopathy with Paget's disease of bone and frontotemporal dementia (IBMPFD) showing clinical features of motor neuron disease] [J]. Rinsho Shinkeigaku, 2013, 53(6): 458-64.  [391] IKAWA M, YONEDA M, KURIYAMA M. [A case of chorea-acanthocytosis onset with at age 86] [J]. Rinsho Shinkeigaku, 2005, 45(8): 603-6.  [392] IKEDA K. Where fronto-temporal dementia should be placed in the history of Pick's disease and related disorders [J]. Seishin shinkeigaku zasshi = Psychiatria et neurologia Japonica, 2000, 102(6): 529-42.  [393] IKENAGA C, MAEDA M, TSUJI S, et al. Clinicopathological features of anti-NT5C1A positive patients in the group of myositis patients with CD8-MHC-1 complex pathology [J]. Neurology, 2016, 86(16).  [394] IMAI H, FURUKAWA Y, SUMINO S, et al. [A 65-year-old woman with dysarthria, dysphagia, weakness, and gait disturbance] [J]. No To Shinkei, 1995, 47(4): 399-410.  [395] IMANI M, BORDA M, WEON M, et al. DEVELOPMENT OF A FULLY AUTOMATIC TECHNIQUE FOR SEGMENTATION AND QUANTIFICATION OF MASSETER AND TONGUE MUSCLES: A CROSSROAD OF SARCOPENIA AND DEMENTIA [J]. Aging Clinical and Experimental Research, 2022, 34: S154-S5.  [396] IMANI M, BORDA M G, WEON M, et al. INVESTIGATING CLINICAL APPLICATIONS OF AN AUTOMATIC TECHNIQUE FOR ANALYZING MASTICATORY AND TONGUE MUSCLES IN DEMENTIA PATIENTS [J]. Aging Clinical and Experimental Research, 2023, 35: S418.  [397] INCE P G, LOWE J, SHAW P J. Amyotrophic lateral sclerosis: Current issues in classification, pathogenesis and molecular pathology [J]. Neuropathology and Applied Neurobiology, 1998, 24(2): 104-17.  [398] INGLIS F. The tolerability and safety of cholinesterase inhibitors in the treatment of dementia [J]. International Journal of Clinical Practice, 2002: 45-63.  [399] INOUE S. Vitamin K, GGCX, and SXR [J]. Annals of Nutrition and Metabolism, 2023, 79: 51-2.  [400] INOUE S, ISHII R, FUKUDA H, et al. Sevoflurane anaesthesia for a patient with adult polyglucosan body disease [J]. Can J Anaesth, 1996, 43(12): 1257-9.  [401] INSKIP M, MAVROS Y, SACHDEV P S, et al. Interrupting the trajectory of frailty in dementia with Lewy bodies with anabolic exercise, dietary intervention and deprescribing of hazardous medications [J]. BMJ Case Rep, 2020, 13(4).  [402] IRUMUDOMON O, GHOSH P S. Clinical Reasoning: Young adult with dysphagia and severe weight loss [J]. Neurology, 2018, 91(11): E1083-E6.  [403] ISHII A, OKUNE S, HOSAKA T, et al. A family of IBMPFD showing variable clinical features [J]. Journal of Neuromuscular Diseases, 2021, 8(SUPPL 1): S3.  [404] ISHIKAWA T, MORITA M, NAKANO I. Brain perfusion imaging in amyotrophic lateral sclerosis with dementia [J]. Brain and Nerve, 2007, 59(10): 1093-8.  [405] ISHIKAWA T, NAKAMURA K, SHIMASAKI R, et al. [A case of mitochondrial disease with multiple mitochondrial DNA deletions suspected amyotrophic lateral sclerosis-frontotemporal dementia] [J]. Rinsho Shinkeigaku, 2018, 58(1): 15-20.  [406] ISKUSNYKH I Y, ZAKHAROVA A A, KRYL’SKII E D, et al. Aging, Neurodegenerative Disorders, and Cerebellum [J]. International Journal of Molecular Sciences, 2024, 25(2).  [407] ITO K, SANO T, KAMIYA K, et al. Massive accumulation of 11C-Pittsburg compound B in the occipital lobes of a patient with early-onset dementia accompanied by muscle weakness and hypertonicity [J]. Ann Nucl Med, 2013, 27(10): 935-41.  [408] ITO T, HOKEZU Y, MORI T, et al. [A case of motor neuron disease with presenile dementia showing bilateral degeneration of the pyramidal tract on cranial MRI] [J]. Rinsho Shinkeigaku, 2001, 41(1): 60-3.  [409] IWABUCHI K, YAGISHITA S, AMANO N, et al. [An autopsied Japanese case of hereditary olivo-ponto-cerebellar atrophy compatible with the original one of Menzel's report (1891)] [J]. No To Shinkei, 1993, 45(4): 381-7.  [410] IWABUCHI K, YAGISHITA S, AMANO N, et al. [An autopsy case of complicated form of spastic paraplegia with amyotrophy, mental deficiency, sensory impairment, and parkinsonism] [J]. No To Shinkei, 1990, 42(11): 1075-83.  [411] IZHBOLDINA O, ZHUKOVA N, ZHUKOVA I, et al. Amyotrophic lateral sclerosis/parkinsonism/dementia complex of Guam: A case report [J]. Journal of the Neurological Sciences, 2017, 381: 1055.  [412] IZQUIERDO M, MERCHANT R A, MORLEY J E, et al. International Exercise Recommendations in Older Adults (ICFSR): Expert Consensus Guidelines [J]. J Nutr Health Aging, 2021, 25(7): 824-53.  [413] JABLONKA S, HENNLEIN L, SENDTNER M. Therapy development for spinal muscular atrophy: perspectives for muscular dystrophies and neurodegenerative disorders [J]. Neurological Research and Practice, 2022, 4(1).  [414] JAGER-WITTENAAR H, JONES S, ROTHENBERG E, et al. Malnutrition and dietary treatment in older adults-a conference report [J]. Annals of Nutrition and Metabolism, 2020, 76(1): 96-7.  [415] JAHN K, HEINZE C, SELGE C, et al. Gait disorders in geriatric patients: Classification and therapy [J]. Nervenarzt, 2015.  [416] JENNUM P, CHRISTENSEN J A E, ZOETMULDER M. Neurophysiological basis of rapid eye movement sleep behavior disorder: informing future drug development [J]. Nature and Science of Sleep, 2016, 8: 107-20.  [417] JEONG Y C, PARK J S, KIM S H, et al. Frontotemporal Dementia with Motor Neuron Disease in a Patient with Antiphospholipid Syndrome: A Case Report [J]. Dement Neurocogn Disord, 2016, 15(4): 165-9.  [418] JIMéNEZ E L, ÁLVAREZ M N, MARíN R R, et al. Muscle loss in older adults during an hospitalization for acute disease. The ECOSARC Project [J]. European Geriatric Medicine, 2022, 13: S89-S90.  [419] JIMENEZ GARDUñO A M, MARTINEZ ROJAS V A, JUAREZ HERNANDEZ L J, et al. The role of altered voltage-gated currents in motor-neuron degeneration: Analysing the spinal and bulbar muscular atrophy (SBMA) case [J]. Acta Physiologica, 2019, 227: 78.  [420] JIMENEZ GUTIERREZ G E, BORBOLLA JIMéNEZ F V, MUñOZ L G, et al. The Molecular Role of Polyamines in Age-Related Diseases: An Update [J]. International Journal of Molecular Sciences, 2023, 24(22).  [421] JIN H, XIE W, HE M, et al. Pyroptosis and Sarcopenia: Frontier Perspective of Disease Mechanism [J]. Cells, 2022, 11(7).  [422] JIN HEE L, TAE HUI K, HYUN-GHANG J, et al. Association of sarcopenia and cognitive impairment among older people in Korea [J]. European Neuropsychopharmacology, 2015, 25: S594.  [423] JIN X, SLEE A, SMITHARD D. Clinical audit of older hospital patients investigating the prevalence of malnutrition, sarcopenia and frailty [J]. European Geriatric Medicine, 2022, 13: S374.  [424] JO D, YOON G, KIM O Y, et al. A new paradigm in sarcopenia: Cognitive impairment caused by imbalanced myokine secretion and vascular dysfunction [J]. Biomed Pharmacother, 2022, 147: 112636.  [425] JOU M J. Aβ augments mCa2+ independent mros-mediated shift of lethal transient mitochondrial permeability transition to its permanent mode in narp cybrids [J]. Journal of the Neurological Sciences, 2013, 333: e326.  [426] JUBY A, DAVIS C, MINIMAANA S. Dxa body composition (BC) should be assessed in all geriatric patients referred for DXA bone mineral density (BMD) assessment [J]. Journal of Bone and Mineral Research, 2017, 31.  [427] JUN L, ROBINSON M, GEETHA T, et al. Prevalence and Mechanisms of Skeletal Muscle Atrophy in Metabolic Conditions [J]. International Journal of Molecular Sciences, 2023, 24(3).  [428] JUNG C H, MOK J O. Recent Updates on Associations among Various Obesity Metrics and Cognitive Impairment: from Body Mass Index to Sarcopenic Obesity [J]. Journal of Obesity and Metabolic Syndrome, 2022, 31(4): 287-95.  [429] JUNG Y W, HYSOLLI E, KIM K Y, et al. Human induced pluripotent stem cells and neurodegenerative disease: Prospects for novel therapies [J]. Current Opinion in Neurology, 2012, 25(2): 125-30.  [430] JUNTAS M R, PAGEOT N, CAMU W, et al. Sporadic case of inclusion body myositis-paget disease and frontotemporal dementia mimicking amyotrophic lateral sclerosis [J]. Amyotrophic Lateral Sclerosis, 2009, 10: 129-30.  [431] JYVäKORPI S K, PITKALA K H, PURANEN T M, et al. Low protein and micronutrient intakes in various groups of older people [J]. Clinical Nutrition, 2015, 34: S202-S3.  [432] KABASHI E, BRUSTEIN E, CHAMPAGNE N, et al. Zebrafish models for the functional genomics of neurogenetic disorders [J]. Biochimica et Biophysica Acta - Molecular Basis of Disease, 2011, 1812(3): 335-45.  [433] KAHN A J, AUSTAD S, PAHOR M, et al. Meeting Report. Central and peripheral mechanisms of aging and frailty: A report on the 8th longevity consortium symposium, Santa Fe, New Mexico, May 16-18, 2007 [J]. Journals of Gerontology - Series A Biological Sciences and Medical Sciences, 2007, 62(12): 1357-60.  [434] KAKUTANI N, FUKUSHIMA A, NAKAMURA R, et al. Skeletal muscle atrophy is associated with presymptomatic hippocampal atrophy in elderly patients with heart failure [J]. European Heart Journal, 2015, 36: 982.  [435] KALARIA R N. Cerebral vessels in ageing and Alzheimer's disease [J]. Pharmacology & Therapeutics, 1996, 72(3): 193-214.  [436] KALIMO H, VIITANEN M, AMBERLA K, et al. CADASIL:: hereditary disease of arteries causing brain infarcts and dementia [J]. Neuropathology and Applied Neurobiology, 1999, 25(4): 257-65.  [437] KALYAN M, TOUSIF A H, SONALI S, et al. Role of Endogenous Lipopolysaccharides in Neurological Disorders [J]. Cells, 2022, 11(24).  [438] KAMBRATH A V, SURYADEVARA V, SATO A, et al. Musculoskeletal and cardiac defects in the microtubule associated protein Tau (MAPT) P301S Tg+ mouse model of Frontotemporal Dementia [J]. Journal of Bone and Mineral Research, 2020, 35(SUPPL 1): 157.  [439] KANE R L, SHAMLIYAN T, TALLEY K, et al. The association between geriatric syndromes and survival [J]. J Am Geriatr Soc, 2012, 60(5): 896-904.  [440] KANE R L, TALLEY K M C, SHAMLIYAN T, et al. U.S. Preventive Services Task Force Evidence Syntheses, formerly Systematic Evidence Reviews [M]. Common Syndromes in Older Adults Related to Primary and Secondary Prevention. Rockville (MD); Agency for Healthcare Research and Quality (US). 2011.  [441] KANETO T, INOUE K, SHIMODA K, et al. [An autopsied case of progressive spinal muscular atrophy showing tremor and choreiform movement] [J]. Rinsho Shinkeigaku, 2000, 40(8): 801-6.  [442] KANOVA M, KOHOUT P. Tryptophan: A Unique Role in the Critically Ill [J]. International Journal of Molecular Sciences, 2021, 22(21).  [443] KANZAKI M, SATO M, OGAWA G, et al. [A case of dementia with motor neuron disease associated with agraphia--the omission of kana letters] [J]. Rinsho Shinkeigaku, 2004, 44(10): 673-6.  [444] KASE N G, GRETZ FRIEDMAN E, BRODMAN M, et al. The midlife transition and the risk of cardiovascular disease and cancer Part I: magnitude and mechanisms [J]. American Journal of Obstetrics and Gynecology, 2020, 223(6): 820-33.  [445] KASIM Z. Validation of SARC-F for the screening of sarcopenia in elderly patients with dementia: A cross-sectional study [J]. European Geriatric Medicine, 2018, 9: S101.  [446] KASIM Z, RODRíGUEZ-GARCíA W D, PERKISAS S, et al. Validation of SARC-F by proxy (SARC-F-Proxy) for the screening of sarcopenia in elderly patients with dementia: A cross-sectional study [J]. European Geriatric Medicine, 2018, 9: S105.  [447] KASSAR D, CHAND P, IYADURAI S. Parkinsonism-dementia-ALS complex phenotype in an African-American patient [J]. Journal of Neurology, 2012, 259(1): S132.  [448] KASSAR D, IYADURAI S. Parkinsonism-dementia-ALS complex (PDA) phenotype in an African-American patient [J]. Neurology, 2012, 78(1).  [449] KATO A. Arterial stiffening and clinical outcomes in dialysis patients [J]. Pulse, 2015, 3(2): 89-97.  [450] KATUSIC Z S, AUSTIN S A. Neurovascular Protective Function of Endothelial Nitric Oxide - Recent Advances [J]. Circulation Journal, 2016, 80(7): 1499-503.  [451] KATZEFF J S, BRIGHT F, PHAN K, et al. Biomarker discovery and development for frontotemporal dementia and amyotrophic lateral sclerosis [J]. Brain, 2022, 145(5): 1598-609.  [452] KAWADA T. Sarcopenia and sleep status in older female patients with mild to moderate Alzheimer's disease: a risk assessment [J]. Psychogeriatrics, 2024, 24(2): 521.  [453] KAWAKAMI I, ARAI T, NIIZATO K, et al. A case of early onset dementia as depressive state at age 32 with chorea-like involuntary movement [J]. Neuropathology, 2013, 33(3): 338.  [454] KAZAMEL M, SORENSON E, MCEVOY K, et al. Spectrum of valosin containing protein (VCP)opathy: A case series [J]. Neurology, 2015, 84.  [455] KEIGHRON C N, AVAZZADEH S, GOLJANEK-WHYSALL K, et al. Extracellular Vesicles, Cell-Penetrating Peptides and miRNAs as Future Novel Therapeutic Interventions for Parkinson's and Alzheimer's Disease [J]. Biomedicines, 2023, 11(3).  [456] KELLEHER K, LYNCH S A, LYNCH B. Late and Atypical presentation of MECP2 mutation [J]. Archives of Disease in Childhood, 2019, 104: A350.  [457] KERR N R, BOOTH F W. Contributions of physical inactivity and sedentary behavior to metabolic and endocrine diseases [J]. Trends Endocrinol Metab, 2022, 33(12): 817-27.  [458] KHALIL B, MORDERER D, PRICE P L, et al. mRNP assembly, axonal transport, and local translation in neurodegenerative diseases [J]. Brain Research, 2018, 1693: 75-91.  [459] KHALIMONCHUK O, BECKER D F. Molecular Determinants of Mitochondrial Shape and Function and Their Role in Glaucoma [J]. Antioxidants and Redox Signaling, 2023, 38(13): 896-919.  [460] KHAN A, JAHAN S, IMTIYAZ Z, et al. Neuroprotection: Targeting Multiple Pathways by Naturally Occurring Phytochemicals [J]. Biomedicines, 2020, 8(8).  [461] KHAN H A, RAUOF K A. ROLE OF FLAVANOIDS IN TREATMENT OF ALZHEIMERS DISEASE [J]. International Journal of Applied Pharmaceutics, 2023, 15(1): 161.  [462] KHARE A, INDIRANI M, KALAL S, et al. Metabolic imaging with FDG PET in motor neuron disease [J]. Indian Journal of Nuclear Medicine, 2015, 30(5): S30.  [463] KIHIRA T, MURATA K, MORITA S, et al. TDP-43 immunoreactivity in IBM muscle [J]. Neuropathology, 2009, 29(3): 340.  [464] KIM S H, SIN D S, LIM J Y. Newly Diagnosed Sarcopenia and Alzheimer's Disease in an Older Patient With Chronic Inflammation [J]. Ann Geriatr Med Res, 2019, 23(1): 38-41.  [465] KIM Y, JANG S N. Mapping the knowledge structure of frailty in journal articles by text network analysis [J]. PLoS One, 2018, 13(4): e0196104.  [466] KIMONIS V. Inclusion Body Myopathy with Paget Disease of Bone and/or Frontotemporal Dementia [M]//ADAM M P, FELDMAN J, MIRZAA G M, et al. GeneReviews(®). Seattle (WA); University of Washington, Seattle  Copyright © 1993-2024, University of Washington, Seattle. GeneReviews is a registered trademark of the University of Washington, Seattle. All rights reserved. 1993.  [467] KIMONIS V, BADADANI M, NALBANDIAN A, et al. Inclusion body myopathy and paget's disease of bone with frontotemporal dementia: New insights [J]. Dementia and Geriatric Cognitive Disorders, 2010, 30: 27.  [468] KIMONIS V E, FULCHIERO E, VESA J, et al. VCP disease associated with myopathy, Paget disease of bone and frontotemporal dementia: Review of a unique disorder [J]. Biochimica et Biophysica Acta - Molecular Basis of Disease, 2008, 1782(12): 744-8.  [469] KIMONIS V E, MEHTA S G, FULCHIERO E C, et al. Clinical studies in familial VCP myopathy associated with Paget disease of bone and frontotemporal dementia [J]. Am J Med Genet A, 2008, 146a(6): 745-57.  [470] KIMURA M, SAITO S. [Anesthesia for patients with neurological diseases] [J]. Masui, 2010, 59(9): 1100-4.  [471] KINOSHITA M, MUTOH S, KASAI A, et al. General Anesthesia in a Patient With Neuronal Intranuclear Inclusion Disease: A Case Report [J]. A A Pract, 2022, 16(10): e01633.  [472] KIRK R. Clinical trials in CNS - SMi's eighth annual conference [J]. IDrugs, 2010, 13(2): 66-9.  [473] KITAMURA S, TAJI N, KOMIYAMA T, et al. [A case of motor neuron disease with dementia--cerebral blood flow and cerebral oxygen metabolism] [J]. Rinsho Shinkeigaku, 1992, 32(1): 57-61.  [474] KITO Y, KAZUI H, YOSHIDA T, et al. [Language and semantic memory impairment in a patient with motor neuron disease and semantic dementia: a case report] [J]. Brain Nerve, 2010, 62(6): 625-30.  [475] KLEINEBERG N, KNAUSS S, GüLKE E, et al. Neurological implications of COVID-19-results of the LEOSS registry [J]. European Journal of Neurology, 2021, 28(SUPPL 1): 102.  [476] KLINGELHöFER L, STORCH A, REICHMANN H, et al. Idiopathic Parkinson's disease and proximal myotonic myopathy-A case report of a 71-year-old woman [J]. Journal of Neurology, 2010, 257: S71.  [477] KLOTH K, COZMA C, BESTER M, et al. Dystonia as initial presentation of compound heterozygous GBA2 mutations: Expanding the phenotypic spectrum of SPG46 [J]. European Journal of Medical Genetics, 2020, 63(9).  [478] KOBAYASHI K, KURACHI M, GYOUBU T, et al. Progressive dysphasic dementia with localized cerebral atrophy: report of an autopsy [J]. Clin Neuropathol, 1990, 9(5): 254-61.  [479] KOFOED R H, NOSEWORTHY K, WU K, et al. Focused ultrasound increases gene delivery to deep brain structure following the administration of a recombinant adeno-associated virus in the cerebrospinal fluid [Z]. 2024.10.1101/2024.02.09.579587  [480] KOGA Y, YATSUGA S, AKITA Y, et al. Natural course of melas in Japanese cohort study [J]. Journal of the Neurological Sciences, 2009, 283(1-2): 248.  [481] KOLOSOVA N G, STEFANOVA N A, KORBOLINA E E, et al. [The senescence-accelerated oxys rats--a genetic model of premature aging and age-dependent degenerative diseases] [J]. Advances in gerontology = Uspekhi gerontologii / Rossiĭskai͡a akademii͡a nauk, Gerontologicheskoe obshchestvo, 2014, 27(2): 336-40.  [482] KONDOH H, TERUYA T, KAMEDA M, et al. Decline of ergothioneine in frailty and cognition impairment [J]. FEBS Lett, 2022, 596(10): 1270-8.  [483] KONDOH T, AMAMOTO N, DOI T, et al. Dramatic improvement in Down syndrome-associated cognitive impairment with donepezil [J]. Ann Pharmacother, 2005, 39(3): 563-6.  [484] KOSTOGLOU-ATHANASSIOU I, ATHANASSIOU L, SPYROPOULOS P, et al. Dementia and sarcopenia [J]. Osteoporosis International, 2018, 29(1): S410-S1.  [485] KRAJCIK S, MIKUS P. Sarcopenic obesity: Diagnosis and nutritional treatment [J]. Vnitrni Lekarstvi, 2017, 63(9): 2S40.  [486] KRAMER A. An Overview of the Beneficial Effects of Exercise on Health and Performance [Z]. 2020: 3-22.10.1007/978-981-15-1792-1_1  [487] KRAMER A. An Overview of the Beneficial Effects of Exercise on Health and Performance [J]. Adv Exp Med Biol, 2020, 1228: 3-22.  [488] KRAUSE S. Insights into muscle degeneration from heritable inclusion body myopathies [J]. Frontiers in Aging Neuroscience, 2015, 7(FEB).  [489] KRENTZ A J, VILJOEN A, SINCLAIR A. Insulin resistance: A risk marker for disease and disability in the older person [J]. Diabetic Medicine, 2013, 30(5): 535-48.  [490] KRZNARIĆ Ž, BENDER D V, KELEČIĆ D L, et al. Croatian guidelines for nutrition in the elderly, Part II - Clinical nutrition [J]. Lijecnicki Vjesnik, 2011, 133(9-10): 299-307.  [491] KUCHTA K, CAMERON S. Phytotherapy for Cachexia: Where Do We Stand? [J]. Frontiers in Pharmacology, 2020, 11.  [492] KUMARI N, ANAND S, SHAH K M, et al. Emerging Role of Plant-Based Bioactive Compounds as Therapeutics in Parkinson's Disease [J]. Molecules, 2023, 28(22).  [493] KURT INCESU T, BECKMANN Y, TOKUC¸OʇU F, et al. Unusual clinical manifestations of hereditary inclusion myopathy [J]. European Journal of Neurology, 2014, 21: 525.  [494] KUSHNIR A, WAJSBERG B, MARKS A R. Ryanodine receptor dysfunction in human disorders [J]. Biochim Biophys Acta Mol Cell Res, 2018, 1865(11 Pt B): 1687-97.  [495] KYE M J, GONçALVES I C G. The role of miRNA in motor neuron disease [J]. Frontiers in Cellular Neuroscience, 2014, 8(JAN).  [496] KYE M J, GONçALVES I D G. The role of rniRNA in motor neuron disease [J]. Frontiers in Cellular Neuroscience, 2014, 8.  [497] LA SPADA A R, WEYDT P, PINEDA V V. Frontiers in Neuroscience  Huntington’s Disease Pathogenesis: Mechanisms and Pathways [M]//LO D C, HUGHES R E. Neurobiology of Huntington's Disease: Applications to Drug Discovery. Boca Raton (FL); CRC Press/Taylor & Francis  Copyright © 2011 by Taylor and Francis Group, LLC. 2011.  [498] LABRIE F. DHEA, important source of sex steroids in men and even more in women [M]//MARTINI L, CHROUSOS G P, LABRIE F, et al. Neuroendocrinology: Pathological Situations and Diseases. 2010: 97-148.  [499] LABRLE F. Highly efficient treatment of vaginal atrophy and sexual dysfunction by a strictly local action of prasterone (DHEA) [J]. Revista Argentina de Endocrinologia y Metabolismo, 2012, 49: 10-1.  [500] LABRLE F. Role of intracrinology or peripheral sex steroid formation in women and men [J]. Revista Argentina de Endocrinologia y Metabolismo, 2012, 49: 16-8.  [501] LAI C H, CHEN H C, LIOU T H, et al. Exercise Interventions for Individuals With Neurological Disorders A Systematic Review of Systematic Reviews [J]. American Journal of Physical Medicine & Rehabilitation, 2019, 98(10): 921-30.  [502] LAMBERT-SMITH I A, SAUNDERS D N, YERBURY J J. The pivotal role of ubiquitin-activating enzyme E1 (UBA1) in neuronal health and neurodegeneration [J]. International Journal of Biochemistry and Cell Biology, 2020, 123.  [503] LARIJANI B, SHAFIEE G, OSTOVAR A, et al. Association of osteosarcopenia and cognitive impairment in a community dwelling older population: The Bushehr Elderly Health (BEH) program [J]. Journal of Bone and Mineral Research, 2018, 33: 313.  [504] LAVORGNA T R, GRESSETT T E, CHASTAIN W H, et al. Perlecan: a review of its role in neurologic and musculoskeletal disease [J]. Frontiers in Physiology, 2023, 14.  [505] LE QUINTREC J L, AGRHUM WORKING GROUP A. Exercise and weight loss in the management of hip and knee osteoarthritis in very old patients: Current data and how to prescribe? [J]. Osteoporosis International, 2019, 30(SUPPL 2): S229.  [506] LEAVITT B R, WILD E J, BANG J, et al. Intrathecal drug delivery of antisense oligonucleotides in huntington's disease: Experience of ionis/roche RG6042 development programme and best practice considerations for real-world use [J]. Neurology, 2020, 94(15).  [507] LECARPENTIER Y, VALLéE A. Opposite interplay between PPAR gamma and canonical Wnt/beta-catenin pathway in amyotrophic lateral sclerosis [J]. Frontiers in Neurology, 2016, 7(JUN).  [508] LECCESE D, RODOLICO G R, SPERTI M, et al. Sex influences clinical phenotype in valosin-containing protein mutations: A case family report and systematic literature review [J]. Clin Neurol Neurosurg, 2023, 232: 107875.  [509] LECHA M, PEñALVA A, BACH P, et al. Malnutrition and risk of malnutrition in complex chronic outpatients in Baix Llobregat (Barcelona) [J]. Clinical Nutrition ESPEN, 2023, 58: 730.  [510] LEE C, LIN H, WANG Y, et al. Ginkgo biloba extract ameliorates aging-related sarcopenia [J]. Cytotherapy, 2020, 22(5): S173.  [511] LEE C N, YANG S C, LI C K, et al. ALARM SYSTEM FOR BED EXIT AND PROLONGED BED REST; proceedings of the International Conference on Machine Learning and Cybernetics (ICMLC), Chengdu, PEOPLES R CHINA, F Jul 15-18, 2018 [C]. 2018.  [512] LEE H G, ARAI I, KWON S. A Herbal Prescription of Insamyangyeongtang as a Therapeutic Agent for Frailty in Elderly: A Narrative Review [J]. Nutrients, 2024, 16(5).  [513] LEE J, YOON D, SUNG K W, et al. Targeted degradation of SNCA/α-synuclein aggregates in neurodegeneration using the AUTOTAC chemical platform [J]. Autophagy, 2024, 20(2): 463-5.  [514] LEE M, MENG D, KIERNAN M, et al. Exploring motor imagery and motor cortical function in amyotrophic lateral sclerosis using magnetoencephalography [J]. Amyotrophic Lateral Sclerosis and Frontotemporal Degeneration, 2015, 16: 110.  [515] LEE O, MOON S, LEE S U, et al. Relationship between cognitive function and metabolic risk factors in Korean elderly [J]. FASEB Journal, 2015, 29(1).  [516] LEE Y, LEE B H, YIP W, et al. Neurofilament proteins as prognostic biomarkers in neurological disorders [J]. Current Pharmaceutical Design, 2019, 25(43): 4560-9.  [517] LEHRER S, RHEINSTEIN P H. Transspinal delivery of drugs by transdermal patch back-of-neck for Alzheimer's disease: a new route of administration [J]. Discov Med, 2019, 27(146): 37-43.  [518] LEU-SEMENESCU S, ARNULF I. Disruptive nocturnal behavior in elderly subjects: could it be a parasomnia? [J]. Psychologie & Neuropsychiatrie Du Vieillissement, 2010, 8(2): 97-109.  [519] LEWIS M. V.E.R.C.I. (Veterans Enrolled in Restorative Care plus Intervention) exercise project: A model to improve strength, balance, mobility, and decrease fall risk in aging veterans [J]. Journal of the American Medical Directors Association, 2018, 19(3): B23.  [520] LI B, YANG Z, LI Y, et al. Exploration beyond osteoarthritis: the association and mechanism of its related comorbidities [J]. Front Endocrinol (Lausanne), 2024, 15: 1352671.  [521] LI D, MCINTOSH C S, MASTAGLIA F L, et al. Neurodegenerative diseases: a hotbed for splicing defects and the potential therapies [J]. Translational Neurodegeneration, 2021, 10(1).  [522] LI K, KUDO E, KAMATA M, et al. Intravascular large B cell lymphoma (IVLBCL), Asian variant [J]. Brain Pathology, 2010, 20: 67.  [523] LI M, WANG L, LIU J H, et al. Relationships between Rapid Eye Movement Sleep Behavior Disorder and Neurodegenerative Diseases: Clinical Assessments, Biomarkers, and Treatment [J]. Chinese Medical Journal, 2018, 131(8): 966-73.  [524] LICASTRO F, CANDORE G, LIO D, et al. Innate immunity and inflammation in ageing: A key for understanding age-related diseases [J]. Immunity and Ageing, 2005, 2.  [525] LIEWLUCK T, MILONE M, MAUERMANN M L, et al. A novel VCP mutation underlies scapuloperoneal muscular dystrophy and dropped head syndrome featuring lobulated fibers [J]. Muscle Nerve, 2014, 50(2): 295-9.  [526] LIM R, HOH W, RAMOS D. Nurses' Opinions on a Geriatric Care Bundle Intervention: An Experience from a Singapore Community Hospital [J]. Journal of the American Geriatrics Society, 2023, 71: S36.  [527] LIMPHONG P, ZHANG H, CHENG P, et al. Modeling human myofibrillar desmin-related cardiomyopathy with induced pluripotent Stem cells [J]. Circulation, 2011, 124(21).  [528] LISK C. Geriatric medicine teaching in core medicine; where are we currently and where do we need to be [J]. European Geriatric Medicine, 2016, 7: S137.  [529] LIU Y, YU J T, ZONG Y, et al. C9ORF72 mutations in neurodegenerative diseases [J]. Molecular Neurobiology, 2014, 49(1): 386-98.  [530] LIU-AMBROSE T, DONALDSON M G. Exercise and cognition in older adults: is there a role for resistance training programmes? [J]. British journal of sports medicine, 2009, 43(1): 25-7.  [531] LO A C, HOUENOU L J, OPPENHEIM R W. Apoptosis in the nervous system: Morphological features, methods, pathology, and prevention [J]. Archives of Histology and Cytology, 1995, 58(2): 139-49.  [532] LOW Y H, ASI Y, FOTI S C, et al. Heterogeneous Nuclear Ribonucleoproteins: Implications in Neurological Diseases [J]. Molecular Neurobiology, 2021, 58(2): 631-46.  [533] LOWENSTINE L J, MCMANAMON R, TERIO K A. Comparative Pathology of Aging Great Apes: Bonobos, Chimpanzees, Gorillas, and Orangutans [J]. Veterinary pathology, 2016, 53(2): 250-76.  [534] LU J X, WANG Y, ZHANG Y J, et al. Axonal mRNA localization and local translation in neurodegenerative disease [J]. Neural Regeneration Research, 2021, 16(10): 1950-7.  [535] LUDLOW A T, LUDLOW L W, ROTH S M. Do telomeres adapt to physiological stress? Exploring the effect of exercise on telomere length and telomere-related proteins [J]. BioMed Research International, 2013, 2013.  [536] LUND K, VON STEMANN J, HANSEN M, et al. Inflammatory phenotypes and tailored risk evaluation in kidney transplantation [J]. Nephrology Dialysis Transplantation, 2018, 33: i291.  [537] LUNN J S, SAKOWSKI S A, HUR J, et al. Stem cell technology for neurodegenerative diseases [J]. Ann Neurol, 2011, 70(3): 353-61.  [538] LYNCH T, SANO M, MARDER K S, et al. Clinical characteristics of a family with chromosome 17-linked disinhibition-dementia-parkinsonism-amyotrophy complex [J]. Neurology, 2001, 57(10): S39-S45.  [539] MACHIDA Y, TSUCHIYA K, ANNO M, et al. Sporadic amyotrophic lateral sclerosis with multiple system degeneration: a report of an autopsy case without respirator administration [J]. Acta Neuropathol, 1999, 98(5): 512-5.  [540] MAETZLER W, DREY M, JACOBS A H. Sarcopenia and frailty in neurology [J]. Nervenarzt, 2015.  [541] MAHAMMAD S, MURTHY P S, DIDONNA A, et al. Gigaxonin regulates the degradation of intermediate filament proteins: Insights into giant axonal neuropathy [J]. Molecular Biology of the Cell, 2012, 23(24).  [542] MAHER D, AILABOUNI N, MANGONI A A, et al. Alterations in drug disposition in older adults: a focus on geriatric syndromes [J]. Expert Opinion on Drug Metabolism and Toxicology, 2021, 17(1): 41-52.  [543] MAHLON TANYEL M C, MANCANO L D. Neurologic findings in vitamin E deficiency [J]. American Family Physician, 1997, 55(1): 197-201.  [544] MAI A, ROTILI D, VALENTE S, et al. Histone deacetylase inhibitors and neurodegenerative disorders: Holding the promise [J]. Current Pharmaceutical Design, 2009, 15(34): 3940-57.  [545] MAKHOURI F R, GHASEMI J B. In silico studies in drug research against neurodegenerative diseases [J]. Current Neuropharmacology, 2018, 16(6): 664-725.  [546] MAKINO T, UMEGAKI H, HAYASHI T, et al. ApoE epsilon 4 status modifies the effects of aerobic, resistance, or combined training on cognitive function in older adults [J]. Alzheimer's and Dementia, 2017, 13(7): P867.  [547] MAKIZAKO H, NAKAI Y, TOMIOKA K, et al. Prevalence of sarcopenia defined using the Asia working group for sarcopenia criteria in Japanese community-dwelling older adults: A systematic review and meta-analysis [J]. Aging Medicine and Healthcare, 2019, 10: 23.  [548] MALAFARINA V, SUESCUN PUERTA L, BIAIN UGARTE A, et al. Prevalence of sarcopenia in very old adults with high comorbidity [J]. Osteoporosis International, 2020, 31(SUPPL 1): S603.  [549] MALAFARINA V, SUESCUN PUERTA L, BIAIN UGARTE A, et al. Factors associated to incidence and development of sarcopenia in very old people living in the community [J]. Osteoporosis International, 2020, 31(SUPPL 1): S607-S8.  [550] MANDER A M. Better life better health-lifestyle and diet for a healthy future [J]. BJOG: An International Journal of Obstetrics and Gynaecology, 2013, 120: 571.  [551] MANINI A, GAGLIARDI D, ANTOGNOZZI S, et al. Pathogenic HTT and NOTCH2NLC repeat expansions are rare in Italian amyotrophic lateral sclerosis patients [J]. European Journal of Neurology, 2022, 29: 343.  [552] MANTOVANI A, TARGHER G, ZOPPINI G. Nonalcoholic Fatty Liver Disease and Implications for Older Adults with Diabetes [J]. Clinics in Geriatric Medicine, 2020, 36(3): 527-47.  [553] MAQOUD F, SCALA R, HOXHA M, et al. ATP-sensitive Potassium Channel Subunits in Neuroinflammation: Novel Drug Targets in Neurodegenerative Disorders [J]. Cns & Neurological Disorders-Drug Targets, 2022, 21(2): 130-49.  [554] MARIOTTI A, GALLO M L, STRACCI D, et al. Preliminary results of a novel uro-geriatric pathways: How good are the scores of frailty to categorize frailty among elderly patients? [J]. European Urology, 2023, 83: S469.  [555] MARKS A R. Targeting ryanodine receptors to treat human diseases [J]. J Clin Invest, 2023, 133(2).  [556] MARTIN F G, PAPA E, BORGES J, et al. Osteoporosis and fractures: The inattention of non geriatricians with the screening and treatment [J]. Journal of the American Geriatrics Society, 2015, 63: S251-S2.  [557] MARTIN G M. Stochastic modulations of the pace and patterns of ageing: impacts on quasi-stochastic distributions of multiple geriatric pathologies [J]. Mech Ageing Dev, 2012, 133(4): 107-11.  [558] MARTIN L, REDD A, MELVIN D, et al. Fluoroquinolone antibiotics and possible mitochondrial toxicity [J]. Mitochondrion, 2015, 24: S32-S3.  [559] MARTYNENKO A V, SIBELLI N P, ILNITSKI A N. "The blue zones" resilience-diet (review) [J]. Research Results in Biomedicine, 2022, 8(4): 503-15.  [560] MASRORI P, VAN DAMME P. Amyotrophic lateral sclerosis: a clinical review [J]. European Journal of Neurology, 2020, 27(10): 1918-29.  [561] MATHEWSON S L, AZEVEDO P S, GORDON A L, et al. Overcoming protein-energy malnutrition in older adults in the residential care setting: A narrative review of causes and interventions [J]. Ageing Research Reviews, 2021, 70.  [562] MATSUI Y, MATSUI I. Case Report: Nutritional Examination of Weight Loss Treatment Using Kampo [J]. Front Nutr, 2021, 8: 551373.  [563] MATSUMARU D, MOTOHASHI H. The KEAP1-NRF2 System in Healthy Aging and Longevity [J]. Antioxidants (Basel), 2021, 10(12).  [564] MATSUMOTO C, TOMIYAMA H, MATSUURA M, et al. THE ASSOCIATION OF ORAL HYPOFUNCTION AND ARTERIAL STIFFNESS [J]. Journal of Hypertension, 2023, 41: e192.  [565] MATSUMOTO C, TOMIYAMA H, MATSUURA M, et al. The Association of Sarcopenic Obesity and Increased Arterial Stiffness [J]. Circulation, 2022, 146.  [566] MATSUNAGA A, YONEDA M. Dementia due to Endocrine Diseases [J]. Brain and nerve = Shinkei kenkyū no shinpo, 2016, 68(4): 399-405.  [567] MATSUNAGA S, FUJISHIRO H, TAKECHI H. Efficacy and Safety of Cholinesterase Inhibitors for Mild Cognitive Impairment: A Systematic Review and Meta-Analysis [J]. Journal of Alzheimers Disease, 2019, 71(2): 513-23.  [568] MAZZACCARA C, FERRIGNO M, MASULLO M, et al. De novo mitochondrial dna alteration in child with complex neurological compromission [J]. Biochimica Clinica, 2013, 37: S364.  [569] MCCLUSKEY L. Amyotrophic Lateral Sclerosis: Ethical issues from diagnosis to end of life [J]. Neurorehabilitation, 2007, 22(6): 463-72.  [570] MCGANN P E. Comorbidity in heart failure in the elderly [J]. Clinics in Geriatric Medicine, 2000, 16(3): 631-48.  [571] MCMICHAEL G, GIBSON C, MACLENNAN A, et al. Apolipoprotein E genotype is not associated with cerebral palsy [J]. Developmental Medicine and Child Neurology, 2009, 51: 16.  [572] MECOCCI P, BOCCARDI V, CECCHETTI R, et al. A Long Journey into Aging, Brain Aging, and Alzheimer's Disease Following the Oxidative Stress Tracks [J]. Journal of Alzheimer's Disease, 2018, 62(3): 1319-35.  [573] MEGURO T, MEGURO Y, NAGAI T. Sarcopenia in Heart Failure Patients Without Dementia is Associated with Local Brain Atrophy and Predicts Poor Prognosis [J]. Circulation, 2020, 142(SUPPL 3).  [574] MEHRABAN-FAR S, ALRASSI J, MORTENSEN M M. Dysphagia in the geriatric population [J]. Otolaryngology - Head and Neck Surgery, 2019, 161(2): P90.  [575] MELONE M A B. Molecular mechanisms involved in the pathogenesis of Huntington's disease [J]. Clinical Neuropathology, 2013, 32(3): 212.  [576] MENDELL J R, AL-ZAIDY S A, RODINO-KLAPAC L R, et al. Current Clinical Applications of In Vivo Gene Therapy with AAVs [J]. Molecular Therapy, 2021, 29(2): 464-88.  [577] MENERI M, PIGA D, MAGRI F, et al. Clinico-pathologic features and molecular genetic spectrum in a cohort of adult patients with mtDNA maintenance disorders [J]. Acta Myologica, 2023, 42: 50.  [578] MENTIS A F A, BOUGEA A M, CHROUSOS G P. Amyotrophic lateral sclerosis (ALS) and the endocrine system: Are there any further ties to be explored? [J]. Aging Brain, 2021, 1.  [579] MENZEN M. DIABELOOP IN LONG STANDING TYPE 1 DIABETES WITH DEMENTIA [J]. Diabetes Technology and Therapeutics, 2022, 24(SUPPL 1): A47-A8.  [580] MERCHANT R A, HUI R J Y, KWEK S C, et al. Rapid Geriatric Assessment Using Mobile App in Primary Care: Prevalence of Geriatric Syndromes and Review of Its Feasibility [J]. Frontiers in Medicine, 2020, 7.  [581] MESQUITA J, MATOS M J, VARELA A, et al. Pituitary adenoma mimicking a psychiatric disorder - A case report [J]. Endocrine Abstracts, 2010, 22: P433.  [582] MESSINGER-RAPPORT B J, GAMMACK J K, THOMAS D R, et al. Clinical update on nursing home medicine: 2013 [J]. J Am Med Dir Assoc, 2013, 14(12): 860-76.  [583] MICHAEL A, TEO P. Neurological disorders in patients with falls or poor balance [J]. Journal of the American Geriatrics Society, 2014, 62: S210-S1.  [584] MICHAUD M, BALARDY L, MOULIS G, et al. Proinflammatory cytokines, aging, and age-related diseases [J]. Journal of the American Medical Directors Association, 2013, 14(12): 877-82.  [585] MIGNI A, GALLI F, ANTOGNELLI C, et al. The Choko-Age project: development and analytical characterization of a new chocolate product functionalized with vitamin E to combine with physical exercise in preventing malnutrion in pre-dementia eldery subjects [J]. Free Radical Biology and Medicine, 2022, 189: 27.  [586] MILLER C, MCQUAID S E. The Association of Paget's Disease With Inclusion Body Myositis and Fronto Temporal Dementia (IBMFTD) [J]. Journal of the Endocrine Society, 2020, 4: A1120.  [587] MINAGLIA C, GIANNOTTI C, BOCCARDI V, et al. Cachexia and advanced dementia [J]. Journal of Cachexia, Sarcopenia and Muscle, 2019, 10(2): 263-77.  [588] MINN Y K, SUK S H, KOH I S, et al. Increasing skeletal muscle mass could prevent stroke [J]. Neurology, 2016, 86(16).  [589] MIR W A Y, SHRESTHA D B, FIUMARA F, et al. HIV Encephalopathy Mimicking Acute Demyelinating Processes [J]. Cureus, 2021, 13(10): e18494.  [590] MITSUMOTO H, SLIMAN R J, SCHAFER I A, et al. Motor neuron disease and adult hexosaminidase A deficiency in two families: evidence for multisystem degeneration [J]. Ann Neurol, 1985, 17(4): 378-85.  [591] MITSUYAMA Y. [Yuasa-Mitsuyama disease] [J]. Brain Nerve, 2011, 63(2): 109-18.  [592] MITSUYAMA Y, TAKAMATSU I. [Autopsy case of presenile dementia with muscular atrophy] [J]. No To Shinkei, 1971, 23(4): 409-16.  [593] MIWA H, MORI H, SUMINO S, et al. [Neurological CPC.57. An 80-year-old woman with four years history of muscle atrophy involving lower extremities predominantly on the right side] [J]. No To Shinkei, 1997, 49(9): 857-65.  [594] MIWA T, IRITANI O, MORIMOTO S. Olfactory-cognitive index distinguishes involvement of frontal lobe shrinkage, as in sarcopenia from shrinkage of medial temporal areas, and global brain, as in kihon checklist frailty/dependence, in older adults with progression of normal cognition to alzheimer's disease [J]. Chemical Senses, 2021, 46.  [595] MIYAKAWA T, SHIKAI I, SUZUKI T, et al. An autopsy case of multiple leucoencephalitis showing hemiatrophy of the brain [J]. Folia Psychiatr Neurol Jpn, 1979, 33(1): 71-9.  [596] MIZOI Y, YAMAMOTO T, MINAMI N, et al. Oculopharyngeal muscular dystrophy associated with dementia [J]. Intern Med, 2011, 50(20): 2409-12.  [597] MOFATTEH M. Neurodegeneration and axonal mRNA transportation [J]. American Journal of Neurodegenerative Diseases, 2021, 10(1): 1-12.  [598] MOGI M, LIU S, WATANABE R, et al. Perspectives on frailty as a total life-course disease with consideration of the fetal environment [J]. Geriatrics and Gerontology International, 2023, 23(4): 263-9.  [599] MOGLAN M P, ZAMFIR M V, ZAMFIR M. ADDRESSING FRAILTY IN ELDERLY PATIENTS WITH NEUROCOGNITIVE DISORDERS [J]. Alzheimer's and Dementia, 2019, 15(7): P1434-P5.  [600] MOGLIA C, CALVO A, CANOSA A, et al. A case report of a patient with ALS and an obsessive-compulsive disorder carrying expansion of C9ORF72 [J]. European Journal of Neurology, 2012, 19: 281.  [601] MOHASSEL P, DONKERVOORT S, KIM H, et al. NEW GENES IN NEUROMUSCULAR DISEASES: O.04 Heterozygous frameshift variants in hnRNPA2B1 cause a novel oculopharyngodistal muscular dystrophy [J]. Neuromuscular Disorders, 2020, 30: S47.  [602] MOHTASHAMI Z, SINGH M K, SALIMIAGHDAM N, et al. MOTS-c, the Most Recent Mitochondrial Derived Peptide in Human Aging and Age-Related Diseases [J]. International Journal of Molecular Sciences, 2022, 23(19).  [603] MONTALCINI T, PUJIA A, DONINI L M, et al. A call to action: Now is the time to screen elderly and treat osteosarcopenia, a position paper of the italian college of academic nutritionists med/49 (ican-49) [J]. Nutrients, 2020, 12(9): 1-21.  [604] MOON Y, MOON W J, KIM J O, et al. PREDICTORS OF POOR CLINICAL OUTCOME AND ROLE OF MUSCLE PROFILE IN ALZHEIMER'S DISEASE: A 3-YEAR LONGITUDINAL STUDY [J]. Alzheimer's and Dementia, 2019, 15(7): P700-P1.  [605] MORAES M M, SOARES M A, GORJAO CLARA J. Falls of older patients: The influence of drugs [J]. European Geriatric Medicine, 2016, 7: S239.  [606] MORANDI A, DI SANTO S, MOSSELLO E, et al. Calf circumference as a correlate of delirium in hospitalized older people: data from the Italian delirium day 2017 [J]. European Geriatric Medicine, 2018, 9: S317.  [607] MORANO A E V, DORNELES G P, PERES A, et al. The role of glucose homeostasis on immune function in response to exercise: The impact of low or higher energetic conditions [J]. Journal of Cellular Physiology, 2020, 235(4): 3169-88.  [608] MORGAN S, ORRELL R W. Pathogenesis of amyotrophic lateral sclerosis [J]. British Medical Bulletin, 2016, 119(1): 87-97.  [609] MORGAN-HUGHES J A, HAYES D J, CLARK J B, et al. Mitochondrial encephalomyopathies: biochemical studies in two cases revealing defects in the respiratory chain [J]. Brain, 1982, 105 (Pt 3): 553-82.  [610] MORITA K, KAIYA H, IKEDA T, et al. Presenile dementia combined with amyotrophy: A review of 34 Japanese cases [J]. Archives of Gerontology and Geriatrics, 1987, 6(3): 263-77.  [611] MOSKE-EICK O, KOTTLORS M, HAUG V, et al. Late-onset Autosomal dominant limb girdle muscular dystrophy and Paget's disease of bone unlinked to the VCP gene locus [J]. Neuroradiology Journal, 2010, 23: 428.  [612] MUKHERJEE S, MAHESH K V, BHADADA S K, et al. The Role of Genetic Analysis in Demystifying the Diagnosis in a Middle-Aged Male Presenting With Proximal Muscle Weakness and Sclerotic-Lytic Skeletal Lesions [J]. Cureus, 2023, 15(12): e50924.  [613] MULCAHY P J, IREMONGER K, KARYKA E, et al. Gene therapy: A promising approach to treating spinal muscular atrophy [J]. Human Gene Therapy, 2014, 25(7): 575-86.  [614] MüLLER U, GRAEBER M B. Neurogenetic diseases: Molecular diagnosis and therapeutic approaches [J]. Journal of Molecular Medicine, 1996, 74(2): 71-84.  [615] MüLLER U, GRAEBER M B, HABERHAUSEN G, et al. Molecular basis and diagnosis of neurogenetic disorders [J]. Journal of the Neurological Sciences, 1994, 124(2): 119-40.  [616] MUNDI M S, PATEL J, MCCLAVE S A, et al. Current perspective for tube feeding in the elderly: From identifying malnutrition to providing of enteral nutrition [J]. Clinical Interventions in Aging, 2018, 13: 1353-64.  [617] MURAKAMI N. Parkinsonism-dementia complex on Guam - Overview of clinical aspects [J]. Journal of Neurology, Supplement, 1999, 246(2): II16-II8.  [618] MURATA K Y, ITO H. The etiology and pathogenesis of sporadic inclusion body myositis [J]. Brain and Nerve, 2014, 66(11): 1385-94.  [619] MURATA Y, NORIO T, TSUKAMOTO T, et al. An autopsy case of ALS with dementia (ALS-D) who developed jealous delusion and hallucination with rapidly progressed brain atrophy [J]. Neuropathology, 2010, 30(3): 325.  [620] MYINT H, SIMMONS M, DE LA CRUZ J, et al. A NOVEL PRESSURE INJURY CARE BUNDLE FOR DEPENDENT PATIENTS WITH PRESSURE INJURIES IN Bermuda [J]. Age and Ageing, 2023, 52: i19.  [621] NAG N, LIN X, JELINEK G, et al. Identifying Shared Symptoms and Comorbidities to Inform Risk Reduction Strategies Across Prevalent Neurological disorders [J]. International Journal of Epidemiology, 2021, 50: i166-i7.  [622] NAGAI Y. Development of diseasemodifying therapy for polyglutamine-linked SCA [J]. Clinical Neurology, 2020, 60: S201.  [623] NAGAI Y, TOMIOKA I, ISHIBASHI H, et al. Transgenic monkey model of the polyglutamine diseases recapitulating progressive neurological symptoms and polyglutamine protein inclusions [J]. Journal of the Neurological Sciences, 2017, 381: 55.  [624] NAGANO S, ARAKI T. Axonal Transport and Local Translation of mRNA in Neurodegenerative Diseases [J]. Frontiers in Molecular Neuroscience, 2021, 14.  [625] NAGASAWA C K, GARCIA-BLANCO M A. Early Splicing Complexes and Human Disease [J]. Int J Mol Sci, 2023, 24(14).  [626] NAHARCI M I, KARADAG B, SAHIN S, et al. Low vitamin D level in patients with alzheimer's disease (devit-alz): A crosssectional multicenter study [J]. Osteoporosis International, 2013, 24(1): S127-S8.  [627] NAJAFZADEH M, MOHAMMADIAN F, MIRABIAN S, et al. Rapid eye movement sleep behavior disorder and its relation to Parkinson's disease: The potential of graph measures as brain biomarkers to identify the underlying physiopathology of the disorder [J]. Brain and Behavior, 2024, 14(3).  [628] NAKAHARA S, TAKASAKI M, ABE S, et al. Aggressive nutrition therapy in malnutrition and sarcopenia [J]. Nutrition, 2021, 84.  [629] NAKAMURA K, YOSHIDA K, YOSHINAGA T, et al. Adult or late-onset triple A syndrome Case report and literature review [J]. Journal of the Neurological Sciences, 2010, 297(1-2): 85-8.  [630] NAKAMURA M, UEKI S, KUBO M, et al. Two cases of sporadic adult-onset neuronal intranuclear inclusion disease preceded by urinary disturbance for many years [J]. J Neurol Sci, 2018, 392: 89-93.  [631] NAKAMURA M, UEKI S, KUBO M, et al. Two cases of neuronal intranuclear inclusion disease preceded by urinary disturbance for many years [J]. Clinical Neurology, 2019, 59: S427.  [632] NALBANDIAN A, DONKERVOORT S, DEC E, et al. The multiple faces of valosin-containing protein-associated diseases: Inclusion body myopathy with Paget's disease of bone, frontotemporal dementia, and amyotrophic lateral sclerosis [J]. Journal of Molecular Neuroscience, 2011, 45(3): 522-31.  [633] NARULA J K, SHIVARAJ K, KIM A Y, et al. An interesting case of non-anion gap metabolic acidosis secondary to arginine hydrochloride infusion therapy [J]. Journal of the American Society of Nephrology, 2021, 32: 811.  [634] NATIONAL CLINICAL GUIDELINE C. National Institute for Health and Care Excellence: Clinical Guidelines [M]. Motor Neurone Disease: Assessment and Management. London; National Institute for Health and Care Excellence (UK)  Copyright © National Clinical Guideline Centre, 2016. 2016.  [635] NELSON M, ZHANG X, PAN Z, et al. Mast cell effects on esophageal smooth muscle and their potential role in eosinophilic esophagitis and achalasia [J]. American Journal of Physiology-Gastrointestinal and Liver Physiology, 2021, 320(3): G319-G27.  [636] NEMETH Z, GRANGER J P, RYAN M J, et al. Is there a role of proinflammatory cytokines on degenerin-mediated cerebrovascular function in preeclampsia? [J]. Physiological Reports, 2022, 10(13).  [637] NICHOLSON S J, WITHERDEN A S, HAFEZPARAST M, et al. Mice, the motor system, and human motor neuron pathology [J]. Mammalian Genome, 2000, 11(12): 1041-52.  [638] NIELSEN J E, JENSEN L N, KRABBE K. Hereditary haemochromatosis: a case of iron accumulation in the basal ganglia associated with a parkinsonian syndrome [J]. J Neurol Neurosurg Psychiatry, 1995, 59(3): 318-21.  [639] NIEMAN D C. Clinical implications of exercise immunology [J]. Journal of Sport and Health Science, 2012, 1(1): 12-7.  [640] NIEMEYER-GUIMARãES M, PIVATELLI R. Shared Decision-making in Feeding Strategy for Late-stage Dementia Patients Living in Long-term Assisted-living Institutions for the Elderly (LT/AL): Integration of Early Palliative Care (PC) [J]. Palliative Medicine, 2023, 37(1): 128.  [641] NIKITINA E, DOLGAYA Y, MEDVEDEVA A, et al. Micro-RNAs-regulators of signal cascade of actin remodeling as biomarkers of neurodegenerative disorders and triggers of stress response [J]. Neurodegenerative Diseases, 2015, 15: 1243.  [642] NIMITPHONG H, HOLICK M F. Vitamin D, neurocognitive functioning and immunocompetence [J]. Curr Opin Clin Nutr Metab Care, 2011, 14(1): 7-14.  [643] NISHAL S, PHAUGAT P, BAZAAD J, et al. A Concise Review of Common Plant-derived Compounds as a Potential Therapy for Alzheimer's Disease and Parkinson's Disease: Insight into Structure-Activity-Relationship [J]. Cns & Neurological Disorders-Drug Targets, 2023, 22(7): 1057-69.  [644] NISHIHIRA Y, FU Y J, TAN C F, et al. An autopsy case of sporadic four-repeat tauopathy with dementia, parkinsonism and motor neuron disease [J]. Neuropathology, 2009, 29(3): 370.  [645] NISHIKAWA H, ASAI A, FUKUNISHI S, et al. Screening tools for sarcopenia [J]. In Vivo, 2021, 35(6): 3001-9.  [646] NISHIMURA H, ITO T, FUKAI Y. An autopsy case of sporadic amyotrophic lateral sclerosis with numerous pTDP-43 positive inclusions [J]. Neuropathology, 2011, 31(3): 324.  [647] NISHINAKA T, KURODA S, HAYASHI Y, et al. [Motor neuron disease with Parkinson's disease--case report] [J]. Rinsho Shinkeigaku, 1990, 30(11): 1252-5.  [648] NITOBE S, YAMADA S, KONO Y, et al. Lower extremity muscle weakness after cardiovascular surgery is related to the combined effect among promoting factors of muscle protein catabolism [J]. Physiotherapy (United Kingdom), 2011, 97: eS895.  [649] NOURHASHéMI F, ROLLAND Y, VELLAS B. Prevention of falls and their consequences [J]. Presse Medicale, 2000, 29(22): 1249-54.  [650] O'CONNOR D M, BOULIS N M. Gene therapy for neurodegenerative diseases [J]. Trends in Molecular Medicine, 2015, 21(8): 504-12.  [651] O'NEILL M, DUFFY O, HENDERSON M, et al. Identification of eating, drinking and swallowing difficulties for people living with early-stage dementia: A systematic review [J]. International journal of language & communication disorders, 2023, 58(6): 1994-2007.  [652] OAKES J A, DAVIES M C, COLLINS M O. TBK1: a new player in ALS linking autophagy and neuroinflammation [J]. Mol Brain, 2017, 10(1): 5.  [653] OAKLEY H, JARRETT P, MCCLOSKEY R. The relationship between dementia and falling in long-stay hospital patients [J]. American Journal of Geriatric Psychiatry, 2013, 21(3): S147.  [654] OBA N, FUJIMOTO Y, HIRATA K, et al. [A case of Gerstmann-Sträussler-Scheinker disease with severe muscular atrophy and vertical gaze palsy] [J]. Rinsho Shinkeigaku, 2000, 40(7): 726-31.  [655] OBIS T, MERWIN S, DZIEDZIC J, et al. Unraveling common mechanisms between manganese neurotoxicity and ALS-related motor neuron death [J]. Amyotrophic Lateral Sclerosis and Frontotemporal Degeneration, 2015, 16: 186.  [656] OCHARáN-MERCADO A, LOAEZA-LOAEZA J, CASTRO-CORONEL Y, et al. RNA-Binding Proteins: A Role in Neurotoxicity? [J]. Neurotoxicity Research, 2023, 41(6): 681-97.  [657] OCHI M, KOHARA K, TABARA Y, et al. Thigh muscle mass decline was associated brain small vessel diseases in men; possible link between sarcopenia and dementia [J]. Neurology, 2012, 78(1).  [658] OGAMA N, SAKURAI T, KAWASHIMA S, et al. Association between glucose management and frailty in older adults with type 2 diabetes mellitus [J]. European Geriatric Medicine, 2019, 10: S275.  [659] OHKOSHI K, ISHIDA N, YAMAGUCHI T, et al. Corneal endothelium in a case of mitochondrial encephalomyopathy (Kearns-Sayre syndrome) [J]. Cornea, 1989, 8(3): 210-4.  [660] OHNISHI K, HIRAOKA A, MURAKAMI T, et al. HANDGRIP STREGTH DICLINE IN CHRONIC LIVER DISEASE PATIENTS INDICTES INCREASED RISK OG FALLING [J]. Gastroenterology, 2020, 158(6): S-610.  [661] OHNO K. RNA pathologies in neurological disorders [J]. Clinical Neurology, 2007, 47(11): 801-4.  [662] OHOKA T, URABE Y, SHIRAKAWA T. Therapeutic exercises for proximal femoral fracture of super-aged patients: Effect of walking assistance using body weight-supported treadmill training (BWSTT) [J]. Physiotherapy (United Kingdom), 2015, 101: eS1124-eS5.  [663] OHTA Y, NOMURA E, HATANAKA N, et al. Strong associations of sarcopenia and frailty with cognitive functions in female MCI and AD [J]. Clinical Neurology, 2020, 60: S461.  [664] OKAMOTO K, LLENA J F, HIRANO A. A type of adult polyglucosan body disease [J]. Acta Neuropathol, 1982, 58(1): 73-7.  [665] OKAMOTO T, ISHIHARA T, MIYAZAKI M, et al. Clinical Diversity of Patients with Neuronal Intranuclear Inclusion Disease [J]. Neurology, 2022, 98(18 SUPPL).  [666] OKAWA Y. The Impact of Nutritional Therapy on Gastrointestinal Motility in Older Adults [J]. Healthcare (Basel), 2023, 11(21).  [667] OKETA Y, ONO S. Familial amyotrophic lateral sclerosis with GLY93SER mutation in CU/ZN superoxide dismutase: A clinical and neuropathological study [J]. Amyotrophic Lateral Sclerosis, 2011, 12: 150-1.  [668] OKOUCHI M, EKSHYYAN O, MARACINE M, et al. Neuronal apoptosis in neurodegeneration [J]. Antioxidants and Redox Signaling, 2007, 9(8): 1059-96.  [669] OLAYINKA O, OLAYINKA O O, ALEMU B T, et al. Toxic Environmental Risk Factors for Alzheimer's Disease: A Systematic Review [J]. Aging Medicine and Healthcare, 2019, 10(1): 4-17.  [670] OLESNICKY E C, WRIGHT E G. Drosophila as a Model for Assessing the Function of RNA-Binding Proteins during Neurogenesis and Neurological Disease [J]. J Dev Biol, 2018, 6(3).  [671] OLIVEIRA SANTOS M, GROMICHO M, PRONTO-LABORINHO A, et al. Sporadic Spinal-Onset Amyotrophic Lateral Sclerosis Associated with Myopathy in Three Unrelated Portuguese Patients [J]. Brain Sci, 2023, 13(2).  [672] OMER T, NASSEROLESLAMI B, MOLLOY F, et al. A longitudinal motor unit number index (MUNIX) Estimation study in Frontotemporal Dementia (FTD) [J]. European Journal of Neurology, 2018, 25: 245.  [673] OR O, LANE J M, HALAWA O, et al. Does promis29 correlate with frailty in hip fracture patients? [J]. Journal of Bone and Mineral Research, 2017, 32: S287.  [674] ORMROD D, SPENCER C. Metrifonate: A review of its use in Alzheimer's disease [J]. CNS Drugs, 2000, 13(6): 443-67.  [675] ORRELL R W. Genetics of motor neurone diseases [J]. European Journal of Neurology, 2015, 22: 855.  [676] ORTIZ G G, HUERTA M, GONZáLEZ-USIGLI H A, et al. Cognitive disorder and dementia in type 2 diabetes mellitus [J]. World Journal of Diabetes, 2022, 13(4): 319-37.  [677] OTA H. Sir2 gene [J]. Nippon rinsho Japanese journal of clinical medicine, 2009, 67(7): 1289-92.  [678] OTA K, HASEGAWA R, SHICHIHYO K, et al. MON-115 THE TOTAL MANAGEMENT OF AGED CHRONIC HEMODIALYSIS PATIENTS WITH COGNITIVE IMPAIRMENTS [J]. Kidney International Reports, 2019, 4(7): S351.  [679] OTA S, TSUCHIYA K. [Pure akinesia presenting with antecollis] [J]. No To Shinkei, 2005, 57(10): 893-8.  [680] OTSUKA R. Preventive dietary factors for frailty, sarcopenia, and dementia; Results from NILS-LSA cohort study [J]. Annals of Nutrition and Metabolism, 2023, 79: 103-4.  [681] OUDBIER S J, GOH J, LOOIJAARD S, et al. Pathophysiological Mechanisms Explaining the Association Between Low Skeletal Muscle Mass and Cognitive Function [J]. Journals of Gerontology Series a-Biological Sciences and Medical Sciences, 2022, 77(10): 1959-68.  [682] OZAWA K, SAIDA K, SAIDA T, et al. Familial neuropathy with dementia, retinitis pigmentosa, and dysautonomia [J]. Neurology, 1985, 35(8): 1184-7.  [683] PACIFICO J, GEERLINGS M A J, REIJNIERSE E M, et al. Prevalence of sarcopenia as a comorbid disease: A systematic review and meta-analysis [J]. Experimental Gerontology, 2020, 131.  [684] PACK K, WALSH A, RAKOCEVIC G. Hereditary inclusion body myopathy without PAGET disease and frontotemporal dementia associated with valosin-containing protein mutation: A case report [J]. Neurology, 2016, 86(16).  [685] PAGANELLI R, DI IORIO A, CHERUBINI A, et al. Frailty of older age: The role of the endocrine - Immune interaction [J]. Current Pharmaceutical Design, 2006, 12(24): 3147-59.  [686] PAGANELLI R, DI IORIO A, CHERUBINI A, et al. Frailty of older age: the role of the endocrine--immune interaction [J]. Curr Pharm Des, 2006, 12(24): 3147-59.  [687] PAHLAVANI H A. Exercise therapy to prevent and treat Alzheimer's disease [J]. Frontiers in Aging Neuroscience, 2023, 15.  [688] PANCHERI E, TESTI S, SQUINTANI G, et al. Inclusion body myopathy mistaken for amyotrophic lateral sclerosis: Report on a family [J]. Acta Myologica, 2016, 35(1): 61.  [689] PANOURGIA M P. The role of the geriatrician in the management of fragility fractures: The UK experience [J]. Journal of Musculoskeletal Neuronal Interactions, 2021, 21(1): 180-1.  [690] PAPACHRISTOU E, RAMSAY S E, LENNON L T, et al. BODY COMPOSITION MEASURES AND COGNITIVE FUNCTIONING IN OLDER AGE: RESULTS FROM A CROSS-SECTIONAL STUDY IN OLDER BRITISH MEN [J]. Journal of Epidemiology and Community Health, 2015, 69: A17.  [691] PAPASTERGIOS C, CHLOPICKI B, BERGELIN-AXELSSON M. Amyotrophic lateral sclerosis (ALS) in patient with dermatomyositis [J]. European Journal of Neurology, 2017, 24: 292.  [692] PARAMBI D G T, ALHARBI K S, KUMAR R, et al. Gene Therapy Approach with an Emphasis on Growth Factors: Theoretical and Clinical Outcomes in Neurodegenerative Diseases [J]. Molecular Neurobiology, 2022, 59(1): 191-233.  [693] PARé B, DUPRé N, GOULD P, et al. Histopathological findings in an adult down syndrome patient presenting with ALS [J]. Amyotrophic Lateral Sclerosis and Frontotemporal Degeneration, 2016, 17: 180.  [694] PARK L, AREVALO J A, BROOKS G A. Oxygen Consumption Rates Between Skeletal Muscle Cells Derived From Young and Old Human Donors Elucidate Mitochondrial Dysfunction [J]. Journal of the American Geriatrics Society, 2023, 71: S297.  [695] PASTOR OCHOA C, HERRAIZ J, MORAGO M, et al. Strategies to prevent falls and fractures in care homeless and effect of cognitive impairment and sarcopenia [J]. European Geriatric Medicine, 2011, 2: S92.  [696] PATAI R, NóGRáDI B, MESZLéNYI V, et al. Calciumion is a common denominator in the pathophysiological processes of amyotrophic lateral sclerosis [J]. Ideggyogyaszati Szemle, 2017, 70(7-8): 247-57.  [697] PAUL S, VáZQUEZ L A B, URIBE S P, et al. Current status of microrna-based therapeutic approaches in neurodegenerative disorders [J]. Cells, 2020, 9(7): 1-26.  [698] PAZ M L, BARRANTES F J. Autoimmune Attack of the Neuromuscular Junction in Myasthenia Gravis: Nicotinic Acetylcholine Receptors and Other Targets [J]. ACS Chemical Neuroscience, 2019, 10(5): 2186-94.  [699] PELLEGRINO L, SANSONE G, BISOGNO A, et al. POST COVID-19 BELLY DANCER'S DYSKINESIA [J]. Neurological Sciences, 2022, 43: S331.  [700] PENA S A, IYENGAR R, ESHRAGHI R S, et al. Gene therapy for neurological disorders: challenges and recent advancements [J]. Journal of Drug Targeting, 2020, 28(2): 111-28.  [701] PEREIRA A G, MASSADA M, SOUSA R, et al. Cemented total hip arthroplasty in “high-risk of dislocation patients” [J]. HIP International, 2010, 20(3): 396.  [702] PERIC S, GUNJIC I, SALAK-DJOKIC B, et al. Cognitive function assessment in patients with myotonic dystrophy type 2 [J]. Journal of the Neurological Sciences, 2021, 429.  [703] PERRONE B, LA COGNATA V, SPROVIERI T, et al. Alternative Splicing of ALS Genes: Misregulation and Potential Therapies [J]. Cellular and Molecular Neurobiology, 2020, 40(1).  [704] PéTERMANS J. Physical exercise in the aged [J]. Revue médicale de Liège, 2001, 56(4): 223-7.  [705] PETROV K. Macrocyclic derivatives of 6-methyluracil: New ligands of the peripheral anionic site of acetylcholinesterase [J]. International Journal of Risk and Safety in Medicine, 2015, 27: S72-S3.  [706] PETZOLD A. The 2022 Lady Estelle Wolfson lectureship on neurofilaments [J]. Journal of Neurochemistry, 2022, 163(3): 179-219.  [707] PEYER A K, KINTER J, FRANK S, et al. Novel valosin containing protein mutation in a Swiss family with hereditary inclusion body myopathy, paget's disease of the bone and dementia [J]. Schweizer Archiv fur Neurologie und Psychiatrie, 2010, 161(4): 35S.  [708] PHADKE M, LOKESHWAR M R, BHUTADA S, et al. Kearns Sayre Syndrome--case report with review of literature [J]. Indian J Pediatr, 2012, 79(5): 650-4.  [709] PHAM X, SIRIRATNAM P, RODRIGUES E, et al. Immune myopathy with perimysial pathology in a patient with an unusual clinical phenotype and Anti-Mi-2 antibody [J]. Neuromuscular Disorders, 2023, 33: S93.  [710] PLANAS VILà M. [Nutritional and metabolic aspects of neurological diseases] [J]. Nutr Hosp, 2014, 29 Suppl 2: 3-12.  [711] PLOTKIN L I. Triggering Receptor Expressed on Myeloid Cells 2 (TREM2) Mutations: a Potential Common Cause of Alzheimer's Disease and Musculoskeletal Disorders [J]. FASEB Journal, 2019, 33(SUPPL 1): 15.1.  [712] PLUDOWSKI P, HOLICK M F, PILZ S, et al. Vitamin D effects on musculoskeletal health, immunity, autoimmunity, cardiovascular disease, cancer, fertility, pregnancy, dementia and mortality-A review of recent evidence [J]. Autoimmunity Reviews, 2013, 12(10): 976-89.  [713] PORTARO S, CACCIOLA A, NARO A, et al. A case report of recessive myotonia congenita and early onset cognitive impairment: Is it a causal or casual link? [J]. Medicine (Baltimore), 2018, 97(22): e10785.  [714] PORTET F, TOUCHON J. REM Sleep behavioral disorder [J]. Revue Neurologique, 2002, 158(11): 1049-56.  [715] PRADAT P F, BRUNETEAU G. Clinical characteristics of ALS subsets [J]. Revue Neurologique, 2006, 162(HS2): 4S29-4S33.  [716] PRADAT P F, BRUNETEAU G. Clinical characteristics of amyotrophic lateral sclerosis subsets [J]. Revue neurologique, 2006, 162 Spec No 2: 4S29-4S33.  [717] PRASAD E M, HUNG S Y. Behavioral Tests in Neurotoxin-Induced Animal Models of Parkinson's Disease [J]. Antioxidants, 2020, 9(10).  [718] PRATESI A, TARANTINI F, DI BARI M. Skeletal muscle: An endocrine organ [J]. Clinical Cases in Mineral and Bone Metabolism, 2013, 10(1): 11-4.  [719] PRATHER R S, LORSON M, ROSS J W, et al. Genetically engineered pig models for human diseases [J]. Annual review of animal biosciences, 2013, 1: 203-19.  [720] PRATT W B, GESTWICKI J E, OSAWA Y, et al. Targeting Hsp90/Hsp70-based protein quality control for treatment of adult onset neurodegenerative diseases [Z]. 2015: 353-71.10.1146/annurev-pharmtox-010814-124332  [721] PRATT W B, GESTWICKI J E, OSAWA Y, et al. Targeting Hsp90/Hsp70-based protein quality control for treatment of adult onset neurodegenerative diseases [J]. Annu Rev Pharmacol Toxicol, 2015, 55: 353-71.  [722] PRATT W B, GESTWICKI J E, OSAWA Y, et al. Targeting Hsp90/Hsp70-Based Protein Quality Control for Treatment of Adult Onset Neurodegenerative Diseases [M]//INSEL P A. Annual Review of Pharmacology and Toxicology, Vol 55. 2015: 353-71.  [723] PRATT W B, MORISHIMA Y, GESTWICKI J E, et al. A model in which heat shock protein 90 targets protein-folding clefts: rationale for a new approach to neuroprotective treatment of protein folding diseases [J]. Exp Biol Med (Maywood), 2014, 239(11): 1405-13.  [724] PRESA J L, SARAVIA F, BAGI Z, et al. Vasculo-Neuronal Coupling and Neurovascular Coupling at the Neurovascular Unit: Impact of Hypertension [J]. Frontiers in Physiology, 2020, 11.  [725] PRICE D L, CORK L C, STRUBLE R G, et al. Dysfunction and death of neurons in human degenerative neurological diseases and in animal models [J]. Ciba Foundation symposium, 1987, 126: 30-48.  [726] PRICE D L, WONG P C, BORCHELT D R, et al. Amyotrophic lateral sclerosis and Alzheimer disease. Lessons from model systems [J]. Rev Neurol (Paris), 1997, 153(8-9): 484-95.  [727] PRIFTI-KURTI M. AN IMPORTANT ROLE OF APCA IN AUTOIMMUNE ATROPHIC GASTRITIS AFTER H. PYLORI INFECTION [J]. Clinical Chemistry and Laboratory Medicine, 2023, 61: S425.  [728] PROCACCIO V, BRIS C, CHAO DE LA BARCA J M, et al. Perspectives of drug-based neuroprotection targeting mitochondria [J]. Rev Neurol (Paris), 2014, 170(5): 390-400.  [729] PRUDLO J, KRESS W, LEDIG S, et al. FUS immunoreactive basophilic inclusion body disease (BIBD) without FUS mutation in a case of spinal and bulbar muscular atrophy (Kennedy's Disease) [J]. Dementia and Geriatric Cognitive Disorders, 2010, 30: 42.  [730] PULST S M. [Antisense therapies for neurological diseases] [J]. Nervenarzt, 2019, 90(8): 781-6.  [731] PURANIK N, YADAV D, CHAUHAN P S, et al. Exploring the role of gene therapy for neurological disorders [J]. Current Gene Therapy, 2021, 21(1): 11-22.  [732] QU Y, LIU Y, NOOR A F, et al. Characteristics and advantages of adeno-associated virus vector-mediated gene therapy for neurodegenerative diseases [J]. Neural Regen Res, 2019, 14(6): 931-8.  [733] QUESNELL T, DIMACHKIE M, PASNOOR M, et al. Utility of screening for monoclonal gammopathy in ALS [J]. Neurology, 2015, 84.  [734] RAJALINGAM S, KAMBRATH A V, VIDAL R, et al. Deletion of the Microtubule-associated protein tau (Mapt-/-) results in diastolic heart failure and altered skeletal muscle function in vivo [J]. FASEB Journal, 2020, 34(SUPPL 1).  [735] RAMAKRISHNA K, NALLA L V, NARESH D, et al. WNT-β Catenin Signaling as a Potential Therapeutic Target for Neurodegenerative Diseases: Current Status and Future Perspective [J]. Diseases, 2023, 11(3).  [736] RANI N, ALAM M M, JAMAL A, et al. Caenorhabditis elegans: A transgenic model for studying age-associated neurodegenerative diseases [J]. Ageing Res Rev, 2023, 91: 102036.  [737] RAO M V, NIXON R A. Defective neurofilament transport in mouse models of amyotrophic lateral sclerosis: A review [J]. Neurochemical Research, 2003, 28(7): 1041-7.  [738] RAPIN I, WEIDENHEIM K, LINDENBAUM Y, et al. Cockayne syndrome in adults: review with clinical and pathologic study of a new case [J]. J Child Neurol, 2006, 21(11): 991-1006.  [739] RASHID S, DIMITRIADI M. Autophagy in spinal muscular atrophy: from pathogenic mechanisms to therapeutic approaches [J]. Frontiers in Cellular Neuroscience, 2023, 17.  [740] RATTAN S I S, GONZALEZ-DOSAL R, NIELSEN E R, et al. Slowing down aging from within: Mechanistic aspects of anti-aging hormetic effects of mild heat stress on human cells [J]. Acta Biochimica Polonica, 2004, 51(2): 481-92.  [741] RAVANIDIS S, KATTAN F G, DOXAKIS E. Unraveling the pathways to neuronal homeostasis and disease: Mechanistic insights into the role of RNA-binding proteins and associated factors [J]. International Journal of Molecular Sciences, 2018, 19(8).  [742] RAVITS J. Focality, stochasticity and neuroanatomic propagation in ALS pathogenesis [J]. Experimental Neurology, 2014, 262(Part B): 121-6.  [743] REES M. Short and long term effects of menopause [J]. Maturitas, 2019, 124: 112.  [744] REGO DE FIGUEIREDO I, SALDANHA M, GONçALVES C, et al. NUTRITIONAL EVALUATION IN A GERIATRIC CLINIC [J]. Clinical Nutrition, 2019, 38: S74.  [745] REIS M, CEBOLA M. The Association Between Dementia And Nutritional Status In Elderly [J]. Clinical Nutrition ESPEN, 2023, 54: 688.  [746] REIS S D, PINHO B R, OLIVEIRA J M A. Modulation of Molecular Chaperones in Huntington’s Disease and Other Polyglutamine Disorders [J]. Molecular Neurobiology, 2017, 54(8): 5829-54.  [747] REY F, BERARDO C, MAGHRABY E, et al. Redox Imbalance in Neurological Disorders in Adults and Children [J]. Antioxidants (Basel), 2023, 12(4).  [748] REZUȘ E, CARDONEANU A, BURLUI A, et al. The link between inflammaging and degenerative joint diseases [J]. International Journal of Molecular Sciences, 2019, 20(3).  [749] RIDLER C, MIZIELINSKA S, CLAYTON E, et al. Generation of cell culture models for investigating disease mechanisms in C9orf72 frontotemporal dementia and amyotrophic lateral sclerosis [J]. American Journal of Neurodegenerative Diseases, 2014, 3: 169.  [750] RIEUCAU A, GMIZ M, FROMENTIN I, et al. Who wants to age successfully? a descriptive study of aging subjects who seek help to deal with their frailty [J]. Alzheimer's and Dementia, 2014, 10: P753.  [751] RIZZO F, RIBOLDI G, SALANI S, et al. Cellular therapy to target neuroinflammation in amyotrophic lateral sclerosis [J]. Cellular and Molecular Life Sciences, 2014, 71(6): 999-1015.  [752] ROBERTS S B, SILVER R E, DAS S K, et al. Healthy Aging-Nutrition Matters: Start Early and Screen Often [J]. Advances in nutrition (Bethesda, Md), 2021, 12(4): 1438-48.  [753] ROBERTSON G S, CROCKER S J, NICHOLSON D W, et al. Neuroprotection by the inhibition of apoptosis [J]. Brain Pathology, 2000, 10(2): 283-92.  [754] RODRIGUEZ K, BUFFENSTEIN R. High levels of the small chaperone HSP25 in naked mole-rats may be a determinant of rodent longevity [J]. FASEB Journal, 2014, 28(1).  [755] RONDANELLI M, FALIVA M A, PERONI G, et al. Focus on Pivotal Role of Dietary Intake (Diet and Supplement) and Blood Levels of Tocopherols and Tocotrienols in Obtaining Successful Aging [J]. Int J Mol Sci, 2015, 16(10): 23227-49.  [756] ROSENBLOOM A L. Mecasermin (recombinant human insulin-like growth factor I) [J]. Advances in Therapy, 2009, 26(1): 40-54.  [757] RUAN Q, D’ONOFRIO G, SANCARLO D, et al. Emerging biomarkers and screening for cognitive frailty [J]. Aging Clinical and Experimental Research, 2017, 29(6): 1075-86.  [758] RUGGIERO M, CALVELLO R, PORRO C, et al. Neurodegenerative Diseases: Can Caffeine Be a Powerful Ally to Weaken Neuroinflammation? [J]. International Journal of Molecular Sciences, 2022, 23(21).  [759] RUSSO F B, CUGOLA F R, FERNANDES I R, et al. Induced pluripotent stem cells for modeling neurological disorders [J]. World J Transplant, 2015, 5(4): 209-21.  [760] SAGI O, WOLFSON M, UTKO N, et al. p66ShcA and ageing: Modulation by longevity-promoting agent aurintricarboxylic acid [J]. Mechanisms of Ageing and Development, 2005, 126(2): 249-54.  [761] SAHASHI K, HASHIZUME A, SOBUE G, et al. Progress toward the development of treatment of spinal and bulbar muscular atrophy [J]. Expert Opinion on Orphan Drugs, 2017, 5(6): 503-14.  [762] SAITO T, HOSODA M, AOTO K, et al. [An unusual case of peroneal muscular atrophy with rigidity, polyneuropathy, mental retardation, and diabetes mellitus developed in familial Parkinson's disease] [J]. Rinsho Shinkeigaku, 1995, 35(8): 878-83.  [763] SAITOH Y, IMABAYASHI E, MUKAI T, et al. 18F-THK5351-PET Imaging Visualizes Neurodegenerative Changes in Neurodegenerative Diseases [J]. Clinical Neurology, 2021, 61: S394.  [764] SAKA B, DOGAN H, KARISIK E, et al. Sarcopenia measured with SARC-F is associated with other care problems in nursing homes: Results of the annual LPZ Study [J]. European Geriatric Medicine, 2019, 10: S284.  [765] ŞAKUL A A S. Mineral balance in neurodegenerative diseases [J]. Journal of Cellular Neuroscience and Oxidative Stress, 2023, 15: 9.  [766] SAMARAS N, SAMARAS D, FRANGOS E, et al. A review of age-related dehydroepiandrosterone decline and its association with well-known geriatric syndromes: Is treatment beneficial? [J]. Rejuvenation Research, 2013, 16(4): 285-94.  [767] SANCHEZ J L S, HE L, GIUDICI K, et al. CIRCULATING LEVELS OF APELIN, GDF-15 AND SARCOPENIA: LACK OF ASSOCIATION IN THE MAPT STUDY [J]. Journal of Nutrition, Health and Aging, 2022, 26(4): 476.  [768] SANCHEZ-GARCIA E, MONTERO-ERRASQUIN B, IGLESIAS-GALINSO A, et al. SARC-Za study: Evaluation of different recruitment methods for participation in a non-pharmacological multicomponent intervention for the treatment of sarcopenia in people over 65 years in Zamora (Spain) [J]. European Geriatric Medicine, 2022, 13: S96.  [769] SANFORD A M, BERG-WEGER M, LITTLE M, et al. The prevalence of geriatric syndromes as captured by the rapid geriatric assessment [J]. Journal of the American Geriatrics Society, 2019, 67: S53.  [770] SAREEN D, SVENDSEN C N. Disease modeling using human IPS cells [J]. Reproductive BioMedicine Online, 2010, 20: S70.  [771] SASAKI S. [Phenotypes in ALS--clinical features and pathology] [J]. Brain Nerve, 2007, 59(10): 1013-21.  [772] SAYERS K, KING L, RYAN S, et al. Prevalence of probable sarcopenia and associated factors in older adults undergoing Comprehensive Geriatric Assessment [J]. Age and Ageing, 2023, 52: iii27.  [773] SCHIMKE N, KRAMPFL K, PETRI S, et al. [Cerebellar symptoms in motor neuron diseases. Special form of amyotrophic lateral sclerosis plus syndrome] [J]. Nervenarzt, 2002, 73(8): 751-3.  [774] SCHUCHMAN E. Acid ceramidase: One gene, one enzyme, and multiple phenotypes [J]. Journal of Inborn Errors of Metabolism and Screening, 2017, 5: 323.  [775] SCHWARTZ J L, JONES K L, YEO G W. Repeat RNA expansion disorders of the nervous system: post-transcriptional mechanisms and therapeutic strategies [J]. Critical Reviews in Biochemistry and Molecular Biology, 2021, 56(1): 31-53.  [776] SCHWEITZER K J, BOYLAN K B, WIDER C W, et al. Parkinsonism (P), Motor Neuron Disease (MND), and Dementia (D): Clinical and pathological studies [J]. Annals of Neurology, 2009, 66: S51-S2.  [777] SCODITTI U, GEMIGNANI F, COLONNA F, et al. Peroneal muscular atrophy with parkinsonism, ptosis, and congenital strabismus [J]. Acta Neurol Scand, 1993, 88(4): 251-3.  [778] SCOLES D R, MINIKEL E V, PULST S M. Antisense oligonucleotides: A primer [J]. Neurol Genet, 2019, 5(2): e323.  [779] SCOLES D R, MINIKEL E V, PULST S M. Antisense oligonucleotides [J]. Neurology-Genetics, 2019, 5(2).  [780] SEFEROVIĆ P M. Introduction to the special issue entitled 'Heart failure management of the elderly patient: focus on frailty, sarcopenia, cachexia, and dementia' [J]. European Heart Journal, Supplement, 2019, 21: L1-L3.  [781] SEITZ D, GILL S S, GRUNEIR A, et al. Effects of cholinesterase inhibitors on postoperative outcomes of older adults with hip fractures and dementia [J]. American Journal of Geriatric Psychiatry, 2010, 18(3): S21.  [782] SELCUK N A, FENERCIOGLU A. Reduction of Glucose Metabolism in Basal Ganglia Diagnosed With FDG-PET Scan <i>A Neuroacanthocytosis Case</i> [J]. Clinical Nuclear Medicine, 2010, 35(7): 557-8.  [783] SELMAN C, SWINDELL W R. Putting a strain on diversity [J]. EMBO Journal, 2018, 37(22).  [784] ŞENADIM S. Use of oral nutritional supplements in amyotrophic lateral sclerosis [J]. Turk Beyin Damar Hastaliklar Dergisi, 2019, 25: 42-5.  [785] SENDA K, SATAKE S, KONDO I, et al. Frailty of copd patients at the pulmonary rehabilitation clinic: An exploratory research in validity of the Kihon Checklist (KCL) in patients with chronic comorbidities for the registry study at the frailty prevention clinic in national center for geriatrics and gerontology, Japan [J]. Aging Medicine and Healthcare, 2019, 10: 38.  [786] SHAFIEE G, HESHMAT R, OSTOVAR A, et al. THE RELATIONSHIP BETWEEN OSTEOSARCOPENIA AND COGNITIVE FRAILTY AMONG OLDER PEOPLE: THE BUSHEHR ELDERLY HEALTH (BEH) PROGRAM [J]. Aging Clinical and Experimental Research, 2022, 34: S325.  [787] SHANG N, MERAM C, BANDARA N, et al. Protein and Peptides for Elderly Health [J]. Adv Protein Chem Struct Biol, 2018, 112: 265-308.  [788] SHARMA A. Collaborative Nursing and Nutrition Intervention improves Unintentional Weight Loss at Emory Long Term Skilled Care Facility (Budd Terrace at Wesley Wood Campus) [J]. Journal of Parenteral and Enteral Nutrition, 2022, 46(SUPPL 1): S127-S8.  [789] SHAUGHNESSY K A, HACKNEY K J, CLARK B C, et al. A Narrative Review of Handgrip Strength and Cognitive Functioning: Bringing a New Characteristic to Muscle Memory [J]. J Alzheimers Dis, 2020, 73(4): 1265-78.  [790] SHETTY A K, UPADHYA R, MADHU L N, et al. Novel Insights on Systemic and Brain Aging, Stroke, Amyotrophic Lateral Sclerosis, and Alzheimer's Disease [J]. Aging and Disease, 2019, 10(2): 470-82.  [791] SHIBATA H, UCHIDA Y, KOBAYASHI S, et al. [Primary central nervous system vasculitis: a differential diagnosis of longitudinally extensive spinal cord lesion] [J]. Rinsho Shinkeigaku, 2020, 60(12): 857-60.  [792] SHIBUKAWA K, KAWAMATA M, SEKI S, et al. [The use of propofol combined with nitrous oxide and fentanyl in anesthetic management of a patient with mitochondrial encephalomyopathy] [J]. Masui, 2002, 51(8): 888-91.  [793] SHIMADA H, MAKIZAKO H, DOI T, et al. Impact of cognitive frailty on daily activities in older persons [J]. Alzheimer's and Dementia, 2016, 12(7): P991.  [794] SHIMADA H, MAKIZAKO H, TSUTSUMIMOTO K, et al. Cognitive frailtyand incidence of dementia in older persons [J]. Alzheimer's and Dementia, 2017, 13(7): P1182.  [795] SHIMIZU H, HIDETOMO T, TAKASHI T, et al. Galactosialidosis: Clinicopathological features of four autopsied patients [J]. Brain Pathology, 2019, 29: 50-1.  [796] SHIMIZU Y. Gut microbiota in common elderly diseases affecting activities of daily living [J]. World Journal of Gastroenterology, 2018, 24(42): 4750-8.  [797] SHINDO K, TSUNODA S, SHIOZAWA Z. [A case of Kearns-Sayre-Shy syndrome with abnormal signal intensity on MRI in cerebral white matter and brainstem] [J]. Rinsho Shinkeigaku, 1991, 31(5): 539-42.  [798] SHLISKY J, BLOOM D E, BEAUDREAULT A R, et al. Nutritional Considerations for Healthy Aging and Reduction in Age-Related Chronic Disease [J]. Advances in nutrition (Bethesda, Md), 2017, 8(1): 17-26.  [799] SIKARIYA K, BHARODIYA R, SHAH S, et al. Clinical spectrum of motor neuron disease at tertiary care hospital [J]. Annals of Indian Academy of Neurology, 2018, 21(SUPPL 2): S178-S9.  [800] SIMEONOVA P. eP247: Pathogenic variant in valosin-containing protein causing inclusion body myopathy associated with Paget disease of bone and frontotemporal dementia [J]. Genetics in Medicine, 2022, 24(3): S157.  [801] SINGH M, SINGH S P, YADAV D, et al. Targeted delivery for neurodegenerative disorders using gene therapy vectors: Gene next therapeutic goals [J]. Current Gene Therapy, 2021, 21(1): 23-42.  [802] SIPILä J O T. Adult-Onset Neuroepidemiology in Finland: Lessons to Learn and Work to Do [J]. Journal of Clinical Medicine, 2023, 12(12).  [803] SIRIRATNAM P, RODRIGUES E, MCLEAN C. IMMUNE MYOPATHY with PERIMYSIAL PATHOLOGY (IMPP) in A PATIENT with AN UNUSUAL CLINICAL PHENOTYPE and ANTI-MI-2 ANTIBODY [J]. BMJ Neurology Open, 2023, 5(suppl 1): A62.  [804] SMITH M. One good turn causes another [J]. Journal of General Internal Medicine, 2011, 26: S364.  [805] SOBREIRA-NETO M A, STELZER F G, GITAí L L G, et al. REM sleep behavior disorder: update on diagnosis and management [J]. Arquivos De Neuro-Psiquiatria, 2023, 81(12): 1179-93.  [806] SONE J. Neuronal intranuclear inclusion disease (NIID) [J]. Brain Pathology, 2019, 29: 55.  [807] SONE J, MORI K, HARUKI K, et al. Clinical presentation and diagnosis of neuronal intranuclear inclusion disease [J]. Journal of the Neurological Sciences, 2017, 381: 1017.  [808] SONE J, MORI K, KOIKE H, et al. Diagnostic flowchart of adult-onset neuronal intranuclear inclusion disease [J]. Neurology, 2017, 88(16).  [809] SORBERA L A, BOLóS J, SERRADELL N. Ibutamoren mesilate: Growth hormone secretagogue [J]. Drugs of the Future, 2006, 31(5): 390-9.  [810] SOUSA O V, AMARAL T F. Nutritional and functional status in survival of free-living mild Alzheimer's disease patients [J]. Journal of Cachexia, Sarcopenia and Muscle, 2017, 8(6): 1058-9.  [811] SOYSAL P, SMITH L, TAN S G, et al. Excessive daytime sleepiness is associated with an increased frequency of falls and sarcopenia [J]. European Geriatric Medicine, 2021, 12(SUPPL 1): S237.  [812] SPINELLI E G, AGOSTA F, CANU E, et al. Cognitive changes and white matter tract damage in the motor neuron disease spectrum [J]. European Journal of Neurology, 2014, 21: 64.  [813] STANGA S, BOIDO M, KIENLEN-CAMPARD P. How to build and to protect the neuromuscular junction: The role of the glial cell line-derived neurotrophic factor [J]. International Journal of Molecular Sciences, 2021, 22(1): 1-14.  [814] STANGA S, CARETTO A, BOIDO M, et al. Mitochondrial dysfunctions: A red thread across neurodegenerative diseases [J]. International Journal of Molecular Sciences, 2020, 21(10).  [815] STARDELI T, AFRANTOU T, XIROMERISIOU G, et al. Ubiquilin 2 gene mutation presenting with adult-onset ataxia and spasticity; report of a novel phenotype case [J]. European Journal of Neurology, 2020, 27: 962.  [816] STAUNTON C A, VASILAKI A, BARRETT-JOLLEY R, et al. Genomic profiling using RNAseq to identify key drivers of sarcopenia and motor unit turnover in murine skeletal muscle [J]. FASEB Journal, 2019, 33(SUPPL 1): 537.1.  [817] STENS O, NEUTEL B, GOODMAN E L. An Ounce of Prevention, a Pound of Complications: A Case of Statin-Induced Necrotizing Myopathy in a Frail Elderly Patient [J]. Geriatrics (Basel), 2022, 7(2).  [818] STETKAROVA I, EHLER E. Diagnostics of Amyotrophic Lateral Sclerosis: Up to Date [J]. Diagnostics, 2021, 11(2).  [819] STETKáROVá I, MATEJ R, EHLER E. New insights in the diagnosis and treatment of amyotrophic lateral sclerosis [J]. Ceska a Slovenska Neurologie a Neurochirurgie, 2018, 81(5): 546-54.  [820] STIEBLER M, MüLLER P, HALFPAAP N, et al. Dance against dementia (DiADEM): effects of a sportive dance training on cardiorespiratory and muscular fitness in seniors with mild cognitive impairment (MCI); proceedings of the International Conference on Technology Innovations for Healthcare (ICTIH), Magdeburg, GERMANY, F Sep 14-16, 2022 [C]. 2022.  [821] STUBBS B J, KOUTNIK A P, VOLEK J S, et al. From bedside to battlefield: intersection of ketone body mechanisms in geroscience with military resilience [J]. Geroscience, 2021, 43(3): 1071-81.  [822] SUGIHARA T, ISHIZAKI T, BABA H, et al. Three year outcomes of patients with elderly-onset rheumatoid arthritis treated with a therapeutic strategy targeting low disease activity, and impact of adverse events on physical function [J]. Arthritis and Rheumatology, 2018, 70: 600.  [823] SUGIMOTO T. Clinical impact and underlying mechanisms of frailty & sarcopenia among memory clinic populations [J]. Aging Medicine and Healthcare, 2019, 10: 7-8.  [824] SUGIMOTO T, ONO R, MURATA S, et al. Sarcopenia is Associated With Impairment of Activities of Daily Living in Japanese Patients With Early-Stage Alzheimer Disease [J]. Alzheimer Disease and Associated Disorders, 2017, 31(3): 256-8.  [825] SUI S X, WILLIAMS L J, HOLLOWAY-KEW K L, et al. Skeletal Muscle Health and Cognitive Function: A Narrative Review [J]. Int J Mol Sci, 2020, 22(1).  [826] SULERIA D, FLEURY O, GOLDSMITH D. Apathetic hyperthyroidism presenting with thyrotoxic periodic paralysis (TPP) [J]. Journal of General Internal Medicine, 2021, 36(SUPPL 1): S311.  [827] SUMATHIPALA D, STROMME P, GILISSEN C, et al. TBCK encephaloneuropathy with abnormal lysosomal storage: Use of a structural variant bioinformatics pipeline on WGS data unravels a 20-year clinical mystery [J]. European Journal of Neurology, 2019, 26: 889.  [828] SURAMPALLI A, GOLD B T, SMITH C, et al. A case report comparing clinical, imaging and neuropsychological assessment findings in twins discordant for the VCP p.R155C mutation [J]. Neuromuscul Disord, 2015, 25(2): 177-83.  [829] SURYADEVARA V, KLüPPEL M, DEL MONTE F, et al. The Unraveling <i>Cardiac and Musculoskeletal Defects and Their Role in Common Alzheimer Disease Morbidity and Mortality</i> [J]. American Journal of Pathology, 2020, 190(8): 1609-21.  [830] SURYADEVARA V, KLüPPEL M, MONTE F D, et al. The Unraveling: Cardiac and Musculoskeletal Defects and Their Role in Common Alzheimer Disease Morbidity and Mortality [J]. American Journal of Pathology, 2020, 190(8): 1609-21.  [831] SURYADEVARA V, SATO A, KAMBRATH A V, et al. Musculoskeletal and cardiac defects in the Presenilin-1 (PSEN1) L166P KI mouse model of Alzheimer Disease [J]. Journal of Bone and Mineral Research, 2020, 35(SUPPL 1): 156-7.  [832] SUZUKI K. Neurological disorders associated with impaired glucose tolerance [J]. Nippon rinsho Japanese journal of clinical medicine, 1996, 54(10): 2704-8.  [833] SUZUKI K. Chronic Inflammation as an Immunological Abnormality and Effectiveness of Exercise [J]. Biomolecules, 2019, 9(6).  [834] SUZUKI M, LEE S J, KIM H R. A longitudinal study on the preventive effects of dumbbell exercise and gum-chewing training on the sarcopenia and dementia in old people in Korea (2009-2019) [J]. FASEB Journal, 2012, 26.  [835] SWALLEY S E. Expanding therapeutic opportunities for neurodegenerative diseases: A perspective on the important role of phenotypic screening [J]. Bioorganic and Medicinal Chemistry, 2020, 28(3).  [836] SWART E L, VAN LOENEN A C. Levocarnitine, panacea for metabolic disturbances? [J]. Pharmaceutisch Weekblad, 1994, 129(24): 596-601.  [837] SZETO H H. The development of a therapeutic peptide for mitochondrial protection - From bench to bedside [J]. FASEB Journal, 2011, 25.  [838] TADA M, HATANO Y, TAKESHIMA A, et al. Clinicopathologic study of two patients with amyotrophic lateral sclerosis harboring TBK1 mutations [J]. Clinical Neurology, 2021, 61: S398.  [839] TAKAHASHI J, TAKAHASHI S, UTSUGIZAWA K, et al. [Two siblings with metachromatic leukodystrophy of adult and juvenile onset] [J]. Rinsho Shinkeigaku, 1993, 33(3): 312-6.  [840] TAKAHASHI M, AKAGI M. [Case report of sodium valproate treatment of aggression associated with Alzheimer's disease] [J]. No To Shinkei, 1996, 48(8): 757-60.  [841] TAKAYAMA K I. The biological and clinical advances of androgen receptor function in age-related diseases and cancer [J]. Endocrine Journal, 2017, 64(10): 933-46.  [842] TAKAYAMA K I. The biological and clinical advances of androgen receptor function in age-related diseases and cancer [Review] [J]. Endocr J, 2017, 64(10): 933-46.  [843] TAKAYAMA S, ARITA R, KIKUCHI A, et al. Clinical Practice Guidelines and Evidence for the Efficacy of Traditional Japanese Herbal Medicine (Kampo) in Treating Geriatric Patients [J]. Front Nutr, 2018, 5: 66.  [844] TAKEDA K. The voxel-based specific regional analysis system for Alzheimer's disease (VSRAD) by magnetic resonance imaging scan in peritoneal dialysis (PD) patients, compared with hemodialysis (HD) patients with Alzheimer's disease [J]. Nephrology Dialysis Transplantation, 2020, 35(SUPPL 3): iii1546.  [845] TAKEDA K, TOYONAGA J. The voxel-based specific regional analysis system for Alzheimer's disease (VSRAD) by magnetic resonance imaging scan in peritoneal dialysis (PD) compared with hemodialysis patients with cognitive impairment [J]. Nephrology Dialysis Transplantation, 2019, 34: a528-a9.  [846] TAKEDA M. The deterioration of ADL in the early AD patients would relate to the lower leg muscle weakness [J]. European Geriatric Medicine, 2014, 5: S101.  [847] TAKEDA T, UCHIHARA T, KAWAMURA S, et al. Olfactory dysfunction related to TDP-43 pathology in amyotrophic lateral sclerosis [J]. Clin Neuropathol, 2014, 33(1): 65-7.  [848] TAKESHITA H, YAMAMOTO K, TAKEDA S, et al. PATHOPHYSIOLOGICAL STUDY OF COGNITIVE FRAILTY USING A MOUSE MODEL OF ALZHEIMER'S DISEASE [J]. Journal of Hypertension, 2023, 41: e448-e9.  [849] TAKEUCHI R, TOYOSHIMA Y, TADA M, et al. Globular glial mixed four repeat tau and TDP-43 proteinopathy with motor neuron disease and frontotemporal dementia [J]. Amyotrophic Lateral Sclerosis and Frontotemporal Degeneration, 2015, 16: 74.  [850] TAN C F, KAKITA A, PIAO Y S, et al. Primary lateral sclerosis: a rare upper-motor-predominant form of amyotrophic lateral sclerosis often accompanied by frontotemporal lobar degeneration with ubiquitinated neuronal inclusions? Report of an autopsy case and a review of the literature [J]. Acta Neuropathol, 2003, 105(6): 615-20.  [851] TANAKA H, KOIKE R, TAKAHASHI H. An autopsy case of 62-year-old male with FTLD-MND and parkinsonism [J]. Brain Pathology, 2019, 29: 5.  [852] TANAKA H, TOYOSHIMA Y, TAKAHASHI H. Globular glial tauopathy (type II) clinically mimicking ALS [J]. Amyotrophic Lateral Sclerosis and Frontotemporal Degeneration, 2015, 16: 73-4.  [853] TANAKA K, WADA I, OKUNOMIYA T, et al. Dropped head syndrome that preceded the onset of dementia with Lewy bodies [J]. Clinical Neurology, 2014, 54: S19.  [854] TANJI H, TAKEDA A, TATEYAMA M, et al. [Progressive cerebellar ataxia and distal amyotrophy of Charcot-Marie-Tooth type with hyperglutamataemia:two sibling cases] [J]. Rinsho Shinkeigaku, 1995, 35(7): 793-7.  [855] TAOKA K, OKOSHI Y, HASEGAWA Y, et al. A non-radiation-containing, intermediate-dose methotrexate (MTX) regimen resulted in favorable survival without progression of dementia in elderly patients with primary central nervous system lymphoma (PCNSL) [J]. Blood, 2009, 114(22).  [856] TAY C L, ISHAK N H, ALI M F, et al. A malnourished post-stroke man with multi-morbidity and sarcopenia risk in a long-term stroke clinic: A case report [J]. Malays Fam Physician, 2023, 18: 12.  [857] TAY L, LEUNG B, LIM W S, et al. Sarcopenia in cognitively impaired older adults: The role of inflammation and endocrine dysregulation [J]. Annals of the Academy of Medicine Singapore, 2015, 44(10): S195.  [858] TAYEBI H, AZADNAJAFABAD S, MAROUFI S F, et al. Applications of brain-computer interfaces in neurodegenerative diseases [J]. Neurosurgical Review, 2023, 46(1).  [859] TEKAN U Y, AKSOY E, KARGIN F, et al. Acute respiratory failure in progressive neurological diseases: What is important for intensive care unit and long-term survival? [J]. European Respiratory Journal, 2016, 48.  [860] TESTA C M. Antisense Oligonucleotide Therapeutics for Neurodegenerative Disorders [J]. Current Geriatrics Reports, 2022, 11(3): 19-32.  [861] TESTI S, FERRARINI M, TAIOLI F, et al. Targeted next-generation sequencing (NGS) yield in lower motor neuron (LMN) syndromes [J]. Journal of the Peripheral Nervous System, 2019, 24: S43.  [862] THOMAS E A, D'MELLO S R. Complex neuroprotective and neurotoxic effects of histone deacetylases [J]. Journal of Neurochemistry, 2018, 145(2): 96-110.  [863] TIAN J, SHI J, MANN D M A. Cerebral amyloid angiopathy and dementia [J]. Panminerva Medica, 2004, 46(4): 253-64.  [864] TICINESI A, NOUVENNE A, CERUNDOLO N, et al. The interaction between Mediterranean diet and intestinal microbiome: relevance for preventive strategies against frailty in older individuals [J]. Aging Clin Exp Res, 2024, 36(1): 58.  [865] TICINESI A, TANA C, NOUVENNE A. The intestinal microbiome and its relevance for functionality in older persons [J]. Current Opinion in Clinical Nutrition and Metabolic Care, 2019, 22(1): 4-12.  [866] TINITIGAN R, SUH T. An interesting case of foot deformity and tremor in an elderly woman [J]. Journal of the American Geriatrics Society, 2011, 59: S24.  [867] TITUS M B, CHANG A W, OLESNICKY E C. Exploring the Diverse Functional and Regulatory Consequences of Alternative Splicing in Development and Disease [J]. Frontiers in Genetics, 2021, 12.  [868] TOGO K, UMEMURA A, OEDA T, et al. Spinal and bulbar muscular atrophy with dementia of frontallobe type [J]. Movement Disorders, 2015, 30: S289-S90.  [869] TOKUYAMA W, YAGISHITA S, RYO M, et al. Familial motor neuron disease with prominent onion-bulb-like structures and axonal swelling restricted to the spinal ventral root: autopsy findings in two siblings [J]. Neuropathology, 2010, 30(1): 61-70.  [870] TOMASZEWSKA K, BOMERT I, WILKIEWICZ-WAWRO E. Feline-assisted therapy: Integrating contact with cats into treatment plans [J]. Polish Annals of Medicine, 2017, 24(2): 283-6.  [871] TOMINAGA I, HATTORI M, KAïHOU M, et al. [Dementia and amyotrophy in Kufs disease. The adult type of neuronal ceroid lipofuscinosis] [J]. Rev Neurol (Paris), 1994, 150(6-7): 413-7.  [872] TORII S. A first Antisense Oligonucleotide (ASO) therapy, Nusinersen, for Spinal Muscular Atrophy [J]. Clinical Neurology, 2018, 58: S48.  [873] TOTH C, MARTINEZ J, ZOCHODNE D W. RAGE, diabetes, and the nervous system [J]. Current Molecular Medicine, 2007, 7(8): 766-76.  [874] TOYOSHIMA Y, TAN C F, KOZAKAI T, et al. Is motor neuron disease-inclusion dementia a forme fruste of amyotrophic lateral sclerosis with dementia? An autopsy case further supporting the disease concept [J]. Neuropathology, 2005, 25(3): 214-9.  [875] TRISTANI M, RIQUELME L. The tri-fold risk of malnutrition, dehydration, and dysphagia in persons with dementia: An assessment and management protocol [J]. Alzheimer's and Dementia, 2015, 11(7): P233.  [876] TROESCH B, EGGERSDORFER M, LAVIANO A, et al. Expert Opinion on Benefits of Long-Chain Omega-3 Fatty Acids (DHA and EPA) in Aging and Clinical Nutrition [J]. Nutrients, 2020, 12(9).  [877] TROJSI F, SORRENTINO P, SORRENTINO G, et al. Neurodegeneration of brain networks in the amyotrophic lateral sclerosis-frontotemporal lobar degeneration (ALS-FTLD) continuum: evidence from MRI and MEG studies [J]. Cns Spectrums, 2018, 23(6): 378-87.  [878] TRUJILLO E, DEL MAR TRUJILLO M. Early frailty syndrome in rhematoid arthitis: Screening using the frail scale [J]. Arthritis and Rheumatology, 2017, 69.  [879] TRUJILLO E, GARCIA-MARRERO M R, FUENTES M I, et al. Sarcopenia and early frailty syndrome in rheumatoid arthritis [J]. Annals of the Rheumatic Diseases, 2018, 77: 1343.  [880] TRUJILLO O, CASAR J C, GEJMAN R, et al. Multisystem proteinopathy with motor nerve conduction blocks [J]. Journal of Neuromuscular Diseases, 2016, 3: S149-S50.  [881] TRUONG D D, HARDING A E, SCARAVILLI F, et al. Movement disorders in mitochondrial myopathies. A study of nine cases with two autopsy studies [J]. Mov Disord, 1990, 5(2): 109-17.  [882] TSAO C Y. Cerebellar Ataxia, Vertical Supranuclear Gaze Palsy, Sensorineural Deafness, Epilepsy, Dementia, and Hallucinations in an Adolescent Male [J]. Seminars in Pediatric Neurology, 2014, 21(2): 106-8.  [883] TSENG B, KHANLOU N, SHIEH P. A very complicated patient with muscle weakness [J]. Journal of Clinical Neuromuscular Disease, 2017, 18: S7.  [884] TSUCHIYA K, MIYAZAKI H, AKABANE H, et al. MELAS with prominent white matter gliosis and atrophy of the cerebellar granular layer: a clinical, genetic, and pathological study [J]. Acta Neuropathol, 1999, 97(5): 520-4.  [885] TSUCHIYA K, OZAWA E, FUKUSHIMA J, et al. Rapidly progressive aphasia and motor neuron disease: a clinical, radiological, and pathological study of an autopsy case with circumscribed lobar atrophy [J]. Acta Neuropathol, 2000, 99(1): 81-7.  [886] TSUJI T. Rehabilitation for elderly patients with cancer [J]. Japanese Journal of Clinical Oncology, 2022, 52(10): 1097-104.  [887] TUENA C, PEDROLI E, TRIMARCHI P D, et al. Usability Issues of Clinical and Research Applications of Virtual Reality in Older People: A Systematic Review [J]. Frontiers in Human Neuroscience, 2020, 14.  [888] TULBǍ D, COZMA L, OLARU I, et al. The phenotypic spectrum of motor neuron disease [J]. Romanian Journal of Neurology, 2019, 18: 63.  [889] TUOHIMAA P, KEISALA T, MINASYAN A, et al. Vitamin D, nervous system and aging [J]. Psychoneuroendocrinology, 2009, 34 Suppl 1: S278-86.  [890] TURNER M R. Motor neuron disease: biomarker development for an expanding cerebral syndrome [J]. Clinical medicine (London, England), 2016, 16: s60-s5.  [891] UMEGAKI H. Sarcopenia and frailty in older patients with diabetes mellitus [J]. Geriatr Gerontol Int, 2016, 16(3): 293-9.  [892] UMEGAKI H, MAKINO T, HAYASHI T, et al. Cognitive finction, physical funciton, and body compositions in eldery with diabetes [J]. Neurodegenerative Diseases, 2017, 17: 1684.  [893] UMEGAKI H, MAKINO T, UEMURA K, et al. Cognitive dysfunction in prefrailty baseline analysis of toyota preventional intervention for cognitive decline and sarcopenia (TOPICS) study [J]. Alzheimer's and Dementia, 2017, 13(7): P1148.  [894] UMEMOTO G, FURUYA H. Management of Dysphagia in Patients with Parkinson's Disease and Related Disorders [J]. Internal Medicine, 2020, 59(1): 7-14.  [895] UNSAL P, GüNER OYTUN M, OZSUREKCI C, et al. Prevalence Of Nutrition Disorders And Nutrition Related Conditions In Older Patients With Alzheimer's Disease [J]. Clinical Nutrition ESPEN, 2023, 54: 689.  [896] UTO N S, AMITANI H, ATOBE Y, et al. Herbal Medicine Ninjin'yoeito in the Treatment of Sarcopenia and Frailty [J]. Front Nutr, 2018, 5: 126.  [897] UYAMA E, HIRANO T, ITO K, et al. Adult Chédiak-Higashi syndrome presenting as parkinsonism and dementia [J]. Acta Neurol Scand, 1994, 89(3): 175-83.  [898] VACCHIANO V, MASTRANGELO A, ZENESINI C, et al. High plasma p-tau181 levels in Amyotrophic Lateral Sclerosis: clinical-electrophysiological correlations and longitudinal trajectories [J]. Acta Myologica, 2023, 42: 73.  [899] VALERO T. Mitochondrial biogenesis: pharmacological approaches [J]. (1873-4286 (Electronic)).  [900] VAN GIAU V, AN S S, KIM S Y, et al. Genetic diagnosis of neurodegenerative disorders based on gene panels and primers by next-generation sequencing [J]. Alzheimer's and Dementia, 2016, 12(7): P152.  [901] VAN GIAU V, AN S S A, BAGYINSZKY E, et al. Next generation sequencing (NGS) gene panels and primers for studies on neurodegenerative disorders [J]. Toxicology and Environmental Health Sciences, 2015, 7(4): S32.  [902] VANDERWEYDE T, YOUMANS K, LIU-YESUCEVITZ L, et al. Role of stress granules and RNA-binding proteins in neurodegeneration: A mini-review [J]. Gerontology, 2013, 59(6): 524-33.  [903] VAQUERO-PINTO M N, MIRET-CORCHADO C, SANCHEZ-CASTELLANO C, et al. Prevalence of sarcopenia in very old hip fracture patients [J]. Osteoporosis International, 2017, 28: S490.  [904] VAQUERO-PINTO M N, SáNCHEZ-CASTELLANO C, MERELLO-DE MIGUEL A, et al. Characteristics of sarcopenic patients admitted for hip fracture [J]. European Geriatric Medicine, 2018, 9: S123.  [905] VARAN H D, CEKER E, CATALTEPE E, et al. Adductor pollicis muscle thickness as an anthropometric marker of ultrasonography based sarcopenia [J]. Clinical Nutrition ESPEN, 2023, 58: 470-1.  [906] VECCHIO C F, MARTELLINO C, ARICò I, et al. A CASE OF ALS AND PD: TWO SIDES OF THE SAME COIN? [J]. Neurological Sciences, 2022, 43: S110.  [907] VEGETO E, VILLA A, DELLA TORRE S, et al. The role of sex and sex hormones in neurodegenerative diseases [J]. Endocrine Reviews, 2020, 41(2): 273-319.  [908] VEMPARALA P, KRISHNAMURTHY M. Was Metformin the Culprit for This Lactic Acidosis? [J]. Journal of the Endocrine Society, 2020, 4: A768.  [909] VENTURA M T, CASCIARO M, GANGEMI S, et al. Immunosenescence in aging: Between immune cells depletion and cytokines up-regulation [J]. Clinical and Molecular Allergy, 2017, 15.  [910] VERGHESE J, AYERS E. Contribution of potentially modifiable risk factors of slow gait in the Health & Retirement Study [J]. Journal of the American Geriatrics Society, 2015, 63: S182.  [911] VERMA A, BRADLEY W G. Atypical motor neuron disease and related motor syndromes [J]. Seminars in Neurology, 2001, 21(2): 177-87.  [912] VESA J, SU H, WATTS G D, et al. Defective signaling pathways in VCP associated inclusion body myopathy (IBMPFD) [J]. Journal of Investigative Medicine, 2010, 58(1): 125.  [913] VIADER F. Amyotrophic lateral sclerosis: An emblematic neurodegenerative disease [J]. Bulletin De L Academie Nationale De Medecine, 2023, 207(3): 272-86.  [914] VICENTE DE SOUSA O L, FREITAS AMARAL T. Nutritional and functional factors associated with mild alzheimees disease community-dwelling older adults [J]. Clinical Nutrition, 2016, 35: S143-S4.  [915] VINA J, BORRAS C, SANCHIS-GOMAR F, et al. Pharmacological properties of physical exercise in the elderly [J]. Curr Pharm Des, 2014, 20(18): 3019-29.  [916] VISCHER U M, BAUDUCEAU B, BOURDEL-MARCHASSON I, et al. A call to incorporate the prevention and treatment of geriatric disorders in the management of diabetes in the elderly [J]. Diabetes and Metabolism, 2009, 35(3): 168-77.  [917] VISWAMBHARAN V, THANSEEM I, VASU M M, et al. miRNAs as biomarkers of neurodegenerative disorders [J]. Biomark Med, 2017, 11(2): 151-67.  [918] VOLPATO S, ABETE P, BELLELLI G, et al. Assessing sarcopenia in older hospitalized patients. Feasibility and prevalence estimates of the EWGSOP algorithm [J]. European Geriatric Medicine, 2015, 6: S93-S4.  [919] VON BANK H, KIRSH C, SIMCOX J. Aging adipose: Depot location dictates age-associated expansion and dysfunction [J]. Ageing Res Rev, 2021, 67: 101259.  [920] VON HAEHLING S, ANKER S D. Cachexia as a major underestimated and unmet medical need: facts and numbers [J]. Journal of Cachexia Sarcopenia and Muscle, 2010, 1(1): 1-5.  [921] WAITE S J, MAITLAND S, THOMAS A, et al. Sarcopenia and frailty in individuals with dementia: A systematic review [J]. Archives of Gerontology and Geriatrics, 2021, 92.  [922] WALLACE D C, LOTT M T, SHOFFNER J M, et al. Diseases resulting from mitochondrial DNA point mutations [J]. Journal of Inherited Metabolic Disease, 1992, 15(4): 472-9.  [923] WANG T, BARON M, TRUMP D. An overview of Notch3 function in vascular smooth muscle cells [J]. Progress in Biophysics & Molecular Biology, 2008, 96(1-3): 499-509.  [924] WATTS G D, THOMASOVA D, RAMDEEN S K, et al. Novel VCP mutations in inclusion body myopathy associated with Paget disease of bone and frontotemporal dementia [J]. Clin Genet, 2007, 72(5): 420-6.  [925] WAZA M, ADACHI H, KATSUNO M, et al. Modulation of Hsp90 function in neurodegenerative disorders: A molecular-targeted therapy against disease-causing protein [J]. Journal of Molecular Medicine, 2006, 84(8): 635-46.  [926] WEI S, NGUYEN T T, ZHANG Y, et al. Sarcopenic obesity: epidemiology, pathophysiology, cardiovascular disease, mortality, and management [J]. Frontiers in Endocrinology, 2023, 14.  [927] WEIHL C C, PESTRONK A, KIMONIS V E. Valosin-containing protein disease: Inclusion body myopathy with Paget's disease of the bone and fronto-temporal dementia [J]. Neuromuscular Disorders, 2009, 19(5): 308-15.  [928] WEST E C, WILLIAMS L J, CORNEY K B, et al. Is sarcopenia associated with anxiety symptoms and disorders? A systematic review and meta-analysis protocol [J]. BMJ Open, 2021, 11(11).  [929] WILLIS M S. Alzheimer's disease and the co-morbidities related to the musculoskeletal system: lessons from patients and animals [J]. JBMR Plus, 2019, 3.  [930] WILLIS M S. Alzheimer's Disease, heart failure, and musculoskeletal defects and their relationship to clinical co-morbidities [J]. FASEB Journal, 2020, 34(SUPPL 1).  [931] WILSON D, JACKSON T A, SAPEY E, et al. Is cognitive frailty “functional sarcopenia”? [J]. European Geriatric Medicine, 2016, 7: S133.  [932] WINTER J S, GAPINSKE M, WOODS W S, et al. Programmable exon skipping with adenine base editors [J]. Molecular Therapy, 2018, 26(5): 229-30.  [933] WOO J. 2013: That was the year that was [J]. Age and Ageing, 2014, 43(2): 152-6.  [934] WOO J, YU R, TANG N, et al. Telomere length is associated with decline in grip strength in older persons aged 65 years and over [J]. AGE, 2014, 36(5).  [935] WOOK KIM D, MOON Y. MUSCLE PROFILE AND PROGRESSION OF ALZHEIMER'S DEMENTIA: PREDICTORS OF POOR CLINICAL OUTCOME AND ROLE OF MUSCLE PROFILE, A 3-YEAR LONGITUDINAL STUDY [J]. Alzheimer's and Dementia, 2019, 15(7): P691-P2.  [936] WRZESIEŃ A, ANDRZEJEWSKI K, JAMPOLSKA M, et al. Respiratory Dysfunction in Alzheimer’s Disease—Consequence or Underlying Cause? Applying Animal Models to the Study of Respiratory Malfunctions [J]. International Journal of Molecular Sciences, 2024, 25(4).  [937] WRZESIEŃ A, ANDRZEJEWSKI K, JAMPOLSKA M, et al. Respiratory Dysfunction in Alzheimer's Disease-Consequence or Underlying Cause? Applying Animal Models to the Study of Respiratory Malfunctions [J]. Int J Mol Sci, 2024, 25(4).  [938] WU H, VAN MIERLO R, MCLAUCHLAN G, et al. POS-318 PREDICTIVE ABILITY OF CLINICAL ASSESSMENT TOOLS FOR DELIRIUM FOLLOWING HIP FRACTURE IN PATIENTS LIVING WITH CKD: INSIGHTS FROM A 7-YEAR PROSPECTIVE ANALYSIS [J]. Kidney International Reports, 2022, 7(2): S143.  [939] WU J M, YOUNG C, WANG P J, et al. Late infantile type neuronal ceroid lipofuscinosis: report of one case [J]. Zhonghua Min Guo Xiao Er Ke Yi Xue Hui Za Zhi, 1996, 37(5): 376-80.  [940] WU M Y, ZOU W J, LEE D, et al. APP in the Neuromuscular Junction for the Development of Sarcopenia and Alzheimer’s Disease [J]. International Journal of Molecular Sciences, 2023, 24(9).  [941] WU S B, MA Y S, WU Y T, et al. Mitochondrial DNA mutation-elicited oxidative stress, oxidative damage, and altered gene expression in cultured cells of patients with MERRF syndrome [J]. Molecular Neurobiology, 2010, 41(2-3): 256-66.  [942] WU W, YUAN S, TANG Y, et al. Effect of Exercise and Oral Niacinamide Mononucleotide on Improving Mitochondrial Autophagy in Alzheimer’s Disease [J]. Nutrients, 2023, 15(13).  [943] WURSTER C D, LUDOLPH A C. Antisense oligonucleotides in neurological disorders [J]. Therapeutic Advances in Neurological Disorders, 2018, 11.  [944] WYSOKIŃSKI A, SOBóW T, KŁOSZEWSKA I, et al. Mechanisms of the anorexia of aging—a review [J]. Age, 2015, 37(4).  [945] WYSOKIŃSKI A, SOBóW T, KŁOSZEWSKA I, et al. Mechanisms of the anorexia of aging-a review [J]. Age (Dordr), 2015, 37(4): 9821.  [946] WYTTENBACH A. Role of heat shock proteins during polyglutamine neurodegeneration: Mechanisms and hypothesis [J]. Journal of Molecular Neuroscience, 2004, 23(1-2): 69-95.  [947] XIE T, YE X, KANDUKURI L, et al. Burden among patients with progressive supranuclear palsy [J]. Movement Disorders, 2018, 33: S432-S3.  [948] YADAV M, SHARMA K, SINGH D. The Deleterious Health Effects of Aluminium: An Updated Review [J]. Bioscience Biotechnology Research Communications, 2021, 14(1): 55-65.  [949] YAMADA T, ICHIHARA K, UCHIHARA T, et al. Two autopsy cases of Alzheimer disease with little or no Lewy pathology in contradiction to clinical diagnosis of probable dementia with Lewy bodies [J]. Neuropathology, 2012, 32(3): 343.  [950] YAMAMURA Y, AKAMIZU H, HIRATA T, et al. Malignant lymphoma presenting with neoplastic angioendotheliosis of the central nervous system [J]. Clin Neuropathol, 1983, 2(2): 62-8.  [951] YAMANAMI A, UCHIHARA T, ENDO T, et al. An Autopsy case of amyotrophic lateral sclerosis with precedent clinical diagnosis of Alzheimer disease [J]. Neuropathology, 2010, 30(3): 325.  [952] YAMAZAKI M, IGARASHI H, HAMAMOTO M, et al. [A case of mitochondrial encephalomyopathy with schizophrenic psychosis, dementia and neuroleptic malignant syndrome] [J]. Rinsho Shinkeigaku, 1991, 31(11): 1219-23.  [953] YAN J, CALIENDO J, FECTO F, et al. FUS protein aggregates in sporadic inclusion body myositis [J]. Amyotrophic Lateral Sclerosis, 2009, 10: 121.  [954] YANAGISAWA T, SHIMIZU T, TANAKA K, et al. Brain atrophy in patients of amyotrophic lateral sclerosis after long-termsurvival with tracheostomy positive pressure ventilation [J]. Journal of the Neurological Sciences, 2017, 381: 720.  [955] YANG S, TIAN M, DAI Y, et al. Infection and chronic disease activate a brain-muscle signaling axis that regulates muscle performance [Z]. 2020.10.1101/2020.12.20.423533  [956] YANG Y, XIAO M, LENG L, et al. A systematic review and meta-analysis of the prevalence and correlation of mild cognitive impairment in sarcopenia [J]. Journal of Cachexia, Sarcopenia and Muscle, 2023, 14(1): 45-56.  [957] YEH C H, SHEN Z Q, LIN C C, et al. Rejuvenation: Turning Back Time by Enhancing CISD2 [J]. International Journal of Molecular Sciences, 2022, 23(22).  [958] YITBAREK G Y, ALTY J, LAWLER K, et al. Current evidence on the association of tongue strength with cognitive decline in older adults and the known risk factors of frailty, sarcopenia and nutritional health: a scoping review protocol [J]. Bmj Open, 2023, 13(10).  [959] YLIKALLIO E, AURANEN M, SHCHERBII M, et al. The variant p.g66v in CHCHD10 causes axonal Charcot-Marie-Tooth disease [J]. Journal of the Peripheral Nervous System, 2016, 21(3): 312.  [960] YOH K, IKEDA K, HORIE K, et al. Roles of Estrogen, Estrogen Receptors, and Estrogen-Related Receptors in Skeletal Muscle: Regulation of Mitochondrial Function [J]. International Journal of Molecular Sciences, 2023, 24(3).  [961] YOKOI F, YOSHIDA M, MIMURO M. A neuropathology of 77 year-old woman with familial frontotemporal lober degeneration type 2 associated with spinal arterio-venous fistula [J]. Neuropathology, 2011, 31(3): 326.  [962] YOKOTA T, MATSUNAGA T, FURUKAWA T, et al. [Familial spastic paraplegia with syndrome of continuous muscle fiber activity (Isaacs)] [J]. No To Shinkei, 1989, 41(6): 589-92.  [963] YOSHIDA S, SHIRAISHI R, NAKAYAMA Y, et al. Can Nutrition Contribute to a Reduction in Sarcopenia, Frailty, and Comorbidities in a Super-Aged Society? [J]. Nutrients, 2023, 15(13).  [964] YOSHIMATSU Y, SMITHARD D G. Aspiration pneumonia in the elderly: Is it clinically relevant? [J]. European Geriatric Medicine, 2022, 13: S54-S5.  [965] YU D, LI J, TAI H, et al. Neuronal intranuclear inclusion disease misdiagnosed as Parkinson's disease: a case report [J]. J Int Med Res, 2024, 52(3): 3000605241233159.  [966] YU-TAEGER L, KELP A, RIESS O, et al. Transgenic rat models for polyglutamine diseases [J]. Ceska a Slovenska Neurologie a Neurochirurgie, 2015, 78: 2S7.  [967] ZACH H, KOVACS G, AUFF E, et al. Compassionate use of doxycycline in gerstmann-straeussler-scheinker syndrome. Case report on a descendant of the original family [J]. Journal of the Neurological Sciences, 2013, 333: e112-e3.  [968] ZAIB S, JAVED H, KHAN I, et al. Neurodegenerative Diseases: Their Onset, Epidemiology, Causes and Treatment [J]. Chemistryselect, 2023, 8(20).  [969] ZAUNER K, WINDHAGER E. Dementia and nutrition - A brief overview [J]. Psychiatria Danubina, 2015, 27(4): 446-51.  [970] ZENG T, CHEN Y Q, HUANG H H, et al. Neuronal Intranuclear Inclusion Disease with<i> NOTCH2NLC</i> GGC Repeat Expansion: A Systematic Review and Challenges of Phenotypic Characterization [J]. Aging and Disease, 2024.  [971] ZHANG D, MENG H. PET/CT IMAGING AND IMMUNOHISTOCHEMISTRY (IHC) STUDY OF DIABETIC ENCEPHALOPATHIC RATS; proceedings of the International Conference on Biotechnology and Medical Science (BMS), Nanjing, PEOPLES R CHINA, F Apr 16-17, 2016 [C]. 2017.  [972] ZHANG F, NIU L, LIU X Y, et al. Rapid Eye Movement Sleep Behavior Disorder and Neurodegenerative Diseases: An Update [J]. Aging and Disease, 2020, 11(2): 315-26.  [973] ZHANG H, WU X, LIANG J, et al. Irisin, an exercise-induced bioactive peptide beneficial for health promotion during aging process [J]. Ageing Research Reviews, 2022, 80.  [974] ZHANG X, EDWARDS B J. Malnutrition in Older Adults with Cancer [J]. Current Oncology Reports, 2019, 21(9).  [975] ZHANG X, MENG Y, ZHANG W, et al. Diagnostic Values of Advanced Glycation End Products and Homocysteine in Patients with Alzheimer's Disease and Sarcopenia [J]. Computational and Mathematical Methods in Medicine, 2022, 2022.  [976] ZHANG Z, WU X, ZONG J, et al. The role of mitochondrial protein Coiled-Coil-Helix-Coiledcoil- Helix Domain 10 (Chchd10) in the pathogenesis of heart failure [J]. Obesity Facts, 2023, 16: 93.  [977] ZHOU Z D, SAW W T, TAN E K. Mitochondrial CHCHD-Containing Proteins: Physiologic Functions and Link with Neurodegenerative Diseases [J]. Molecular Neurobiology, 2017, 54(7): 5534-46.  [978] ZHU Z, REISER G. The small heat shock proteins, especially HspB4 and HspB5 are promising protectants in neurodegenerative diseases [J]. Neurochemistry International, 2018, 115: 69-79.  [979] ZUCCARO E, PIOL D, BASSO M, et al. Motor Neuron Diseases and Neuroprotective Peptides: A Closer Look to Neurons [J]. Frontiers in Aging Neuroscience, 2021, 13.  [980] ZUCCHELLI A, MANZONI F, MORANDI A, et al. The association between sarcopenia and delirium: Results from the nationwide multicentre Italian Delirium Day 2017 [J]. European Geriatric Medicine, 2021, 12(SUPPL 1): S185.  [981] ZYCH-TWARDOWSKA E, WAJGT A, LUBOS L, et al. [Parkinsonism-ALS-dementia complex: case report] [J]. Neurol Neurochir Pol, 2003, 37 Suppl 5: 189-96. |
