# Supplementary material for: Prevalence and correlation of sarcopenia with Alzheimer’s disease: A systematic review and meta-analysis
Source: PLoS One. 2025 Mar 3;20(3):e0318920. doi: 10.1371/journal.pone.0318920 (PMC11875368; doi:10.1371/journal.pone.0318920)
Supplement: S5 Table — (DOCX) [file pone.0318920.s010.docx]

**S5 Table. Collinearity between variables in sarcopenia prevalence.**

|  | AD | Sarcopenia | Muscle mass | Participants | Gender | Region | Age | Design |
| --- | --- | --- | --- | --- | --- | --- | --- | --- |
| AD | 1 | 0.163462 | -0.0416 | 0.360555 | 0.170346 | -0.05312 | 0.026236 | -0.17035 |
| Sarcopenia | 0.163462 | 1 | -0.0416 | -0.0416 | 0.170346 | -0.22429 | -0.09515 | 0.209657 |
| Muscle mass | -0.0416 | -0.0416 | 1 | 0.42 | -0.07559 | -0.2043 | 0.112504 | 0.075593 |
| Participants | 0.360555 | -0.0416 | 0.42 | 1 | -0.07559 | 0.289427 | -0.07828 | -0.47246 |
| Gender | 0.170346 | 0.170346 | -0.07559 | -0.07559 | 1 | -0.09652 | 0.171024 | 0.035714 |
| Region | -0.05312 | -0.22429 | -0.2043 | 0.289427 | -0.09652 | 1 | 0.306595 | -0.37001 |
| Age | 0.026236 | -0.09515 | 0.112504 | -0.07828 | 0.171024 | 0.306595 | 1 | 0.197447 |
| Design | -0.17035 | 0.209657 | 0.075593 | -0.47246 | 0.035714 | -0.37001 | 0.197447 | 1 |
