# Supplementary material for: Prevalence and correlation of sarcopenia with Alzheimer’s disease: A systematic review and meta-analysis
Source: PLoS One. 2025 Mar 3;20(3):e0318920. doi: 10.1371/journal.pone.0318920 (PMC11875368; doi:10.1371/journal.pone.0318920)
Supplement: S6 Table — (DOCX) [file pone.0318920.s011.docx]

**S6 Table. The adjusted OR and adjustment factors of the studies.**

| Study | Adjusted OR (95% CI) | Adjustment factors |
| --- | --- | --- |
| Zekeriya Ülger 2022 (b)* | 5.109 (2.002 - 13.033) | Age, sex, weight, height, and presence of comorbidities (hypertension, diabetes mellitus, coronary artery disease, hyperlipidemia, chronic renal disease, and hypothyroidism) |
| Fatma Sena Dost 2022 | 3.723 (1.740 - 7.968) | Age, sex, education years, age-adjusted Charlson Comorbidity Index, number of drugs, recurrent falls, and estimated glomerular filtration rate. |
| Xiaofen Weng 2023 (b) | 5.35 (1.27 - 22.46) | Age, gender, height, weight, smoking, drinking, hypertension, diabetes, education, low physical activity, mini-nutritional assessments, and Hamilton Depression Scale-17 |
| Veysel SUZAN 2022 | 2.048 (1.049 - 3.998) | Parkinson’s disease, polypharmacy, malnutrition, depression, delirium, insomnia, and urinary incontinence |
| Michal S. Beeri 2021 | 1.50 (1.20 - 1.86) | Age, sex, years of education, race, and height squared |
