# Supplementary material for: Prevalence and correlation of sarcopenia with Alzheimer’s disease: A systematic review and meta-analysis
Source: PLoS One. 2025 Mar 3;20(3):e0318920. doi: 10.1371/journal.pone.0318920 (PMC11875368; doi:10.1371/journal.pone.0318920)
Supplement: S7 Table — (DOCX) [file pone.0318920.s012.docx]

**S6 Table. Collinearity between variables in OR.**

|  | AD | Sarcopenia | Muscle mass | Study participants | Study region | Age | Study design |
| --- | --- | --- | --- | --- | --- | --- | --- |
| AD | 1 | 0.408248 | -1 | 0.408248 | 0.408248 | -0.00512 | -0.40825 |
| Sarcopenia | 0.408248 | 1 | -0.40825 | -0.25 | -0.25 | 0.567022 | 0.25 |
| Muscle mass | -1 | -0.40825 | 1 | -0.40825 | -0.40825 | 0.005116 | 0.408248 |
| Study participants | 0.408248 | -0.25 | -0.40825 | 1 | 1 | -0.85836 | -1 |
| Study region | 0.408248 | -0.25 | -0.40825 | 1 | 1 | -0.85836 | -1 |
| Age | -0.00512 | 0.567022 | 0.005116 | -0.85837 | -0.85837 | 1 | 0.858365 |
| Study design | -0.40825 | 0.25 | 0.408248 | -1 | -1 | 0.858365 | 1 |
